# Machine learning applied to simulations of collisions between rotating, differentiated planets

**DOI:** 10.1186/s40668-020-00034-6

**Published:** 2020-12-02

**Authors:** Miles L. Timpe, Maria Han Veiga, Mischa Knabenhans, Joachim Stadel, Stefano Marelli

**Affiliations:** 1 Institute for Computational Science, University of Zürich, Winterthurerstrasse 190, 8057 Zürich, Switzerland; 2 Institute for Mathematics, University of Zürich, Winterthurerstrasse 190, 8057 Zürich, Switzerland; 3 Department of Civil, Environmental and Geomatic Engineering, ETH Zürich, Stefano-Franscini-Platz 5, 8093 Zürich, Switzerland

**Keywords:** Emulation, Giant impacts, Machine learning, Neural network, Planet formation

## Abstract

In the late stages of terrestrial planet formation, pairwise collisions between planetary-sized bodies act as the fundamental agent of planet growth. These collisions can lead to either growth or disruption of the bodies involved and are largely responsible for shaping the final characteristics of the planets. Despite their critical role in planet formation, an accurate treatment of collisions has yet to be realized. While semi-analytic methods have been proposed, they remain limited to a narrow set of post-impact properties and have only achieved relatively low accuracies. However, the rise of machine learning and access to increased computing power have enabled novel data-driven approaches. In this work, we show that data-driven emulation techniques are capable of classifying and predicting the outcome of collisions with high accuracy and are generalizable to any quantifiable post-impact quantity. In particular, we focus on the dataset requirements, training pipeline, and classification and regression performance for four distinct data-driven techniques from machine learning (ensemble methods and neural networks) and uncertainty quantification (Gaussian processes and polynomial chaos expansion). We compare these methods to existing analytic and semi-analytic methods. Such data-driven emulators are poised to replace the methods currently used in N-body simulations, while avoiding the cost of direct simulation. This work is based on a new set of 14,856 SPH simulations of pairwise collisions between rotating, differentiated bodies at all possible mutual orientations.

**Supplementary Information:**

The online version of this article (10.1186/s40668-020-00034-6) contains supplementary material.

## Introduction

Pairwise collisions between planetary-size bodies are the primary agent of planet growth during the late stages of planet formation. These collisions—often called “giant impacts”—are violent events that result in either growth or disruption of the colliding bodies (Leinhardt and Stewart 2012; Stewart and Leinhardt 2012). Collisions shape nearly every aspect of a planet’s final characteristics, including its composition, thermal budget, rotation rate, and obliquity. Collisions can also determine whether a planet will retain an atmosphere, form satellites, or ultimately be hospitable to life. In addition to their role in planet formation, giant impacts have been suggested as explanations for a number of persisting mysteries in our own solar system, including the origin of Earth’s Moon (Benz et al. 1986; Canup and Asphaug 2001), Mercury’s large core (Benz et al. 1988; Chau et al. 2018), Uranus’ sideways tilt (Kegerreis et al. 2018), the martian hemispheric dichotomy (Wilhelms and Squyres 1984), the ice giant dichotomy (Reinhardt et al. 2019), Jupiter’s fuzzy core (Liu et al. 2019), and the Pluto-Charon system (Canup and Asphaug 2003).

Collisions play a central role in N-body studies of planet formation. Since the first N-body simulations were performed in the 1960s (von Hoerner 1960), the underlying numerical schemes have improved in leaps and bounds. Collisional N-body codes now routinely include 10^3^ massive particles,^1^ as well as general relativistic effects, gas dynamics (Morishima et al. 2010; Walsh et al. 2011), and the effect of external perturbations (Hands et al. 2019). However, despite these advances, the methodology for handling collisions between bodies has remained frustratingly primitive. Within N-body codes, a range of techniques for handling collisions can be employed. In the simplest, physically self-consistent case, collisions can be treated as perfectly inelastic mergers (PIM), whereby mass and momentum are conserved, but no fragmentation is possible. While efficient and easy to implement, the downside of PIM is that the outcomes are unphysical for all but a narrow subset of low-energy collisions. Despite its shortcomings, this is the technique that has been employed in the vast majority of N-body simulations to date.

At the other end of the spectrum, an ideal approach would be to simulate every collision using an accurate, high-resolution hydrodynamics code. This has recently been achieved in the context of volatile transfer (Burger et al. 2019). Unfortunately, such a hybrid approach is computationally prohibitive and adds significant complexity to the simulation. Moreover, because collisions must be evaluated sequentially in order to preserve self-consistency, the N-body integrator must remain idle while each collision is evaluated. This substantially increases the time required to complete a single N-body simulation. The problem is further compounded by the fact that, during a typical simulation of late-stage planet formation, the number of collisions can easily reach tens of thousands. This is a problem that will only grow more intractable as N-body codes improve and computing power increases, enabling ever larger numbers of bodies—and thus collisions—within N-body simulations.

In between these two extremes, a number of semi-analytic models have been developed in an effort to improve how collisions are handled within N-body simulations while keeping the computational overhead tractable. These semi-analytic models are derived from collision simulation datasets of varying size and complexity (Leinhardt and Richardson 2005; Leinhardt and Stewart 2012; Genda et al. 2017). One modern semi-analytic approach is the model known as EDACM (Leinhardt and Stewart 2012), which is a set of analytic relations derived from simulations of pairwise collisions between non-rotating gravitational aggregates (i.e., rubble piles) (Leinhardt and Richardson 2005). Whereas PIM is only able to predict limited properties of the largest (and only) remnant, EDACM allows for fragmentation (outcomes with more than one remnant) and is therefore able to predict limited properties of a second post-impact remnant and debris. Since its inception, EDACM has been implemented into the N-body codes Mercury (Chambers 1999, 2013) and pkdgrav (Stadel 2001; Bonsor et al. 2015) and used in several notable studies of terrestrial planet formation (Carter et al. 2015; Quintana et al. 2016). A simpler, but more recent semi-analytic approach is the impact-erosion model (IEM) for gravity-dominated planetesimals (Genda et al. 2017). IEM predicts the normalized debris mass and, from this value, implicitly predicts the mass of a single remnant. These models are a marked improvement, but the downside of such semi-analytic methods is that they are difficult to generalize beyond a narrow set of parameters and have in practice been able to achieve only modest accuracies, in some cases performing worse than PIM (see Table 6).

In recent years, the rise of machine learning and access to increasing computing power have enabled new data-driven approaches. Now, with sufficiently large datasets, surrogate models known as *emulators* can be trained to predict the outcome of collisions “on-the-fly” (i.e., within N-body simulations) (Cambioni et al. 2019). These emulators are lightweight enough to be integrated directly into existing N-body codes (Emsenhuber et al. 2020) and, once trained, can make near-instantaneous predictions of collision outcomes. In this paper, we show that they can far outperform existing analytic and semi-analytic methods. Nascent efforts to emulate collision outcomes have explored artificial neural networks (ANN) (Cambioni et al. 2019; Valencia et al. 2019). These studies have shown that simple ANNs can achieve high accuracy on relatively small datasets ($N=800$).

Machine learning techniques generally rely on the availability of large and well-sampled training datasets. Until recently, simulating such large collision datasets was computationally infeasible. However, computational fluid dynamics (CFD) algorithms and computing resources have advanced to the point where these datasets are now realizable. At the same time, recent improvements in CFD have opened the door to new dimensions in the collision parameter space. Collisions can now be simulated between differentiated bodies, rotating bodies, and bodies with arbitrary mutual orientations. In order to effectively sample these additional dimensions, even larger datasets are needed.

In this work we introduce a new dataset of 14,856 simulations of pairwise collisions between differentiated, rotating bodies. This dataset is larger than any previous dataset and includes effects not accounted for in similar studies, including the effects of pre-impact rotation and variable core mass fractions. These simulations were evaluated for an unprecedented number of post-impact parameters; in this work we investigate a subset of those parameters that are relevant to N-body studies of terrestrial planet formation.

In order to determine which numerical strategies are best suited to emulating collisions, we developed a flexible and robust machine-learning pipeline to train, optimize, and validate classification and regression models from different data-driven methodologies, including techniques from the field of uncertainty quantification (UQ) and machine learning (ML). In addition, the techniques were tested on a range of training dataset sizes, in order to provide constraints on dataset requirements for future studies.

The need to improve collision handling in N-body studies has often been dismissed in the literature, motivated by studies which have shown that the final number, masses, and orbital elements are barely affected by the collision method (Kokubo and Genda 2010). However, a number of more recent studies with improved collision models have overturned those conclusions. Indeed, studies with accurate collision handling have obtained profoundly different planetary system architectures, with a wider range of planetary masses and enhanced compositional diversity (Emsenhuber et al. 2020). Moreover, N-body simulations allowing for fragmentation have shown that roughly half of collisions occurring during planet formation are disruptive (Kokubo and Genda 2010) and, even within the non-disruptive regime, the effect of erosive collisions on planet growth has likely been underestimated or neglected (Inaba et al. 2003; Kobayashi and Tanaka 2010). Studies have also shown that the growth timescale of planets depends strongly on the collision model, in some cases increasing the growth timescale of the planets by a factor of two (Quintana et al. 2016). This has massive implications for the internal and atmospheric evolution of planets (Hamano and Abe 2010), their subsequent habitability, the formation of satellites (Elser et al. 2011), and even the likelihood of detecting giant impacts around other stars (Bonati et al. 2019).

We begin in Sect. 2 by describing the collision datasets that we generated and how each collision was set up, simulated, and analyzed. In Sect. 3, we give an overview of the emulation strategies used in this work and how they were evaluated. In Sect. 4 we report on the performance of the classification and regression models, their dependence on dataset size, and the associated sensitivity metrics. Finally, in Sect. 5, we discuss which techniques are best suited to emulating planetary-scale collisions, their relative ease (or complexity) of implementation, and where future work remains to be done.

## Dataset

### Methods

In order to train, test, and compare emulation strategies, a large number of collision simulations was required. In total, we simulated 14,856 collisions for this work. From the shuffled dataset, we reserved 20% ($N=2972$) as a holdout dataset for testing both the analytic and data-driven models. The remaining 80% ($N=11\text{,}884$) were used as a training dataset for the data-driven models. We additionally used a subset of 200 collisions (12D_LHS200) to study the convergence of the post-impact parameters.

The full dataset (all) is comprised of six individual datasets (Table 2), which are introduced in Sect. 2.1.2. Every collision in these datasets is uniquely defined by 12 pre-impact parameters (Sect. 2.1.1). The large number of dimensions in the parameter space necessitated an efficient sampling strategy, for which we employed Latin hypercube sampling (LHS) and the adaptive response surface method (ARSM) (Sect. 2.1.2).

23,768 unique planet models had to be generated to serve as either a target or projectile in the collisions (Sect. 2.1.3). These models were spun-up to their pre-impact rotation rates using a novel approach that we developed for this work (Sect. 2.1.4). Collisions were simulated using smoothed-particle hydrodynamics (SPH) (Sect. 2.1.5) and were subsequently evaluated for more than a hundred post-impact parameters (Sect. 2.1.6). These post-impact parameters were tested for convergence (Sect. 2.1.7) and a subset of these parameters was chosen to be investigated in this work on account of their relevance to N-body studies of terrestrial planet formation (Table 3).

#### Pre-impact conditions

Each collision is uniquely defined by 12 pre-impact parameters (Table 1). Together, these parameters define the geometry of the impact and the physical and rotational characteristics of the bodies involved in the collision. This set of parameters allows us to investigate the role of collisions in terrestrial planet formation, critically including the role of core mass fraction, rotation, and mutual orientation. The ranges of these parameters were chosen with two constraints in mind. First, the datasets should be focused on terrestrial planet formation. Second, and foremost for this work, the datasets should allow for a fair and robust comparison between distinct emulation strategies.

**Table 1:** Pre-impact parameters. Each collision in the dataset is uniquely defined by a set of 12 parameters. These parameters define the geometry of the collision and the physical characteristics, rotations, and orientations of the bodies involved in the collision. The subscripts ∞, *targ*, and *proj* refer to the asymptotic, target, and projectile values, respectively. The unit $R_{\mathrm{grav}}$ corresponds to maximum asymtotic impact parameter that will result in a collision

Parameter	Range	Unit	Description
$M_{\mathrm{tot}}$	0.1–2	$\mathrm{M}_{\oplus }$	Total mass ($M_{\mathrm{targ}} + M_{\mathrm{proj}}$)
*γ*	0.1–1	–	Mass ratio ($M_{\mathrm{proj}} \div M_{\mathrm{targ}}$)
$b_{\infty }$	0–1	$\mathrm{R}_{\mathrm{grav}}$	Asymptotic impact parameter
$v_{\infty }$	0.1–10	$\mathrm{v}_{\mathrm{esc}}$	Asymptotic impact velocity
$F^{\mathrm{core}}_{\mathrm{targ}}$	0.1–0.9	–	Target core mass fraction
$\Omega _{\mathrm{targ}}$	0–0.9	$\Omega _{\mathrm{crit}}$	Target rotation rate
$\theta _{\mathrm{targ}}$	0–180	deg	Target obliquity
$\phi _{\mathrm{targ}}$	0–360	deg	Target azimuth
$F^{\mathrm{core}}_{\mathrm{proj}}$	0.1–0.9	–	Projectile core mass fraction
$\Omega _{\mathrm{proj}}$	0–0.9	$\Omega _{\mathrm{crit}}$	Projectile rotation rate
$\theta _{\mathrm{proj}}$	0–180	deg	Projectile obliquity
$\phi _{\mathrm{proj}}$	0–360	deg	Projectile azimuth

In order to satisfy the first constraint, we simulated collisions with total masses ($M_{\mathrm{tot}}$) between 0.1–2 Earth masses, which is of interest to late-stage terrestrial planet formation. The ratio of projectile mass to target mass (*γ*) was allowed to range from 0.1 up to equal-mass collisions ($\gamma = 1$). The resulting models range in mass from roughly a lunar mass up to nearly twice that of Earth.

The bodies involved in the collisions—referred to in this work as the *target* and *projectile*—are fully differentiated planets composed of an iron core and granite mantle. The mass fraction of the core relative to the body’s total mass is defined by $F^{\mathrm{core}}_{\mathrm{body}}$, where the *body* subscript can refer to the target, projectile, largest post-impact remnant (LR), or second largest post-impact remnant (SLR). The core mass fractions of the target and projectile range from 0.1–0.9 (i.e., iron cores ranging from 10–90% by mass).

The target and projectile in the collisions are allowed to rotate. The rotation rates range from non-rotating to rotation at 90% the estimated breakup rate ($\Omega _{\mathrm{crit}}$). The estimated breakup rate is calculated according to Maclaurin’s formula for a self-gravitating fluid body of uniform density,

1 $$ \frac{\Omega _{\mathrm{crit}}^{2}}{\pi G \rho } = 0.449331, $$

where *G* is the gravitational constant and *ρ* is the bulk density of the body (Chandrasekhar 1969). Here, we calculate the bulk density of the body by using the mass and radius of the non-rotating model. Because the Maclaurin formula assumes a uniform density, the estimated breakup rate is more accurate for lower mass bodies and bodies with small core mass fractions. For high-mass bodies and those bodies with large core mass fractions, where the density profile strongly deviates from uniformity, the estimated breakup rate will be a lower bound. While the Maclaurin formula is a somewhat blunt approximation, it serves as a good estimate of the permissible rotation rates and therefore provides an upper limit for rotation rates in the pre-impact parameter space. We set the maximum rotation at 90% of the critical rate in order to avoid borderline unstable cases at lower masses. While it would be better to use empirically derived breakup rates for each model, such a study would require significant computational resources that were beyond the scope of this work.

The orientations of the target and projectile are uniquely defined by the obliquity (*θ*) and azimuth (*ϕ*) of their angular momentum vectors (i.e., rotation axes). These angles are allowed to vary between 0–180^∘^ and 0–360^∘^, respectively, where the obliquity is measured relative to the unit vector normal to the collision plane (*ẑ*) and the azimuth relative to a pre-defined reference direction (*ŷ*) in the collision plane. This allows for every possible mutual orientation between the target and projectile prior to impact.

In defining the pre-impact geometry of the collision, we depart from previous work by specifying the asymptotic impact parameter ($b_{\infty }$) and asymptotic relative velocity ($v_{\infty }$). In contrast, previous studies have generally used the associated quantities at the moment of impact ($b_{\mathrm{imp}}$ and $v_{\mathrm{imp}}$, respectively). However, this latter parameterization can result in unphysical initial conditions. Indeed, prior to impact, the mutual gravitational interaction between the target and projectile can alter their shapes, rotation rates, and relative orientations. This also alters the pre-impact trajectory and subsequent collision. This is due to the fact that both the target and projectile act as reservoirs of energy, whereby some fraction of the orbital energy in the pre-impact trajectory is transferred into the tidal deformation and rotational energy of the bodies. The simulations in this work therefore begin with the target and projectile separated by 10 critical radii, where the critical radius is given by $R_{\mathrm{crit}} = R_{\mathrm{targ}} + R_{\mathrm{proj}}$. Note that we use the *non-rotating* radii of the target and projectile in calculating the critical radius. This parameterization avoids the degeneracy introduced by arbitrary mutual orientations of rotating bodies. Indeed, rapidly rotating bodies can take on significantly oblate shapes, increasing their radii and making a clear definition of the critical radius problematic when the orientations are taken into account. With respect to the data-driven models, this parameterization is ideal because it does not introduce any additional colinearity into the pre-impact parameter space. A parameterization of $b_{\infty }$ that takes into account the orientations (*θ* and *ϕ*) and rotation rates (Ω) would introduce significant colinearity and was therefore avoided.

The parameter space investigated in this work is larger than any extant collision dataset known to the authors at the time of writing. Nonetheless, the parameter space is limited by computational resources and sampling requirements. It therefore does not yet include the full range of collisions relevant to planet formation, but does serve as a good training, test, and validation space for the emulators in this work. The emulation strategy developed in the work that follows easily allows for the parameter space to be expanded as computational resources become available.

#### Sampling strategy

In order to make a robust comparison between different emulation strategies, the underlying datasets must be well-sampled and well-behaved. However, generating a well-sampled training dataset in a high-dimensional parameter space is not a trivial task. The large number of dimensions quickly renders many approaches computationally infeasible. Indeed, a uniform grid sample would require $n^{d}$ simulations, where *d* is the number of dimensions and *n* is the desired number of samples in each dimension. A low resolution 12-dimensional dataset with 10 samples in each dimension would then require 10^12^ simulations, which is roughly eight orders of magnitude beyond current practical computational limits.

In order to overcome this problem while maintaining flexibility in the dataset requirements, we used a Latin hypercube sample (LHS) based version of the adaptive response surface method (LHS-ARSM) in order to sample a series of LHS (Wang 2003). Latin hypercube sampling is a statistical method for generating a near-random sample of parameter values from a *d*-dimensional distribution (McKay et al. 1979). LHS works on a function of *d* parameters by dividing each parameter into *n* equally probable intervals. The samples generated in this fashion are then distributed such that there is only one sample in each axis-aligned hyperplane. The advantage of this scheme is that it does not require additional samples for additional dimensions. LHS techniques have been used to considerable success in other high-dimensional astrophysical applications (Knabenhans et al. 2019).

In this study, the training dataset sizes required to reach optimal accuracies were not known *a priori*. Therefore, a procedure was needed to expand an existing dataset while maintaining certain properties, such as Latin hypercube, space-filling, and stratification properties. LHS-ARSM achieves this by sequentially generating sample points while preserving these distributional properties as the sample size grows. Unlike LHS, LHS-ARSM generates a series of smaller subsets that exhibit the following properties: the first subset is a Latin hypercube, the progressive union of subsets remains a LHS (and achieves maximum stratification in any one-dimensional projection), and the entire sample set at any time is a Latin hypercube. Benchmarking tests show that LHS-ARSM leads to improved efficiency of sampling-based analyses over older versions of ARSM (Wang 2003).

For the 12D_LHS10K dataset, we generated an initial LHS of 1000 collisions using the standard *maximin* distance criterion in order to guarantee space-filling properties. We then used LHS-ARSM to progressively enrich the sample in steps of 1000 collisions until we reached a total sample size of 10,000. We separately generated a 12D LHS sample of 500 collisions, designated 12D_LHS500, and a 12D LHS of 200 collisions designated 12D_LHS200. We subsequently used the 12D_LHS200 dataset to study the temporal convergence of the post-impact parameters, as a convergence study on the larger datasets was computationally infeasible.

In addition to the 12D datasets introduced above, we simulated two datasets of 500 collisions each, but with fewer dimensions. In the 6D_LHS500 and 4D_LHS500 datasets, the target and projectile are non-rotating, therefore fixing the rotational input parameters (Ω, *θ*, and *ϕ* for each body). In the 4D_LHS500 dataset, the core mass fractions of the target and projectile ($F_{\mathrm{targ}}^{\mathrm{core}}$ and $F_{\mathrm{proj}}^{\mathrm{core}}$, respectively) are additionally held constant at 0.33.

The 12D_LHS10K, 12D_LHS500, and 12D_LHS200, as well as the 6D_LHS500 and 4D_LHS500 datasets share the same parameter ranges (for those parameters that are varied) and therefore represent a composite sample in a shared parameter space. Superimposing LHS of the same dimension has the effect of increasing the resolution of the sample uniformly. However, superimposing LHS of different dimensions increases the resolution along specific hyperplanes of the higher dimension sample. Indeed, the resolution of the training dataset increases for values of the paramaters that are not varied in the lower dimension LHS (e.g., for core mass fractions of 0.33 in the case of 4D_LHS500).

While this causes the training dataset to deviate from truly uniform sampling, we found that the additional simulations provided a net improvement to the performance of our models. Moreover, the additional simulations provide increased resolution in the regions of the parameter space in which collisions are most likely to occur, which is a desirable feature in a training dataset. The choice to include the lower dimension LHS was therefore justified and tends to improve the performance of the models in the regions of the parameter space which are critical for planet formation.

In training the data-driven models in this work, it became clear that a large number of additional simulations were required at lower velocities. These additional collisions are necessary to sample both sides of the boundary between merging and hit-and-run collisions, which represents a relatively sharp discontinuity in the parameter space. We therefore simulated an additional 3384 simulations with asymptotic relative velocities between 0.1–1 $\mathrm{v}_{\mathrm{esc}}$. The number of simulations in this region was chosen to match the resolution in the parameter space at higher velocities.

The relatively large number of simulations at lower velocities is due to the increased gravitational focusing radius, which grows rapidly at these velocities. Because we sample the asymptotic impact parameter ($b_{ \infty }$) in units of critical radii ($R_{\mathrm{crit}}$), more simulations are required at low velocities in order to maintain the desired resolution. As with the superimposed samples at higher velocities, the resulting increase in resolution at lower velocities is desirable because this is the region of the parameter space where collisions are most likely to occur. Moreover, it has the intended benefit of increasing the resolution of the training dataset around the transition region between merging and hit-and-run, which is a difficult transition to capture.

The datasets used in this work are summarized in Table 2 and were combined to create a composite training dataset. This dataset was used to train and test the data-driven classification and regression models in the work that follows.

**Table 2:** Summary of the collision datasets in this work. Each simulation requires two unique models to serve as the target and projectile. In this work, we combined six distinct datasets to create a dataset of 14,856 collisions. The 12D_LHS200 dataset was additionally used to study the convergence of the post-impact parameters, as a convergence study the larger datasets was computationally infeasible. The 12D_LHSLOW dataset was simulated to study low asymptotic relative velocities from 0.1-1 $v_{\mathrm{esc}}$

Dataset	Type	Collisions	Models	$v_{\infty }$ ($\mathrm{v}_{\mathrm{esc}}$)
12D_LHS10K	ARSM	10,000	20,000	1–10
12D_LHS500	LHS	500	1000	1–10
12D_LHS200	LHS	200	400	1–10
12D_LHSLOW	LHS	3384	6768	0.1–1
6D_LHS500	LHS	500	1000	1–10
4D_LHS500	LHS	500	1000	1–10
all	Composite	14,856	29,712	0.1–10
train	Composite	11,884	23,768	0.1–10
test	Composite	2972	5944	0.1–10

#### Generating planet models

The collisions in this work are pairwise collisions between a target and projectile, where the target is the more massive of the two bodies. In order to simulate collisions between these bodies using a particle-based method such as SPH, we had to first create suitable particle representations (i.e., models) of each body. We used ballic (Reinhardt and Stadel 2017) to generate non-rotating, low-noise particle representations of each body. The ballic code solves the equilibrium internal structure equations using the Tillotson equation of state (EOS) and can generate models with distinct compositional layers. In this work we investigated fully differentiated two-layer bodies with iron cores and granite mantles.

#### Pre-impact rotation

**Figure 1:**
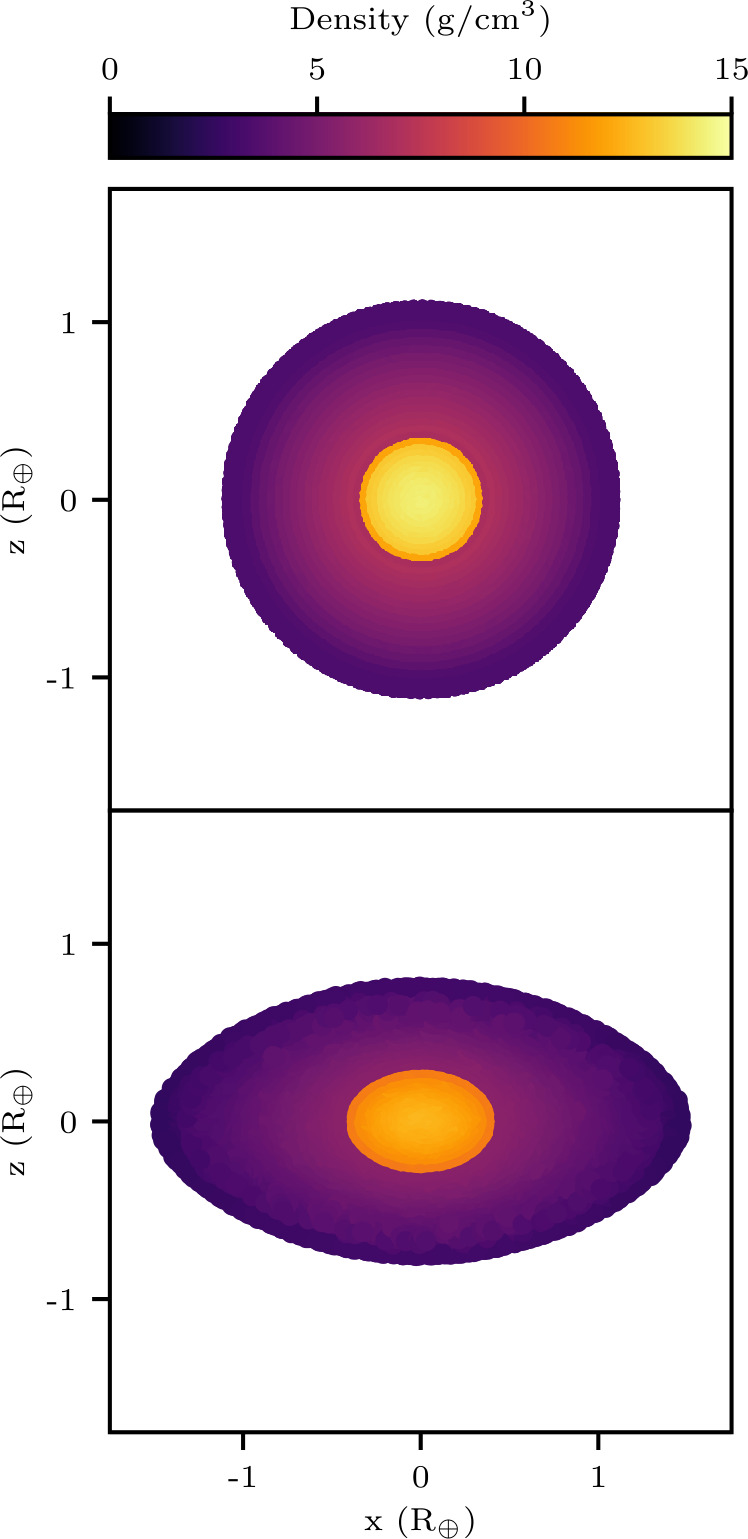
*Cross-section of a model*. The top panel shows the cross-section of a model in its non-rotating state as generated by ballic. In the bottom panel, a cross-section of that same model is shown in its rotating state after being spun-up by Gasoline. The model shown in this figure is designated YRMmYF in the 12D_LHS200 dataset and has the following properties: $M = 1.192~\mathrm{M}_{\oplus }$, $F^{\mathrm{core}}_{\mathrm{body}} = 0.122$, $\Omega = 0.869~\Omega _{\mathrm{crit}}$, $\epsilon _{\mathrm{body}} = 0.486$, and $\epsilon _{\mathrm{core}} = 0.2966$, where *ϵ* is the flattening. Note that the flattening of the core is less than that of the entire body

In order to facilitate collisions between rotating planets, we developed a method to induce rotation in the non-rotating models generated by ballic. The planets were first generated as non-rotating spherical models, after which a linearly increasing centrifugal force was applied to the particles in the rotating frame. The maximum centrifugal force applied to each particle is that which is required to achieve the desired rotation rate, $F_{c} = m_{p} r_{xy} \Omega ^{2}$, where $m_{p}$ is the particle mass and $r_{xy}$ is the particle’s distance from the rotational axis. Once the maximum centrifugal force has been reached, $F_{c}$ is held constant and the model is allowed to relax to a low-noise state. The particles are then transformed into the non-rotating frame and allowed to relax again. This method can spin-up a body up to its critical rotation rate (and beyond if not careful) and therefore allows us to probe collisions between rotating planets at any mutual orientation. An example of a model before and after the spin-up procedure is shown in Fig. 1. This represents a significant improvement over previous work, which has generally only considered collisions between non-rotating bodies.

#### Simulating collisions

The collisions in the datasets reported here have been simulated with Gasoline (Wadsley et al. 2004), a massively-parallel SPH code. The version of Gasoline used in this work has been modified specifically to handle planetary collisions and has been used in previous work to study the origin of the Moon, Mercury’s large core (Chau et al. 2018), and the ice giant dichotomy (Reinhardt et al. 2019). These modifications are described in detail in previous papers (Reinhardt and Stadel 2017; Reinhardt et al. 2019). Gasoline uses the Tillotson EOS (Tillotson 1962; Brundage 2013), which allows us to simulate collisions between differentiated planets with iron cores and granite mantles.

The resolution of the collisions in this work ranges from 20,000 to 110,000 particles. The resolution of each collision is set by the pre-impact mass ratio, whereby the smaller body (the projectile) is required to have $N_{\mathrm{proj}} = 10\text{,}000$ particles. The particle mass is constant and therefore the larger body (the target) has $N_{\mathrm{targ}} = 10\text{,}000/\gamma $ particles. The minimum mass ratio that we consider is $\gamma =0.1$ and therefore the maximum resolution is 110,000 particles.

The simulations used in this work were simulated at the Swiss National Supercomputing Center (CSCS) and are publicly available in the Dryad repository: 10.5061/dryad.j6q573n94.

#### Post-impact analysis

**Figure 2:**
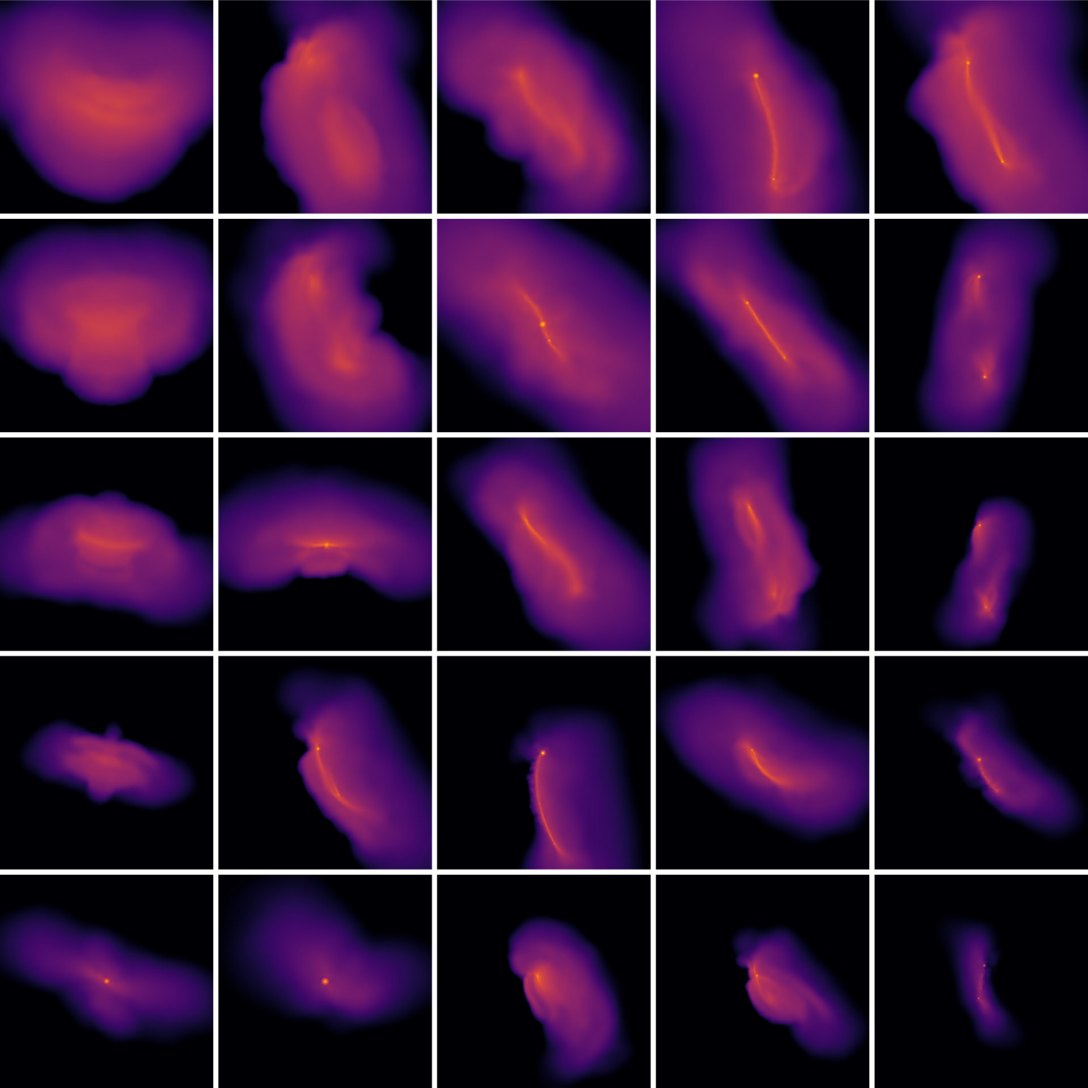
*Diversity of collision outcomes*. The images above show the outcomes for a subset of the collisions in the 12D_LHS200 dataset. The images are ordered by their impact geometry. From left to right, the impact parameter ($b_{\infty }$) increases from head-on ($b_{\infty } = 0$) to grazing impacts ($b_{\infty } \rightarrow 1$). From bottom to top, the relative asymptotic velocity increases ($v_{\infty }$). Thus, collisions near the top left are high-velocity, head-on impacts, whereas the collisions near the lower right are low-velocity, grazing collisions. Head-on, high velocity impacts are catastrophically disruptive to both the target and projectile, whereas grazing collisions tend to result in hit-and-run outcomes. At lower velocities, the target and projectile tend to merge and form a single remnant. In all collisions, debris is generated. The spatial distribution of this debris is strongly dependent on the collision geometry. Emulators must be able to accurately predict post-impact properties for a wide range of collision outcomes. The color scale indicates log-density

In this work, we consider a wide range of pre-impact conditions. This diversity of pre-impact conditions leads to a diverse set of post-impact states. The post-impact states for a subset of collisions in this work are shown in Fig. 2, wherein the collisions are roughly ordered by their pre-impact geometry. Collisions near the top left are high-velocity head-on impacts, whereas collisions near the bottom right are low-velocity grazing impacts. The range of collision outcomes required a robust script to retrieve the desired post-impact properties.

Every collision was evaluated for more than a hundred post-impact properties. We focus on a subset of these properties that are likely to prove important for N-body studies of terrestrial planet formation. These properties are listed in Table 3. In particular, we focus on the properties of the LR, SLR, and the debris field.

**Table 3:** Post-impact parameters. In this work we consider the following subset of post-impact parameters, focusing on the LR, SLR, and debris field. These parameters were chosen for their relevance to N-body studies of terrestrial planet formation. Detailed definitions of the post-impact parameters and how they are evaluated can be found in Appendix A

Parameter	Constraints	Unit	Description
*ξ*	−10–1	–	Accretion efficiency
$M_{\mathrm{LR}}$	0–$M_{\mathrm{tot}}$	$\mathrm{M}_{\oplus }$	Mass
$M^{\mathrm{norm}}_{\mathrm{LR}}$	0–1	$\mathrm{M}_{\mathrm{tot}}$	Normalized mass
$R_{\mathrm{LR}}$	>0	$\mathrm{R}_{\oplus }$	Radius
$F^{\mathrm{core}}_{\mathrm{LR}}$	0–1	–	Core mass fraction
$\Omega _{\mathrm{LR}}$	>0	Hz	Rotation rate
$\theta _{\mathrm{LR}}$	0 − 180	deg	Obliquity
$J_{\mathrm{LR}}$	0–$J_{\mathrm{tot}}$	J⋅*s*	Angular momentum
$F^{\mathrm{melt}}_{\mathrm{LR}}$	0–1	–	Melt fraction
$\delta ^{\mathrm{mix}}_{\mathrm{LR}}$	0–0.5	–	Mixing ratio
$M_{\mathrm{SLR}}$	0–$M_{\mathrm{tot}}$	$\mathrm{M}_{\oplus }$	Mass
$M^{\mathrm{norm}}_{\mathrm{SLR}}$	0–0.5	$\mathrm{M}_{\mathrm{tot}}$	Normalized mass
$R_{\mathrm{SLR}}$	>0	$\mathrm{R}_{\oplus }$	Radius
$F^{\mathrm{core}}_{\mathrm{SLR}}$	0–1	–	Core mass fraction
$\Omega _{\mathrm{SLR}}$	>0	Hz	Rotation rate
$\theta _{\mathrm{SLR}}$	0–180	deg	Obliquity
$J_{\mathrm{SLR}}$	0–$J_{\mathrm{tot}}$	J⋅*s*	Angular momentum
$F^{\mathrm{melt}}_{\mathrm{SLR}}$	0–1	–	Melt fraction
$\delta ^{\mathrm{mix}}_{\mathrm{SLR}}$	0–0.5	–	Mixing ratio
$M_{\mathrm{deb}}$	0–$M_{\mathrm{tot}}$	$\mathrm{M}_{\oplus }$	Mass
$M^{\mathrm{norm}}_{\mathrm{deb}}$	0–1	$\mathrm{M}_{\mathrm{tot}}$	Normalized mass
$F^{\mathrm{Fe}}_{\mathrm{deb}}$	0–1	–	Iron mass fraction
$J_{\mathrm{deb}}$	0–$J_{\mathrm{tot}}$	J⋅*s*	Angular momentum
$\delta ^{\mathrm{mix}}_{\mathrm{deb}}$	0–0.5	–	Mixing ratio
$\bar{\theta }_{\mathrm{deb}}$	−90–90	deg	Mean altitude
$\theta ^{\mathrm{stdev}}_{\mathrm{deb}}$	>0	deg	Stddev altitude
$\bar{\phi }_{\mathrm{deb}}$	0–360	deg	Mean azimuth
$\phi ^{\mathrm{stdev}}_{\mathrm{deb}}$	>0	deg	Stddev azimuth

Collisions were simulated for a time equal to 100 times the timescale of the collision (100*τ*). The collision timescale *τ* is equivalent to the crossing time of the encounter and is given by,

2 $$ \tau = \frac{2 R_{\mathrm{crit}}}{v_{\mathrm{imp}}} , $$

where $v_{\mathrm{imp}}$ is the velocity at *impact* (see Appendix A) and we reiterate that $R_{\mathrm{crit}}$ depends on the non-rotating radii of the colliding bodies.

In order to identify the post-impact LR, SLR, and debris field we used the SKID group finder (Stadel 2001). SKID identifies coherent, gravitationally bound clumps of material. It does this by identifying regions which are bounded by a critical surface in the density gradient (akin to identifying watershed regions). Then it removes the most unbound particles one-by-one from the resulting structure until all particles are self-bound. In this work, the minimum number of particles in a SKID clump was set to 10. This usually produces a much larger number of clumps than just the two that would correspond to the first and second largest remnants. For this reason the analysis routine checks if these clumps are further bound to either of the first or second largest clumps, if not, they are identified as part of the debris field of the collision.

In addition, in order to qualify as a remnant, the two largest SKID clumps are required to meet a minimum mass requirement. The largest clump only qualifies as the LR if its mass is greater than 10% of the target mass ($M_{\mathrm{LR}} > 0.1~M_{\mathrm{targ}}$). Similarly, the second largest clump only qualifies as the SLR if its mass is greater than 10% of the projectile mass ($M_{\mathrm{SLR}} > 0.1~M_{\mathrm{proj}}$). If one or both of the clumps does not meet the relevant mass requirement, then it is considered to be part of the debris.

A number of the post-impact properties investigated here do not have obvious definitions and require some explanation. We define or provide explanations for the post-impact parameters in Appendix A. In addition, we investigate the normalized masses to determine whether or not such normalization leads to improved regression performance for the data-driven techniques.

#### Convergence of post-impact parameters

**Figure 3:**
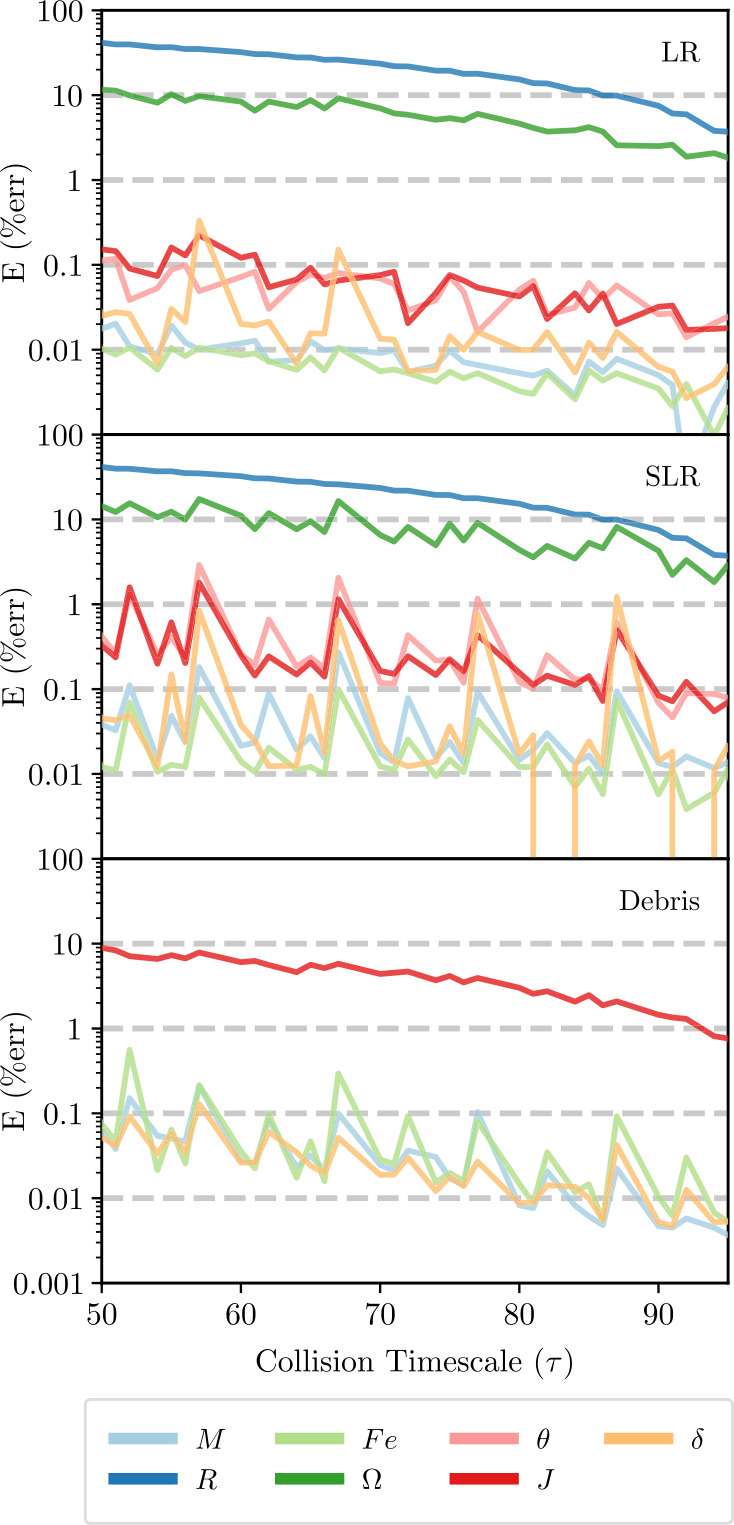
*Convergence of post-impact properties*. Here we show the convergence of the parameters in Table 3 for the 12D_LHS200 dataset. The median of the relative errors for each parameter are shown for uniformly spaced intervals of *τ*. Note that the radii, rotation rates, and angular momentum of the debris have not converged by 100*τ*. Future datasets may provide better convergence, however with the current data they are not suitable for training data-driven models. The remnant angular momenta converge quickly and are therefore better suited for studying rotation

We evaluated the convergence of all post-impact properties considered in this work (Table 3) using the 12D_LHS200 dataset.^2^ Convergence was measured relative to the post-impact quantity’s value at 100*τ* (the value used to train the emulators). In order to quantify the convergence, we calculated the absolute relative error *E* at uniformly sampled intervals of *τ*,

3 $$ E ( \tau ) = \frac{\lvert y ( \tau ) - y_{100} \rvert }{y_{100}}, $$

where $y(\tau )$ is the value of the post-impact parameter at *τ* and $y_{100}$ is the value used in the training dataset. For a single post-impact quantity, this yields 200 measurements of *E* at each evaluated step of *τ*. The median of these relative errors is plotted as a function of *τ* in Fig. 3.

Most post-impact parameters have converged to within 1% of their training value by 50*τ*, however the radii (*R*), rotation rates (Ω), and debris angular momentum ($J_{\mathrm{deb}}$) are still converging at 100*τ*. We note that the non-convergence of the radii and rotation rates is the result of both numerical considerations and ongoing physical processes post-impact (e.g., differentiation, thermal equilibration, etc.). The choice of EOS in the SPH simulations is thought to a significant role in the convergence of the post-impact radii and rotation rates, which are coupled. However, at the time of writing the magnitude of this effect is not well understood.

The debris field generally provides a much smaller reservoir for angular momentum than either the LR or SLR. Therefore, ongoing exchange of angular momentum with one or more massive remnants generally has a large effect on the debris angular momentum budget, while at the same time having a negligible effect on the LR and SLR angular momenta. These properties (i.e., the post-impact radii and debris angular momentum) are therefore not suitable for training data-driven methods on account of their non-convergence. Future datasets based on longer simulations are required to determine the convergence timescale of these properties and may subsequently allow these parameters to be used in emulation tasks.

In order to track the rotation of planets during N-body simulations, a substitute for the rotation rate is therefore needed. We investigated the convergence of the rotational angular momenta of the remnants ($J_{\mathrm{LR}}$ and $J_{\mathrm{SLR}}$) and found that they converge quickly following the impact. Indeed, following an impact, the angular momentum is quickly partitioned between the surviving bodies and has largely converged within a few tens of *τ*. While the debris angular momentum does not show the same convergence, it can instead be calculated implicitly from the angular momenta of the remnants and initial total angular momentum. We therefore suggest that N-body studies should utilize the angular momenta budget of the remnants to track rotation, rather than the rotation rates themselves.

## Emulation strategies

In order to overcome the limitations of analytic and semi-analytic approaches, techniques from the field of ML have proved promising (Cambioni et al. 2019). Techniques from UQ have also achieved considerable success in other areas of astrophysics investigating high-dimensional emulation (Knabenhans et al. 2019) (hereafter we refer to ML and UQ as “data-driven” techniques). These techniques can provide accurate and efficient strategies for emulating collisions. Data-driven methods have the major advantage of being generalizable to any quantifiable post-impact property, whereas analytic prescriptions are difficult to expand beyond a narrow set of properties. In order to identify the emulation methods best suited to the problem at hand, we have evaluated and compared the ability of several distinct classification and regression techniques to accurately classify and predict the post-impact properties of planetary-scale collisions.

To predict the outcome of a collision, an emulation pipeline must first classify each collision into certain regimes. These regimes can be as coarse or granular as desired, but at very least the collisions must be distinguished by their number of post-impact remnants. This is necessary to determine which regression models are called in the subsequent emulation step and ensures that regression models are not asked to make out-of-sample predictions (or at least minimizes such cases). Because we consider a maximum of two post-impact remnants in this work—designated the LR and SLR—our classifier must classify collisions into the following classes: 0 (no remnants), 1 (one remnant; the LR), or 2 (two remnants; the LR and SLR). In all collisions, debris is produced. Once the classification step has predicted which remnants, if any, exist, the regression models are called on to predict the properties of the existing remnant(s) and debris.

The regression techniques that we consider in this work are polynomial chaos expansion (PCE), Gaussian processes (GP), eXtreme Gradient Boosting (XGB), and multi-layer perceptrons (MLP). The latter two techniques—XGB and MLP—are additionally used in the classification step. We compare these data-driven techniques to the the most commonly employed analytic model, perfectly inelastic merging (PIM), as well as two more advanced semi-analytic techniques, the impact-erosion model (IEM) (Genda et al. 2017) and EDACM (Leinhardt and Stewart 2012).

In discussing the training and validation of the classification and regression models in this work, we adopt the terminology used in ML literature to describe the models, their parameters, and their associated input and output. In particular, we refer to the pre-impact parameters as *features*, the process of selecting these features as *feature selection*, and the analysis of the relationships between pre- and post-impact properties as *feature importance*. The meta-parameters that define the architectures and numerical behavior of the models are referred to as *hyperparameters*, and the process of selecting an optimal set of hyperparameters is known as *hyperparameter optimization* (HPO). The post-impact quantities that we are attempting to predict would usually be referred to as *targets* in this terminology. However, in order to avoid confusion with the target body involved in the collision, we simply refer to them as *post-impact quantities/properties*.

### Emulation pipeline

The emulation pipeline is comprised of two distinct stages: classification and regression. In the first stage, a classifier is used to predict how many post-impact remnants are produced by the collision (0, 1, or 2). In the second stage, a set of single-target regressors are used to predict the post-impact properties of the debris and existing remnants.

#### Classification stage

A classification step is necessary to determine which post-impact properties need to be predicted by the regression models. The classification step must therefore determine which post-impact remnants are produced by the collision. In this work, we consider at most two post-impact remnants; the resulting classes are 0 (no remnants), 1 (one remnant; the LR), and 2 (two remnants; the LR and SLR).

We consider two distinct strategies for classifying the number of post-impact remnants. In the first strategy, we first use a binary classification model to predict whether or not the LR exists. If it does not, the collision is assigned a label of 0 (no remnants). If it does exist, a second binary classification model is called on to predict whether or not the SLR exists. If it does not, the collision belongs to class 1 (LR only). If the SLR is predicted to exist, then the collision belongs to class 2 (LR and SLR). We refer to this strategy as *sequential binary classification*. This strategy requires two classification models.

In the second strategy, known as *multiclass classification*, a single classification model is used to predict the number of post-impact remnants directly (i.e., either 0, 1, or 2). For both strategies, we test both MLP and XGB classification models.

#### Regression stage

The approach to collision emulation introduced here produces a single classifier and a set of single-target regression models, whereby each regression model is optimized for a specific post-impact property. With this strategy, the regression models are simple and achieve optimal accuracy for each individual post-impact property. However, the drawback of decoupling the post-impact quantities from one another is that the resulting regression models are agnostic to the underlying physical relationships and constraints between the quantities (e.g., mass conservation). It’s therefore not guaranteed that the emulator predictions will be physically self-consistent. In this paper, we focus on comparing the accuracy of regression strategies and in a forthcoming paper, we introduce a method for imposing physical constraints and self-consistency on the regression models.

### Analytic & semi-analytic methods

The following sections introduce the analytic and semi-analytic methods considered in this work. The PIM model is an extremely simplified analytic prescription, but has been used in most N-body simulations to date. The semi-analytic models were developed on much simpler datasets than the one against which they are evaluated in this work. These datasets did not include variable core mass fractions, rotation, or orientations, however we evaluate them on our dataset to show that they are not able to generalize to include these effects and should therefore be replaced by data-driven methods.

#### Perfectly Inelastic Merging (PIM)

PIM is an analytic method in which all collisions are treated as perfectly inelastic mergers.^3^ In a perfectly inelastic merger, the masses and momenta of the colliding bodies are conserved in a single post-impact remnant. There is no net conversion of kinetic energy into other forms such as heat, noise, or potential energy during the impact. This is the simplest possible model for emulating the outcome of a pairwise collision while maintaining physical self-consistency (but not accuracy).

The outcome of a perfectly inelastic merger is always a single remnant, which we refer to here as the LR for consistency. There are no additional remnants or debris. PIM can predict the mass and core mass fraction of the LR, and can additionally make naïve predictions of certain rotational parameters for the LR. PIM has been employed in the vast majority of N-body simulations to date. Details of our implementation of PIM can be found in Appendix B.

#### Genda et al. (2017) (IEM)

The impact-erosion model (IEM) is a semi-analytic model for gravity-dominated planetesimals (Genda et al. 2017). IEM predicts the normalized mass of the debris ($M^{\mathrm{norm}}_{\mathrm{deb}}$) as a function of the specific impact energy ($Q_{R}$) scaled to the catastrophic disruption threshold ($Q^{\prime \star }_{\mathrm{RD}}$). The normalized mass of the debris $M^{\mathrm{norm}}_{\mathrm{deb}}$ is expressed as,

4 $$ M^{\mathrm{norm}}_{\mathrm{deb}} = 0.44 \phi \max (0, 1-\phi ) + 0.5 \phi ^{0.3} \min (1, \phi ), $$

where $\phi = Q_{R} / Q^{\prime \star }_{\mathrm{RD}}$. IEM assumes that only a single remnant is produced by the collision (referred to as the LR for consistency) and therefore $M^{\mathrm{norm}}_{\mathrm{LR}}$ can be determined via a straightforward relation, $M^{\mathrm{norm}}_{\mathrm{LR}} = 1 - M^{\mathrm{norm}}_{\mathrm{deb}}$. For consistency, we use the same values of $Q_{R}$ and $Q^{\prime \star }_{\mathrm{RD}}$ in the calculations of IEM and EDACM. Details of the calculation of $Q_{R}$ and $Q^{\prime \star }_{\mathrm{RD}}$ used here and in EDACM can be found in Appendix C.

#### Leinhardt and Stewart (2012) (EDACM)

EDACM is a set of analytic relations that predict the masses of the LR, SLR, and debris, as well as the core mass fraction of the LR (Leinhardt and Stewart 2012) via a mantle stripping law (Marcus et al. 2010). In order to evaluate and compare the performance of EDACM to the other emulators developed in this work, we implemented EDACM as prescribed in Leinhardt and Stewart (2012). EDACM has been used in several recent N-body studies of planet formation (Carter et al. 2015; Quintana and Lissauer 2017). Most notably, EDACM allows for collision outcomes with more than one remnant (referred to as *fragmentation*) and is thus capable of predicting a larger set of post-impact parameters than either PIM or IEM. We give a brief overview of EDACM in Appendix C and explain where our implementation differs from that used in previous studies.

### Data-driven methods

The analytic and semi-analytic models presented in the preceding section express an relatively simple relationships, based on naive physical assumptions (perfect merging) or fits to empirical data (IEM and EDACM). In contrast, the data-driven models that follow use machine learning algorithms to construct an approximate mapping between the pre-impact properties and individual post-impact properties. These non-linear mappings are derived purely from a training dataset of collision simulations.

#### Polynomial chaos expansion (PCE)

PCE is a popular technique in the field of UQ, where it is typically used to replace a computable-but-expensive computational model with an inexpensive-to-evaluate polynomial function (Ghanem and Spanos 1991). In this work, we use a PCE based on tensor products of Legendre polynomials (Benner et al. 2017). Recent work has demonstrated that data-driven PCE models can yield point-wise predictions with accuracies comparable to that of other machine learning regression models (e.g., neural networks) (Torre et al. 2019). In this work, we use UQLab (Marelli and Sudret 2014) to train and evaluate all PCE models. The documentation for UQLab is freely available at https://www.uqlab.com/documentation. An overview PCE as used in this work is provided in Appendix D.

#### Gaussian processes (GP)

GPs are a generic supervised learning method designed to solve regression and probabilistic classification problems (Rasmussen and Williams 2005). They are a non-parametric method that finds a distribution over the possible functions $f(x)$ that are consistent with the observed data. ML algorithms that involve a GP use a measure of the similarity between points (the kernel function) to predict a value for an unseen point from training data. The Gaussian radial basis function (RBF) kernel is commonly used, however in this work we test multiple kernels, including the constant, Matérn ($\nu =3/2$), rational quadratic, and RBF kernels (see Table 4).

**Table 4:** Summary of hyperspaces for the data-driven models investigated in this work. For the GP, MLP, and XGB models, the optimization algorithm (see Sect. 3.5) searches these spaces over 100 iterations to identify the most performant hyperparameter set for each model

Method	Hyperparameter	Range
MLP	Number of layers	∈{1,2,3}
	Neurons per layer	∈{1,2,…,24}
GP	Kernel	Constant, Matérn 3/2, rational quadratic, radial-basis functions
	Noise (*α*)	∈[0,10^−2^]
	Kernel restart	∈{0,1,…,5}
XGB	Number of estimators	∈{1,10,…,1000}
	Maximum tree depth	∈{3,4,…,12}
	Column subsample ratio	∈{0.5,…,1}
PCE	Polynomial order	∈{2,3,…,15}
	*q*-norm	∈{0.5,0.6,…,1.0}
	Maximum interaction	∈{2,3,…,5}
	Feature importance	=0.01

A potential downside of GPs is that they are not sparse (i.e., they use all of the sample and features information to perform the prediction) and they lose efficiency in high dimensional spaces (Rasmussen and Williams 2005). While our 12-dimensional space is relatively small for GPs, the number of training examples is much larger than that for which GPs are generally employed. More advanced algorithms have been suggested to improve the scaling of GPs, such as bagging and enforced sparsity, but we have not attempted to implement these here. A brief mathematical introduction to GPs is provided in Appendix E.

#### eXtreme Gradient Boosting (XGB)

XGBoost (XGB) is an open-source decision-tree-based ensemble ML algorithm that uses a gradient boosting framework (Chen and Guestrin 2016). It has become one of the most popular ML techniques in the previous years and is well documented. Gradient boosting is a machine learning technique for regression and classification problems which produces a prediction model in the form of an additive expansion of simple parameterized functions *h* (typically called *weak* or *base learners*) (Friedman 2001). These base learners are usually simple classification and regression trees (CART). In gradient boosting, the base learners are generated sequentially in such a way that the present base learner is always more effective than the previous one. Thus, the overall model improves sequentially with each iteration. A detailed overview of the XGB models used here is available in Appendix F.

#### Multi-Layer Perceptron (MLP)

MLPs are a class of feed-forward deep neural network that consist of multiple, fully-connected (i.e., dense) hidden layers. In MLPs, the mapping *f* between the pre- and post-impact parameters is defined by a composition of functions $g_{1}, g_{2}, \ldots, g_{n}$ (*n* being the number of layers in the network), yielding,

5 $$ f(\vec{x}) = g_{n} \bigl( \cdots g_{2} \bigl( g_{1} (\vec{x} ) \bigr) \bigr) , $$

where each function $g_{i}(w_{i},b_{i},h_{i}(\cdot ))$ is parameterized by a weights matrix ($w_{i}$), a bias vector ($b_{i}$), and an activation function ($h_{i}(\cdot )$). The weights matrix and bias vector are the parameters of the network that are tuned by minimizing a loss function which measures how well the mapping *f* performs on a given dataset. In this work, the MLPs are implemented with Python’s Keras library and models consist of an input layer with 12 nodes, one to three hidden layers with up to 24 nodes each, and an output layer with a single node (i.e., a scalar output). All activation functions in the resulting network are the Rectified Linear Unit (ReLU). A detailed overview of the MLPs used in this work is provided in Appendix G.

### Data pre-processing

Prior to training the classfication and regression models, a number of transformations are applied to the pre- and post-impact quantities. For regression tasks, these transformations ensure that the training data is well-defined (i.e., undefined values are removed). For classification tasks, the transformations encode either binary or multiclass labels. In both cases, the transformations generally improve training efficiency and performance. We describe these transformations here.

#### Classification

In order to provide training and test labels for the classification models, we encode collision outcomes as integers. These labels depend on whether the model is a binary classifier or multiclass classifier. In binary classification, the labels encode whether the remnant (LR or SLR, depending on the task) exists or not, whereby the labels are 0 (does not exist) or 1 (exists). In multiclass classification, the outcomes are encoded as 0, 1, or 2, where the label corresponds directly to the number of post-impact remnants. These labels are defined for all collisions and the classification models will therefore always leverage the full training dataset.

#### Regression

Of the post-impact properties that we consider in this work, the mass and angular momentum properties are always defined as either zero (in the case where the associated remnant doesn’t exist) or a finite number. However, in the case of all other post-impact properties, the property’s value will be undefined if the associated remnant does not exist. Therefore, before training the regression models, it is necessary to first remove entries from the dataset where the target value is undefined.

Undefined entries occur when either the LR or SLR was not produced by a collision. This is often the case in head-on, high-velocity impacts, after which only debris is present, and in the case of mergers, in which no SLR survives. Because collision outcomes with an LR are more common than those with both an LR and SLR, the resulting training and test set sizes for the regression models will differ between LR, SLR, and debris properties. The training set size will therefore be largest for debris (which is produced in all collisions) properties, smaller for LR properties, and smallest for SLR properties. However, to reiterate, this is only the case for properties not related to the mass or angular momentum, which are defined in all cases.

#### Standardization

In both classification and regression tasks, the pre-impact quantities are standardized to improve training efficiency and performance. For regression tasks, the post-impact quantities are standardized in the same manner as the pre-impact quantities.

The procedure for standardizing the input data differs between PCE and the other data-driven methods. In the case of PCE, the input parameters are linearly mapped into a hypercube $[-1,1]^{12}$, within which the distribution of the transformed features is still uniform.

For the other methods, the pre- and post-impact parameters are scaled using the *standard scaling* method. The result of standardization (a.k.a. Z-score normalization) is that the features will be rescaled such that they evince the properties of a standard normal distribution, $\mu = 0$ and $\sigma = 1$, where *μ* and *σ* are the mean and standard deviation of the distribution, respectively. The *z*-values are then calculated as,

6 $$ z = \frac{x - \mu }{\sigma } . $$

Standardization is a general requirement for many ML algorithms. The only family of algorithms that are scale-invariant are tree-based methods (e.g., XGB). However, since we are comparing several different ML algorithms here, some of which depend strongly on standardization, we standardize the input and output features for all techniques (except as noted above for PCE).

#### Subsampling

The classification and regression performances reported in Tables 5 and 6, respectively, are for models trained on the full training dataset ($N=11\text{,}884$). However, for the purpose of investigating performance as a function of dataset size, we have sub-sampled the training dataset to create a series of smaller datasets. These subsets were generated by drawing random samples from the training dataset while the holdout test dataset remains unchanged.

**Table 5:** Performance of the classification methods in this work. The accuracy is reported for the binary, sequential binary, and multiclass classification models. For the sequential binary and multiclass classifiers, the labels are analogous to the number of post-impact remnants (0,1,2). For the binary classifiers, the labels correspond to whether the LR/SLR exists (1) or not (0)

Type	Classes	Method	Accuracy
Binary (LR)	0 | 1,2	MLP	0.9879
		XGB	0.9882
Binary (SLR)	0,1 | 2	MLP	0.9680
		XGB	0.9731
Sequential Binary	0 | 1 | 2	MLP	0.9563
		XGB	0.9616
Multiclass	0 | 1 | 2	MLP	0.9532
		XGB	0.9627

**Table 6:** Coefficients of determination ($\mathrm{r^{2}}$-scores) for the analytic, semi-analytic, and data-driven methods investigated in this work. The data-driven models were trained on the train dataset and all models were evaluated on the holdout test dataset. The $\mathrm{r^{2}}$-scores quantify the correlation between the predicted and “true” values of the post-impact parameters, where the true values are obtained from SPH simulations. Entries listed as $n/a$ indicate the method was not designed to make a prediction for the parameter in question. Mass and angular momentum properties reflect the performance of the classification step, whereas the other properties quantify only the regression performance (see: Sect. 3.6.3). The (semi-)analytic methods use the classification scheme inherent to those methods, while the data-driven methods use a multiclass XGB classifier

Parameter	(Semi-)analytic			Data-driven			
	PIM	IEM	EDACM	PCE	GP	XGB	MLP
*ξ*	−1.1196	0.7518	0.6421	0.9733	0.9355	0.9793	0.9896
$M_{\mathrm{LR}}$	−0.0392	0.7698	0.6932	0.9741	0.9571	0.9829	0.9863
$M^{\mathrm{norm}}_{\mathrm{LR}}$	−1.7384	0.3950	0.2436	0.9415	0.9031	0.9747	0.9803
$F^{\mathrm{core}}_{\mathrm{LR}}$	0.5549	*n/a*	−0.0792	0.9564	0.9450	0.9516	0.9568
$J_{\mathrm{LR}}$	−144.4870	*n/a*	*n/a*	0.8162	0.7857	0.9121	0.9045
$\Omega _{\mathrm{LR}}$	−347.4151	*n/a*	*n/a*	0.8831	0.8702	0.9229	0.9133
$\theta _{\mathrm{LR}}$	−0.9391	*n/a*	*n/a*	0.8589	0.8278	0.8852	0.8764
$F^{\mathrm{melt}}_{\mathrm{LR}}$	*n/a*	*n/a*	*n/a*	0.9084	0.9647	0.9762	0.9798
$\delta ^{\mathrm{mix}}_{\mathrm{LR}}$	−1.2559	*n/a*	*n/a*	0.9251	0.8942	0.9710	0.9747
$M_{\mathrm{SLR}}$	−1.7159	−1.7159	0.0773	0.9601	0.8257	0.9442	0.9418
$M^{\mathrm{norm}}_{\mathrm{SLR}}$	−4.2472	−4.2472	−1.3057	0.9409	0.7052	0.9317	0.9028
$F^{\mathrm{core}}_{\mathrm{SLR}}$	*n/a*	*n/a*	*n/a*	0.9141	0.9265	0.9426	0.9332
$J_{\mathrm{SLR}}$	*n/a*	*n/a*	*n/a*	0.8893	0.8285	0.8819	0.8713
$\Omega _{\mathrm{SLR}}$	*n/a*	*n/a*	*n/a*	0.8803	0.9140	0.9044	0.9073
$\theta _{\mathrm{SLR}}$	*n/a*	*n/a*	*n/a*	0.8080	0.7933	0.8176	0.7969
$F^{\mathrm{melt}}_{\mathrm{SLR}}$	*n/a*	*n/a*	*n/a*	0.9272	0.9720	0.9693	0.9749
$\delta ^{\mathrm{mix}}_{\mathrm{SLR}}$	*n/a*	*n/a*	*n/a*	0.7864	0.7897	0.8171	0.7714
$M_{\mathrm{deb}}$	−0.5495	0.8635	0.7346	0.9672	0.9647	0.9867	0.9933
$M^{\mathrm{norm}}_{\mathrm{deb}}$	−0.8056	0.8448	0.7469	0.9848	0.9685	0.9895	0.9937
$F^{\mathrm{Fe}}_{\mathrm{deb}}$	*n/a*	*n/a*	*n/a*	0.9419	0.8811	0.9396	0.9538
$\delta ^{\mathrm{mix}}_{\mathrm{deb}}$	*n/a*	*n/a*	*n/a*	0.6436	0.5257	0.6747	0.6722
$\bar{\theta }_{\mathrm{deb}}$	*n/a*	*n/a*	−0.0227	0.3903	0.3364	0.4834	0.4653
$\theta ^{\mathrm{stdev}}_{\mathrm{deb}}$	*n/a*	*n/a*	−12.0333	0.8680	0.8634	0.9095	0.8812
$\bar{\phi }_{\mathrm{deb}}$	*n/a*	*n/a*	−19.7818	0.8168	0.7969	0.8603	0.8299
$\phi ^{\mathrm{stdev}}_{\mathrm{deb}}$	*n/a*	*n/a*	−0.7637	0.7657	0.7475	0.8149	0.7787

We created training subsets with set sizes increasing in steps of 100 up to 1000 and from thereon in steps of 1000 up to 11,000. Note that there is a difference between the training set size (TSS) and the *effective* TSS on which the regression models are actually trained. Because we remove undefined values in the pre-processing step, the effective TSS is dependent on the post-impact property in question. The effective TSS is therefore generally lower than the TSS for LR quantities and even lower for SLR quantities. To reiterate, this is because the number of remnants depends on the initial conditions of the collision. Outcomes with an LR are more common than outcomes with both an LR and SLR. This also affects the holdout test dataset. This is important because the effective test set size ($N_{\mathrm{test}}$) determines the expected variance *σ* of the performance measures,

### Hyperparameter optimization (HPO)

Once the data has been pre-processed, we perform HPO in order to identify the optimal set of hyperparameters for each data-driven model. The HPO procedure for PCE—which is implemented with MATLAB/UQLab—is different from that of the methods implemented in Python (i.e., GP, MLP, and XGB). In the case of the latter methods, we used the hyperopt library to identify the optimal hyperparameters for each model and post-impact parameter pair. The hyperopt package is a Python library designed to optimize hyperparameters over awkward search spaces with real-valued, discrete, and conditional dimensions, which makes it ideal for iterating machine learning hyperparameters. We employed hyperopt’s Bayesian sequential model-based optimization (SMBO) with a Tree-structured Parzen Estimator (TPE), which we found converged on optimal architectures more quickly than purely random or grid-based strategies.

The Python-based HPO procedure identifies an optimal architecture over 100 iterations. Each step in the HPO procedure employs a 5-fold cross-validation on the training dataset, using 80% of the training dataset for training and the remaining 20% as a validation set. At no point during HPO do the models see the holdout test dataset. For classification tasks, the negative average accuracy score (Sect. 3.6.1) across all five folds was used as the objective loss function during HPO. For regression tasks, the negative average $r^{2}$-score (see Sect. 3.6.2) across all five folds was used as the objective loss function.

The PCEs considered in this work have two distinct groups of hyperparameters. The HPO procedure for PCE searches over only one of these groups. The first group contains the maximal polynomial order, *p*, of the PCE and *q*-norm. A grid of these parameters is searched for the best configuration using a greedy algorithm (in that the optimal values for *p* and *q*-norm are only approximated). The second group of parameters consists of the maximum interaction, *r*, and the feature importance threshold. These parameters were optimized by trial and error. It is common to set *r* to very low values (∼2–3) following the *sparsity-of-effects* principle (Marelli and Sudret 2017). Here, we use a larger value of $r=4$, which results in more expensive training of the PCEs. We found that this value leads to the best performance, whereas higher values of *r* render the training even more expensive and does not substantially increase the performance (and in some cases leads to worse performance). The feature importance threshold was not varied, but rather set to 1% as it has been noticed that this is a conservative cut that still reduces the computation cost of PCE noticeably.

Each of the four data-driven methods requires a unique set of hyperparameters. The hyperparameter spaces searched for each emulation method are summarized in Table 4.

Because we do not enforce sparsity in the GPs used in this work, they require prohibitively long training times as dataset sizes increase. Therefore, for the GP models, we only carry out HPO up to training set sizes of $N=1000$. Beyond this training set size, we do not attempt HPO for GP models, but instead recycle the optimal hyperparameters identified for the GP models at $N=1000$ for each post-impact property.

### Performance evaluation

Once an optimal architecture was identified by the HPO procedure, the optimal architecture was re-trained on 100% of the training dataset. The resulting model was then evaluated on the holdout test dataset. Evaluating the performance of either a classication or regression model requires a carefully chosen metric appropriate to the problem.

#### Classification

In order to evaluate the performance of our classification models, we consider two metrics. The first, the accuracy score, is simply the fraction of correct predictions over the total number of predictions,

7 $$ \mathrm{acc} = \frac{\mathrm{TP} + \mathrm{TN}}{\mathrm{TP} + \mathrm{TN} + \mathrm{FP} + \mathrm{FN}} , $$

where predictions are either true positives (TP), true negatives (TN), false positives (FP), or false negative (FN). As we are not more concerned by either false positives (FP) or false negatives (FN), this metric is well suited evaluating our classification models.

However, while the accuracy quantifies the rate of correct predictions, it does not give any information as to the nature of the incorrect predictions. We therefore also consider the distribution of mass residuals resulting from the incorrect predictions (FP and FN predictions). Given two classification models with identical accuracy, the model with the lower mean and standard deviation in its residual distribution is preferred.

#### Regression

There are several commonly employed regression metrics that are not suitable for collision emulation due to the range of the post-impact properties. For example, mean squared error (MSE) is not scale invariant and relative error metrics are ill-suited to the many parameters that can take on null values. For this reason, we use the coefficient of determination, known as the $r^{2}$-score, to measure the quality of the regressors,

8 $$ r^{2} = 1 - \frac{\mathrm{SS}_{\mathrm{res}}}{\mathrm{SS}_{\mathrm{tot}}}, $$

where $\mathrm{SS}_{\mathrm{res}} = \sum_{i} (y_{i} - \hat{y}_{i})^{2}$ is the residual sum of squares and $\mathrm{SS}_{\mathrm{tot}} = \sum_{i} (y_{i} - \bar{y})^{2}$ is the total sum of squares. Here, $y_{i}$ is the *i*th expected value, *ȳ* is the mean of the expected distribution, and $\hat{y}_{i}$ is the *i*th predicted value. The $r^{2}$-score has been used as the performance metric in similar work (Cambioni et al. 2019) and is therefore a prudent choice in order to make comparisons to other studies.

In addition to the $r^{2}$-score, which quantifies the regression performance globally, we also consider the residuals as a function of each individual pre-impact property. Because we consider 12 pre-impact properties, 27 post-impact properties, and four data-driven models, the number of residual plots is in excess of a thousand. We therefore provide the residuals for a single post-impact property (accretion efficiency) at the end of the paper (Figs. 8–11) and provide the remaining residual plots as Additional file 1.

#### Linking classification and regression

Ideally, in order to evaluate the performance of our emulation method, we would evaluate the performance of the classification and regression models together, as a unified emulation pipeline. However, for many of the post-impact properties, false positive (FP) and false negative (FN) predictions in the classification stage result in meaningless regression predictions which cannot be evaluated by the regression metric. When evaluating the regression models, we must therefore be careful to distinguish which models reflect the classification performance in their $r^{2}$-scores and which do not.

##### Mass and angular momentum properties

In the case of either a FP or FN prediction by the classifier, these properties have physically meaningful values; these properties take on null values when they don’t exist and can therefore be incorporated into the regression performance metric. Thus, the $r^{2}$-scores for these properties reflect the performance of both the classification and regression models used in the emulation pipeline.

##### Other properties

For these properties, in the case of a FP prediction by the classifier, there is no meaningful value with which to compare the subsequent regression prediction. In the case of a FN prediction, there is no default value of the property to use as the “predicted” value. Indeed, the values of these properties do not trend toward any particular value as the mass of the associated remnant approaches zero. It is therefore not possible to incorporate the misclassified collisions into the $r^{2}$-scores of these properties. As a result, the $r^{2}$-scores for these properties reflects only the performance of the regression model used in the pipeline.

The analytic and semi-analytic emulation methods include their own classification schemes, which are used in evaluating their regression performance. The data-driven emulation methods use a multiclass XGB classifier (see Sect. 3.4.1) during the classification stage.

### Feature importance

The data-driven techniques that we consider in this work allow us to evaluate and compare feature importance for each post-impact property. Importance metrics are powerful methods for quantifying relationships between pre- and post-impact parameters. In this work, we report Sobol’ indices derived from PCE and SHAP values derived from XGB models. We consider feature importance metrics from these distinct methods in order to compare how fundamentally different techniques make their predictions. If both methods leverage the same pre-impact properties to predict a given post-impact properties, then this would strongly indicate an underlying physical relationship between the pre- and post-impact properties.

#### Sobol’ indices

Sobol’ indices (Sobol’ 1993; Le Gratiet et al. 2016) measure how sensitive a given post-impact parameter is to each of the individual pre-impact parameters, as well as to any of their interactions. The indices quantify the relative contribution of variance explained by one variable—or group of variables—to the total variance,

9 $$ S_{i_{1}\dots i_{s}} = \frac{\sigma ^{2}_{i_{1}\dots i_{s}}}{\sigma ^{2}} , $$

where $S_{i_{1}\dots i_{s}}$ is the Sobol’ index of order *s*. The first order Sobol’ indices are the values $S_{i}$ which characterize the variance explained by the variable $x_{i}$. The higher order Sobol’ indices (second order $S_{ij}$ with $i\neq j$ etc.) quantify how much variance is explained not by single variables but rather by their interactions.

The Sobol’ indices are a particularly useful sensitivity measurement tool in the context of PCE because a Sobol’ decomposition can be computed directly from a PCE by employing a simple reordering of terms. Hence the computation of Sobol’ indices from a PCE is analytic and exact. For a more thorough introduction to Sobol’ sensitivity analysis we refer to the following references (Marelli et al. 2017; Le Gratiet et al. 2016).

#### SHAP (SHapley Additive exPlanation) values

To understand how our models are making certain predictions we use the SHAP framework proposed in Lundberg and Lee (2017). This is based on Shapley values (Roth 1988), introduced in a game theory context as a solution to fairly distributing gains and costs of a given game *v* to a set of collaborating players *N*. The Shapley value *ϕ* of one player *i* is the average expected marginal contribution of player *i* after all possible combinations of other players (denoted as *S*) have been considered:

$$ \phi _{i} (v) = \frac{1}{n} \sum _{S \subseteq N\setminus \{i\}} \binom{n-1 }{ \vert S \vert }^{-1} \bigl(v\bigl(S \cup \{i\}\bigr) - v(S) \bigr). $$

Analogously, in the context of model interpretability, the *game* *v* is how well the model output is represented (for a fixed input *x*) and the set of players *N* are the features. In Lundberg and Lee (2017), the game $v(S)$ is defined as the conditional expectation $E(f(x)|x_{S})$, for model *f*, observation *x* and $x_{S}$, the observation in which the features coincide with observation *x* on the set of features *S*. To avoid the necessity to train many models that include or exclude features to evaluate $v(S)$, specific model based approximations can be used. In our work, SHAP values are computed from the gradient boosting models as described in Lundberg et al. (2018).

## Results

The following sections describe the performance of the classification and regression models, dependence on the training set size (TSS), and the results of the feature importance analyses. We first discuss the performance of the classification strategies and models. We then discuss the performance of the single-target regression models for the post-impact properties considered in this work and their dependence on TSS. Finally, we report the feature importance results.

### Classification performance

We considered two distinct classification strategies. In the first strategy, we trained one multitarget classifier to directly classify the number of post-impact remnants (0, 1, or 2). In the second strategy, we trained two binary classifiers to separately classify the existence of the LR and SLR. These binary classifiers were then used in sequence to classify, first, if a single remnant (the LR) exists and, if so, does a second remnant (the SLR) exist? This second strategy, which we refer to as sequential binary classification, produces the same class labels (0, 1, or 2) as the multitarget classifier and can therefore be compared directly. For each strategy, we tested both MLP and XGB models.

**Figure 4:**
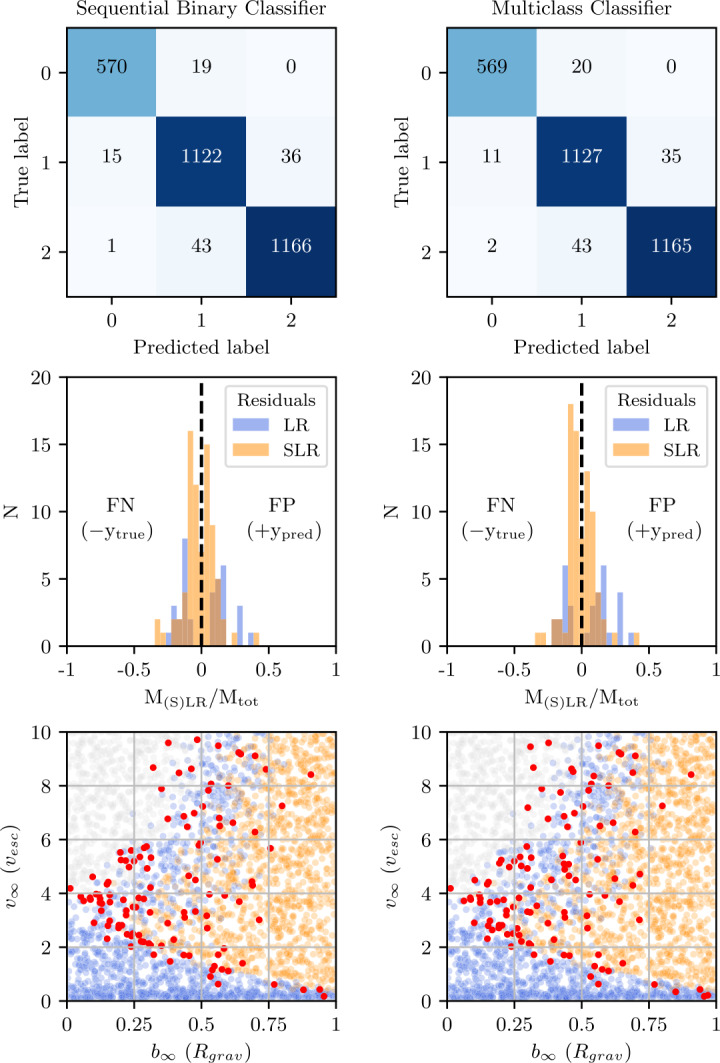
*Comparison of classification strategies*. Performance metrics for two distinct classification strategies: binary sequential (left column) and multiclass (right column). In the top panels, the confusion matrices for each strategy are shown; each collision is plotted as its predicted label (rows) and true label (columns). Predictions along the diagonal are correct classifications, whereas those in off-diagonal cells are misclassifications. In the middle panels, the distribution of masses resulting from false negatives (FN) and false positives (FP) are plotted. These mass residuals are important to constrain, because FP and FN predictions cannot be quantified in the regression stage. In the bottom panels, the classifier predictions are plotted along the $b_{\infty }$–$v_{\infty }$ hyperplane, where gray indicates class 0 (no remnants), blue is class 1 (one remnant), orange is class 2 (two remnants), and misclassified collisions are indicated by red markers. The misclassified collisions are clustered near the transitions between classes

In order to evaluate and compare the classification strategies, we considered the prediction accuracy, as well as the distribution of mass residuals resulting from false negative (FN) and false positive (FP) predictions. The accuracy of the data-driven classification models is reported in Table 5. As is evident from these results, the accuracy of both classification strategies, regardless of the underlying model (i.e., MLP or XGB), is practically identical. Confusion matrices for the two strategies are provided in the top panels of Fig. 4.

The mass residuals resulting from FP and FN predictions, as evaluated on the holdout test dataset, are shown in the middle panels of Fig. 4. The mass residuals are computed as follows: For FP predictions, the predicted value, $y_{\mathrm{pred}}$, has been predicted by the associated XGB regression model. For FN predictions, the residual is given by the true value, $y_{\mathrm{true}}$. The distribution of residuals produced by the classifier is an important consideration, especially in light of the indistinguishable accuracy scores. Over the course of an N-body simulation, we would prefer that the residuals do not show a significant bias (i.e., the distribution mean should be as close to zero as possible) and the standard deviation should be minimized. The means and standard deviations of the mass residual distributions resulting from the sequential binary classifier are $\mu _{\mathrm{LR}} = 0.0215$ and $\sigma _{\mathrm{LR}} = 0.1679$ for the LR and $\mu _{\mathrm{SLR}} = -0.0146$ and $\sigma _{\mathrm{SLR}} = 0.1139$ for the SLR. For the multiclass classifier, the means and standard deviations are $\mu _{\mathrm{LR}} = 0.0521$ and $\sigma _{\mathrm{LR}} = 0.1640$ and $\mu _{\mathrm{SLR}} = -0.0119$ and $\sigma _{\mathrm{SLR}} = 0.1068$.

In the bottom panels of Fig. 4, the distribution of misclassified collisions is shown along the $b_{\infty }-v_{\infty }$ hyperplane (roughly corresponding to the collision geometry). This illustrates, unsurprisingly, that the misclassfied collisions are concentrated near the transitions between classes. The misclassified points are clustered tightly around the transition from merging to hit-and-run type outcomes, which is expected because this transition is a sharp transition in the parameter space. The other misclassified points are largely concentrated in the regime that represents the transition from no remnants, to one remnant, and then to two remnants in the hit-and-run regime.

Just as with the classification accuracy and the mass residual means and standard deviations, the distributions of misclassfied collisions are practically identical. Thus, there is no discernible difference between the performance of the classification strategies, nor between the MLP and XGB models. We suggest that the multiclass classification strategy is therefore to be preferred on account of its simpler implementation and reduced computational overhead.

### Regression performance

We now discuss the performance of the regression models with respect to the subset of post-impact properties investigated in this work (Table 3). The performances of the regression models on each post-impact property are quantified by $r^{2}$-scores (see Sect. 3.6.2) and are tabulated in Table 6. Given the large number of post-impact properties, we first describe a few general results that are apparent from the regression performances:

- For all post-impact properties, the data-driven models outperform the analytic and semi-analytic methods. Only in the case of the debris mass, $M_{\mathrm{deb}}$, do the semi-analytic methods approach the accuracy of the data-driven methods.
- The MLP and XGB models consistently perform best and, to within the expected variance, achieve equivalent accuracy for most post-impact properties.
- The PCE models tend to achieve $r^{2}$-scores slightly below those of the MLP and XGB models, with the notable exception being the case of the SLR (normalized) mass, where PCE outperforms the other methods.
- For most post-impact properties, the GP models perform significantly worse than the other data-driven methods. However, they still perform significantly better than the analytic or semi-analytic methods.
- Despite having the largest effective training set size ($N=11\text{,}884$), some debris properties proved difficult to regress, including the mixing ratio and the spatial distribution properties.

#### Analytic & semi-analytic methods

The analytic and semi-analytic methods investigated in this work achieved relatively poor $r^{2}$-scores relative to the data-driven methods. While limited to a narrow set of parameters, IEM is the most accurate of these methods for LR properties, where EDACM performs significantly worse. PIM performs worst, with the notable exception that it excels at predicting the core mass fraction of the LR.

The analytic and semi-analytic methods’ regression performances on $M_{\mathrm{LR}}$ are significantly below that of the data-driven methods, achieving $r^{2}$-scores of 0.7698 and 0.6932 for IEM and EDACM, respectively. Their relative performance is somewhat surprising, as EDACM uses an explicit relationship to predict $M_{\mathrm{LR}}$, whereas IEM only predicts $M_{\mathrm{deb}}$ and provides no explicit relation for $M_{\mathrm{LR}}$. PIM does poorly when predicting $M_{\mathrm{LR}}$. This latter result is perhaps not surprising, as PIM assumes all collisions result in perfect accretion and studies have shown that this is not the case in most collisions (Quintana et al. 2016).

Of the analytic and semi-analytic methods, only EDACM is capable of making explicit (non-zero) prediction for $M_{\mathrm{SLR}}$ ($M^{\mathrm{norm}}_{\mathrm{SLR}}$). The resulting $r^{2}$-score, 0.0773 (−1.3057), is much worse than the associated score for its prediction of $M_{\mathrm{LR}}$ ($M^{\mathrm{norm}}_{\mathrm{LR}}$). EDACM’s significantly worse performance when predicting the mass of the SLR as opposed to the LR is likely influenced by two important aspects of the EDACM algorithm. First, EDACM delineates collisions into multiple regimes (e.g., perfect merging, hit-and-run), in which different analytic relations are used. Second, the calculation of $M_{\mathrm{SLR}}$ uses $M_{\mathrm{LR}}$ as an input (via $M^{\mathrm{norm}}_{\mathrm{LR}}$; see Eq. (25) in Appendix C). Thus, any error in the prediction of $M_{\mathrm{LR}}$ will propagate to the prediction of $M_{\mathrm{SLR}}$. Note that the data-driven models do not suffer from this issue, as the prediction of the post-impact properties are entirely decoupled from each other.

In the case of the debris properties, only IEM explicitly predicts the mass. IEM predicts $M^{\mathrm{norm}}_{\mathrm{deb}}$, from which $M^{\mathrm{norm}}_{\mathrm{LR}}$ is subsequently derived. IEM’s prediction of $M^{\mathrm{norm}}_{\mathrm{deb}}$ is surprisingly good with an $r^{2}$-score of 0.8448, but still approximately 10% lower than that of the data-driven methods. This reverse approach taken by IEM, first predicting the $M^{\mathrm{norm}}_{\mathrm{deb}}$, allows it to make an accurate, if implicit, prediction of $M_{\mathrm{LR}}$, relative to the other analytic and semi-analytic methods.

We additionally compared the ability of the analytic and semi-analytic methods to predict the normalized mass quantities. In the case of the LR, this resulted in significantly worse performance for these methods. Similarly for IEM and EDACM, the $r^{2}$-scores are significantly lower when predicting the normalized masses of the LR and SLR, but are similar for the debris mass. The poor performance of the analytic and semi-analytic methods on the normalized quantities is expected as a side-effect of how the $r^{2}$-score is calculated. Because the normalized quantities are scaled by the total mass of the collision ($M_{\mathrm{tot}}$, which is different for each collision), the distribution of $M_{\mathrm{tot}}$ skews the predicted distribution of $M_{\mathrm{LR}}$. Thus, the normalized quantities are only of interest to the data-driven methods, which predict the normalized masses directly and therefore don’t suffer from this issue.

The core mass fraction of the LR ($F^{\mathrm{core}}_{\mathrm{LR}}$) is predicted by both PIM and EDACM (via a mantle stripping formula (Marcus et al. 2010)). Here, PIM performs unexpectedly well, yielding an $r^{2}$-score of 0.5549. PIM’s unexpected performance on $F^{\mathrm{core}}_{\mathrm{LR}}$ provides physical insight into the processes that determine $F^{\mathrm{core}}_{\mathrm{LR}}$, suggesting that the cores of pre-impact bodies often merge. In contrast, EDACM yields an objectively poor $r^{2}$-score of −0.0792 for $F^{\mathrm{core}}_{\mathrm{LR}}$, despite utilizing a more complicated formulation.

**Figure 5:**
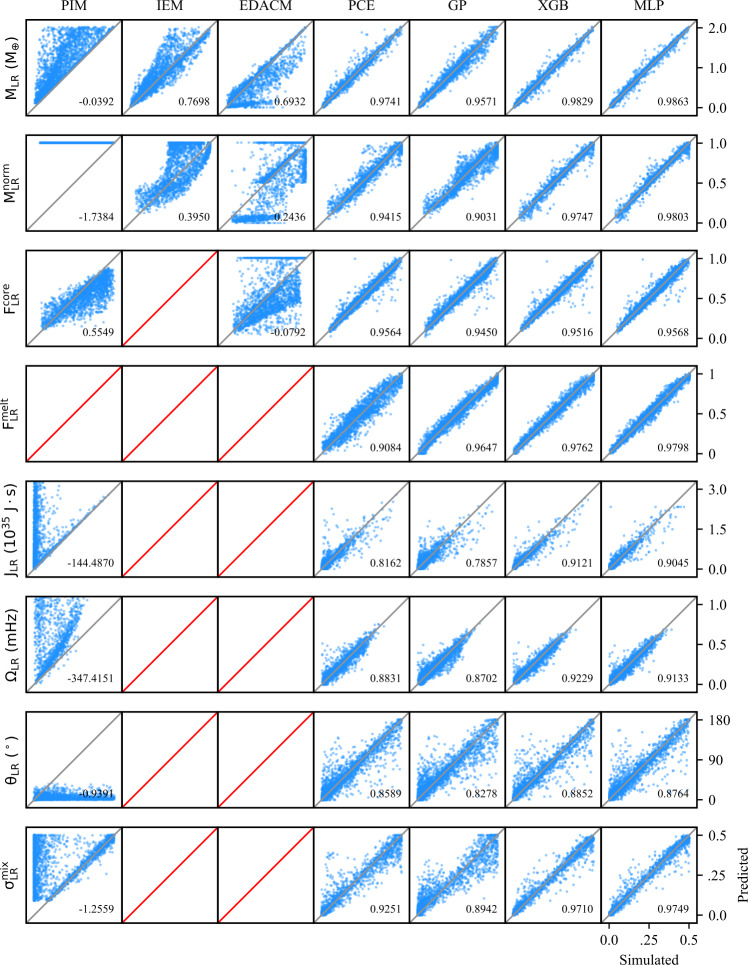
*Simulated versus predicted values for LR properties*. Simulated versus predicted values for post-impact parameters related to the largest remnant. The blue points represent individual predictions by the model, assuming perfect pre-classification of the existence or non-existence of the remnant. The grey lines, stretching from the lower left to the upper right, indicate a 1:1 correlation. For a perfect model all blue points would lie on this line. Cells with no points and a red line indicate that the model is not able to make predictions for the post-impact property in question

For both $F^{\mathrm{core}}_{\mathrm{LR}}$ and $M_{\mathrm{SLR}}$, a large factor in EDACM’s poor performance are the collisions that comprise the super-catastrophic disruption (SCD) regime (Leinhardt and Stewart 2012) (see Appendix C). In Fig. 5, it’s clear that $M_{\mathrm{LR}}$ is systematically under-predicted for a subset of collisions, which corresponds to the SCD regime. The poor predictions in this subset of collisions are propagated to the calculations of both $F^{\mathrm{core}}_{\mathrm{LR}}$ and $M_{\mathrm{SLR}}$, causing the former to be systematically over-predicted and the latter to be under-predicted.

In addition to the data-driven methods, only PIM makes any prediction of rotational properties. These predictions were not expected to be very accurate, given the assumptions of the model (see Appendix B). Indeed, the resulting regression performances are exceptionally poor. As Fig. 13 illustrates, PIM tends to greatly overestimate the angular momentum budget of the LR ($J_{\mathrm{LR}}$), which results in similar overestimates of its rotation rate ($\Omega _{\mathrm{LR}}$). This has the opposite effect on $\theta _{\mathrm{LR}}$, which is systematically underpredicted by PIM. The obliquities are predicted to be low because the angular momentum delivered by the impact tends to dominate the resulting angular momentum budget.

The method for handling debris in the N-body implementation of EDACM (Chambers 2013) performs poorly relative to the data-driven methods as well. This is unsurprising given the simplifying assumptions of the debris model (see Appendix C). This would suggest that more accurate models for handling debris within N-body simulations are sorely needed.

#### Data-driven methods

The data-driven methods universally evince better accuracy than the analytic and semi-analytic methods. Of the data-driven methods, the MLP and XGB models generally achieve the best performance, but are often matched by the PCE models. The GP models, on the other hand, generally perform significantly worse than the other data-driven methods.

**Figure 6:**
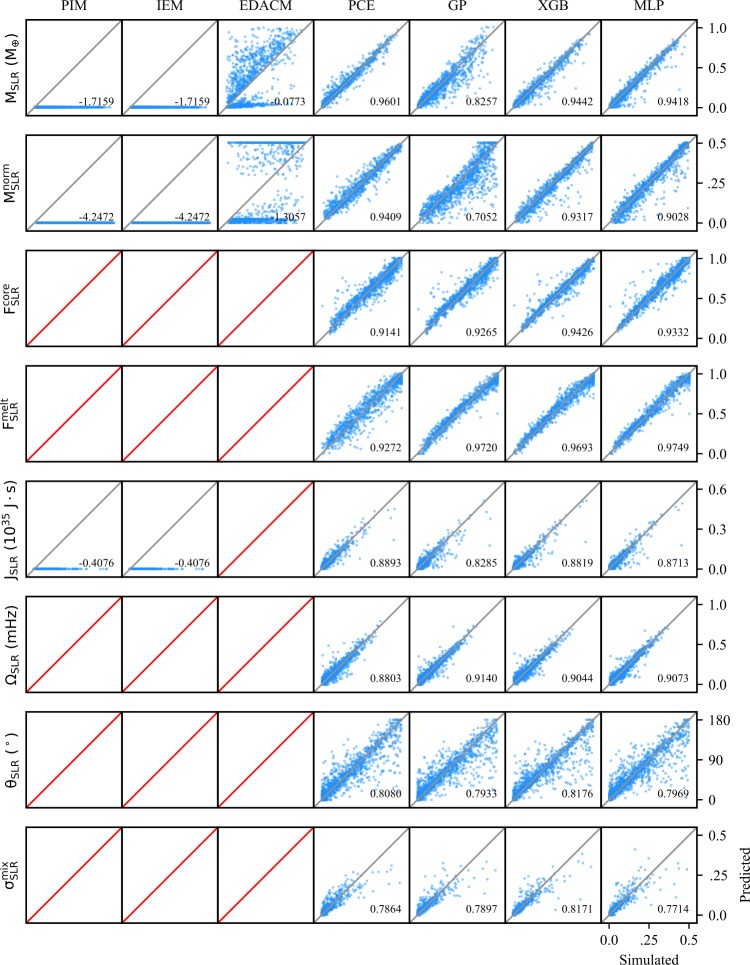
*Simulated versus predicted values for SLR properties*. Simulated versus predicted values for post-impact parameters related to the second largest remnant. The blue points represent individual predictions by the model, assuming perfect pre-classification of the existence or non-existence of the remnant. The grey lines, stretching from the lower left to the upper right, indicate a 1:1 correlation. For a perfect model all blue points would lie on this line. Cells with no points and a red line indicate that the model is not able to make predictions for the post-impact property in question

**Figure 7:**
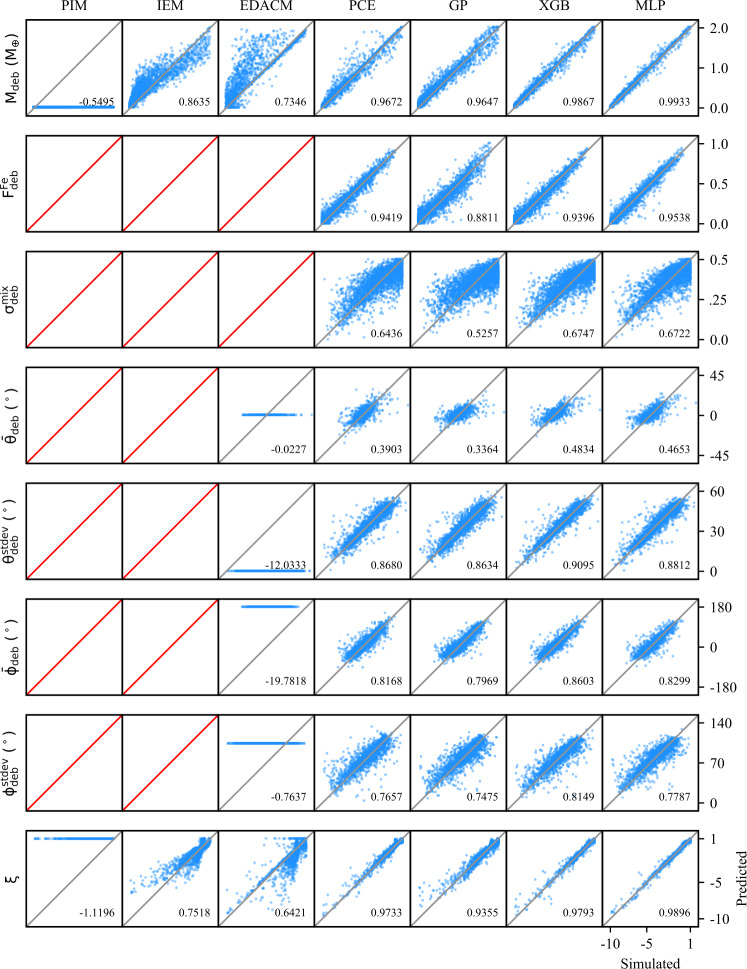
*Simulated versus predicted values for debris properties*. Simulated versus predicted values for post-impact parameters related to the second largest remnant. The blue points represent individual predictions by the model, assuming perfect pre-classification of the existence or non-existence of the remnant. The grey lines, stretching from the lower left to the upper right, indicate a 1:1 correlation. For a perfect model all blue points would lie on this line. Cells with no points and a red line indicate that the model is not able to make predictions for the post-impact property in question

The data-driven predictions for each post-impact property are plotted relative to their true (i.e., simulated) values in Fig. 5 for LR properties, Fig. 6 for SLR properties, and Fig. 7 for debris properties (and the accretion efficiency).

**Figure 8:**
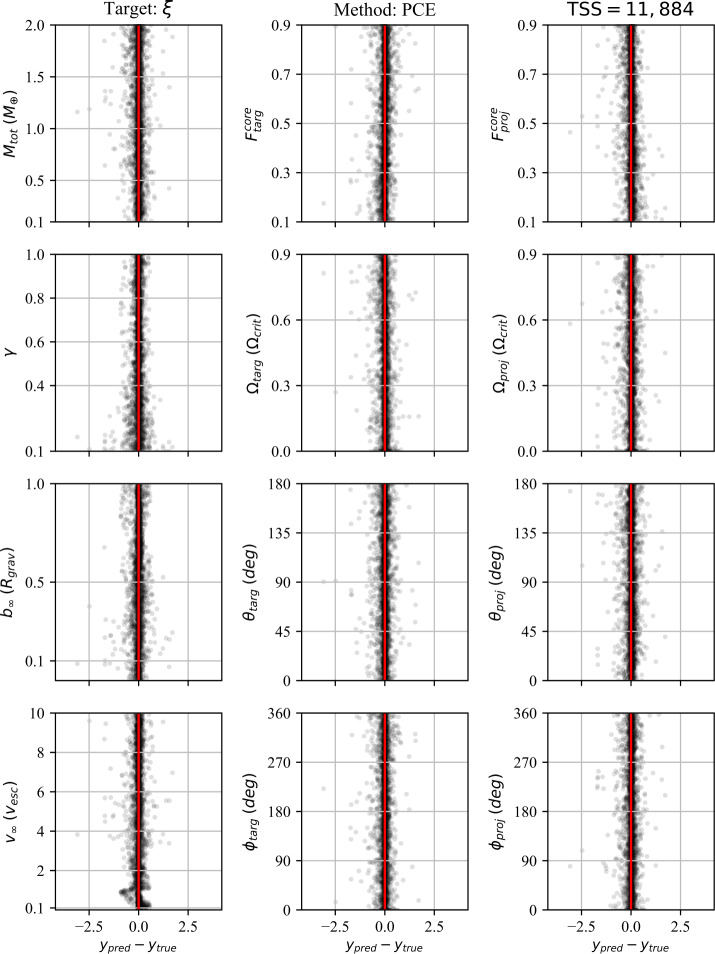
*Accretion efficiency residuals of the PCE model*. The $r^{2}$-score alone is insufficient to assess the performance of a regressor. The distribution of residuals for each post-impact property is an important consideration. Residuals for all post-impact properties and models are available in the material

**Figure 9:**
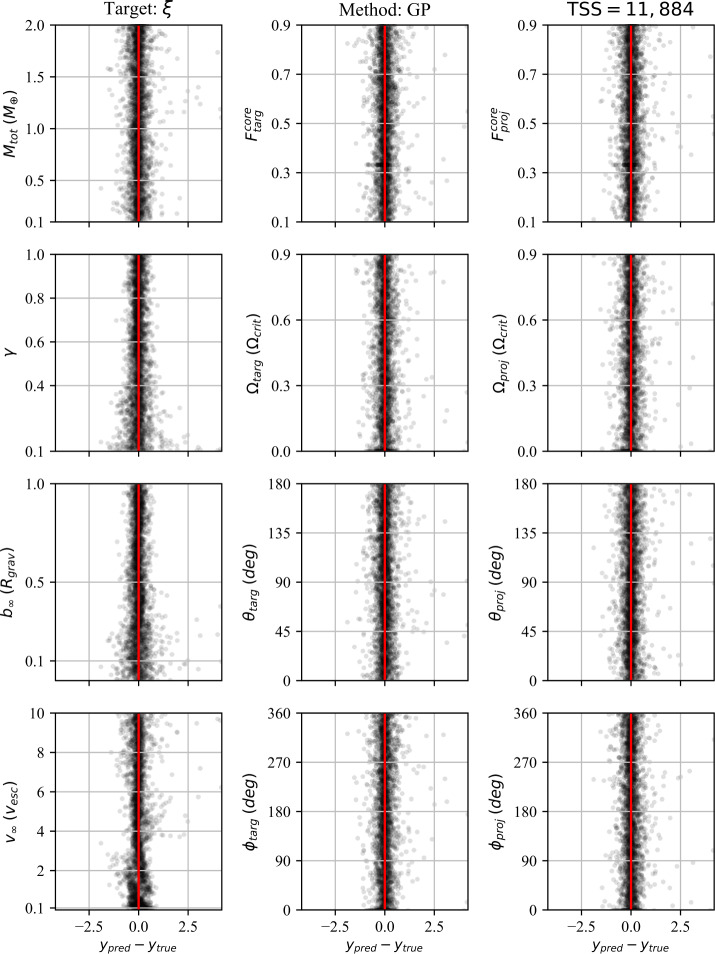
*Accretion efficiency residuals of the GP model*. The $r^{2}$-score alone is insufficient to assess the performance of a regressor. The distribution of residuals for each post-impact property is an important consideration. Residuals for all post-impact properties and models are available in Additional file 1

**Figure 10:**
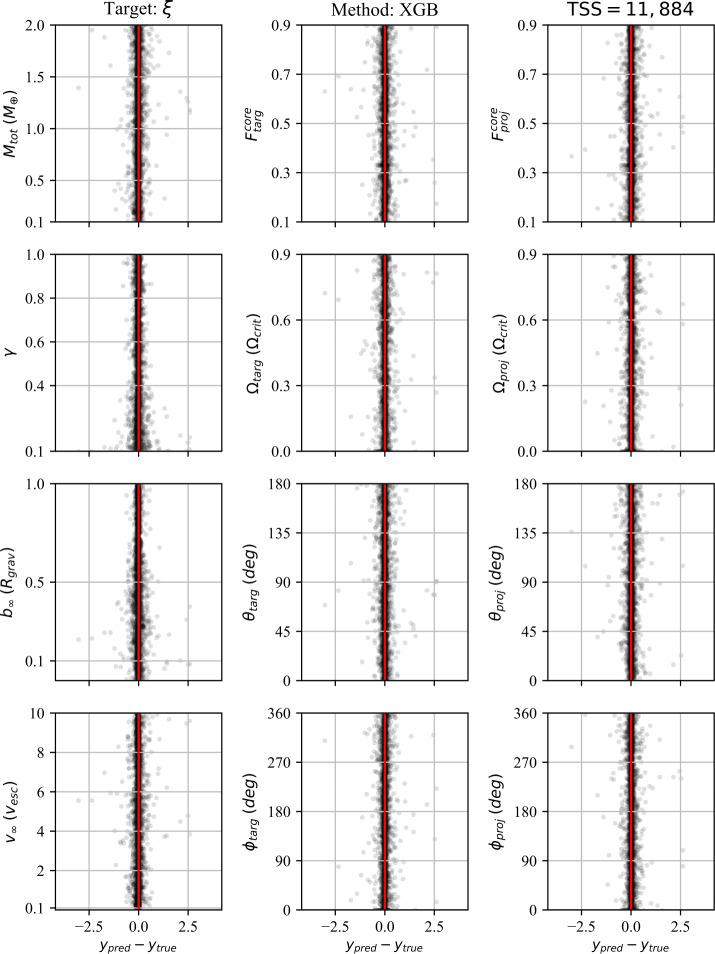
*Accretion efficiency residuals of the XGB model*. The $r^{2}$-score alone is insufficient to assess the performance of a regressor. The distribution of residuals for each post-impact property is an important consideration. Residuals for all post-impact properties and models are available in Additional file 1

**Figure 11:**
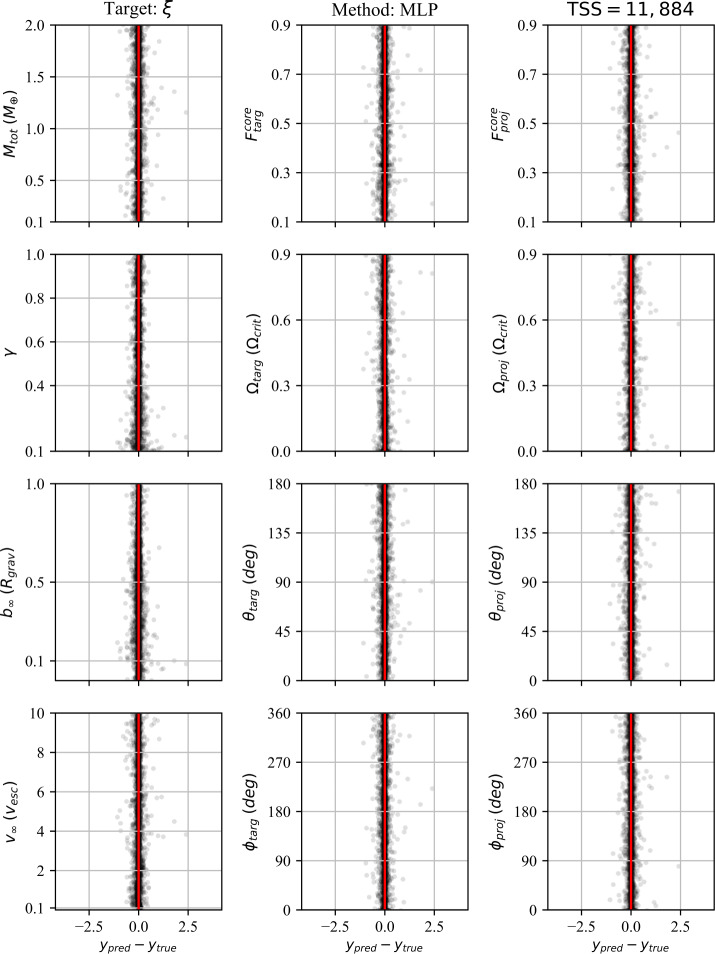
*Accretion efficiency residuals of the MLP model*. The $r^{2}$-score alone is insufficient to assess the performance of a regressor. The distribution of residuals for each post-impact property is an important consideration. Residuals for all post-impact properties and models are available in Additional file 1

In Figs. 8–11, we show the prediction residuals for accretion efficiency resulting from each of the four data-driven methods. The distribution of residuals is an important consideration in addition to the $r^{2}$-score, as it can reveal dependencies of the residuals on individual pre-impact properties. The most common residual dependence revealed by these plots (see Additional file 1) is that which corresponds to the boundary between the merging and hit-and-run regimes. This manifests as increased residual values at low velocities (${\approx}1~\mathrm{v}_{\mathrm{esc}}$). This dependence tends to be particular pronounced for GP models, which are not able to capture the relative sharp transition between these regimes.

Given the large number of plots required to illustrate the residuals, we show only those for a single post-impact property (accretion efficiency) here and provide the remaining residuals in Additional file 1.

For a given post-impact parameter, the MLP, PCE, and XGB models achieve similar performances. Indeed, the differences in performance are generally small and fall within the expected variance of the test dataset. This demonstrates that, despite fundamentally different underlying methodologies, data-driven methods are capable of achieving roughly the same performance given a sufficiently large dataset.

In many cases, the performance of the GP models is below that of the other data-driven models. The lower $r^{2}$-scores for GPs are likely, at least in part, a result of the limitations on HPO for GPs. Recall that HPO is only carried out for GP models on training datasets with sizes of $N \leq 1000$. Due to these limitations, the GP models are not fully optimized on the full training dataset, while the other data-driven methods are.

For different post-impact properties, the best achieved accuracy can differ significantly. Given that the different data-driven techniques are able to achieve the indistinguishable accuracy, this suggests that the difficultly in reaching higher accuracy lies not with the emulation methodology, but rather with the data or the underlying physical processes that determine the post-impact quantity. In the former case, this may be due to insufficient fidelity of the simulations, insufficient resolution of the training dataset, or ill-defined parameterizations of the post-impact properties.

A known source of uncertainty in the post-impact quantities is the post-impact group finding step. In subsequent steps, the group finding algorithm can assign particles to a group to which they were previously not a part of. While the number of these particles is almost always small (on the order of a few), this can have a large effect on the calculation of post-impact quantities, especially for remnants or debris fields composed of a small number of particles.

Parameters whose accuracy are likely affected by the underlying physical process are, for example, the obliquities. In this case, the limitation on performance may be a result of the obliquity (via the angular momentum vector) being highly variable at low rotation rates. Another set of parameters affected in this way are likely those related to the debris field spatial distribution (e.g., $\bar{\theta }_{\mathrm{deb}}$ and $\bar{\phi }_{\mathrm{deb}}$). It may be that these quantities are inherently noisy as a result of being sensitive to small changes in the impact geometry. Parameters such as these may benefit from being separated into distinct outcome regimes.

### Dependence on training set size

In the preceding sections we have discussed the performance of the regression models as trained on the full training dataset ($N=11\text{,}884$). Here we discuss their performance on smaller subsets of the the training data, in order to quantify regression performance relative to dataset size. All subsets are evaluated against the full holdout test set.

**Figure 12:**
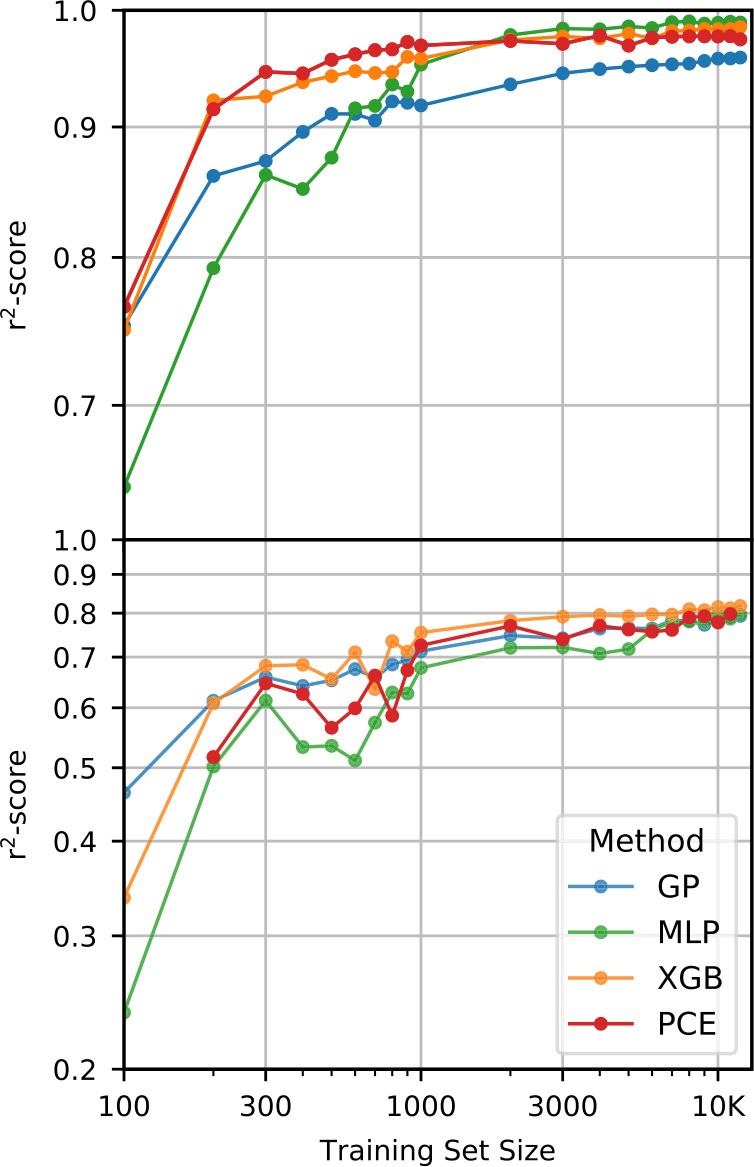
*Performance as a function of training set size*. Performance on the holdout test dataset (quantified by $r^{2}$-scores) is shown as a function of training set size (TSS). Regression performance for a well-performing parameter $M_{\mathrm{LR}}$ is shown in the top panel and a relatively difficult to regress parameter $\theta _{\mathrm{SLR}}$ the lower panel

The regression performances of the emulators see their most dramatic improvement on training dataset sizes of less than a thousand (Fig. 12). On dataset sizes above roughly a thousand, the $r^{2}$-scores continue to improve slowly until a few thousand, after which only marginal gains are achieved. For many post-impact properties, near-optimal performances are achieved quickly. However, some post-impact properties continue to see improvement with increasing training set sizes. This suggests that, while the masses and several other properties only require relatively small training datasets, other properties relevant to terrestrial planet formation will require datasets even larger than those considered here. This is especially true of properties related to the SLR, for which the effective TSS is generally about half that of the TSS for LR properties.

### Feature importance

Using Sobol’ indices (Sect. 3.7.1) derived from PCE and SHAP values (Sect. 3.7.2) derived XGB models, we quantify the importance of the pre-impact properties in determining each post-impact property. We consider these two distinct metrics in order to compare how the data-driven methods make their predictions. These feature importance metrics leverage our data-driven models to provide physical insight into a high-dimensional problem that would otherwise be difficult analyze.

#### Sobol’ analysis

**Figure 13:**
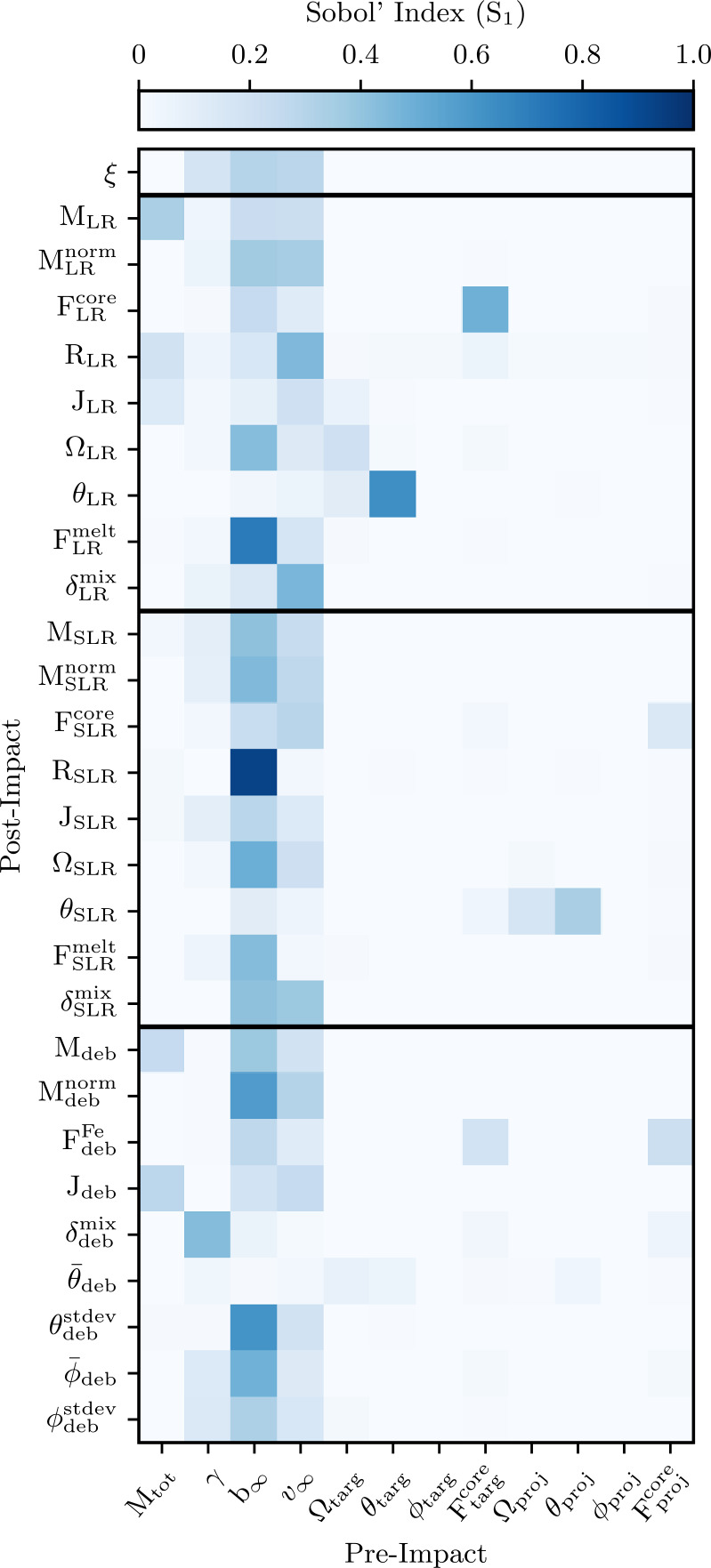
*Sobol’ indices*. The Sobol’ index is a sensitivity metric that quantifies the contribution of each pre-impact parameter in determining the value of a given post-impact quantity. For all post-impact properties, the Sobol’ analysis indicates that the geometry of the impact is important in determining the outcome. Additionally, for parameters related to the post-impact rotation, the pre-impact rotational states of the target and projectile are also important

The Sobol’ indices in Fig. 13 suggest that, for most post-impact properties, the geometry and energy of the impact—determined by *γ*, $b_{\infty }$, and $v_{\infty }$—are the strongest factors in deciding the outcome of a collision. However, for some post-impact properties, other pre-impact parameters are important. This is true for the obliquities and core mass fractions, which are generally dependent on the pre-impact values of the associated body—i.e., the target for the LR and projectile for the SLR. Pointedly, the Sobol’ analysis also shows that the azimuthal orientation (*ϕ*) of the pre-impact bodies tend to play an insignificant role in the outcome of collisions.

#### SHAP values

**Figure 14:**
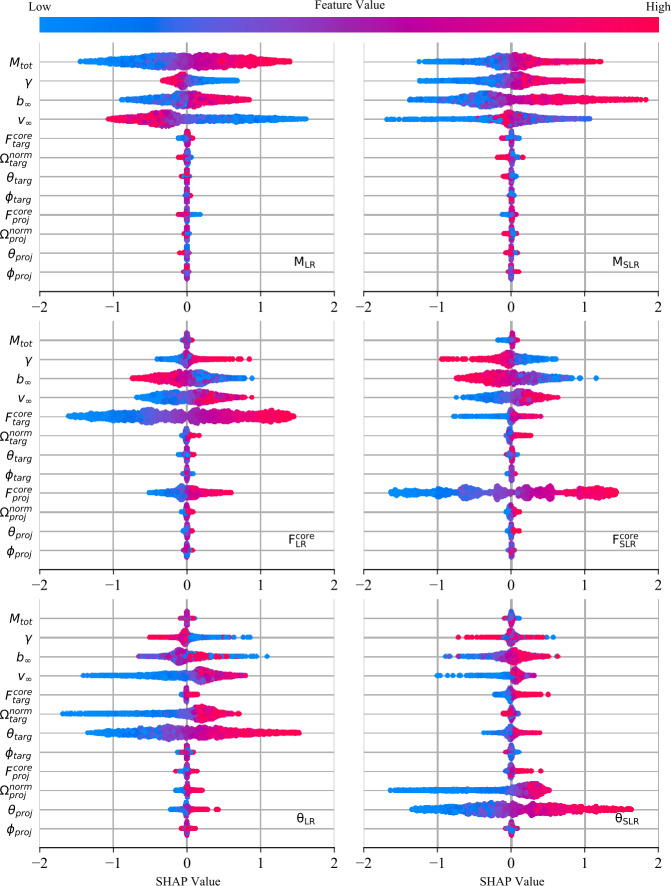
*SHAP values for a selected subset of post-impact parameteres*. The SHAP values are a useful metric for explaining how data-driven models classify or predict collision outcomes. On the *x*-axis, the SHAP value quantifies the magnitude of the contribution by each pre-impact quantity. Negative SHAP values push the value of the post-impact parameter lower, whereas positive SHAP values push the value higher. The normalized value of the pre-impact parameter (ordered along the *y*-axis) is indicated by color, with bluer values indicating lower pre-impact parameter values and higher values red

As opposed to the global view provided by the Sobol’ indices in the previous section, the SHAP values provide a local view of feature importance for each post-impact property. In Fig. 14, we show SHAP values on the test set for a selected subset of post-impact properties of the LR and SLR.

In predicting the masses of the remnants ($M_{\mathrm{LR}}$ and $M_{\mathrm{SLR}}$), the XGB models leverage the total ($M_{\mathrm{tot}}$) and impact geometry (*γ*, $b_{\infty }$, and $v_{\infty }$). The other pre-impact properties—those related to the internal and rotational properties of the target and projectile—appear to play little role in the predictions. The feature importance metrics are largely intuitive in this case, indicating that lower total masses ($M_{\mathrm{tot}}$) lead to lower predictions of the remnant masses. Similarly, head-on, high-velocity impacts drive the predictions toward lower remnant masses, which is expected in disruptive collisions.

In the case of the remnant core mass fractions, the SHAP values indicate that the most important pre-impact property is the core mass fraction of the associated pre-impact body (i.e., the target for the LR and projectile for the SLR). This is also somewhat intuitive and matches the results of the Sobol’ analysis.

The remnant obliquities ($\theta _{\mathrm{LR}}$ and $\theta _{\mathrm{SLR}}$) are relatively difficult properties to regress. These post-impact properties show a strong dependence on the pre-impact rotation rate (Ω) and obliquity (*θ*) of the associated body. Once again, the Sobol’ indices indicate the same feature importance. For the obliquity of the LR ($\theta _{\mathrm{LR}}$), the impact velocity ($v_{\infty }$) is also important.

## Discussion

We now discuss several aspects of collision emulation that are of interest in addition to accuracy. We begin by discussing the importance of the underlying training data. We then discuss the relationships between pre- and post-impact properties extracted from our data-driven models, the technical considerations that must be made when implementing such models, and finally we suggest directions for future work that might improve the methodology and models which we have developed here.

### Training data

The results which we have presented here show that functionally distinct data-driven methods can achieve equivalent prediction accuracy, suggesting that further gains in accuracy are limited by the underlying training data and not by the model algorithms. While we have demonstrated that training dataset sizes of at most a few thousand are sufficient to achieve high accuracy, there are still significant improvements to be made to those datasets. Indeed, simply increasing the training set size is not likely to significantly improve prediction accuracy, as Fig. 12 shows. Instead, improvements to the training dataset should be focused in those regions where the classification and regression models struggle.

In particular, Fig. 4 clearly shows that the classification models perform poorly at the transitions between collision regimes (e.g., from merging to hit-and-run). This poor classification performance is mirrored by increased regression residuals around these transitions for many post-impact properties. This strongly suggests that future training datasets will require improved sampling near these transitions if the models are to accurately capture their behavior.

The simulations that comprise the training datasets must also be evaluated in detail. In addition to the underlying CFD algorithms and material EOS used, the simulations must be stringently checked for both temporal and numerical convergence. Temporal convergence refers to how long a simulation requires after impact until the post-impact properties have converged to consistent value. Numerical convergence refers to the convergence of these properties as the particle resolution of the simulations is increased. Temporal convergence has the greatest effect on the precision of the models (the ability of the models to accurately reproduce the simulations), whereas numerical convergence is critical for training accurate models (the ability of the models to represent reality).

We have made an exhaustive analysis of the temporal convergence of the simulations used in this work (see Sect. 2.1.7), but the numerical convergence of the post-impact parameters is still an open question. It is important to note, however, that the *numerical* convergence of the underlying training dataset does not have an effect on the achievable accuracy of the data-driven models. Indeed, numerical convergence leads to at most minor a shift in the distribution post-impact values in the training dataset, which is easily relearned by the data-driven models.

The numerical convergence of the post-impact impact properties in this work has recently been evaluated in the context terrestrial planet formation (Meier et al. 2020). These results show that, of the post-impact properties here, only the rotation rate (Ω), obliquity (*θ*), and mixing ratio ($\delta ^{\mathrm{mix}}_{\mathrm{deb}}$) do not yet show numerical convergence at the particle resolutions used here. We have already pointed out that Ω is to be avoided on account of its failure to achieve temporal convergence, but the latter two properties require further investigation.

The datasets used to train and validate the data-driven models here include at least six additional dimensions to any previous study of its kind, as well as more expansive ranges in each of their dimensions. We have sampled asymptotic relative velocities of up to 10 times the escape velocity. Previous studies have considered much lower asymptotic relative velocities—indeed, they sampled lower *impact* velocities—than we have in this work. The high velocities considered in this work might seem excessive, but such velocities are needed to capture the low-probability collisions that can occur during planet formation. Indeed, recent studies have shown that it’s possible for planetary-sized objects to be exchanged between stars in a crowded stellar environment, leaving those objects on highly-eccentric orbits that could result in a collision (Hands et al. 2019). These velocities would be extremely fast and the ensuing collisions catastrophic.

### Feature importance

The data-driven models investigated here provide insight into the physical relationships between pre- and post-impact properties. While ML methods are often criticized for being so-called “black boxes”, advances in model interpretability have made data-driven methods powerful tools for understanding complex relationships. The Sobol’ indices shown in Fig. 13, the PCE feature selections reported in Table 7, and the SHAP values provided in Fig. 14 illustrate clearly the relationships between the pre- and post-impact properties.

**Table 7:** Features selected by PCE. For each post-impact property, the PCE algorithm selects a subset of features to use in the model. For most post-impact parameters, the algorithm selects pre-impact parameters related to the impact geometry (*γ*, $b_{\infty }$, $v_{\infty }$). Where other pre-impact properties have been selected, they tend to have a physically intuitive relationship to the post-impact property. Note that PCE did not select the pre-impact azimuthal orientations, $\phi _{\mathrm{targ}}$ and $\phi _{\mathrm{proj}}$, indicating that these properties generally do not play a role in determining collision outcomes

Parameter	Features selected
$M_{\mathrm{LR}}$	$M_{\mathrm{tot}}$, *γ*, $b_{\infty }$, $v_{\infty }$
$M^{\mathrm{norm}}_{\mathrm{LR}}$	*γ*, $b_{\infty }$, $v_{\infty }$
$F^{\mathrm{core}}_{\mathrm{LR}}$	*γ*, $b_{\infty }$, $v_{\infty }$, $F^{\mathrm{core}}_{\mathrm{targ}}$, $F^{\mathrm{core}}_{\mathrm{proj}}$
$J_{\mathrm{LR}}$	$M_{\mathrm{tot}}$, *γ*, $b_{\infty }$, $v_{\infty }$, $\Omega _{\mathrm{targ}}$, $\theta _{\mathrm{targ}}$
$\Omega _{\mathrm{LR}}$	*γ*, $b_{\infty }$, $v_{\infty }$, $\Omega _{\mathrm{targ}}$, $\theta _{\mathrm{targ}}$, $F^{\mathrm{core}}_{\mathrm{targ}}$
$\theta _{\mathrm{LR}}$	*γ*, $b_{\infty }$, $v_{\infty }$, $\Omega _{\mathrm{targ}}$, $\theta _{\mathrm{targ}}$
$F^{\mathrm{cond}}_{\mathrm{LR}}$	*γ*, $b_{\infty }$, $v_{\infty }$
$\delta ^{\mathrm{mix}}_{\mathrm{LR}}$	*γ*, $b_{\infty }$, $v_{\infty }$
$M_{\mathrm{SLR}}$	$M_{\mathrm{tot}}$, *γ*, $b_{\infty }$, $v_{\infty }$
$M^{\mathrm{norm}}_{\mathrm{SLR}}$	*γ*, $b_{\infty }$, $v_{\infty }$
$F^{\mathrm{core}}_{\mathrm{SLR}}$	*γ*, $b_{\infty }$, $v_{\infty }$, $F^{\mathrm{core}}_{\mathrm{targ}}$, $F^{\mathrm{core}}_{\mathrm{proj}}$
$J_{\mathrm{SLR}}$	$M_{\mathrm{tot}}$, *γ*, $b_{\infty }$, $v_{\infty }$
$\Omega _{\mathrm{SLR}}$	*γ*, $b_{\infty }$, $v_{\infty }$, $\Omega _{\mathrm{proj}}$, $F^{\mathrm{core}}_{\mathrm{proj}}$
$\theta _{\mathrm{SLR}}$	*γ*, $b_{\infty }$, $v_{\infty }$, $\theta _{\mathrm{targ}}$, $F^{\mathrm{core}}_{\mathrm{targ}}$, $\Omega _{\mathrm{proj}}$, $\theta _{\mathrm{proj}}$, $F^{\mathrm{core}}_{\mathrm{proj}}$
$F^{\mathrm{cond}}_{\mathrm{SLR}}$	$b_{\infty }$, $v_{\infty }$, $\Omega _{\mathrm{targ}}$, $F^{\mathrm{core}}_{\mathrm{proj}}$
$\delta ^{\mathrm{mix}}_{\mathrm{SLR}}$	$b_{\infty }$, $v_{\infty }$
$M_{\mathrm{deb}}$	$M_{\mathrm{tot}}$, $b_{\infty }$, $v_{\infty }$
$M^{\mathrm{norm}}_{\mathrm{deb}}$	$b_{\infty }$, $v_{\infty }$
$F^{\mathrm{Fe}}_{\mathrm{deb}}$	$b_{\infty }$, $v_{\infty }$, $F^{\mathrm{core}}_{\mathrm{targ}}$, $F^{\mathrm{core}}_{\mathrm{proj}}$
$\delta ^{\mathrm{mix}}_{\mathrm{deb}}$	*γ*, $b_{\infty }$, $v_{\infty }$, $F^{\mathrm{core}}_{\mathrm{targ}}$, $F^{\mathrm{core}}_{\mathrm{proj}}$
$\bar{\theta }_{\mathrm{deb}}$	*γ*, $v_{\infty }$, $\Omega _{\mathrm{targ}}$, $\theta _{\mathrm{targ}}$, $F^{\mathrm{core}}_{\mathrm{targ}}$, $\theta _{\mathrm{proj}}$
$\theta ^{\mathrm{stdev}}_{\mathrm{deb}}$	$M_{\mathrm{tot}}$, *γ*, $b_{\infty }$, $v_{\infty }$
$\bar{\phi }_{\mathrm{deb}}$	*γ*, $b_{\infty }$, $v_{\infty }$
$\phi ^{\mathrm{stdev}}_{\mathrm{deb}}$	*γ*, $b_{\infty }$, $v_{\infty }$, $F^{\mathrm{core}}_{\mathrm{targ}}$, $F^{\mathrm{core}}_{\mathrm{proj}}$

In general, both the Sobol’ indices and SHAP values indicate that the most important pre-impact properties are those related to the geometry and energy of the impact. These properties are the mass ratio (*γ*), asymptotic relative velocity ($v_{\infty }$), and asymptotic impact parameter ($b_{ \infty }$). For rotational quantities (*J*, Ω, and *θ*), the pre-impact rotational state of the associated body—target for the LR and projectile for the SLR—also play a significant role.

The Sobol’ analysis, along with the results of the PCE feature selection and SHAP values for the core mass fractions, also explain why the analytic PIM method does so well at predicting $F^{\mathrm{core}}_{\mathrm{LR}}$. In addition to the impact geometry, the feature importance metrics all indicate that the core mass fractions of the target ($F^{\mathrm{core}}_{\mathrm{targ}}$) and projectile ($F^{\mathrm{core}}_{\mathrm{proj}}$) are crucial in determining the $F^{\mathrm{core}}_{\mathrm{LR}}$. This would add further weight to the idea that, with the exception of hit-and-run collisions, the cores of the target and projectile tend to merge.

The Sobol’ analysis, associated PCE feature selections, and SHAP values pointedly show that the pre-impact azimuthal orientations ($\phi _{\mathrm{targ}}$, $\phi _{\mathrm{proj}}$) play an insignificant role in determining the outcome the post-impact properties. While this would suggest that these parameters can be ignored in future studies (in order to reduce the number of pre-impact parameters), it would be prudent to first assess their contributions to post-impact properties not considered here, as well as in higher fidelity simulations.

### Ease of implementation

The data-driven models developed and evaluated in this work operate by fundamentally distinct underlying methodologies, both from a mathematical and algorithmic point of view. Therefore, an important consideration of these models going forward is their complexity and relative ease of implementation into existing or future N-body codes. There are a number of considerations that need to be taken into account regarding practical development and use of the models. First, what are the dataset requirements? Second, what are the computational resources required to train the models? And third, what are the limitations when integrating the model into an existing N-body integrator, both in terms of speed and complexity?

Most of the improvement in performance relative to training set size is achieved up to sizes of roughly a thousand, with marginal increases thereafter (Fig. 12). The results would therefore suggest that datasets of approximately a few thousand simulations would be suitable for most post-impact properties, such as masses or core mass fractions. For other, more difficult-to-emulate post-impact properties, such as $\theta _{\mathrm{SLR}}$, larger dataset sizes are advisable. The datasets should additionally be large enough to allow for robust training and validation practices, such as the HPO with k-fold cross-validation used in this work.

While Fig. 12 shows that the dataset requirements are similar for the data-driven models, the computational resources needed to train, optimize, and validate them are not. We have avoided an explicit comparison between training times and memory requirements, on one hand because the models only have to be trained once and, on the other hand, because not all models were trained on the same hardware, rendering a fair comparison problematic. However, the qualitative differences between methods is worth mentioning. As training set sizes increase, the time required to train, optimize, and validate the models increases. The times required to train and optimize the MLP, PCE, and XGB models are negligible for the datasets investigated here, whereas the times required to train the GP models grow quickly. The GP models lack of scalability quickly became a problem and we were consequently unable to perform HPO on GP models above $N = 1000$. Therefore, on account of both its poor performance relative to the other data-driven methods and its poor scalability, we conclude that GPs are not well suited to the problem at hand, especially given that training set sizes are expected to continue growing.

In terms of accessibility, neural networks (such as MLPs) and XGBoost are both extremely popular ML methods and as a result many implementations from Python into other languages are readily available. Likewise, PCEs have already been used in other astrophysical applications to great success (Knabenhans et al. 2019). In order to utilize these models in an N-body integrator, a way to store their architecture, hyperparameters, and coefficients, weights, and/or biases is required. These parameters must be readily accessible by the integrator, and therefore speed and memory requirements must be considered. For example, while the matrices containing the weights and biases of neural networks can grow very large, the MLPs investigated here are relatively small networks, with no more than three hidden layers with up to at most 24 neurons each. Therefore, the associated weights and biases matrices are negligibly small and can be used without issue in existing N-body codes. Given the excellent performance of the MLP models here, it is unlikely that the number of layers or neurons per layer will grow significantly in the future.

We provide all of the models reported in this study as serialized joblib files at https://github.com/mtimpe/aegis-emulator.

### Future work

The data-driven emulation strategies explored here have proven to be extremely flexible and robust. This suggests that the greatest benefit to collision models and subsequent emulation-based N-body simulations will come from improvements to the datasets used to train the models. The most obvious improvements are needed in the underlying simulation methods (e.g., smoothed-particle hydrodynamics). Higher resolution simulations, improvements to the underlying CFD algorithms, as well as improved and additional equations of state are the obvious improvements in this respect.

In particular, both the classification and regression models tend to see their worst performance at the interface between collision outcome regimes (e.g., merging versus hit-and-run). Therefore, datasets intended to be used as training data for data-driven models should focus on these regions.

An important caveat that bears repeating in all machine learning applications is that data-driven methods will faithfully emulate the data they are given. Therefore, the accuracy of the underlying numerical methods and distributions of the input features are critical considerations. Unfortunately, there is as of yet no comprehensive study for planetary collisions comparing the results of different CFD methods (e.g., AMR, SPH) or implementations of those methods in the literature. Therefore, while data-driven techniques may achieve excellent accuracies, their performance does not give any information as to the accuracy of the underlying simulations. Thus, a comprehensive code comparison for planetary collision codes would be of great benefit to the community.

We have not attempted to impose any physical limitations on our data-driven models in this work. Thus, while the predictions of the models may be accurate, they may not be physically self-consistent. In the context of N-body studies, the conservation of mass and momentum is of particular importance and therefore a robust method is needed to ensure the physical self-consistency of the models. In a forthcoming paper, we explore strategies for emulating physically conserved quantities, such as mass and angular momentum. Multi-target regression models may prove useful for imposing physical self-consistency on the models, which at present must be achieved entirely *ex post*.

In addition, ML and UQ are rapidly advancing fields and are used in a wide range of applications. More advanced techniques (e.g., ensemble learning) are therefore likely to prove useful in the future. Such techniques were beyond the scope of this paper, but the models investigated here may benefit from them significantly.

## Conclusions

Using a new set of 14,856 SPH simulations of collisions between differentiated, rotating planets, we have demonstrated that data-driven methods from machine learning (eXtreme Gradient Boosting and multi-layer perceptrons) and uncertainty quantification (Gaussian processes and polynomial chaos expansion) can accurately predict the outcome of a wide range of post-impact properties. Of these data-driven models, multi-layer perceptrons and XGBoost models consistently achieved the best performances. We additionally showed that extant analytic (perfect merging) and semi-analytic methods (IEM and EDACM) perform poorly compared to data-driven methods when effects such as variable core mass fractions and pre-impact rotation are included.

In terms of training dataset requirements, the best performances are reached around a few thousand collisions, however some parameters continue to show improvement, suggesting that larger training datasets will be useful in the future. Particular attention should be paid to the pre-impact parameter space near transitions between outcome regimes (e.g., merging and hit-and-run), as this is where data-driven models perform worst.

We have leveraged Sobol’ indices from polynomial chaos expansion (PCE) and SHAP values from XGboost (XGB) in order to quantify relationships between pre- and post-impact quantities. These metrics reveal that the impact geometry is usually the most important factor in predicting most post-impact properties, however in some cases other pre-impact properties are important.

We summarize the several notable conclusions here:

- Data-driven classification methods, including multi-layer perceptrons (MLPs) and XGBoost models (XGB) are able to accurately classify collision outcomes to approximately 95% accuracy. The misclassified collisions are concentrated at the transitions between collision outcome regimes (e.g., merging to hit-and-run).
- Data-driven regression methods can achieve high accuracy for a wide range of post-impact properties. Of the data-driven methods considered here, multi-layer perceptrons (MLPs), polynomial chaos expansion (PCE), and XGBoost (XGB) perform best. Gaussian processes (GPs) perform significantly worse and do not scale to the dataset sizes considered here.
- Data-driven methods are able to generalize to any quantifiable post-impact parameter. Extant analytic and semi-analytic methods are limited to a narrow range of post-impact properties and achieve far lower accuracy.
- Further improvements to collision emulation should focus on the underlying training data. In particular, better sampling of the transition regimes is needed. The numerical convergence of the simulations that comprise the training data also needs further analysis.

## Supplementary Information

Below is the link to the electronic supplementary material.

Supplementary information (ZIP 48.3 MB)

## Data Availability

The simulations supporting the conclusions of this article are available in the Dryad repository: https://doi.org/10.5061/dryad.j6q573n94. The machine learning models and training pipeline reported in this work are available on GitHub: https://github.com/mtimpe/aegis-emulator.

## Appendix A: Definitions

### Pre-impact trajectory

In this work we use the asymptotic relative velocity ($v_{\infty }$) and asymptotic impact parameter ($b_{\infty }$) to specify the initial trajectory of the projectile in the target’s frame of reference. Most previous studies have used the associated quantities at the moment of impact—$b_{\mathrm{imp}}$ and $v_{\mathrm{imp}}$, respectively. Therefore, we provide formulae for converting quickly between the two. These conversions can be derived from the conservation of energy and angular momentum. We first calculate $v_{\mathrm{imp}}$ from $v_{\infty }$,

10 $$ v^{2}_{\mathrm{imp}} = v^{2}_{\infty } + \frac{2 G M_{\mathrm{targ}}}{R_{\mathrm{crit}}}, $$

where *G* is the gravitational constant, $M_{\mathrm{targ}}$ is the mass of the target, and $R_{\mathrm{crit}} = R_{\mathrm{targ}} + R_{\mathrm{proj}}$ (using the non-rotating radii of the bodies). The impact parameter ($b_{\mathrm{imp}}$) can then be obtained via,

11 $$ b_{\mathrm{imp}} = b_{\infty } \frac{v_{\infty }}{v_{\mathrm{imp}}}. $$

Note that this conversion assumes that the target and projectile are perfectly rigid bodies, which is not the case in either reality or in CFD simulations. Therefore, the conversion is an approximation, because the shapes, rotation rates, and orientations of the target and projectile, as well as their pre-impact trajectories, will be altered by gravitational interactions prior to impact.

### Accretion efficiency

The accretion efficiency, *ξ*, quantifies how much of the projectile was accreted onto the target (referred to as the largest remnant (LR) post-impact),

12 $$ \xi = \frac{M_{\mathrm{LR}} - M_{\mathrm{targ}}}{M_{\mathrm{proj}}} . $$

However, if the collision is sufficiently disruptive, the accretion efficiency can take on negative values. The minimum accretion efficiency is set by $-1/\gamma $.

### Iron content

The core mass fraction ($F^{\mathrm{core}}_{\mathrm{body}}$) is a measure of the iron in either the target, projectile, LR, or SLR, relative to the body’s total mass. In the simulations investigated here, the SPH particles that comprise the pre-impact bodies are either iron or granite. Thus, it is straightforward to calculate the iron (i.e., core) mass fraction,

13 $$ F^{\mathrm{core}}_{\mathrm{body}} = \frac{N_{\mathrm{iron}}}{N_{\mathrm{gran}} + N_{\mathrm{iron}}}, $$

where $N_{\mathrm{gran}}$ and $N_{\mathrm{iron}}$ are the number of granite and iron particles, respectively. Similarly, while the debris doesn’t have a core, it’s iron mass fraction ($F^{\mathrm{Fe}}_{\mathrm{deb}}$) is calculated in the same manner.

### Melt fraction

The melt fraction ($F^{\mathrm{melt}}_{\mathrm{body}}$) is the fraction of the post-impact material that is in a non-condensed state, as defined by the Tillotson EOS. This is useful for estimating the depth of the post-impact magma ocean. Note that the Tillotson EOS doesn’t allow for mixed states, so this quantity should be be used with caution and only as a rough estimate of the post-impact melt fraction. Our motivation for including it here was to show that data-driven emulation can be extended to parameters which have not been considered before. Improvements to the EOS in future datasets will improve the usefulness of quantities such as this.

### Mixing ratio

The mixing ratio ($\delta ^{\mathrm{mix}}_{\mathrm{body}}$) in this study is defined as the fraction of “foreign” material present in the LR, SLR, or debris. While this gives no information about the source of the foreign material (i.e., whether foreign refers to the target or projectile), it is easier to regress because it does not suffer from the non-negligible number of hit-and-run collisions in which the projectile becomes the LR and the target the SLR. These cases create a significant discontinuity in the response surface, which makes it difficult to regress. However, coupled with a classifier that identifies the dominant material source, the mixing ratio is a powerful tool for studying compositional exchange during collisions.

### Debris field spatial distribution

The mean and standard deviations of the debris altitude (*θ*) and azimuth (*ϕ*) are a way to quantify the direction and spread of the post-impact debris field. The altitude of the debris particles are measured relative to the initial collision plane and the azimuths are measure relative to an arbitrary reference direction within the collision plane. Here, the azimuths are measured relative to the initial velocity vector of the projectile in the reference frame of the target.

## Appendix B: Perfectly Inelastic Merging (PIM)

Perfectly inelastic merging (PIM) assumes perfect conservation of mass and momentum, allowing a set of simple analytic formulae to be derived. The formulae predict the mass and core mass fraction of the largest (and only) remnant (referred to as the LR for consistency). During the collision, there is no net conversion of kinetic energy to other forms such as heat, noise, or thermal energy. Mass is conserved in the only remant, such that

14 $$ M_{\mathrm{LR}} = M_{\mathrm{targ}} + M_{\mathrm{proj}} , $$

where $M_{\mathrm{targ}}$ and $M_{\mathrm{proj}}$ are the masses of the target and projectile, respectively.

We can similarly calculate the core mass fraction of the LR by noting that, in a perfect merger, the cores of the target and projectile will be incorporated in their entirety into the LR,

15 $$ F^{\mathrm{core}}_{\mathrm{LR}} = \frac{F^{\mathrm{core}}_{\mathrm{targ}} M_{\mathrm{targ}} + F^{\mathrm{core}}_{\mathrm{proj}} M_{\mathrm{proj}}}{M_{\mathrm{targ}} + M_{\mathrm{proj}}} , $$

where $F^{\mathrm{core}}_{\mathrm{targ}}$ and $F^{\mathrm{core}}_{\mathrm{proj}}$ are the core mass fractions of the target and projectile, respectively.

PIM can also predict the rotational angular momentum, rotation rate, and obliquity of the LR. The rotation model assumes perfect angular momentum conservation and assumes that the orbital angular momentum of the collision remains with the post-impact remnant. The angular momentum in the system is determined by the rotational angular momenta of the target and projectile and the orbital angular momentum of the pre-impact trajectory,

16 $$ \vec{J_{\mathrm{LR}}} = \vec{J_{\mathrm{targ}}} + \vec{J_{\mathrm{proj}}} + J_{\mathrm{orb}} \vec{\hat{k}} , $$

where $J_{\mathrm{orb}} = M_{\mathrm{proj}} b_{\infty } v_{\infty }$ is the orbital angular momentum delivered by the impact. The obliquity of the remnant ($\theta _{\mathrm{LR}}$) is subsequently measured relative to the unit vector normal to the collision plane ($\hat{z} = [0,0,1]$). The rotation rate of the remnant can be calculated from the magnitude of the angular momentum vector,

17 $$ \Omega _{\mathrm{LR}} = \frac{\lvert \vec{J_{\mathrm{LR}}\rvert }}{I_{\mathrm{LR}}} , $$

where $I_{\mathrm{LR}}$ is the moment of inertia of the LR. Because the bodies themselves are not physically resolved in PIM, the moment of inertia of the LR must be analytically approximated (and in turn the radius),

18 $$ I_{\mathrm{LR}} = \frac{2}{5} M_{\mathrm{LR}} R^{2}_{\mathrm{LR}} ,\qquad R_{\mathrm{LR}} = \biggl( \frac{3 M_{\mathrm{LR}}}{4 \pi \rho _{\mathrm{LR}}} \biggr)^{1/3} , $$

where $\rho _{\mathrm{LR}} = \rho _{\mathrm{gran}} ( 1 - F^{\mathrm{core}}_{\mathrm{LR}} ) + \rho _{\mathrm{iron}} F^{\mathrm{core}}_{\mathrm{LR}}$. The density of iron is $\rho _{\mathrm{iron}} = 7.86\text{ g}/\text{cm}^{3}$ and $\rho _{\mathrm{gran}} = 2.7\text{ g}/\text{cm}^{3}$ is the density of granite.

## Appendix C: Leinhardt and Stewart (2012) (EDACM)

EDACM as introduced by Leinhardt and Stewart (2012; hereafter LS12) is a set of analytic relations defined for multiple distinct (non-overlapping) collision regimes. These collision regimes are delineated by a combination of $b_{\mathrm{imp}}$, $v_{\mathrm{imp}}$, $Q_{R}$, and $Q^{\prime \star }_{\mathrm{RD}}$. Here, $b_{\mathrm{imp}}$ and $v_{\mathrm{imp}}$ are the impact parameter and velocity at the moment of impact, $Q_{R}$ is the specific impact energy, and $Q^{\prime \star }_{\mathrm{RD}}$ is the catastrophic disruption threshold.

We have followed the implementation of EDACM as provided in LS12 for the LR and SLR properties, and its subsequent N-body implementation (Chambers 2013) for the debris properties. LS12 provides a step-by-step procedure for calculating $Q^{\prime \star }_{\mathrm{RD}}$, the projectile’s interacting mass $M_{\mathrm{interact}}$, and the velocities for the onset of erosion $v_{\mathrm{erosion}}$ and super-catastrophic disruption (SCD) $v_{\mathrm{scd}}$, which are used below. These calculations are beyond the scope of this appendix, but we direct the reader to Appendix A of LS12 as a reference. Here, we provide a brief overview of EDACM and point out where our implementation differs.

### Perfect merging

In EDACM, The mutual escape velocity is calculated using the interacting mass in the collision,

19 $$ v^{\prime }_{\mathrm{esc}} = \sqrt{ \frac{2 G M^{\prime }}{R^{\prime }}} , \qquad R^{ \prime } = \biggl( \frac{3 M^{\prime }}{4 \pi \rho _{1}} \biggr)^{1/3} , $$

where $M^{\prime } = M_{\mathrm{targ}} + M_{\mathrm{interact}}$ and $M_{\mathrm{interact}}$ is the interacting mass of the projectile. $\rho _{1} = 1\text{ g}/\text{cm}^{3}$ is an assumed bulk density (see Table 8) of the bodies. This bulk density is low for planetary-scale bodies, but we use it here for consistency with previous implementations (Leinhardt and Stewart 2012; Chambers 2013). If the impact velocity is less than the escape velocity ($v_{\mathrm{imp}} < v^{\prime }_{\mathrm{esc}}$), then the outcome is assumed to be a perfect merger and EDACM is therefore equivalent to PIM in this regime,

20 $$ M^{\mathrm{norm}}_{\mathrm{LR}} = 1. $$

**Table 8:** Summary of variables used in EDACM and the values used in our implementation. All values are those suggested in LS12. However, we note that our determination of the target/projectile radii and bulk densities are different, having been calculated for differentiated bodies

Parameter	Value	Description
$\rho _{1}$	1 g/cm^3^	Assumed bulk density
*η*	−1.5	Exponent of the power-law fragment distribution in the SCD regime
$c^{\star }$	1.9	Head-on equal-mass disruption energy in units of specific gravitational binding energy
*μ̄*	0.36	Velocity exponent in coupling parameter
*β*	2.85	Slope of fragment size distribution
$N_{\mathrm{LR}}$	1	Disruption (*γ* ≤ 0.95)
$N_{\mathrm{SLR}}$	2	Disruption (*γ* ≤ 0.95)
$N_{\mathrm{LR}}$	2	Hit & run (*γ*>0.95)
$N_{\mathrm{SLR}}$	4	Hit & run (*γ*>0.95)

### Disruption and accretion regimes

For impact velocities exceeding the escape velocity ($v_{\mathrm{imp}} \ge v^{\prime }_{\mathrm{esc}}$), collisions are further broken up into grazing ($b_{\mathrm{imp}} > b_{\mathrm{crit}}$) and non-grazing ($b_{\mathrm{imp}} < b_{\mathrm{crit}}$),

21 $$ b_{\mathrm{crit}} = \frac{R_{\mathrm{targ}}}{R_{\mathrm{targ}} + R_{\mathrm{proj}}} , $$

where $R_{\mathrm{targ}}$ and $R_{\mathrm{proj}}$ are the radii of the target and projectile, respectively. The radii are determined via the bulk densities,

22 $$ R_{\mathrm{body}} = \biggl( \frac{3 M_{\mathrm{body}}}{4 \pi \rho _{\mathrm{body}}} \biggr)^{1/3} . $$

Here, we differ from LS12 in that we are using differentiated bodies, and therefore we calculate the bulk density of our bodies as,

23 $$ \rho _{\mathrm{body}} = \rho _{\mathrm{gran}} \bigl( 1 - F^{\mathrm{core}}_{\mathrm{body}} \bigr) + \rho _{\mathrm{iron}} F^{\mathrm{core}}_{\mathrm{body}} , $$

where the density of iron is $\rho _{\mathrm{iron}} = 7.86\text{ g}/\text{cm}^{3}$ and $\rho _{\mathrm{gran}} = 2.7\text{ g}/\text{cm}^{3}$ is the density of granite.

For non-grazing impacts, where $v^{\prime }_{\mathrm{esc}} < v_{\mathrm{imp}} < v_{\mathrm{scd}}$, the impact is in either the *disruption* or *partial accretion* regime. In these regimes, a universal law for $M^{\mathrm{norm}}_{\mathrm{LR}}$ applies,

24 $$ M^{\mathrm{norm}}_{\mathrm{LR}} = 1 - 0.5 \frac{Q_{R}}{Q^{\prime \star }_{\mathrm{RD}}} . $$

### Hit & run regime

Grazing collisions ($b_{\mathrm{imp}} > b_{\mathrm{crit}}$) where $v^{\prime }_{\mathrm{esc}} < v_{\mathrm{imp}} < v_{\mathrm{erosion}}$ are defined as hit & run collisions. In this regime, $M_{\mathrm{LR}}$ is again calculated by the universal law (Eq. (24)). If, in the resulting prediction, $M_{\mathrm{LR}} < M_{\mathrm{targ}}$, then the outcome is a single large remnant (i.e., the LR) and debris. However, if $M_{\mathrm{LR}} \ge M_{\mathrm{targ}}$, then the LR is assumed to be the original target ($M_{\mathrm{LR}} = M_{\mathrm{targ}}$) and the SLR is calculated assuming the “reverse collision” scenario. This scenario is described in detail in LS12, and the resulting relation used to predict $M^{\mathrm{norm}}_{\mathrm{SLR}}$ is,

25 $$ M^{\mathrm{norm}}_{\mathrm{SLR}} = \frac{ ( 3 - \beta ) ( 1 - N_{\mathrm{LR}} M^{\mathrm{norm}}_{\mathrm{LR}} )}{N_{\mathrm{SLR}} \beta }, $$

where $\beta = 2.85$, $N_{\mathrm{LR}}=1$, $N_{\mathrm{SLR}}=2$, and $M^{\mathrm{norm}}_{\mathrm{LR}}$ is determined by the universal law (Eq. (24)). This relation needs to be modified slightly for nearly equal-mass ($\gamma \sim 1$) hit & run collisions. We modify the relation according Leinhardt and Stewart (2012) when $\gamma > 0.95$.

### Super-catastrophic disruption regime

For all impact angles/parameters, a collision is in the SCD regime if $v_{\mathrm{imp}} > v_{\mathrm{scd}}$. In this regime, $M^{\mathrm{norm}}_{\mathrm{LR}}$ is determined using a power-law relation,

26 $$ M^{\mathrm{norm}}_{\mathrm{LR}} = \frac{0.1}{1.8^{\eta }} \biggl( \frac{Q_{R}}{Q^{\prime \star }_{\mathrm{RD}}} \biggr)^{\eta }, $$

where $\eta = -1.5$.

### Debris

Following the EDACM implementation for the N-body integrator Mercury (Chambers 2013), the mass not allocated to the LR (in the case of non-hit-and-run collisions) is split into one or more equal-mass fragments, where the masses are as close as possible to, but always more massive than, $M_{\mathrm{frag}} = 4.7 \times 10^{-3}~\mathrm{M}_{\oplus }$. This limit was set by the computational limits of the Mercury integrator at the time of the study. With the LR acting as the center of mass, the trajectories of the resulting fragments are arranged at uniform intervals around a circle lying in the collision plane. This results in the a mean altitude of the debris fragments $\bar{\theta }_{\mathrm{deb}}$ of 0 degrees with a standard deviation $\theta ^{\mathrm{stdev}}_{\mathrm{deb}}$ of 0 degrees. The mean azimuth of the fragments $\bar{\phi }_{\mathrm{deb}}$ is 180 degrees. The standard deviation of the debris fragments $\phi ^{\mathrm{stdev}}_{\mathrm{deb}}$ is that of a uniform distribution from 0–360, which is 103.9 degrees in this case.

### Mantle stripping

EDACM predicts the core mass fractions of its remnants by using a mantle-stripping prescription introduced in earlier work (Marcus et al. 2010). This prescription is based on simulations of collisions in which the colliding bodies have chondritic compositions (i.e., $F^{\mathrm{core}}_{\mathrm{targ}} = F^{\mathrm{core}}_{\mathrm{proj}} = 0.33$).

## Appendix D: Polynomial Chaos Expansion (PCE)

PCE is a probabilistic method whereby the model output is projected on a basis of orthogonal stochastic polynomials in the random inputs. The stochastic projection provides a compact and convenient representation of the model output variability with regards to the inputs. In this work, PCEs are used to represent the relationships between the pre- and post-impact parameters of the collisions. The PCE coefficients are obtained from a non-intrusive regression based method. PCE represents the post-impact parameters by a series expansion,

27 $$ \hat{y} = \sum^{+\infty }_{\alpha \in N^{\mathcal{M}}} y_{\alpha } \Psi _{\alpha }(\vec{x}), $$

where *ŷ* is the predicted post-impact value, $y_{\alpha }$ are the coefficients to be calculated and $\Psi _{\alpha }$ are the multivariate orthonormal basis functions. Orthonormality for PCE basis functions is always defined with respect to a weighting function given by the joint probability distribution $f_{\mathbf{X}}(\vec{x})$ of the sampled input features,

28 $$\begin{aligned} \bigl\langle \Psi _{\mathbf{n}}(\vec{x}),\Psi _{\mathbf{m}}(\vec{x}) \bigr\rangle &\equiv \int _{{\mathcal{D}}_{\mathbf{X}}}\Psi _{ \mathbf{n}}(\vec{x}) \Psi _{\mathbf{m}}(\vec{x})f_{\mathbf{X}}( \vec{x})\, {\mathrm{d}}x^{d} \\ &= \delta _{\mathbf{n}\mathbf{m}} , \end{aligned}$$

where ${\mathcal{D}}_{\mathbf{X}}$ is the full input space and *d* is its dimensionality and $\delta _{\mathbf{n}\mathbf{m}}$ is the Kronecker delta. In our case, this input distribution is chosen to be uniform in all $d=12$ dimensions (classic LHS; see Sect. 2.1.2) as we do not want to impose any non-trivial priors on the collisional input parameters. Following Xiu and Karniadakis (2002), in this work all the basis functions hence need to be based on Legendre polynomials,

29 $$\begin{aligned}& P_{0} (x) = 1 , \end{aligned}$$

30 $$\begin{aligned}& P_{1} (x) = x , \end{aligned}$$

31 $$\begin{aligned}& (n+1) P_{n+1} (x) = (2n+1) x P_{n}(x) - n P_{n-1}(x) , \end{aligned}$$

where *n* is the polynomial order and the norm of the *n*th Legendre polynomial is,

32 $$ \lVert {P_{n}} \rVert ^{2} = \frac{1}{2n+1} , $$

with which we can define the normalized Legendre polynomials,

33 $$ \tilde{P_{n}}(x) = \sqrt{2n+1} P_{n}(x) . $$

In order to construct the multivariate basis functions from the univariate Legendre polynomials, we calculate the tensor product,

34 $$ \Psi _{\mathbf{n}}(\vec{x}) \equiv \prod _{i=1}^{12}P^{i}_{n_{i}}(x_{i}) . $$

The Legendre polynomials are further defined over the interval $[-1,1]$. This is why all input features need to be linearly mapped into a 12D unit hypercube before they can be passed into the individual Legendre polynomials.

### Truncation of the polynomial basis

The most straightforward way of truncating a PCE is via a maximal polynomial order. Note that this means that the *total* polynomial order may not exceed this maximum. The subscript *α* is a multi-index specifying uniquely how a basis function of order *n* is composed by individual Legendre polynomials: The first entry in the multi-index is given by the order of the first factor in (34), the second index refers to the order of the second factor and so on. The sum of all entries in the multi-index may thus never be larger than the maximum polynomial order.

### Expansion coefficients

The goal of PCE regression is to determine the coefficients $y_{\alpha }$ of the expansion, truncated at some polynomial order, given a training data. In PCE the underlying model is assumed to take a random variable as input and, as a consequence, the output of the model has to be treated as a random variable as well. In fact, PCE maps probability distributions of input features to probability distributions of output. Because PCE belongs to the class of spectral decomposition methods, its expansion coefficients decrease polynomially, leading to favorable convergence properties. As it turns out, sometimes the prediction performance can be improved if only carefully chosen terms remain in the expansion while others are left out. There are two more hyperparameters in this approach that further reduce the number of terms kept in the expansion. The expansion coefficients, moreover, contain information about the global output uncertainty given the uncertain input features. This latter property of PCE allows us to quantify feature importance via the Sobol’ indices. The OLS algorithm is used to compute the coefficients in the polynomial chaos expansion.

### In this work

The PCE regression models in this work are constructed as follows: first, for any given target, a computationally cheap version of PCE based on an ordinary least squares (OLS) loss function is computed. This allows us to quantify which features are relevant for the current target via Sobol’ analysis (see Sect. 3.7). We only retain those features with a total Sobol’ index larger than 1% (as otherwise the next step would be computationally too demanding). Based on this reduced set of features the PCE is then computed a second time. This time the PCE is obtained by minimization of a least squares loss function which is augmented by a penalty term through which a sparse representation of the final emulator is enforced. The loss function is minimized with the least-angle regression (LAR) algorithm (Efron et al. 2004). For an in-depth introduction to PCE, we refer the reader to Knabenhans et al. (2019) and references therein.

## Appendix E: Gaussian Processes (GP)

GPs are a non-parametric method that finds a distribution over the possible functions $f(x)$ that are consistent with the observed data (Rasmussen and Williams 2005). They are stochastic processes, such that every finite collection of its random variables has a multivariate normal distribution. The distribution of a GP is the joint distribution of all of its random variables. The function to be modeled is therefore represented as a stochastic process *f* (i.e., a collection of random variables indexed by some variable $x\in \mathcal{X}$),

35 $$ f = f(x):x\in \mathcal{X} , $$

where we approximate *f* with a GP. GPs define a distribution over the function’s values at a finite, but arbitrary, set of points ($x_{1}, \ldots, x_{N}$), assuming that $p(f(x_{1}),\ldots,f(x_{N}))$ is jointly Gaussian, with a mean $\mu (x)$ and covariance $\sigma (x)$ given by $\sigma _{ij} = k (x_{i},x_{j})$, where *k* is a positive definite kernel function. The key idea is that if $x_{i}$ and $x_{j}$ are deemed by the kernel to be similar, then it expects the output of the function at those points to be similar too.

In regression problems, we are interested in predicting the value $y_{i}$ of $f(x)$ at a specific points $x_{i}$. In the general case, observations are noisy, which means that we observe,

36 $$ y_{i} = f(x_{i}) + \varepsilon , $$

where *ε* is assumed to be independent and identically distributed Gaussian noise with variance $\sigma _{n}^{2}$. The prior on the noisy observation becomes

37 $$ \operatorname{cov}(y_{i},y_{j}) = k(x_{i},x_{j}) + \sigma _{n}^{2}\delta _{ij} , $$

where $k(x_{i},x_{j})$ is the kernel and $\delta _{ij}$ is the Kronecker delta function. Typically, the value of the prediction for some input $x_{i}$ is given by the mean of *f* at $x_{i}$.

### Kernel function

Machine learning algorithms that involve a GP use kernel functions to measure similarity between points and predict the value of an unseen point from training data. The prediction is an estimate for the unseen point based on the kernel function. The Gaussian radial basis function (RBF) kernel is commonly used, however in this work we test multiple kernels, including the constant, Matérn ($\nu =3/2$), rational quadratic, and RBF kernels (see Table 4).

In this work, we use scikit-learn’s open-source implementation of GPs. The hyperparameters of the kernel are optimized during fitting of the GP by maximizing the log-marginal-likelihood (LML) based on the chosen optimizer (we use scikit-learn’s default optimizer). As the LML may have multiple local optima, the optimizer is started repeatedly by specifying the number of restarts. The noise level in the targets is specified by *α* and can be helpful for dealing with numerical issues during fitting. We test models without noise and with $\alpha = 10^{-2}$.

## Appendix F: eXtreme Gradient Boosting (XGB)

XGBoost (XGB) is a scalable, open source machine learning algorithm for tree boosting (Chen and Guestrin 2016). For a given dataset with *n* examples and *d* features, a tree ensemble model uses *K* additive functions to predict the output,

38 $$ \hat{y}_{i} = \phi (\vec{x}_{i}) = \sum ^{K}_{k=1} f_{k}( \vec{x}_{i}),\quad f_{k} \in \mathcal{F} , $$

where $\hat{y}_{i}$ is the predicted output value for a given set of input features $\vec{x}_{i}$, $\mathcal{F}$ is the function space of all possible classification and regression trees (CART). Each $f_{k}$ corresponds to an independent tree structure *q* with leaf weights *w*. To learn the set of functions used in the model, XGB minimizes the following *regularized* objective function,

39 $$ \mathcal{L} (\phi ) = \sum_{i} l( \hat{y}_{i},y_{i}) + \sum_{k} \Omega (f_{k}) , $$

where *l* is a differentiable convex loss function that measures the difference between the prediction $\hat{y}_{i}$ and the target $y_{i}$. XGB’s default loss function for regression, which we use in this work, is the squared error, $l = (\hat{y}_{i} - y_{i})^{2}$. The second term Ω is a regularization term that penalizes the complexity of the model, which helps to avoid over-fitting.

XGB is a decision-tree-based ensemble machine learning algorithm that uses a gradient boosting framework (Chen and Guestrin 2016). Gradient tree boosting considers a function $h(x;\vec{a}_{m})$, which is a small regression tree,

40 $$ f(\vec{x};\{\beta _{m}, \vec{a}_{m} \}_{1}^{M}) = \sum_{m=1}^{M} \beta _{m} h(x;\vec{a}_{m}) , $$

where the parameters $\vec{a}_{m}$ are the splitting variables (i.e., on which input feature does the node make the split), split locations (i.e., in what location or value of the input variable to make the split) and number of terminal nodes, which we fix to be *L*. In this work, the splitting variables are the pre-impact parameters in Table 1.

During training, at each iteration *m*, a regression tree partitions the *x*−(input) space into *L*-disjoint regions $\{R_{l,m}\}_{l=1}^{L}$ and predicts a separate constant value in each one. For some input *x⃗*, the output of the weak learner can be written as

41

where $\bar{y}_{lm}$ is the value predicted in region $R_{lm}$. The model $f(\vec{x})$ is updated, at each iteration *m*, as

42 $$ f_{m}(\vec{x}) = f_{m-1}(\vec{x}) + \beta _{m} h(\vec{x};\vec{a}_{m}) , $$

where the coefficients $\beta _{m}$ and the parameters $\vec{a}_{m}$ are jointly obtained by minimizing

43 $$ (\beta _{m},\vec{a}_{m}) = \arg \min _{\vec{a},\beta } \sum_{i=1}^{N} \bigl[ \tilde{y}_{i} - \beta h(\vec{x}_{i};\vec{a}) \bigr]^{2} , $$

where the residuals are given by

44 $$ \tilde{y}_{i} = - \biggl[ \frac{\partial }{\partial f_{m-1}(x_{i})} \Phi \bigl(y_{i}, f_{m-1}(\vec{x}_{i})\bigr) \biggr],\quad i = 1,N $$

and an arbitrary, differentiable loss function $\Phi (y,f(\vec{x}))$. This loss function could be, for example, mean squared error loss, or Huber loss. A more efficient algorithm is presented in Chen and Guestrin (2016), in which the search for best split is not achieved through an exact greedy algorithm (which requires to search for all possible splits on all features), but rather by an approximate algorithm, which proposes candidate splitting points according to percentiles of feature distribution.

In the XGB models used in this work, we use squared error as the loss function, a learning rate of $\nu =0.1$, and a L1 regularization term on the weights of $\alpha = 10$.

## Appendix G: Multi-Layer Perceptrons (MLP)

Multi-layer perceptrons (MLP) are a type of deep, feed-forward, artificial neural network that consist of three or more layers (Rumelhart et al. 1986). These layers include an input layer, output layer, and one or more hidden layers. Each of these layers is composed of a variable number of nodes (also called *neurons*). The layers in a MLP are fully connected, such that each node in one layer connects—with a certain weight, $w_{ij}$—to every node in the following layer. With the exception of the input layer, the nodes are wrapped in non-linear functions known as *activation functions* to regularize their output. The resulting network is a supervised learning algorithm that learns a function $f(\cdot ): R^{d} \mapsto R^{o}$ by training on a dataset, where *d* is the number of input dimensions and *o* is the number of output dimensions. Given a set of features $\vec{x} = x_{1}, x_{2}, \ldots , x_{d}$ and a corresponding target *y* (in the case of single-target models), it can learn a non-linear function approximator for either classification or regression. In this work, we train MLPs to learn a mapping from a 12-dimensional input space (the pre-impact parameters in Table 1) to a scalar output space (i.e., one of the post-impact parameters in Table 3) The resulting regression models are then non-linear functions that map $f(\vec{x}): R^{12} \mapsto R^{1}$.

While the input nodes provide the inputs, the hidden layers are the computational workhorse of the network. The output of a node in a hidden layer can be represented as,

45 $$ y = \psi \Biggl( \sum^{N}_{i=1} w_{i} x_{i} + b_{i} \Biggr) , $$

where *ψ* is the activation function and $w_{i}$ and $b_{i}$ are the weights and biases of the *i*th layer, respectively. MLPs learn by changing these weights and biases with each new piece of data they see. The magnitude and direction of the changes are based on the difference between the output value and expected result. In order to quantify the degree of error in the output node, a loss function $\mathcal{L}$ is defined,

46 $$ \mathcal{L}(y) = \frac{1}{N}\sum _{i=1}^{N} (\hat{y}_{i} - y_{i})^{2} , $$

where *y* is the expected (i.e., training) value and *ŷ* is the value predicted by the network. This particular loss function is the mean squared error (MSE). Note that the MSE is the loss function used to determine the weights and biases of the network, but the *validation* metric used to evaluate the performance of the trained model is the $r^{2}$-score (see Sect. 3.6). Finding the minimum of the loss function, which is itself a composition of many non-linear functions, is generally impossible analytically. Thus, in order to find the minimum of the loss function, we use a stochastic gradient descent algorithm (Snyman 2005).

The MLPs used in this work consist of an input layer with 12 nodes, one to three hidden layers with up to 24 nodes each, and an output layer with a single node (i.e., a scalar output). All activation functions in the resulting network are the Rectified Linear Unit (ReLU). The ReLU activation function is linear for all positive values, and zero for all negative values, such that $y = \max (0, x)$. For an in-depth introduction to MLPs and the algorithms used here, we direct the reader to the following general comprehensive introduction of neural networks (Goodfellow et al. 2016).

## Footnotes

1 Collisionless N-body codes are capable of simulating orders-of-magnitude larger numbers of particles, having recently reached $2 \times 10^{12}$ particles (Potter et al. 2017).

2 We utilized a smaller dataset for this purpose because generating time series for each post-impact property is computationally expensive and therefore impractical for the larger datasets. However, we still wanted to ensure that we tested the convergence for the full range of collisions.

3 PIM is sometimes referred to as “perfect accretion” or “perfect merging”.

## Abbreviations

AMR: Adaptive Mesh Refinement
ANN: Artificial Neural Network
ARSM: Adaptive Response Surface Method
CART: Classification And Regression Tree
CFD: Computational Fluid Dynamics
CSCS: Swiss National Supercomputing Center
EOS: Equation Of State
ETSS: Effective Training Set Size
EVSS: Effective Validation Set Size
GP: Gaussian Processes
HPO: HyperParameter Optimization
IEM: Impact-Erosion Model
LHS: Latin Hypercube Sample
LR: Largest Remnant
ML: Machine Learning
MLP: Multi-Layer Perceptron
MSE: Mean Squared Error
MTR: Multi-Target Regression
PIM: Perfectly Ineslastic Merging
PCE: Polynomial Chaos Expansion
SLR: Second-Largest Remnant
SMBO: Sequential Model-Based Optimization
SPH: Smoothed-Particle Hydrodynamics
STR: Single-Target Regression
TPE: Tree-structured Parzen Estimator
TSS: Training Set Size
UQ: Uncertainty Quantification
XGB: eXtreme Gradient Boosting
