# Supplementary material for: Machine learning applied to simulations of collisions between rotating, differentiated planets
Source: Comput Astrophys Cosmol. 2020 Dec 2;7(1):2. doi: 10.1186/s40668-020-00034-6 (PMC7716936; doi:10.1186/s40668-020-00034-6)
Supplement: Supplementary file 1 — Supplementary information (ZIP 48.3 MB) [file 40668_2020_34_MOESM1_ESM.zip › residuals_debris_iron_fraction_gp_11884.pdf]

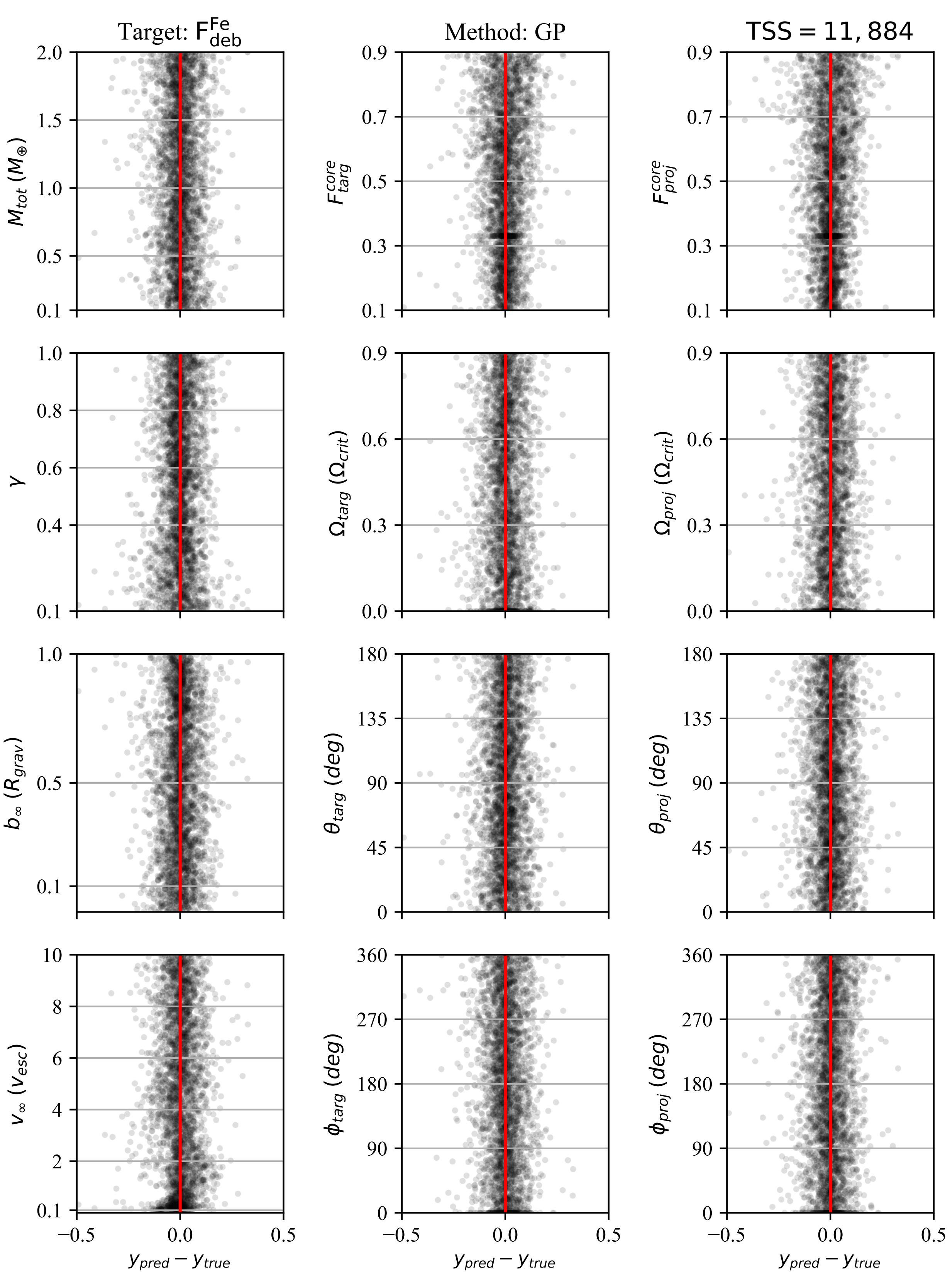
Target: F^{Fe}_{deb}
Method: GP
TSS = 11, 884
M_{tot} ($M_\oplus$)
F^{core}_{targ}
F^{core}_{proj}
γ
Ω_{targ} (Ω_{crit})
Ω_{proj} (Ω_{crit})
b_∞ (R_{grav})
θ_{targ} (deg)
θ_{proj} (deg)
v_∞ (v_{esc})
ϕ_{targ} (deg)
ϕ_{proj} (deg)
$y_{pred} - y_{true}$
$y_{pred} - y_{true}$
$y_{pred} - y_{true}$
