# Supplementary material for: Machine learning applied to simulations of collisions between rotating, differentiated planets
Source: Comput Astrophys Cosmol. 2020 Dec 2;7(1):2. doi: 10.1186/s40668-020-00034-6 (PMC7716936; doi:10.1186/s40668-020-00034-6)
Supplement: Supplementary file 1 — Supplementary information (ZIP 48.3 MB) [file 40668_2020_34_MOESM1_ESM.zip › residuals_debris_iron_fraction_pce_11884.pdf]

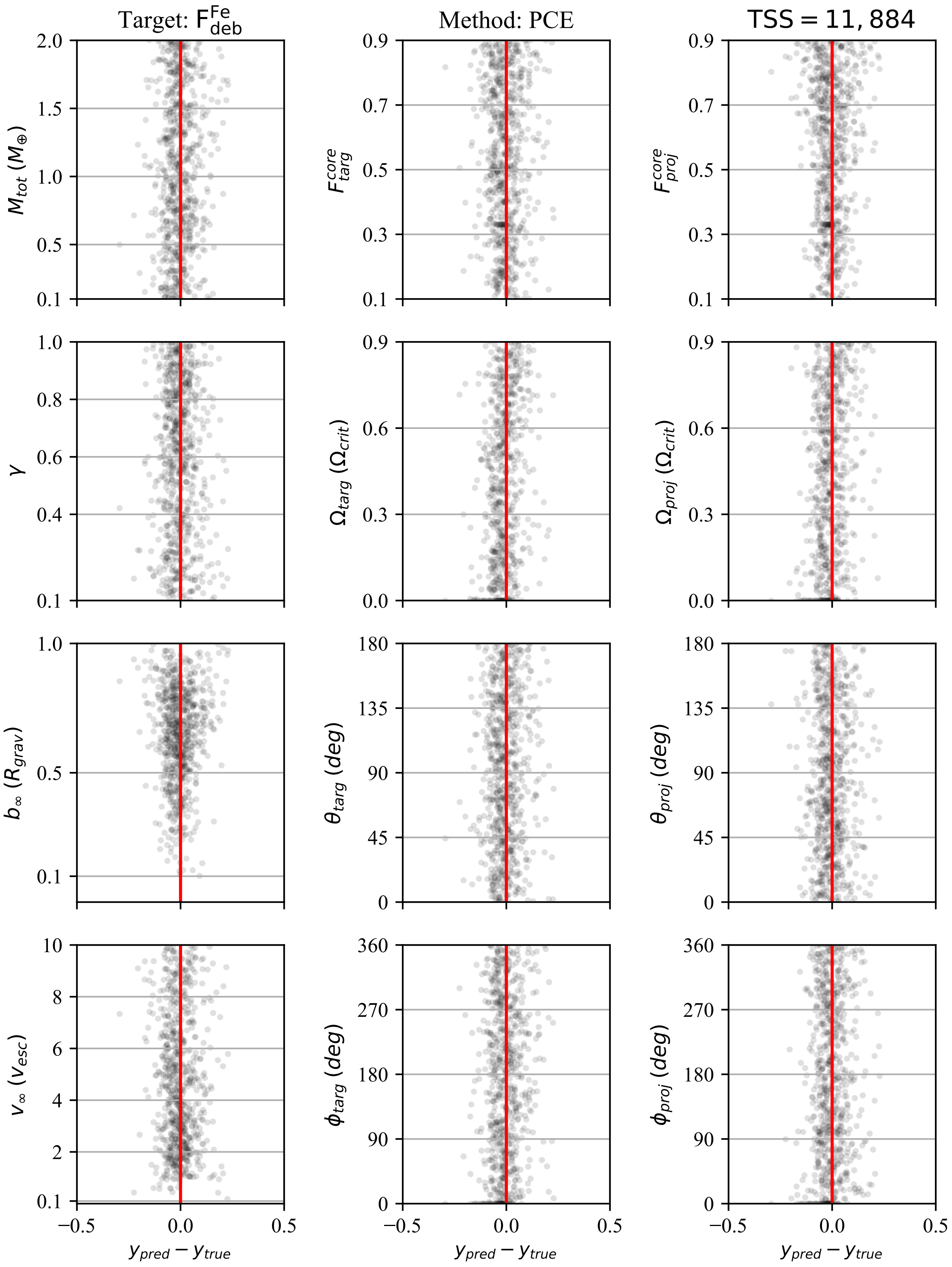

Target: $F^{Fe}_{deb}$
Method: PCE
TSS = 11, 884
$M_{tot}$ ($M_{\oplus}$)
$F^{core}_{targ}$
$F^{core}_{proj}$
$\gamma$
$\Omega_{targ}$ ($\Omega_{crit}$)
$\Omega_{proj}$ ($\Omega_{crit}$)
$b_{\infty}$ ($R_{grav}$)
$\theta_{targ}$ (deg)
$\theta_{proj}$ (deg)
$v_{\infty}$ ($v_{esc}$)
$\phi_{targ}$ (deg)
$\phi_{proj}$ (deg)
$y_{pred} - y_{true}$
