# Supplementary material for: Machine learning applied to simulations of collisions between rotating, differentiated planets
Source: Comput Astrophys Cosmol. 2020 Dec 2;7(1):2. doi: 10.1186/s40668-020-00034-6 (PMC7716936; doi:10.1186/s40668-020-00034-6)
Supplement: Supplementary file 1 — Supplementary information (ZIP 48.3 MB) [file 40668_2020_34_MOESM1_ESM.zip › residuals_debris_mass_norm_pce_11884.pdf]

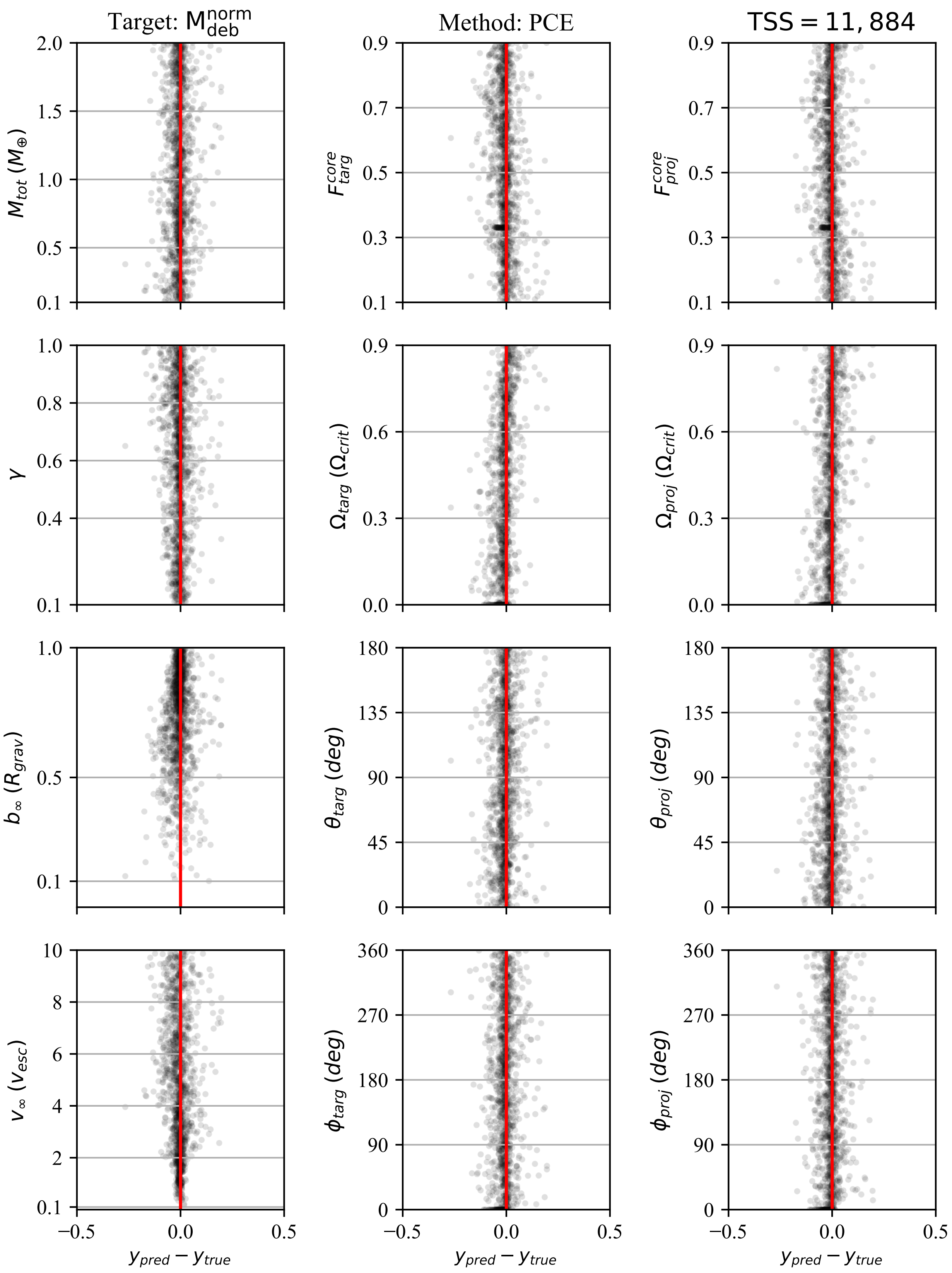

Target: $M_{deb}^{norm}$
Method: PCE
$TSS = 11,884$
$M_{tot}\ (M_\oplus)$
$F_{targ}^{core}$
$F_{proj}^{core}$
$\gamma$
$\Omega_{targ}\ (\Omega_{crit})$
$\Omega_{proj}\ (\Omega_{crit})$
$b_\infty\ (R_{grav})$
$\theta_{targ}\ (deg)$
$\theta_{proj}\ (deg)$
$v_\infty\ (v_{esc})$
$\phi_{targ}\ (deg)$
$\phi_{proj}\ (deg)$
$y_{pred} - y_{true}$
