# Supplementary material for: Machine learning applied to simulations of collisions between rotating, differentiated planets
Source: Comput Astrophys Cosmol. 2020 Dec 2;7(1):2. doi: 10.1186/s40668-020-00034-6 (PMC7716936; doi:10.1186/s40668-020-00034-6)
Supplement: Supplementary file 1 — Supplementary information (ZIP 48.3 MB) [file 40668_2020_34_MOESM1_ESM.zip › residuals_slr_core_fraction_gp_11884.pdf]

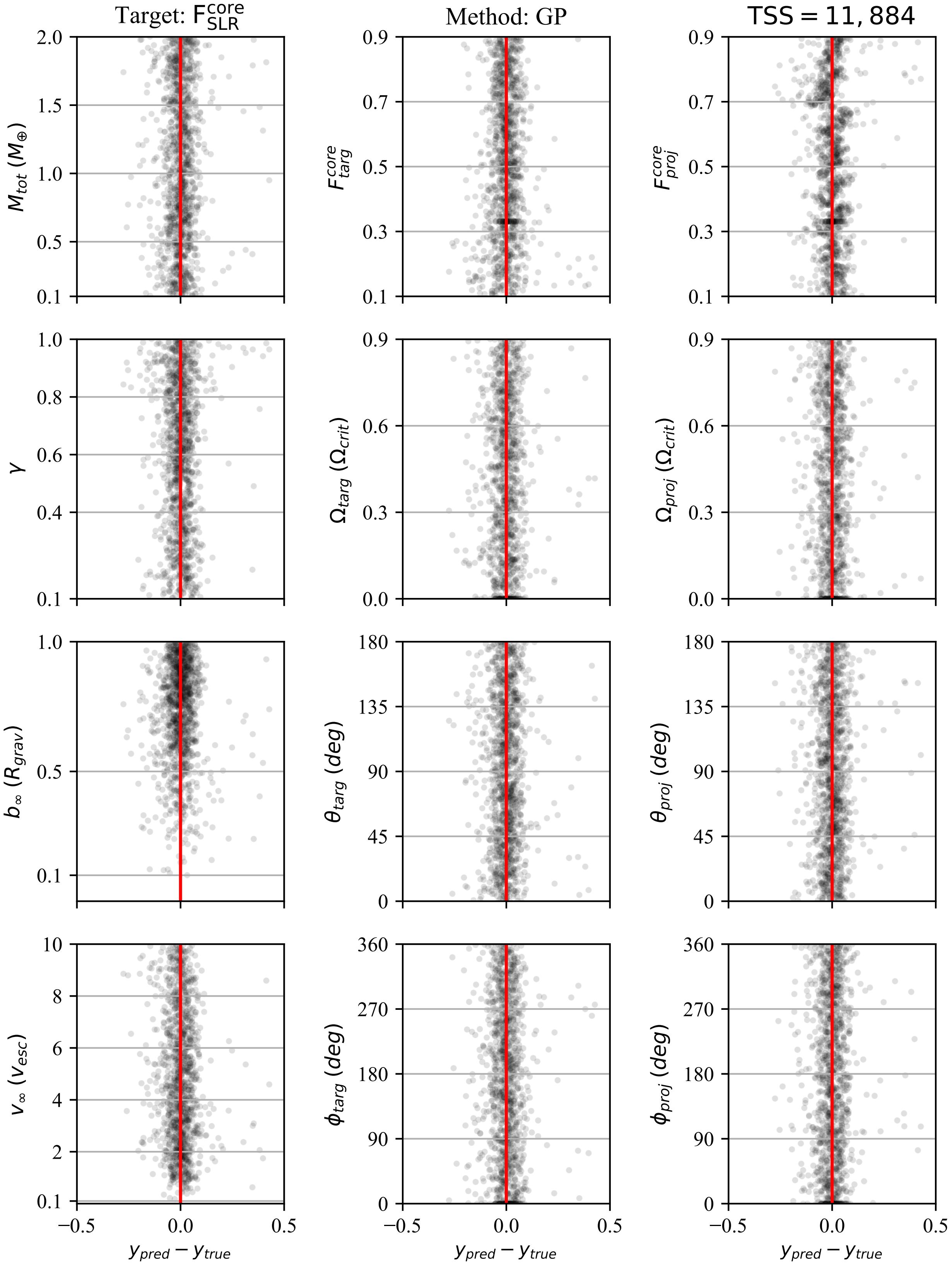

Target: F_{SLR}^{core}
Method: GP
$TSS = 11,884$
$M_{tot}\ (M_{\oplus})$
F_{targ}^{core}
F_{proj}^{core}
γ
$\Omega_{targ}\ (\Omega_{crit})$
$\Omega_{proj}\ (\Omega_{crit})$
$b_{\infty}\ (R_{grav})$
$\theta_{targ}\ (deg)$
$\theta_{proj}\ (deg)$
$v_{\infty}\ (v_{esc})$
$\phi_{targ}\ (deg)$
$\phi_{proj}\ (deg)$
$y_{pred} - y_{true}$
