# Supplementary figures and images for: Machine learning applied to simulations of collisions between rotating, differentiated planets
Source: Comput Astrophys Cosmol. 2020 Dec 2;7(1):2. doi: 10.1186/s40668-020-00034-6 (PMC7716936; doi:10.1186/s40668-020-00034-6)

Target:  $F_{\text{deb}}^{\text{Fe}}$ 

Method: XGB

TSS = 11,884

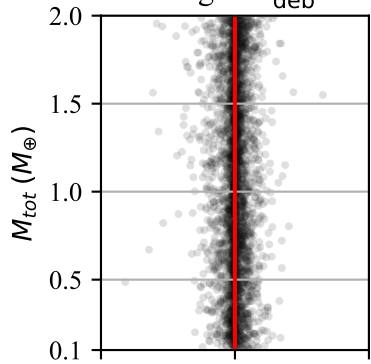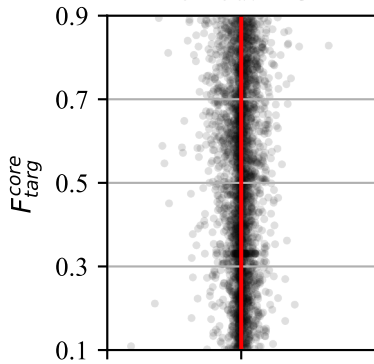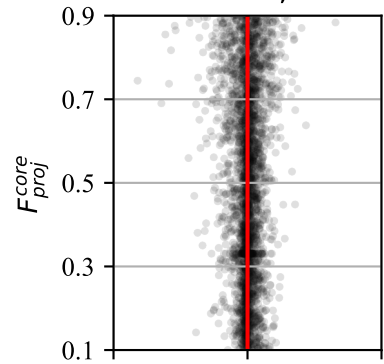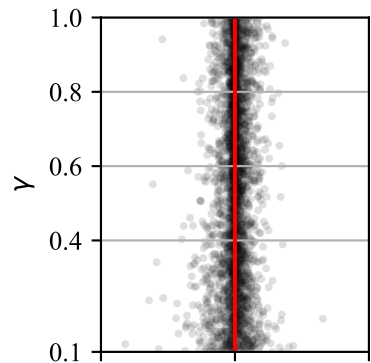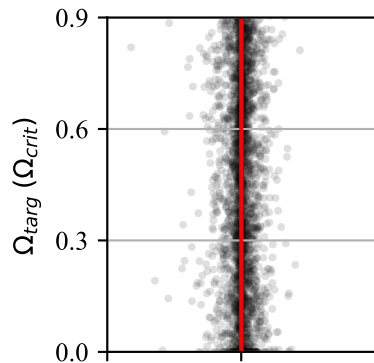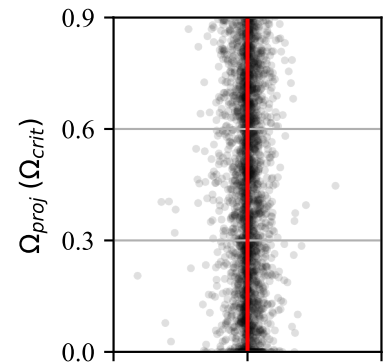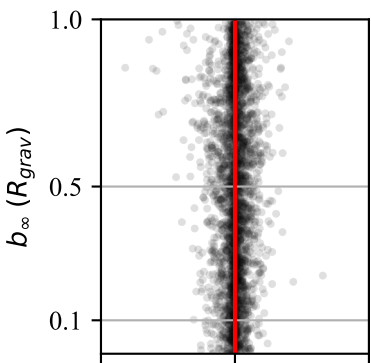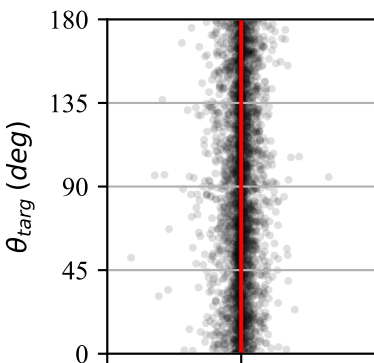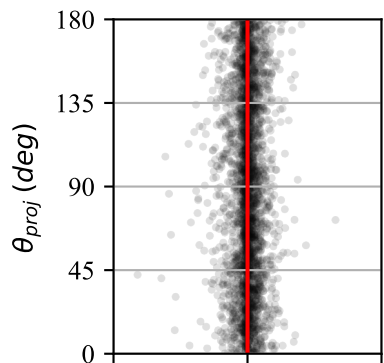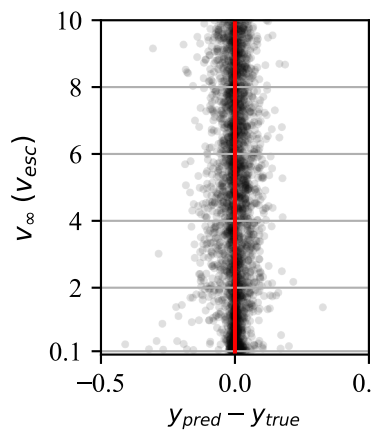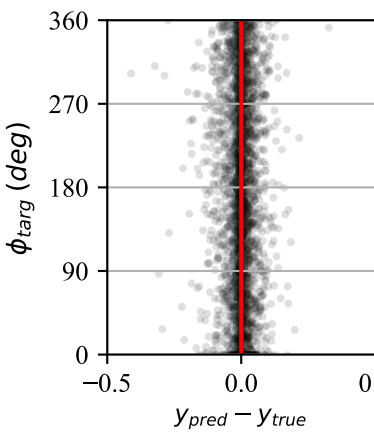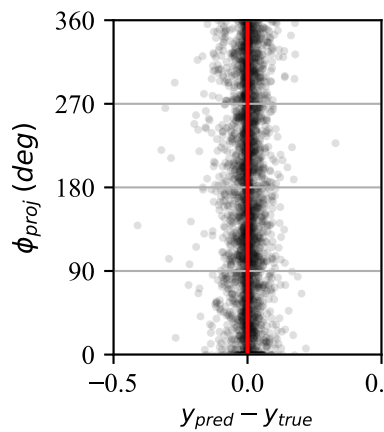

Supplement: Supplementary file 1 — Supplementary information (ZIP 48.3 MB) [file 40668_2020_34_MOESM1_ESM.zip › residuals_debris_iron_fraction_xgb_11884.pdf]

Target:  $M_{\text{deb}}$ 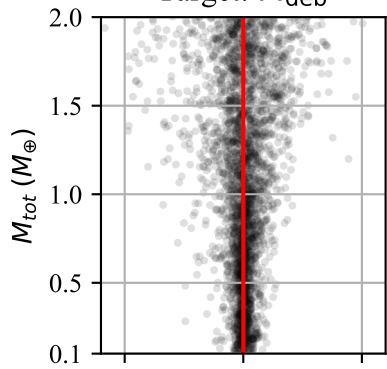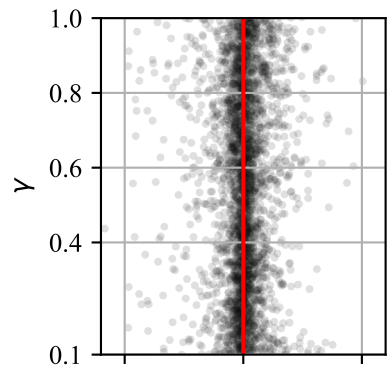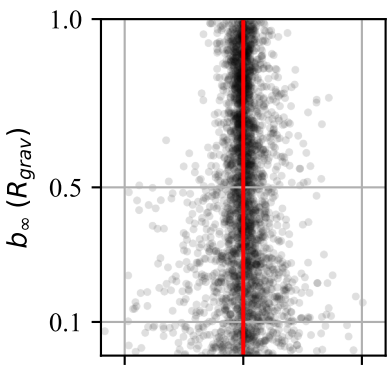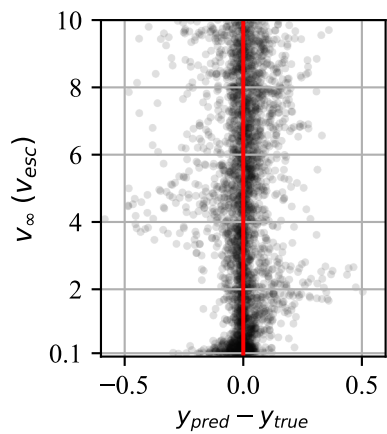

Method: GP

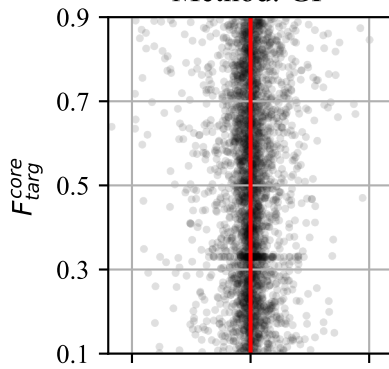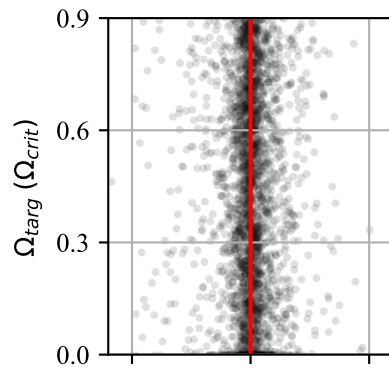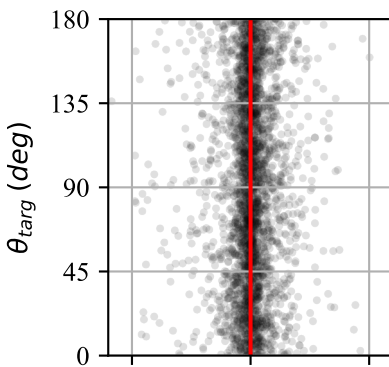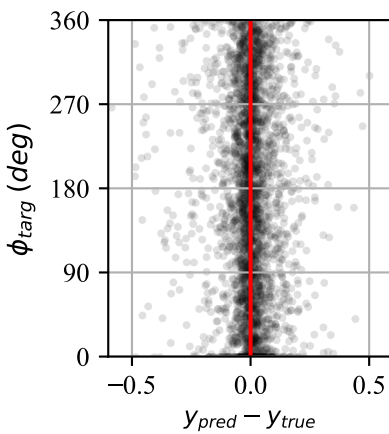

TSS = 11,884

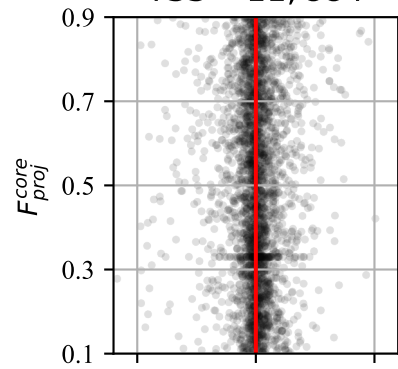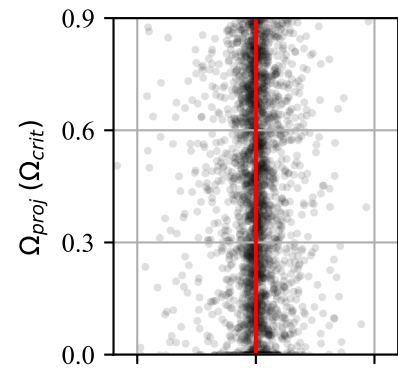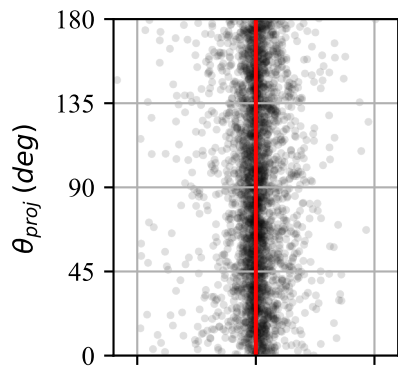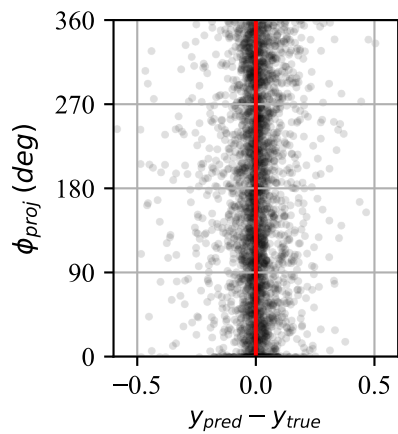

Supplement: Supplementary file 1 — Supplementary information (ZIP 48.3 MB) [file 40668_2020_34_MOESM1_ESM.zip › residuals_debris_mass_gp_11884.pdf]

Target:  $M_{\text{deb}}$ 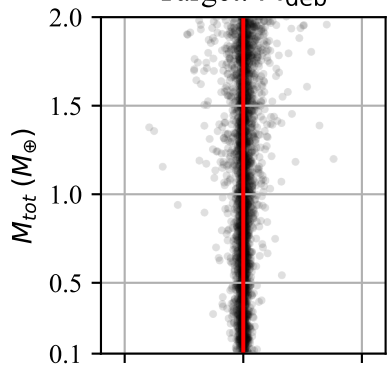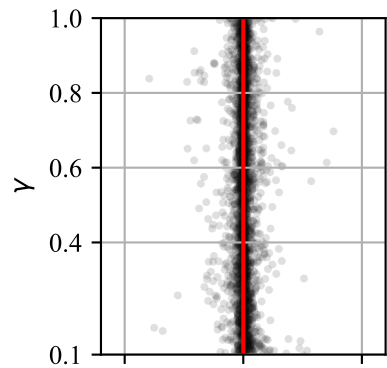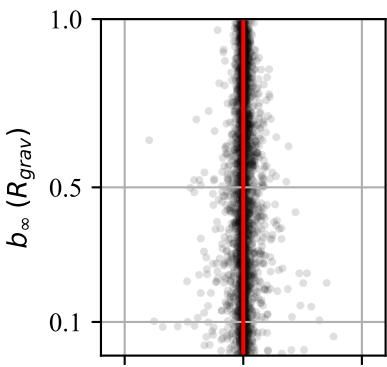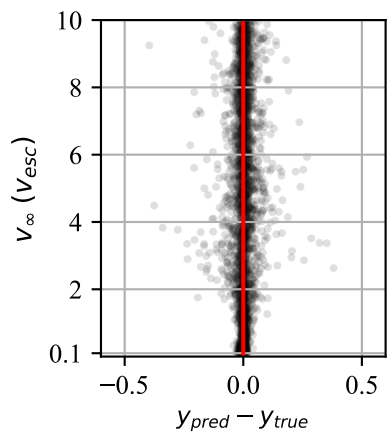

Method: MLP

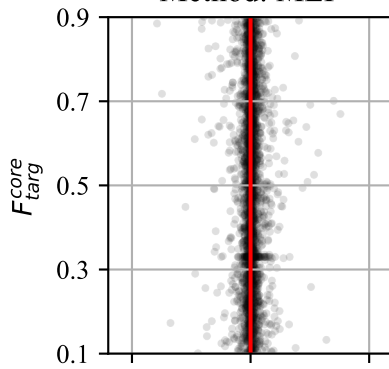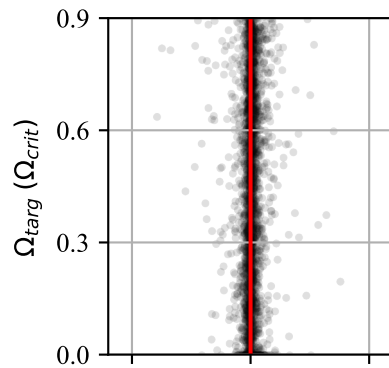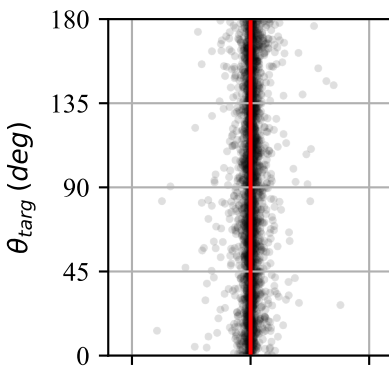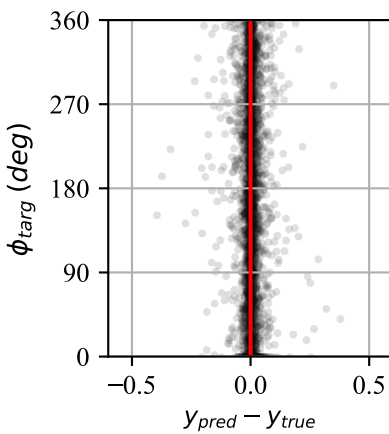

TSS = 11,884

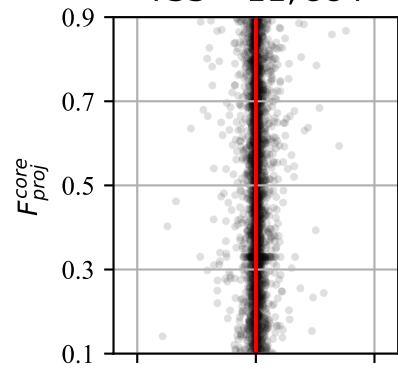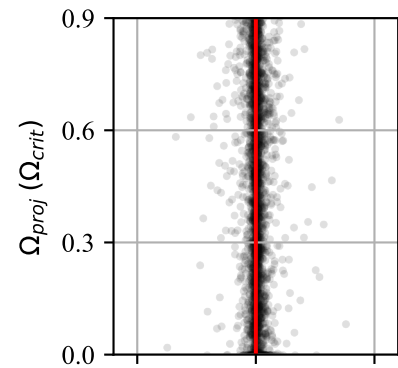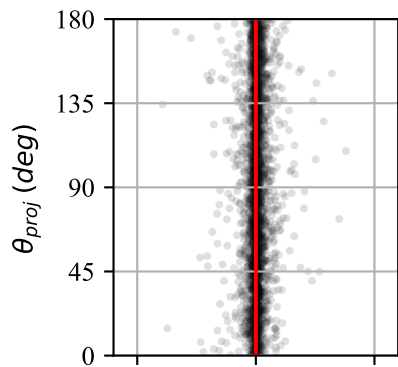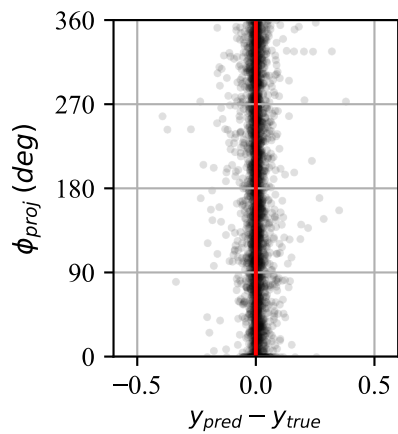

Supplement: Supplementary file 1 — Supplementary information (ZIP 48.3 MB) [file 40668_2020_34_MOESM1_ESM.zip › residuals_debris_mass_mlp_11884.pdf]

Target:  $M_{\text{deb}}^{\text{norm}}$ 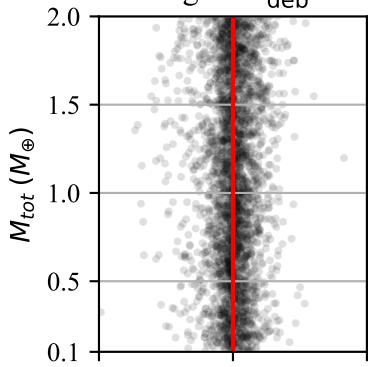

Method: GP

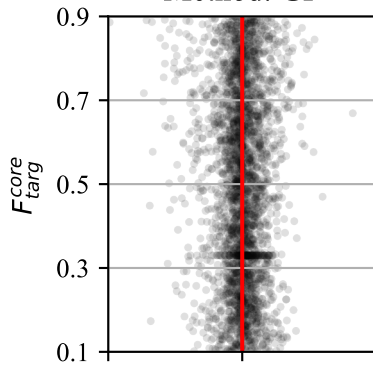

TSS = 11, 884

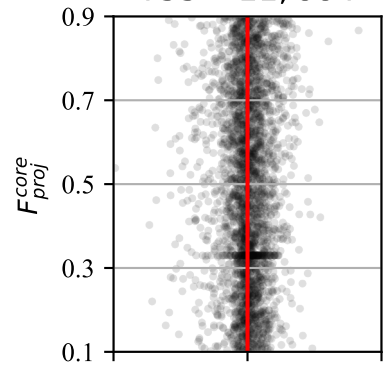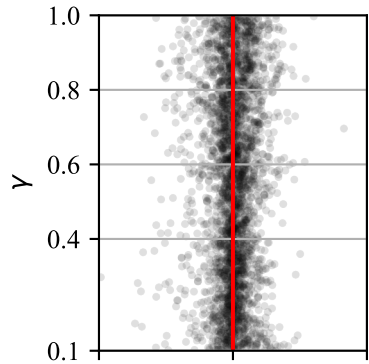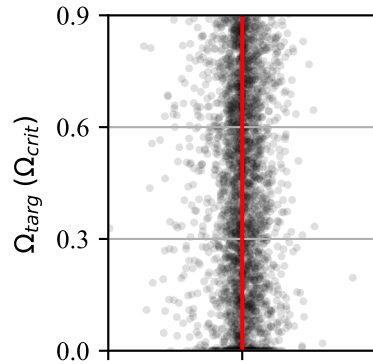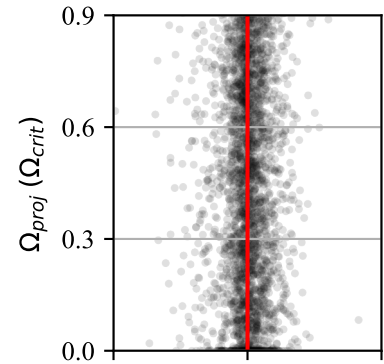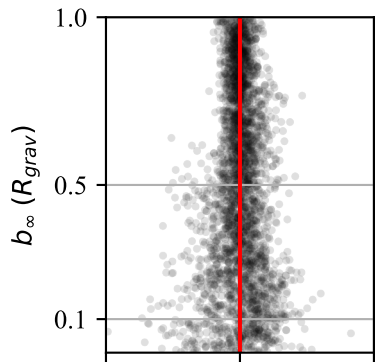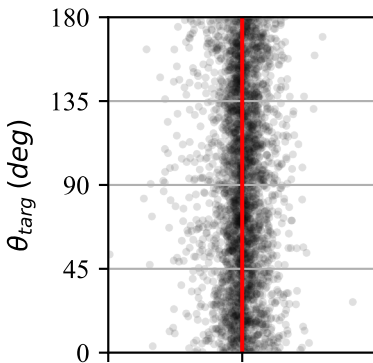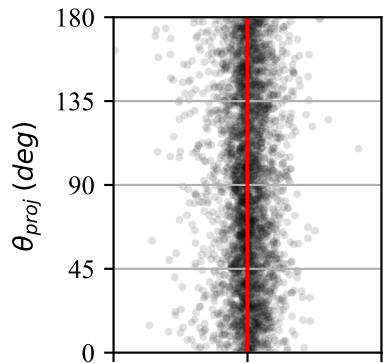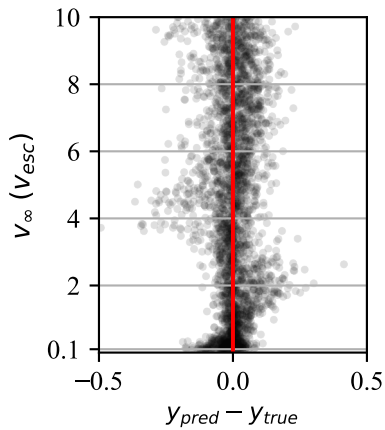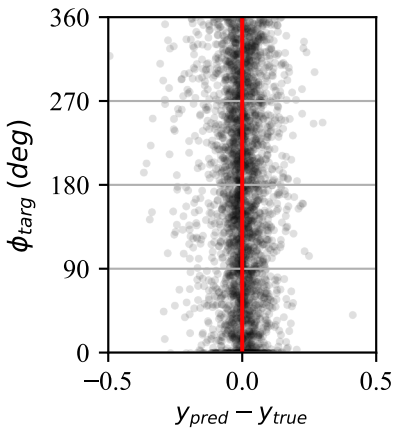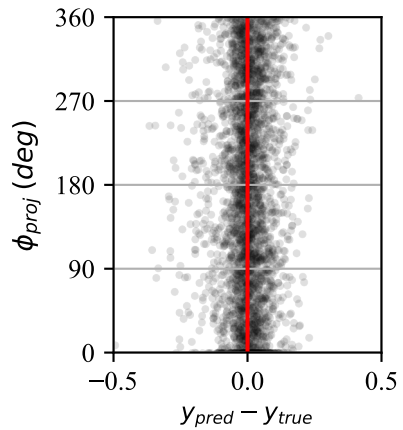

Supplement: Supplementary file 1 — Supplementary information (ZIP 48.3 MB) [file 40668_2020_34_MOESM1_ESM.zip › residuals_debris_mass_norm_gp_11884.pdf]

Target:  $M_{\text{deb}}$ 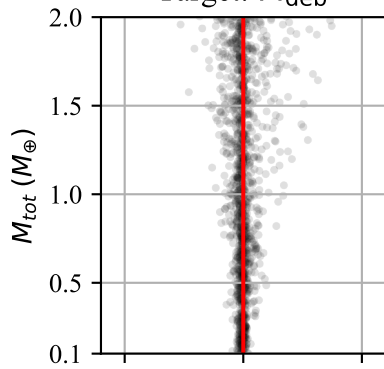

Method: PCE

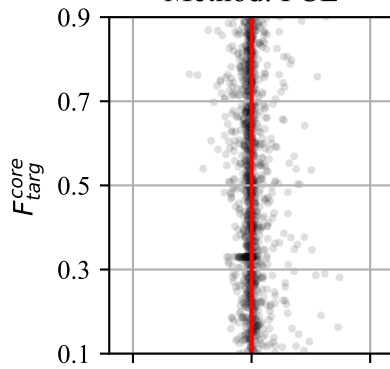

TSS = 11,884

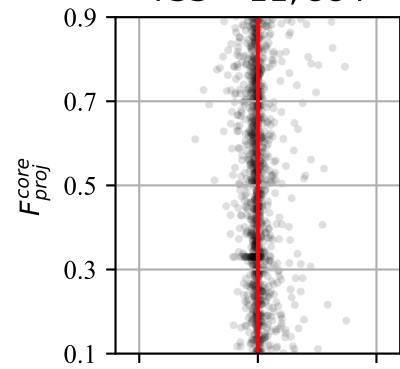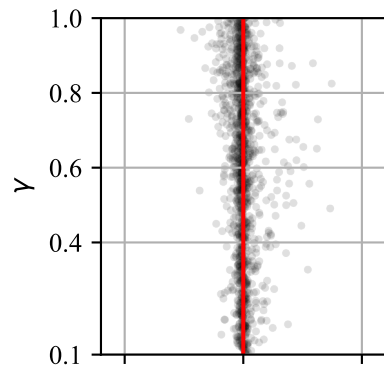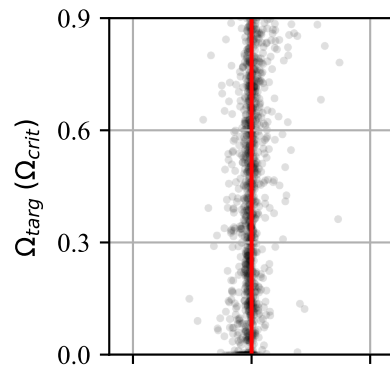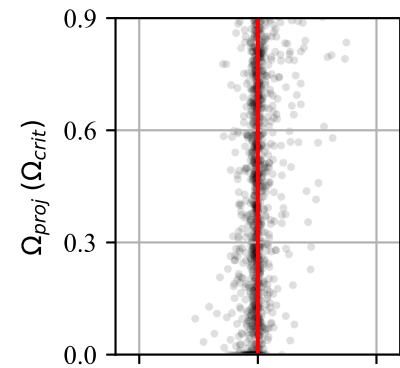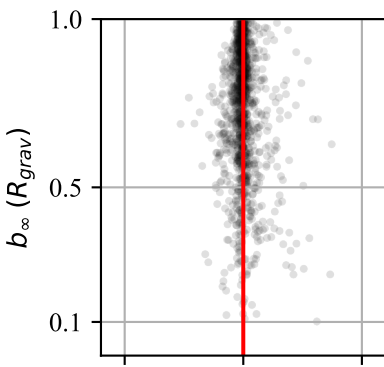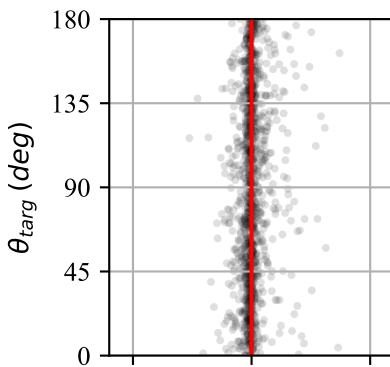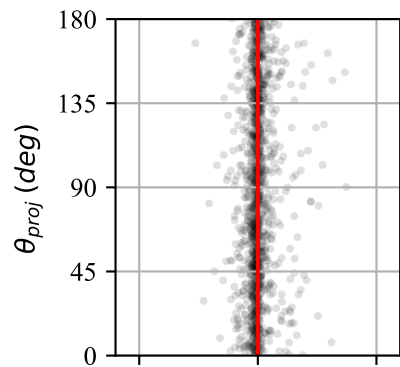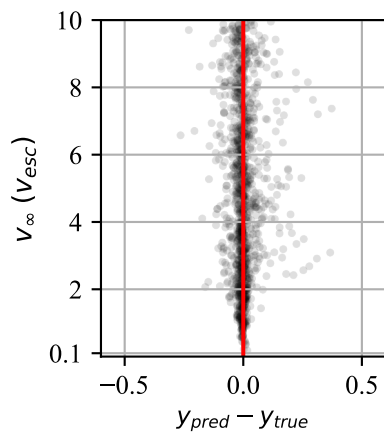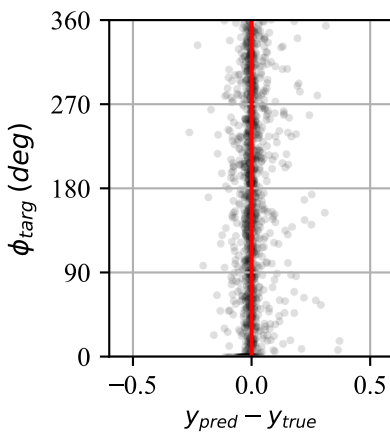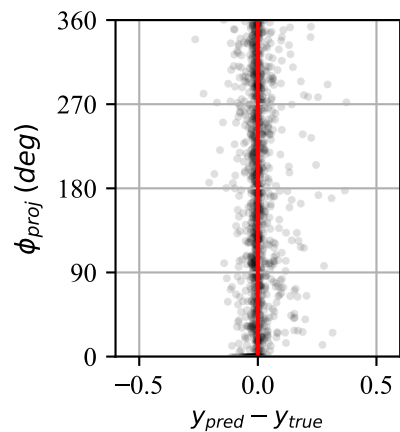

Supplement: Supplementary file 1 — Supplementary information (ZIP 48.3 MB) [file 40668_2020_34_MOESM1_ESM.zip › residuals_debris_mass_pce_11884.pdf]

Target:  $M_{\text{deb}}$ 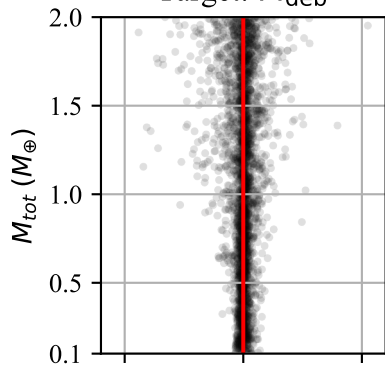

Method: XGB

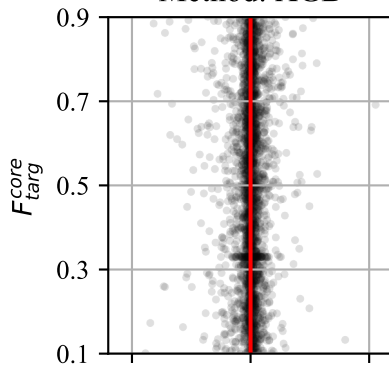

TSS = 11,884

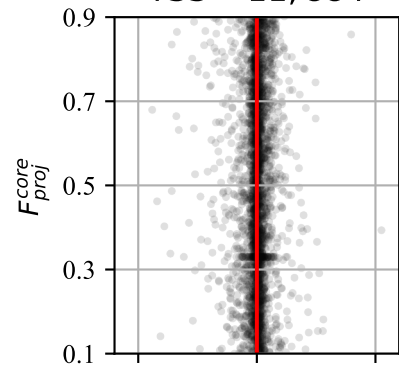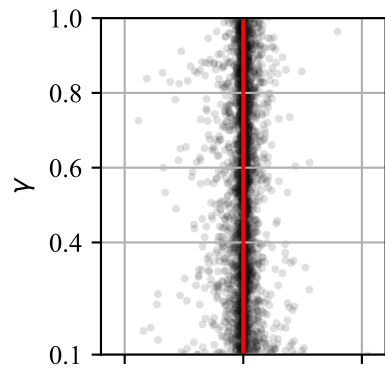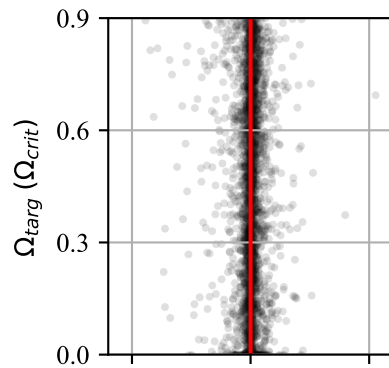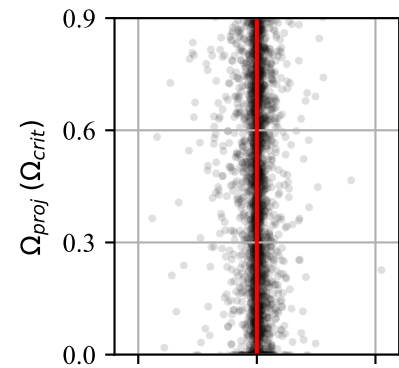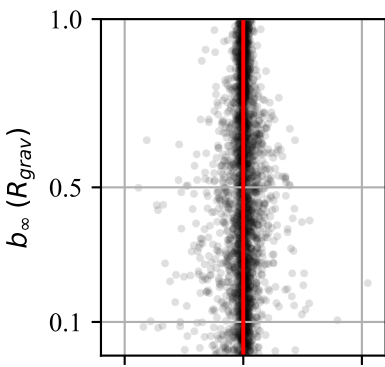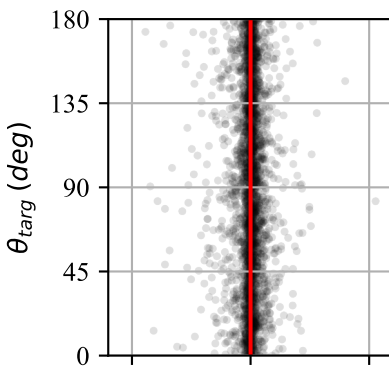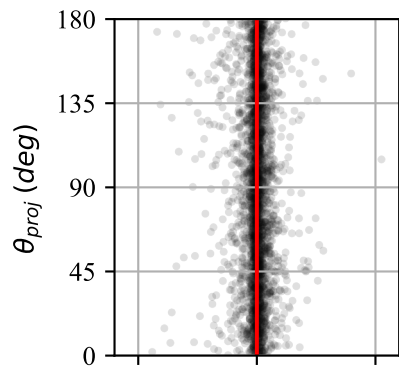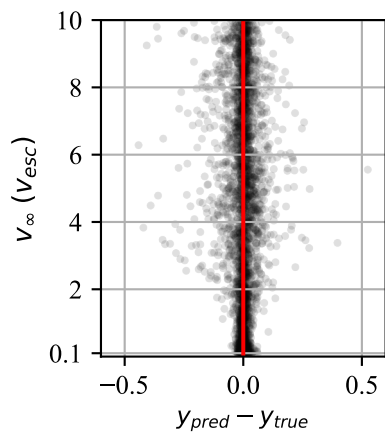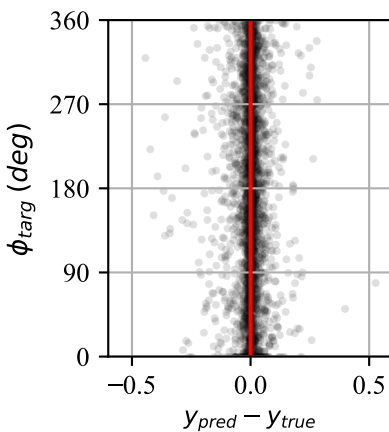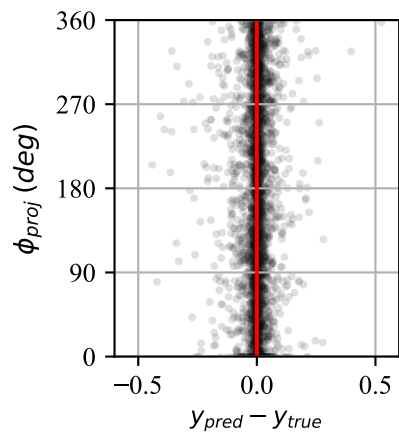

Supplement: Supplementary file 1 — Supplementary information (ZIP 48.3 MB) [file 40668_2020_34_MOESM1_ESM.zip › residuals_debris_mass_xgb_11884.pdf]

Target:  $\bar{\theta}_{\text{deb}}$ 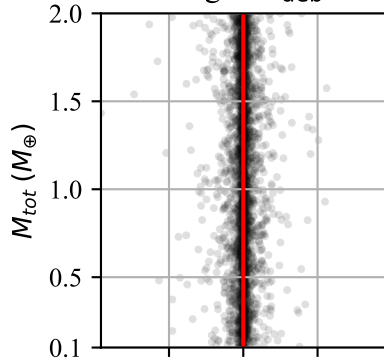

Method: GP

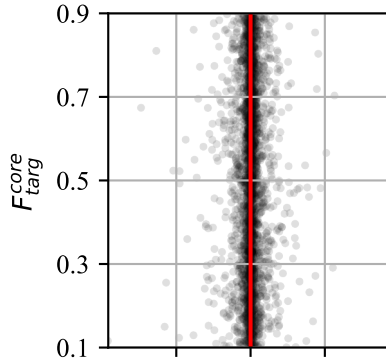

TSS = 11,884

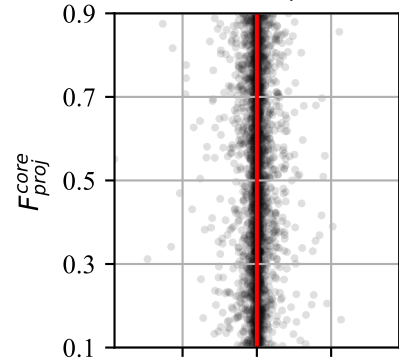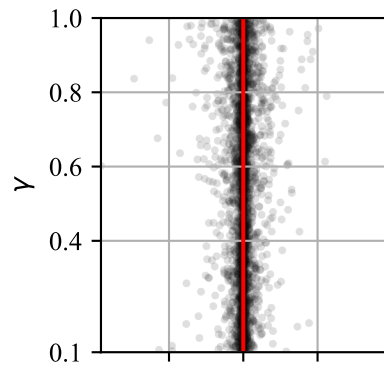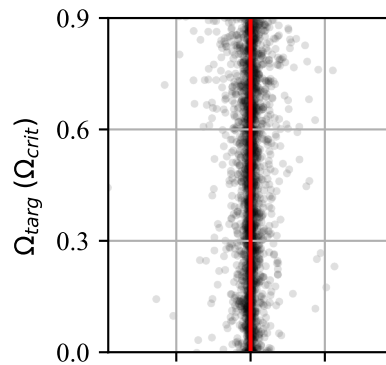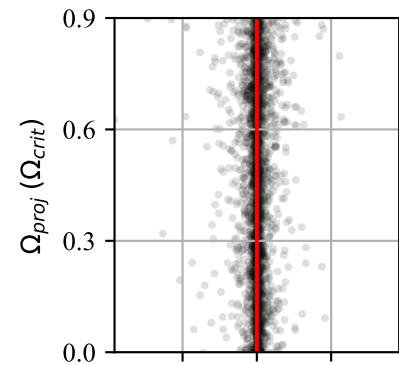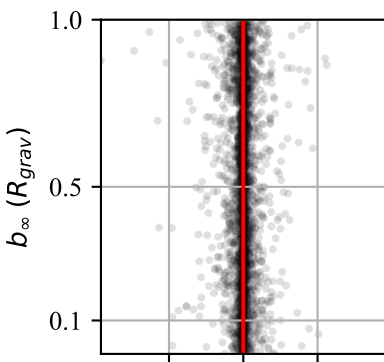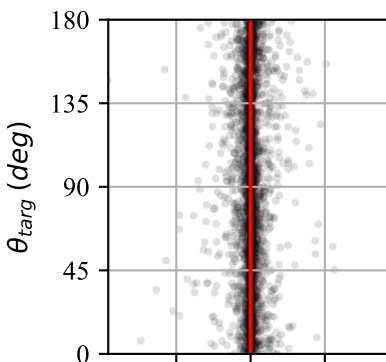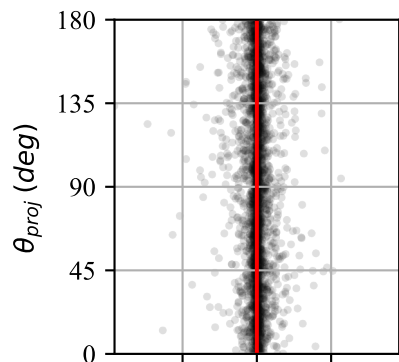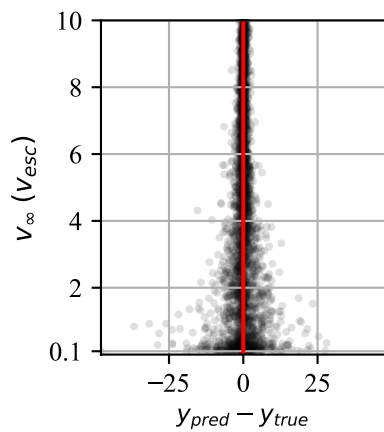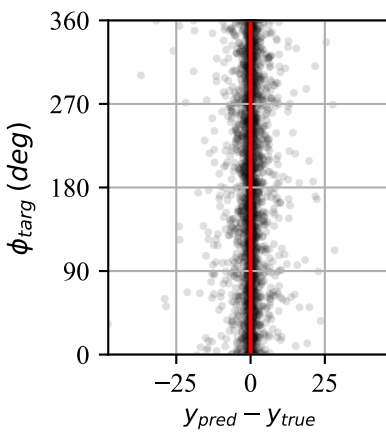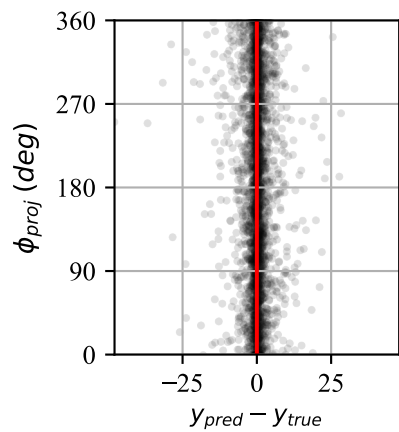

Supplement: Supplementary file 1 — Supplementary information (ZIP 48.3 MB) [file 40668_2020_34_MOESM1_ESM.zip › residuals_debris_mean_altitude_gp_11884.pdf]

Target:  $\bar{\theta}_{\text{deb}}$ 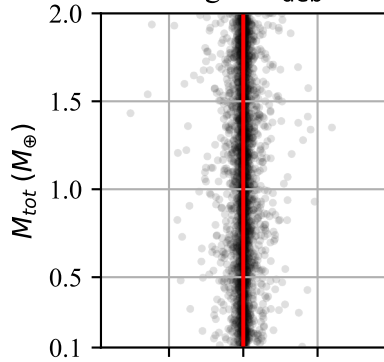

Method: MLP

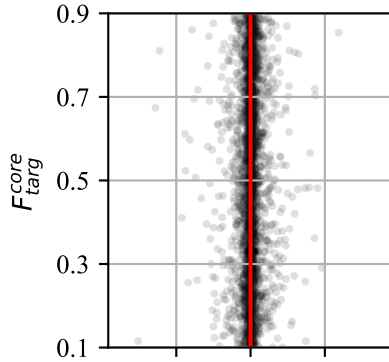

TSS = 11,884

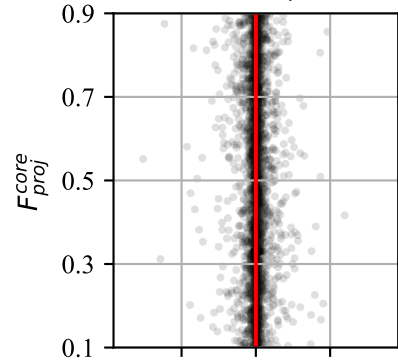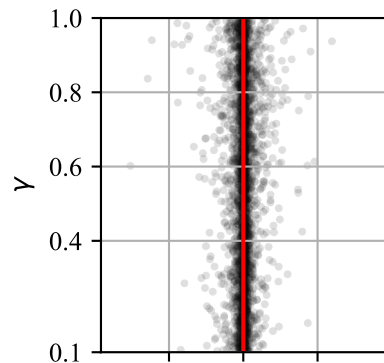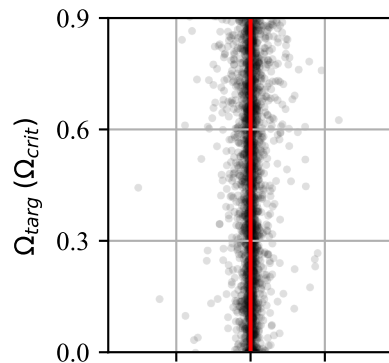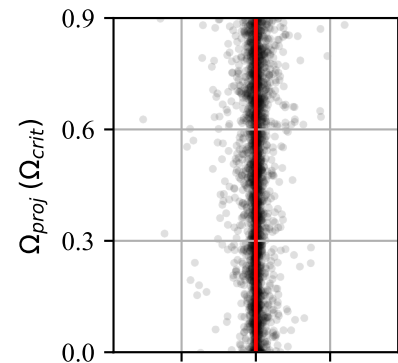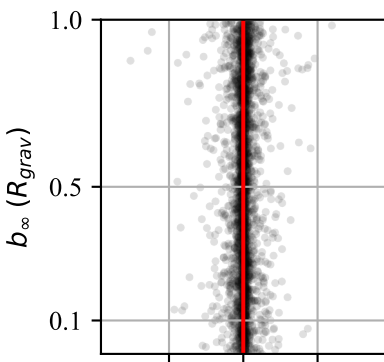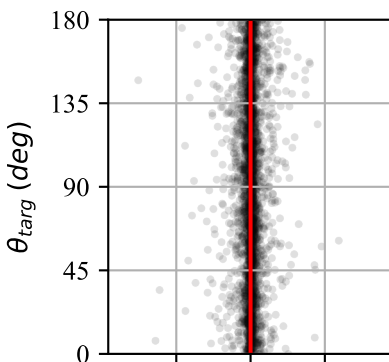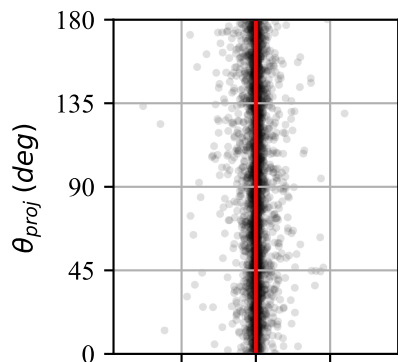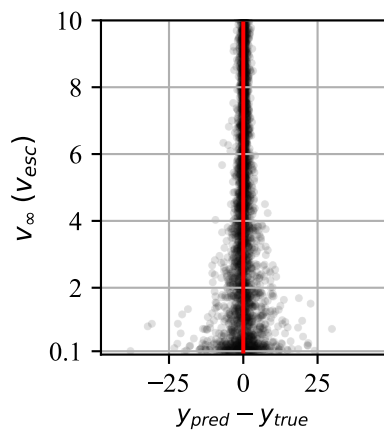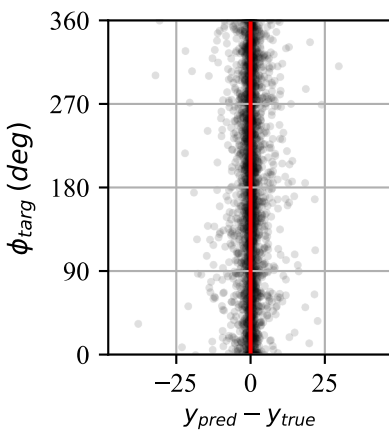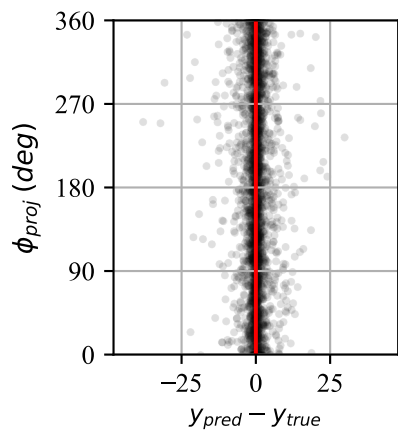

Supplement: Supplementary file 1 — Supplementary information (ZIP 48.3 MB) [file 40668_2020_34_MOESM1_ESM.zip › residuals_debris_mean_altitude_mlp_11884.pdf]

Target:  $\bar{\theta}_{\text{deb}}$ 

Method: XGB

TSS = 11,884

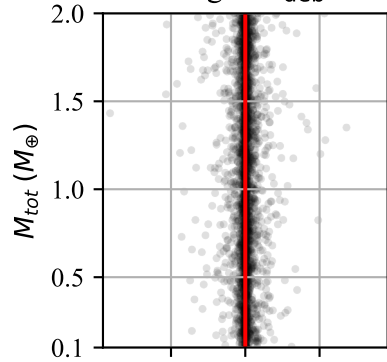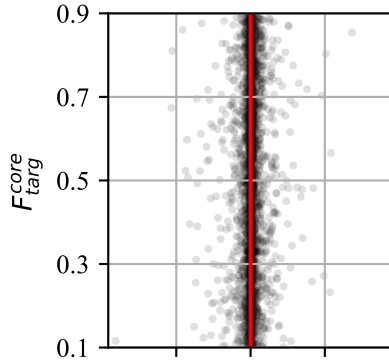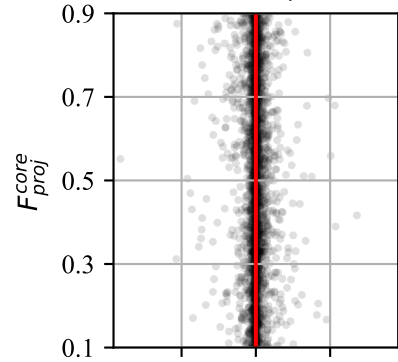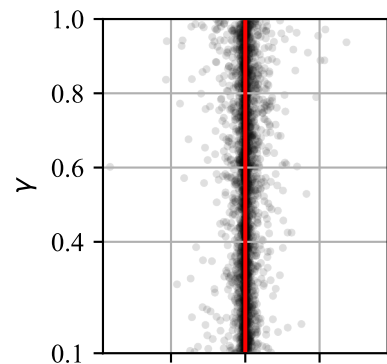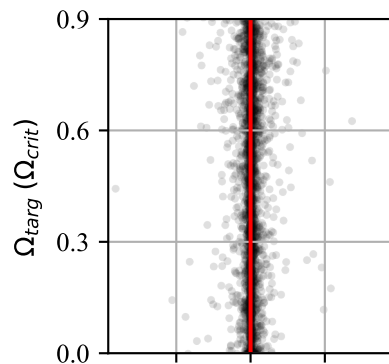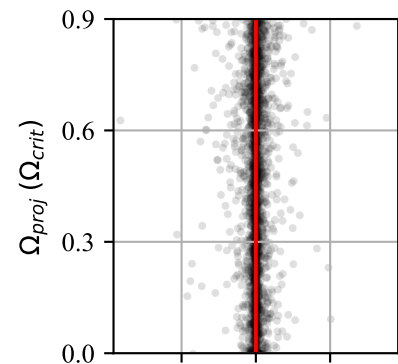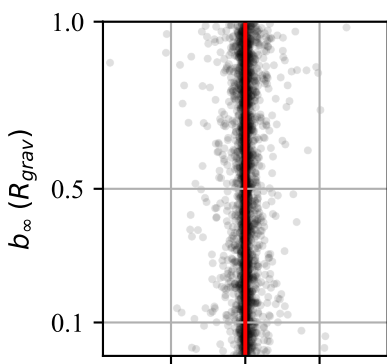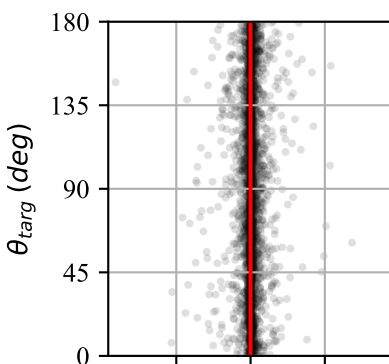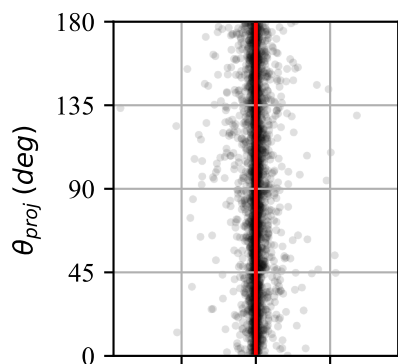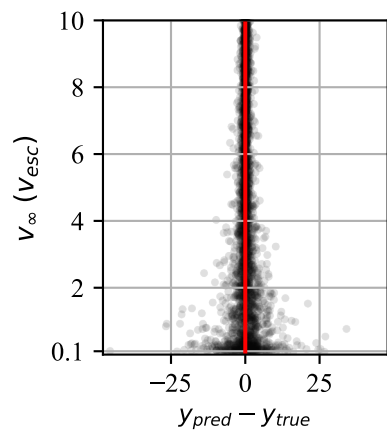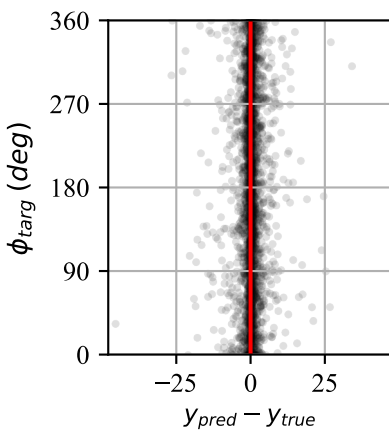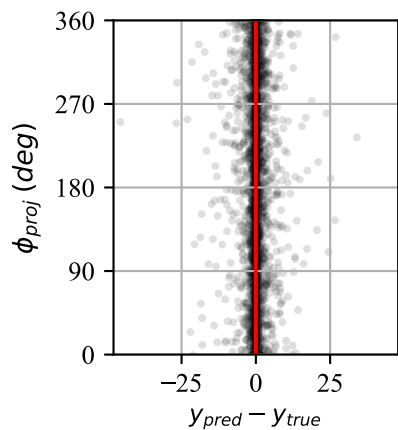

Supplement: Supplementary file 1 — Supplementary information (ZIP 48.3 MB) [file 40668_2020_34_MOESM1_ESM.zip › residuals_debris_mean_altitude_xgb_11884.pdf]

Target:  $\bar{\phi}_{\text{deb}}$ 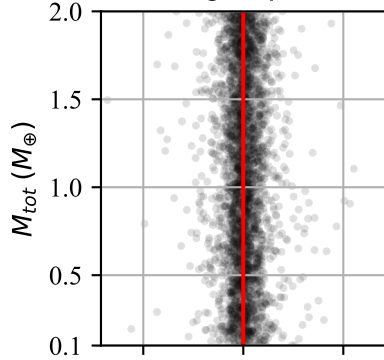

Method: GP

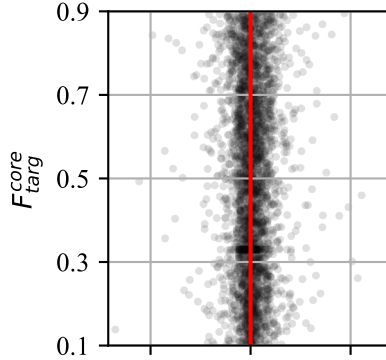

TSS = 11,884

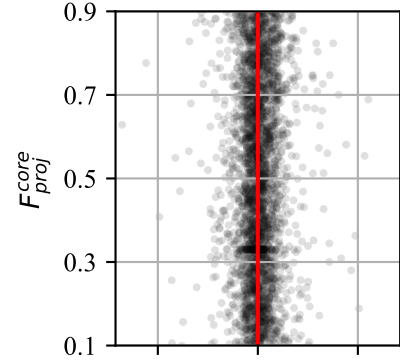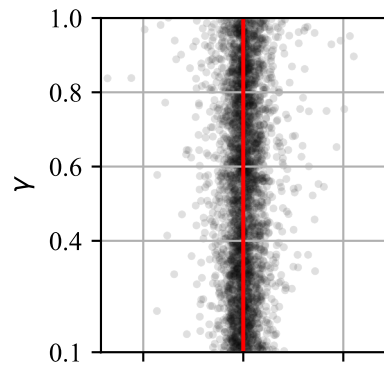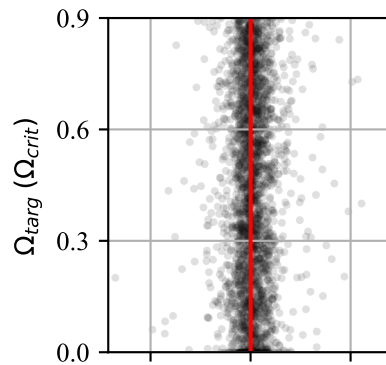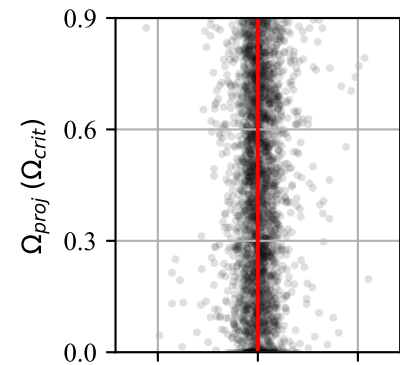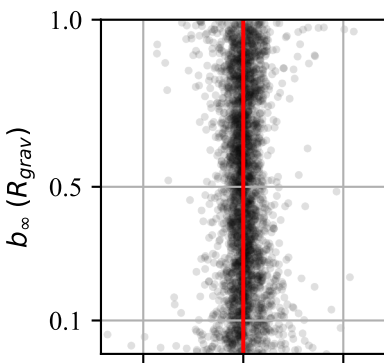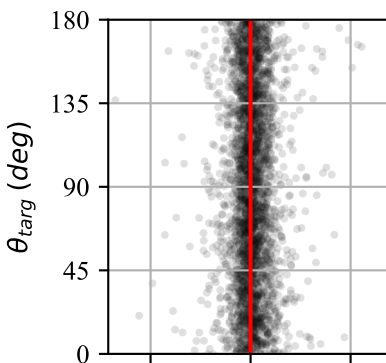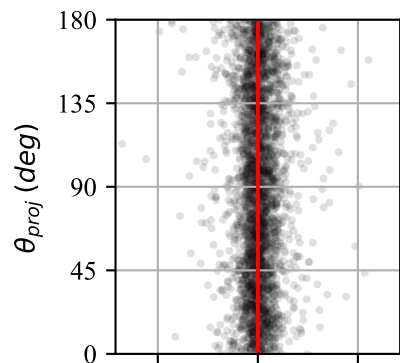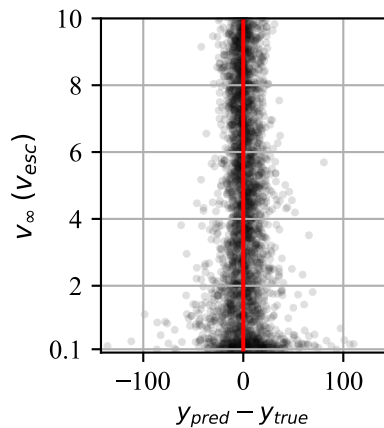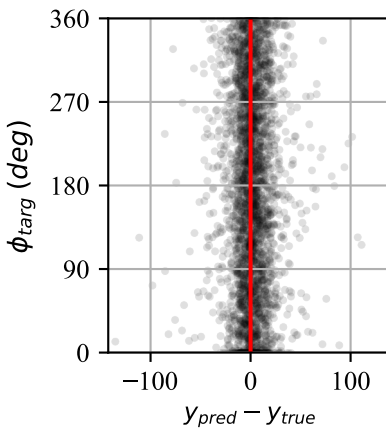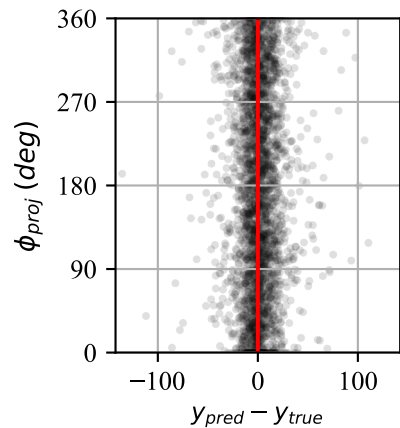

Supplement: Supplementary file 1 — Supplementary information (ZIP 48.3 MB) [file 40668_2020_34_MOESM1_ESM.zip › residuals_debris_mean_azimuth_gp_11884.pdf]

Target:  $\bar{\phi}_{\text{deb}}$ 

Method: MLP

TSS = 11,884

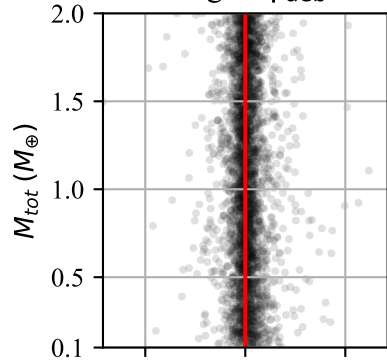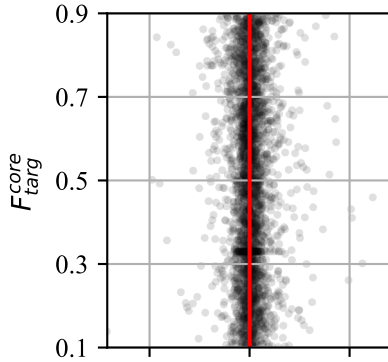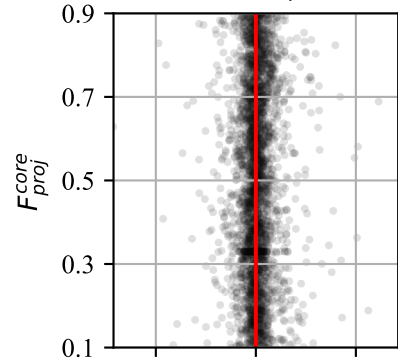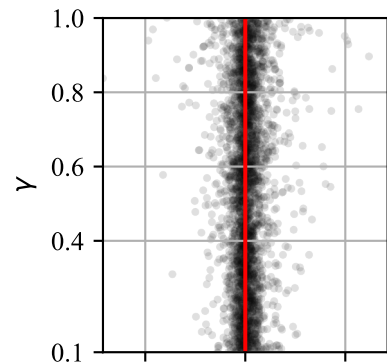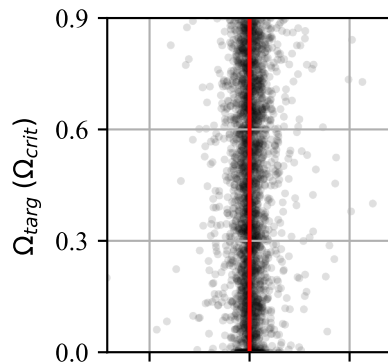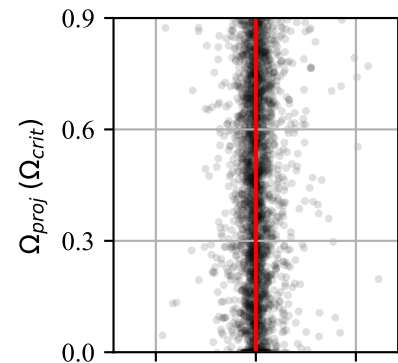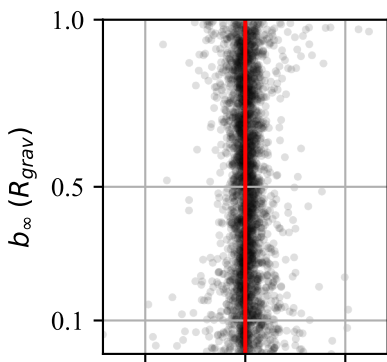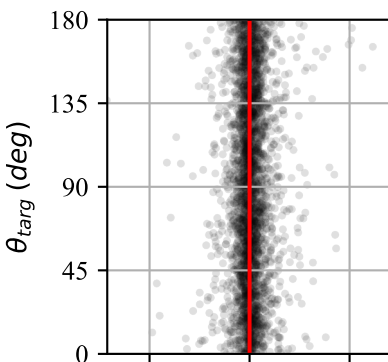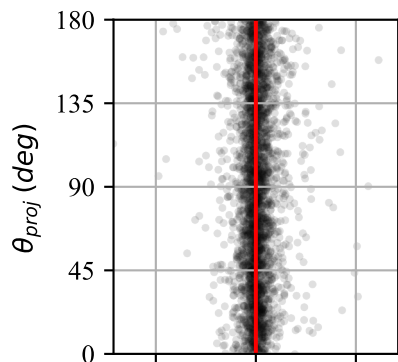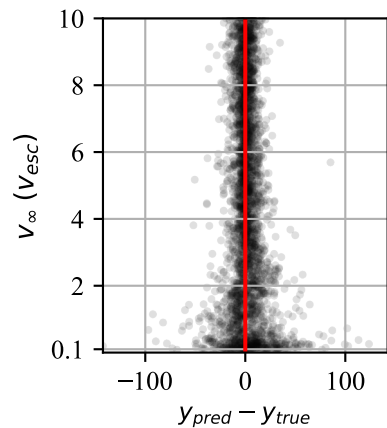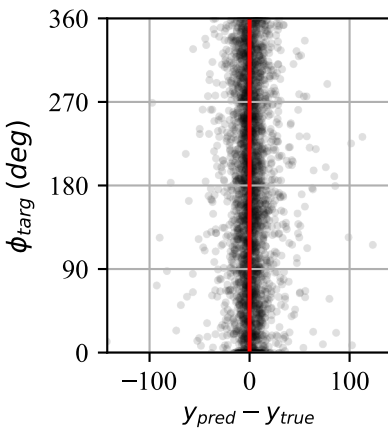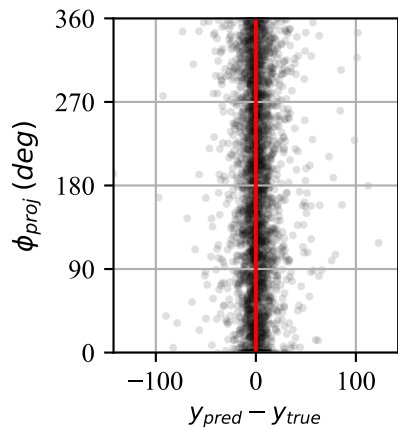

Supplement: Supplementary file 1 — Supplementary information (ZIP 48.3 MB) [file 40668_2020_34_MOESM1_ESM.zip › residuals_debris_mean_azimuth_mlp_11884.pdf]

Target:  $\bar{\phi}_{\text{deb}}$ 

Method: XGB

TSS = 11,884

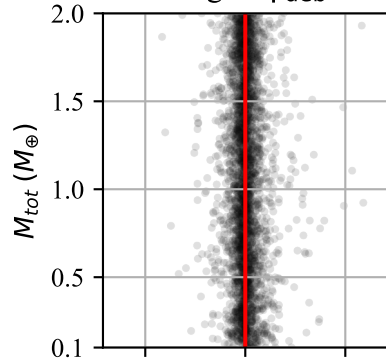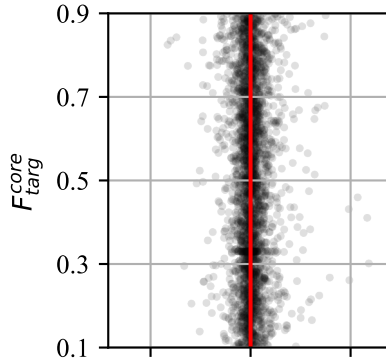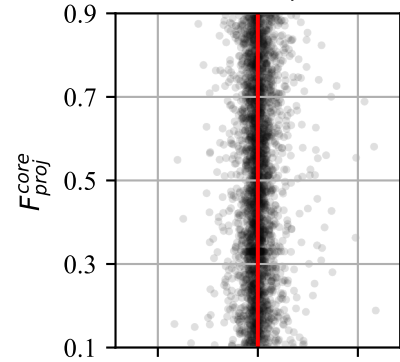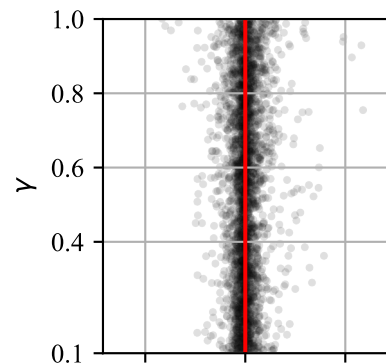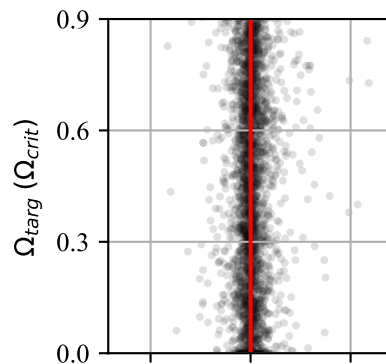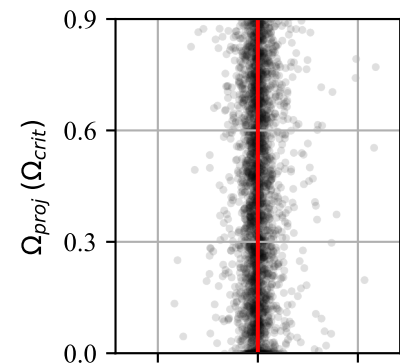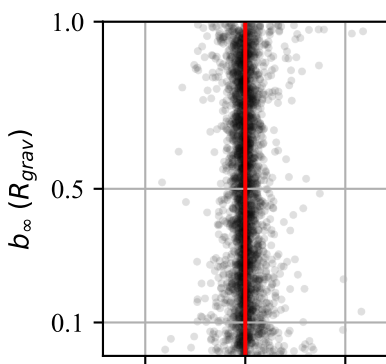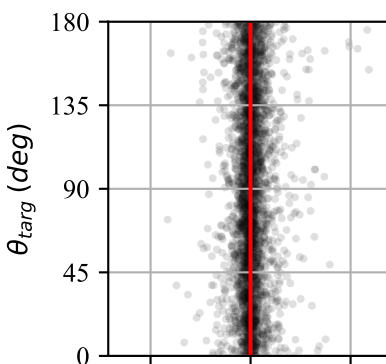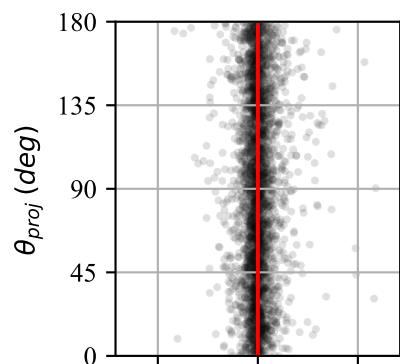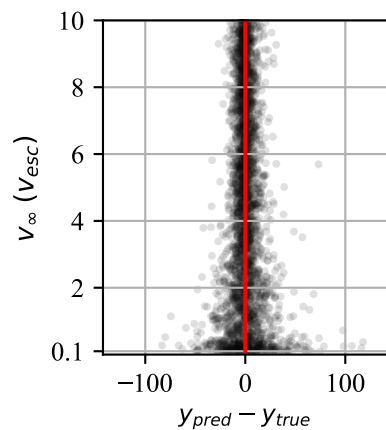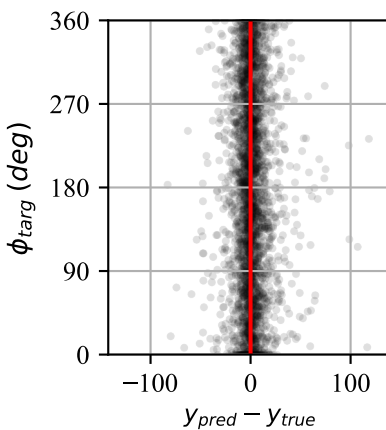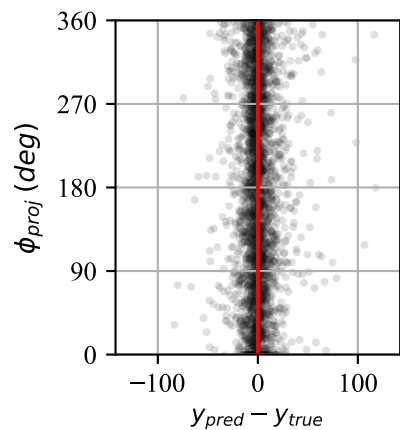

Supplement: Supplementary file 1 — Supplementary information (ZIP 48.3 MB) [file 40668_2020_34_MOESM1_ESM.zip › residuals_debris_mean_azimuth_xgb_11884.pdf]

Target:  $\delta_{\text{deb}}^{\text{mix}}$ 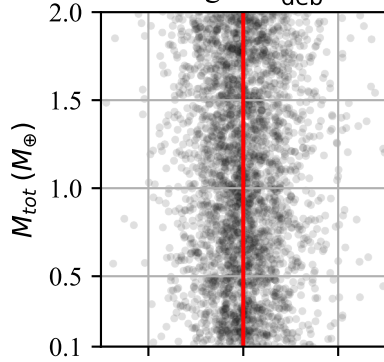

Method: GP

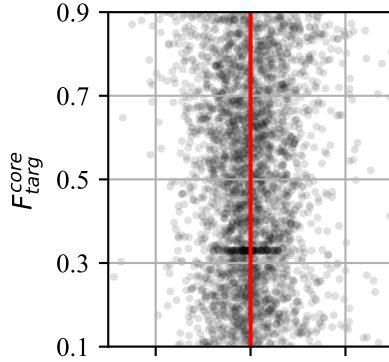

TSS = 11,884

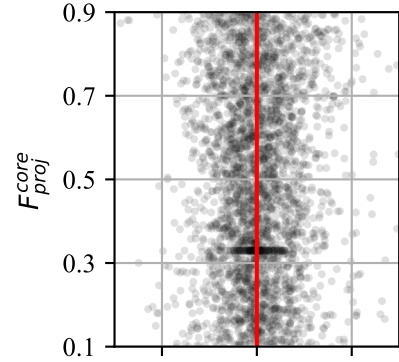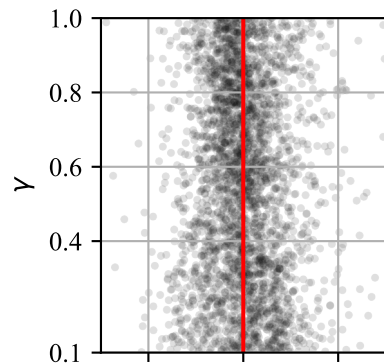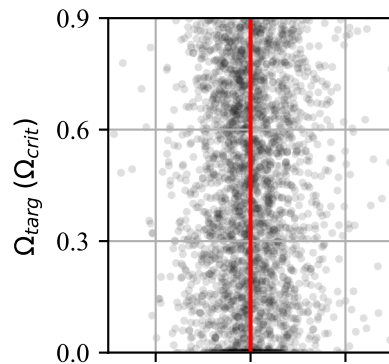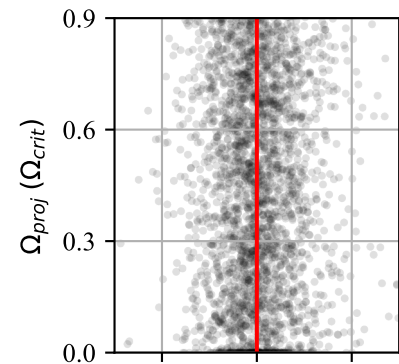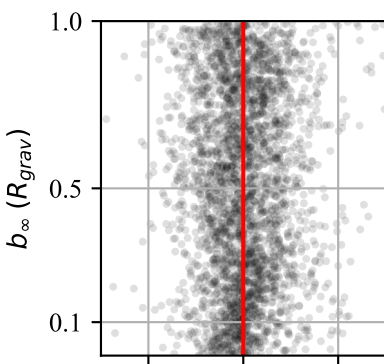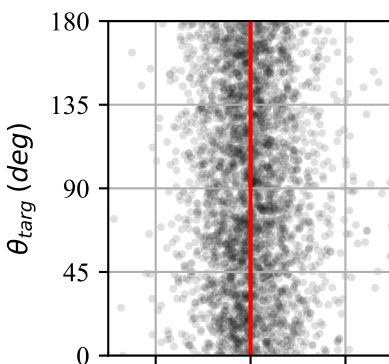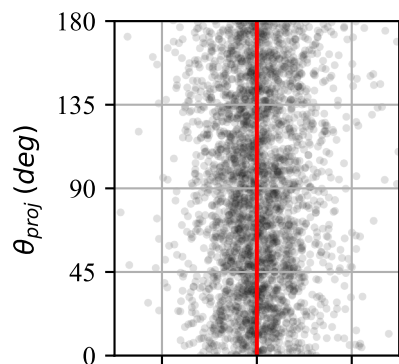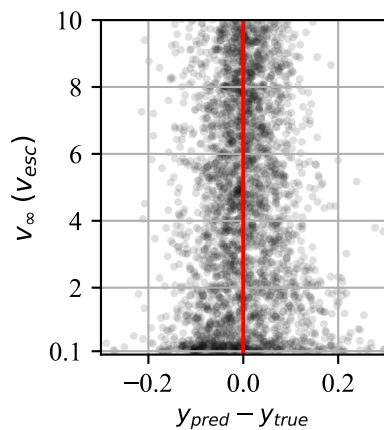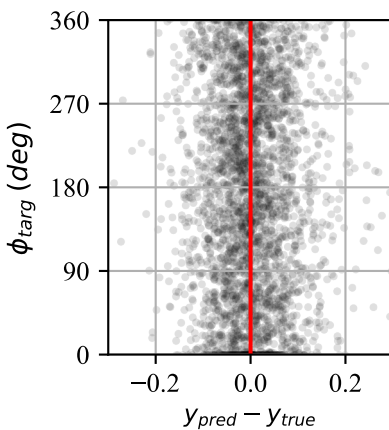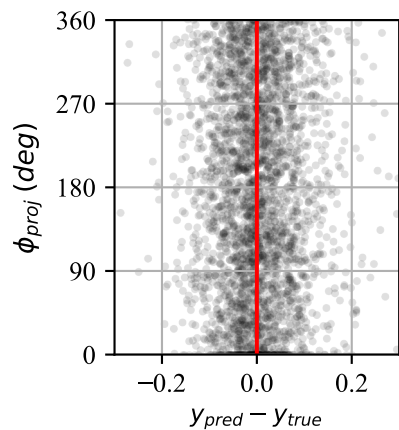

Supplement: Supplementary file 1 — Supplementary information (ZIP 48.3 MB) [file 40668_2020_34_MOESM1_ESM.zip › residuals_debris_mixing_impurity_gp_11884.pdf]

Target:  $\delta_{\text{deb}}^{\text{mix}}$ 

Method: MLP

TSS = 11,884

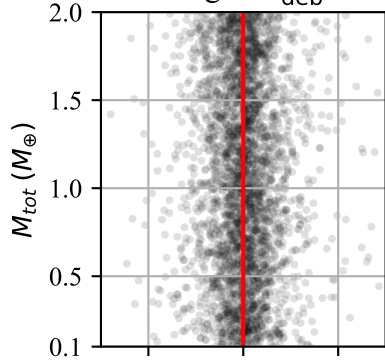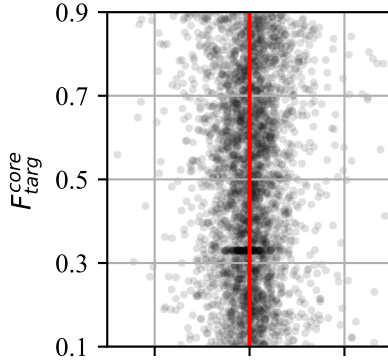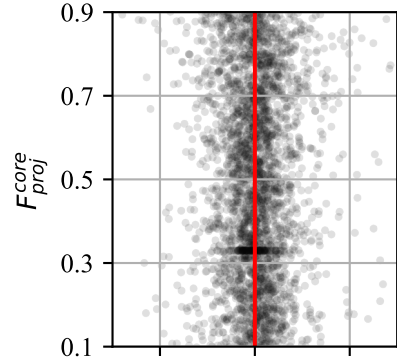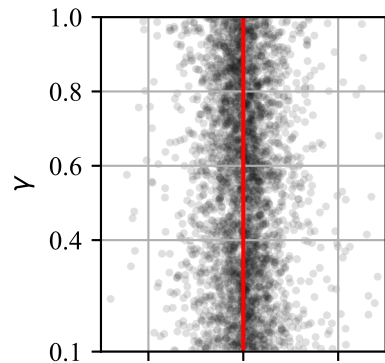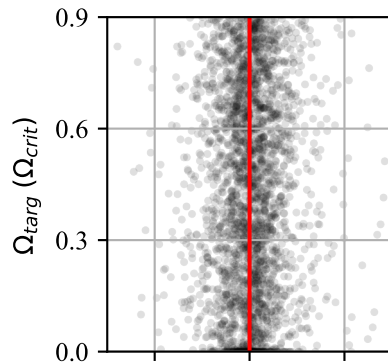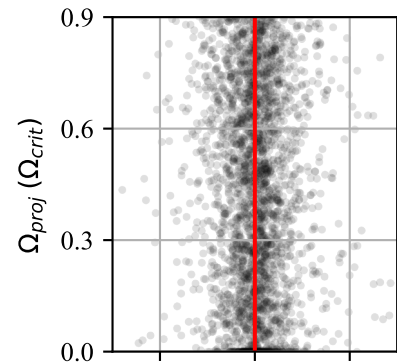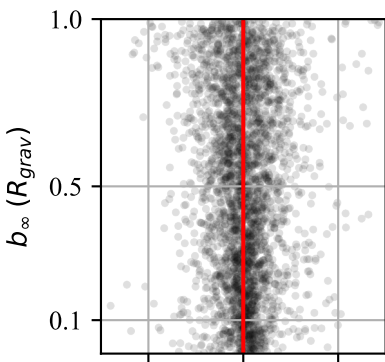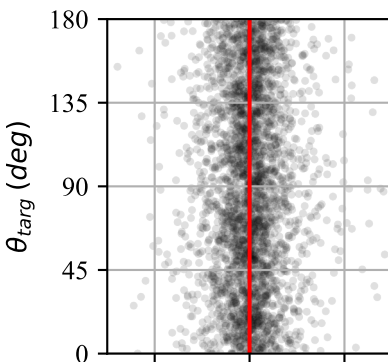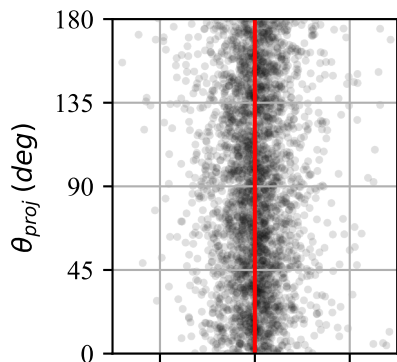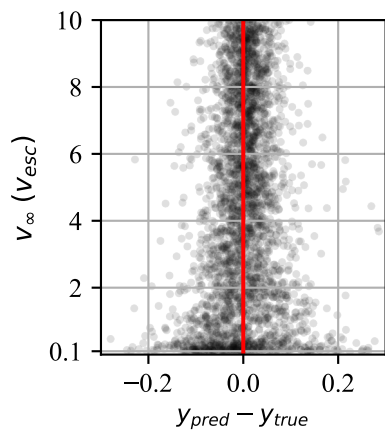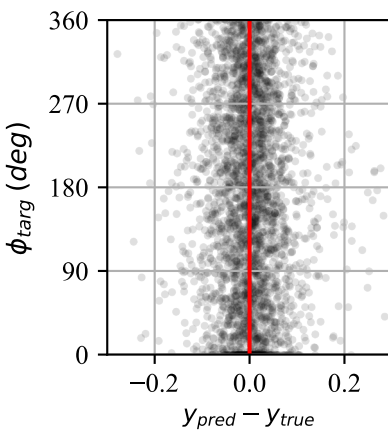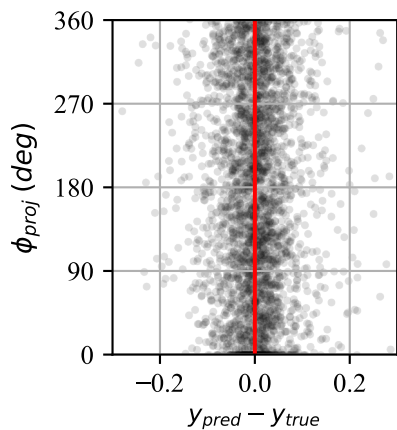

Supplement: Supplementary file 1 — Supplementary information (ZIP 48.3 MB) [file 40668_2020_34_MOESM1_ESM.zip › residuals_debris_mixing_impurity_mlp_11884.pdf]

Target:  $\delta_{\text{deb}}^{\text{mix}}$ 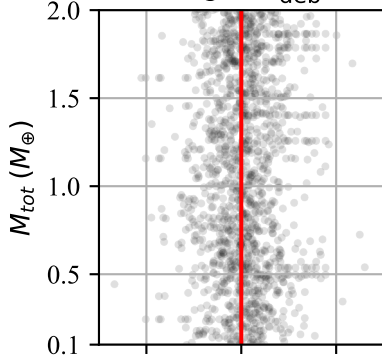

Method: PCE

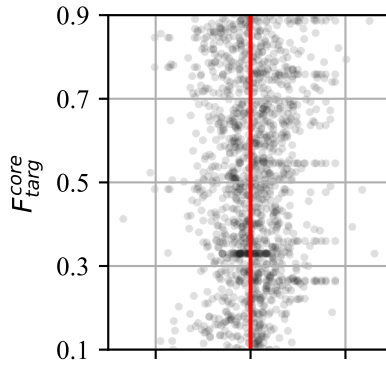

TSS = 11,884

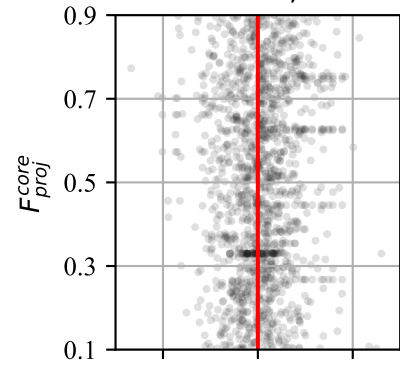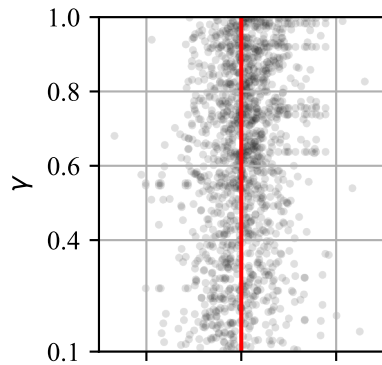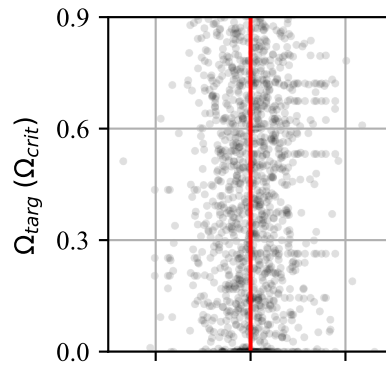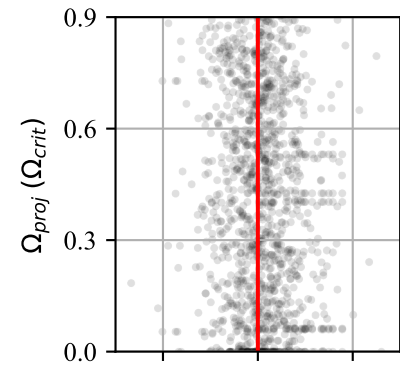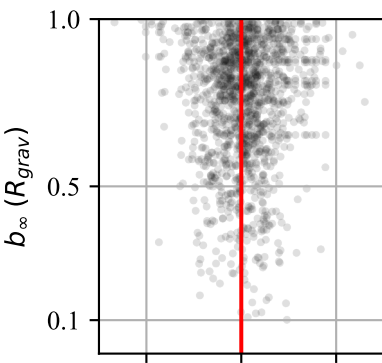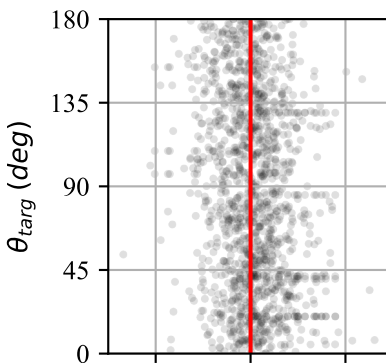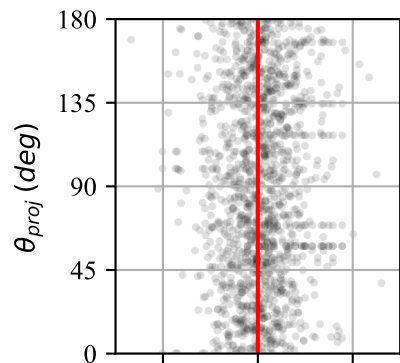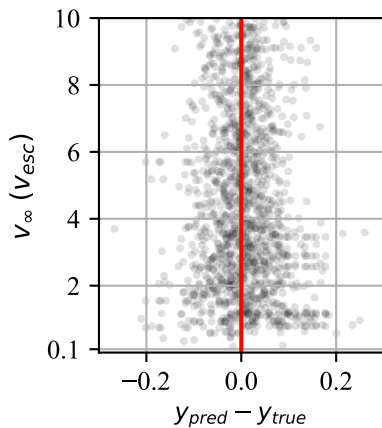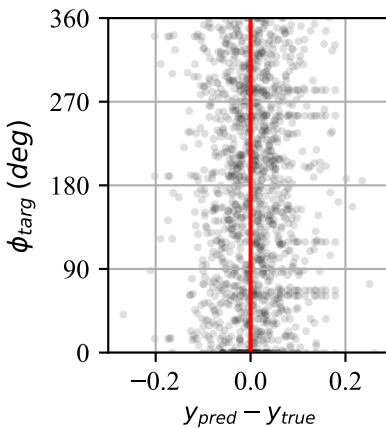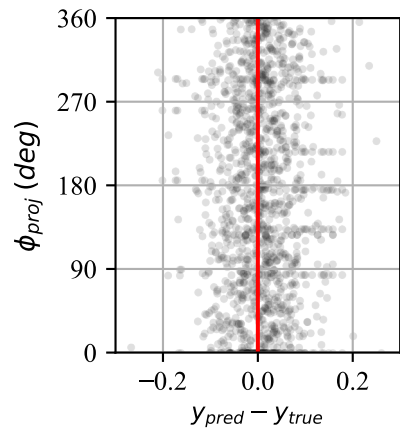

Supplement: Supplementary file 1 — Supplementary information (ZIP 48.3 MB) [file 40668_2020_34_MOESM1_ESM.zip › residuals_debris_mixing_impurity_pce_11884.pdf]

Target:  $\delta_{\text{deb}}^{\text{mix}}$ 

Method: XGB

TSS = 11,884

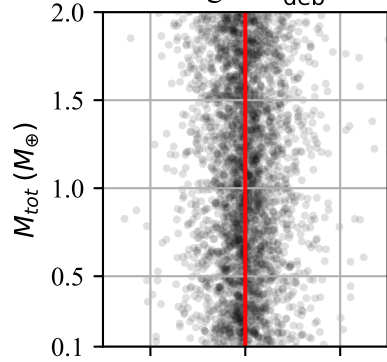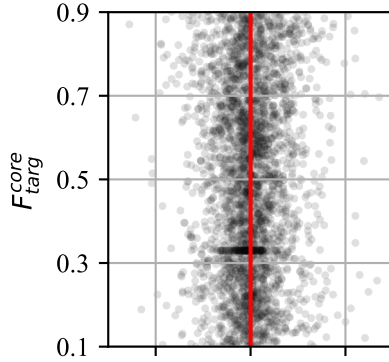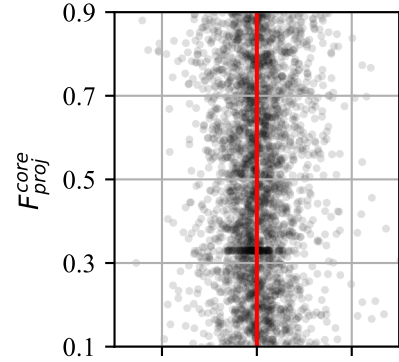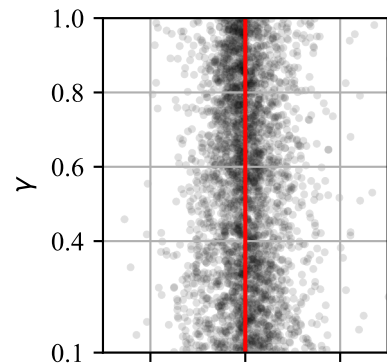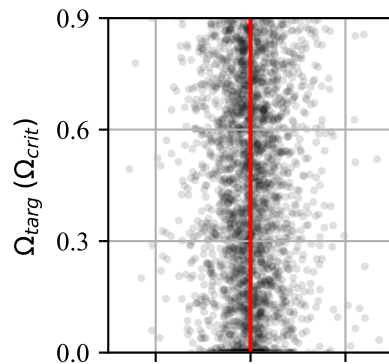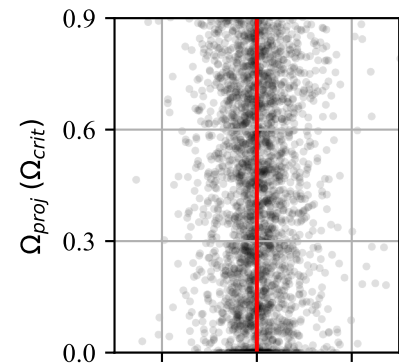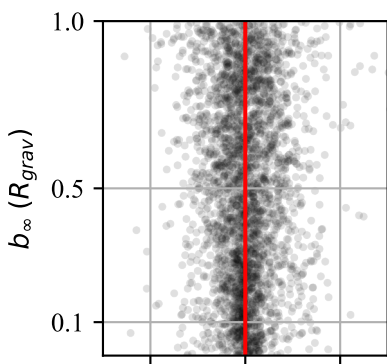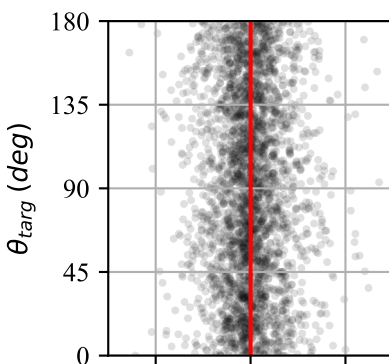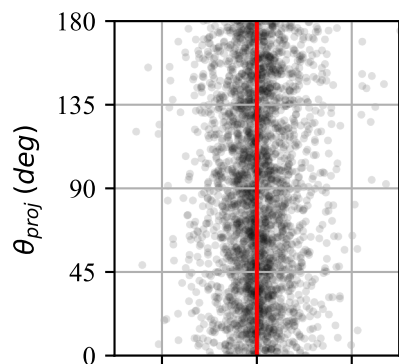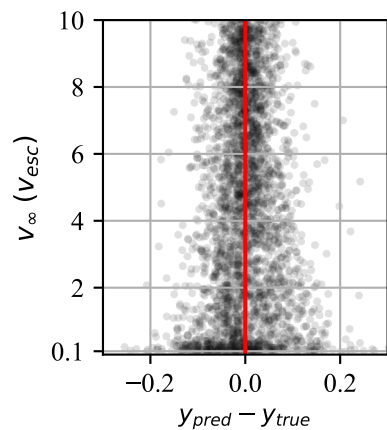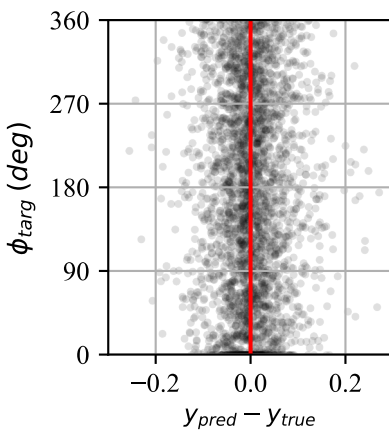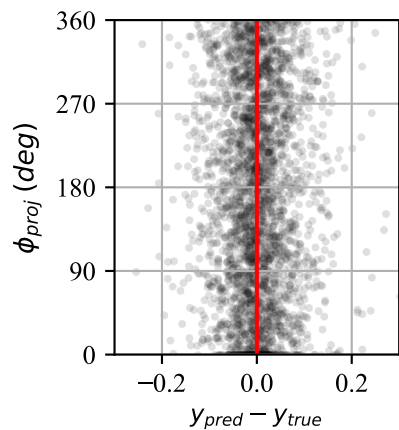

Supplement: Supplementary file 1 — Supplementary information (ZIP 48.3 MB) [file 40668_2020_34_MOESM1_ESM.zip › residuals_debris_mixing_impurity_xgb_11884.pdf]

Target:  $\theta_{\text{deb}}^{\text{stddev}}$

Method: GP

TSS = 11,884

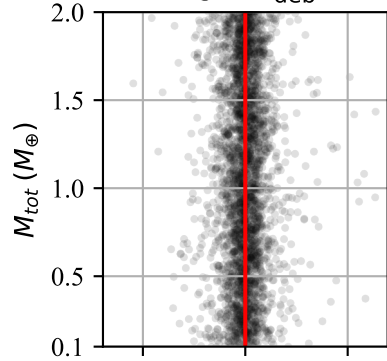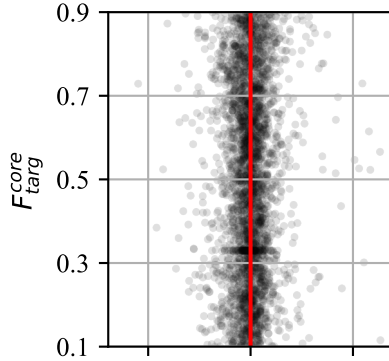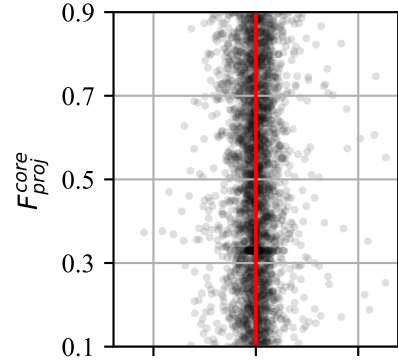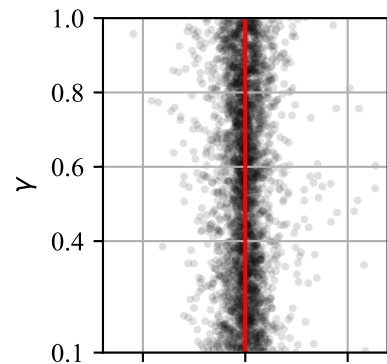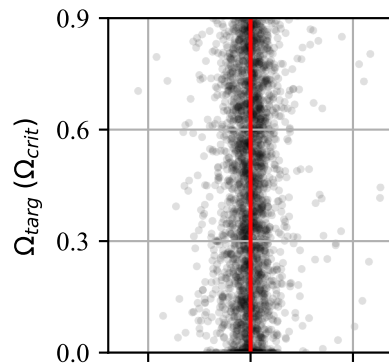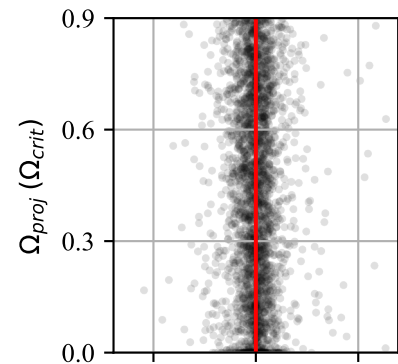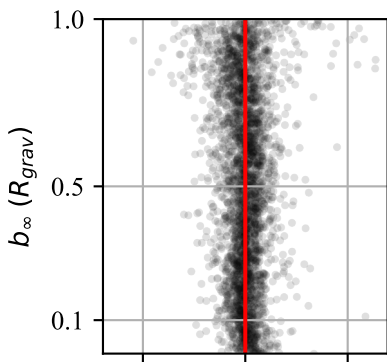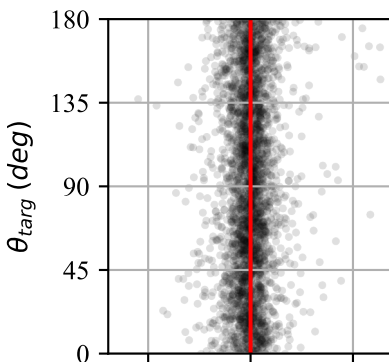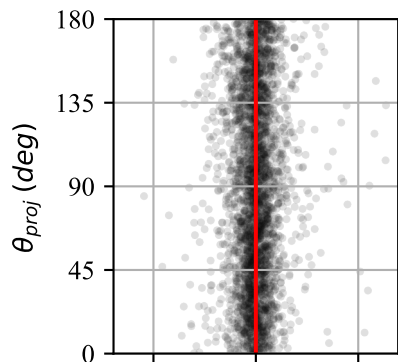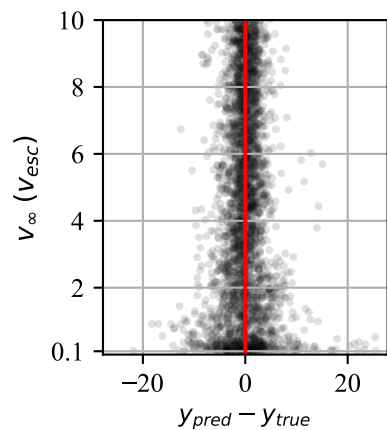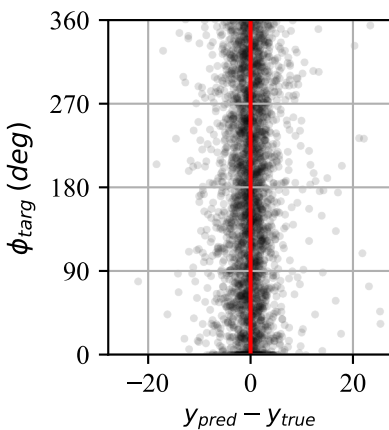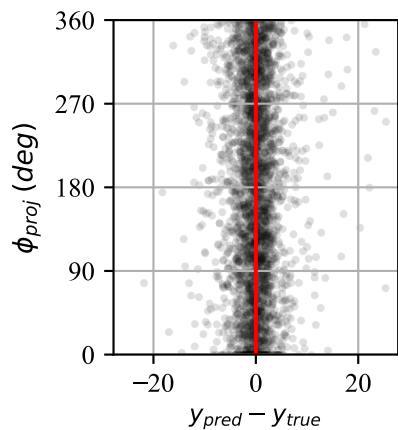

Supplement: Supplementary file 1 — Supplementary information (ZIP 48.3 MB) [file 40668_2020_34_MOESM1_ESM.zip › residuals_debris_stddev_altitude_gp_11884.pdf]

Target:  $\theta_{\text{deb}}^{\text{stddev}}$ 

Method: MLP

TSS = 11,884

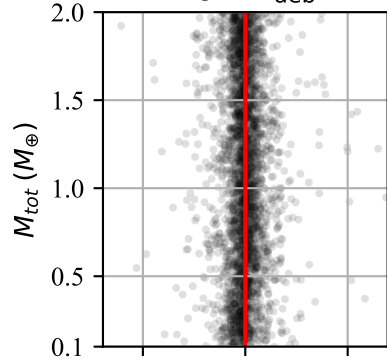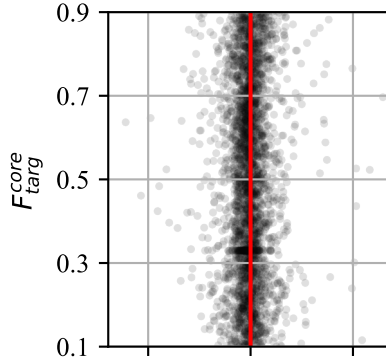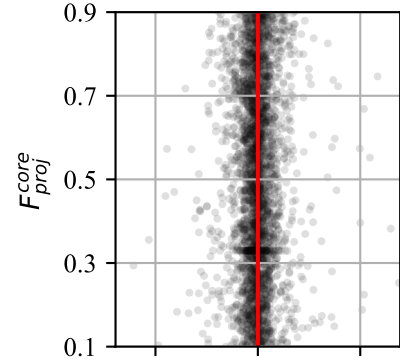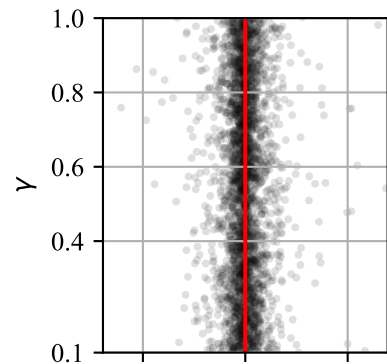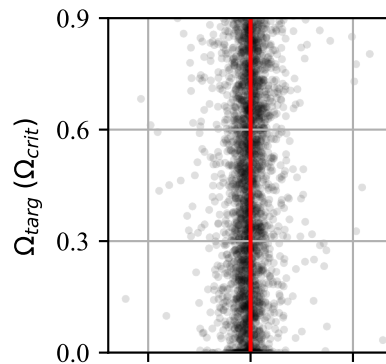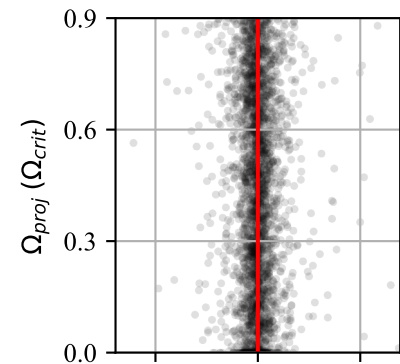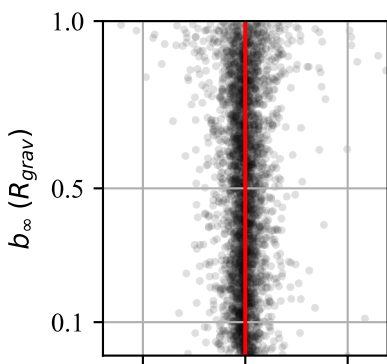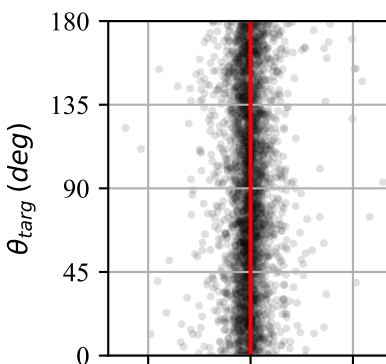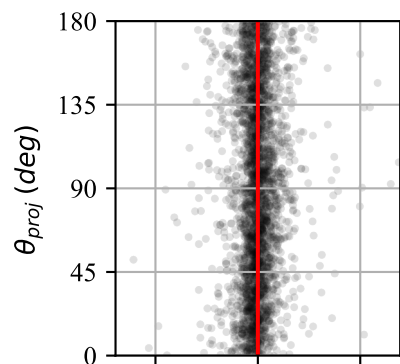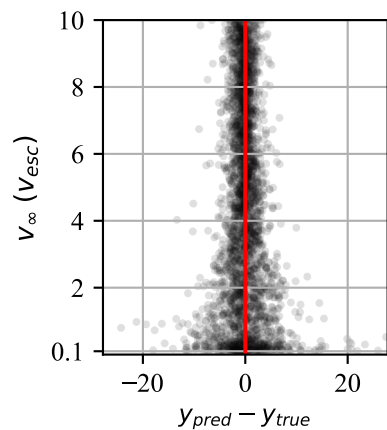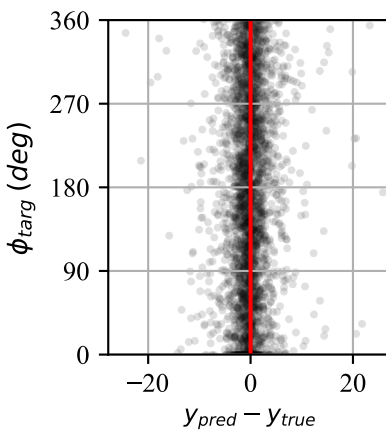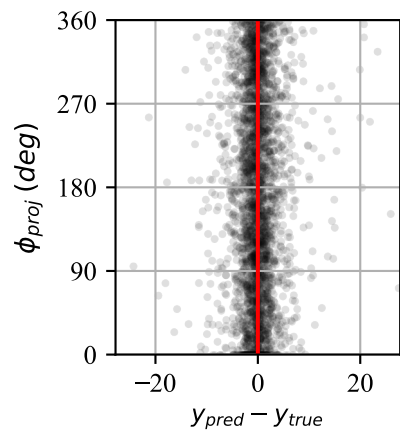

Supplement: Supplementary file 1 — Supplementary information (ZIP 48.3 MB) [file 40668_2020_34_MOESM1_ESM.zip › residuals_debris_stddev_altitude_mlp_11884.pdf]

Target:  $\theta_{\text{deb}}^{\text{stdev}}$

Method: PCE

TSS = 11,884

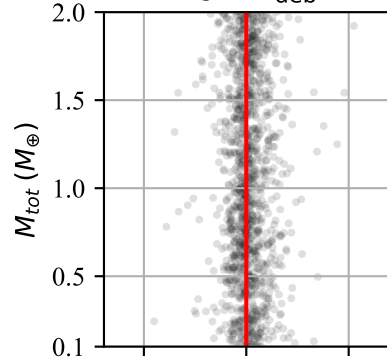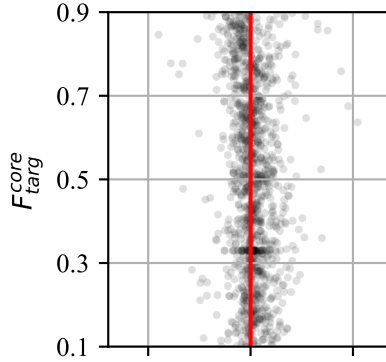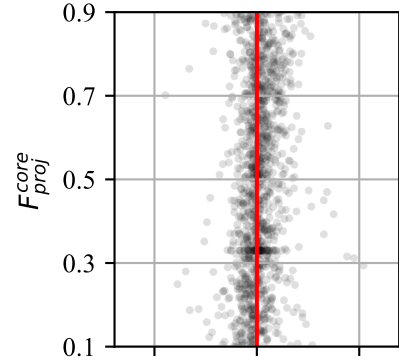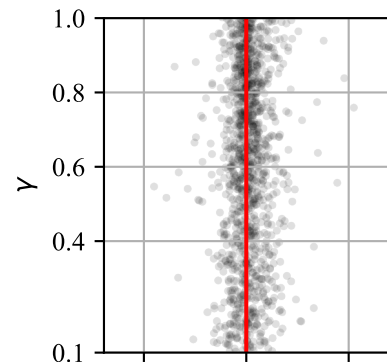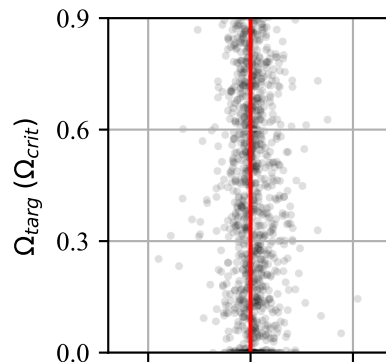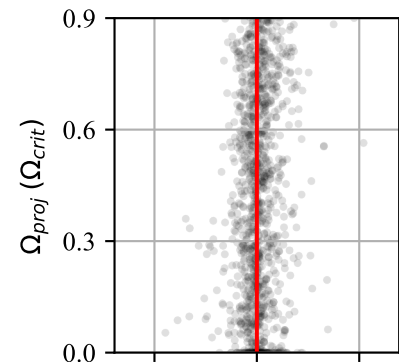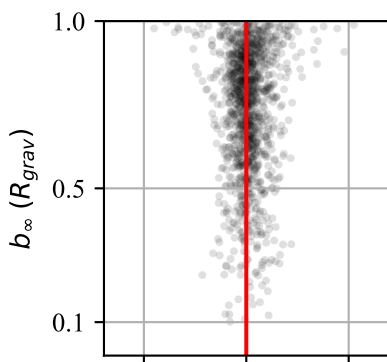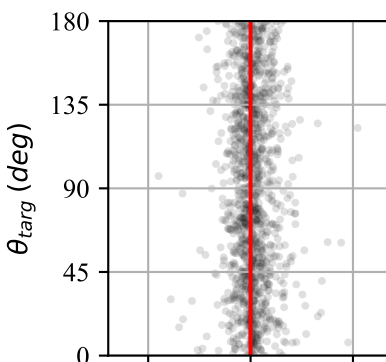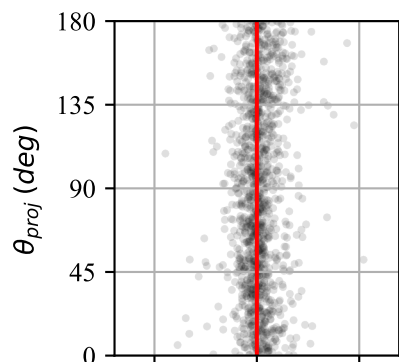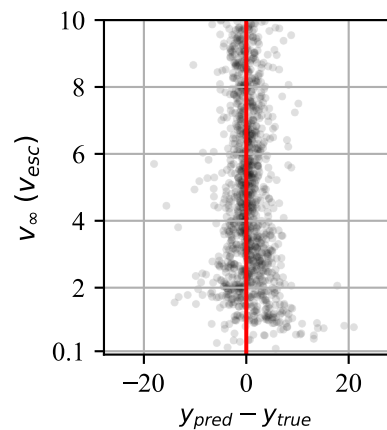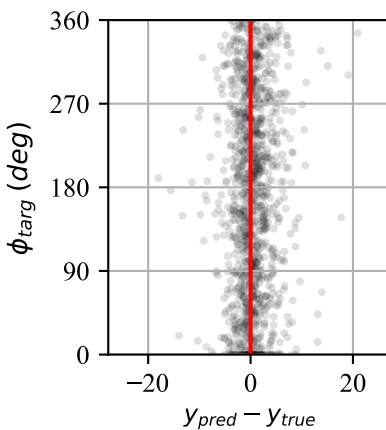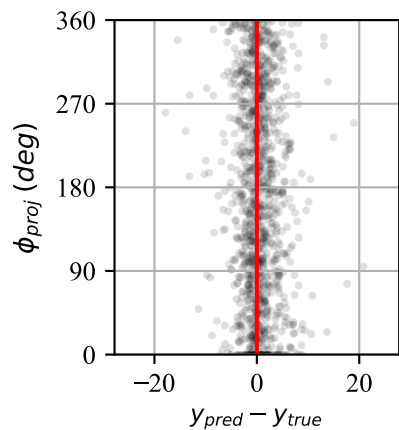

Supplement: Supplementary file 1 — Supplementary information (ZIP 48.3 MB) [file 40668_2020_34_MOESM1_ESM.zip › residuals_debris_stddev_altitude_pce_11884.pdf]

Target:  $\theta^{\text{stddev}}_{\text{deb}}$ 

Method: XGB

TSS = 11,884

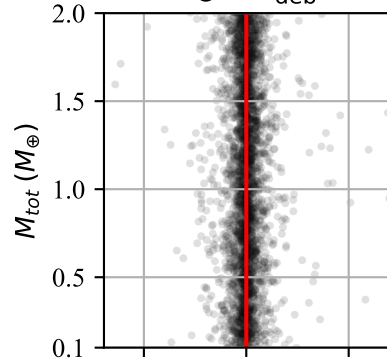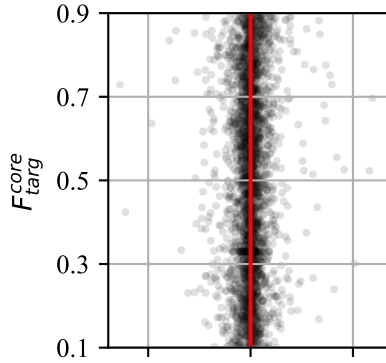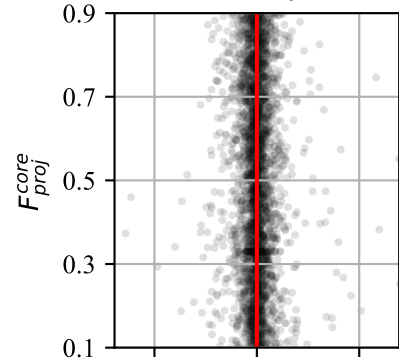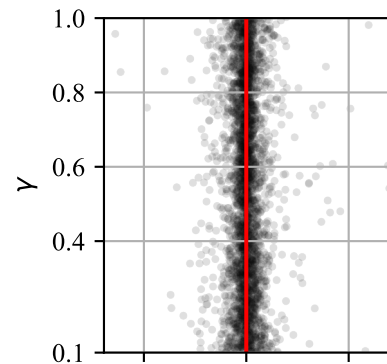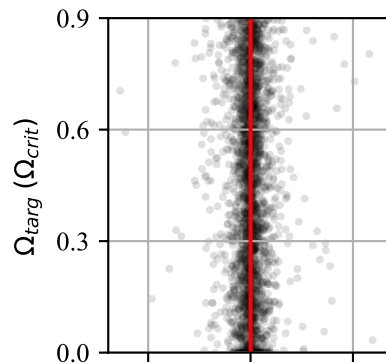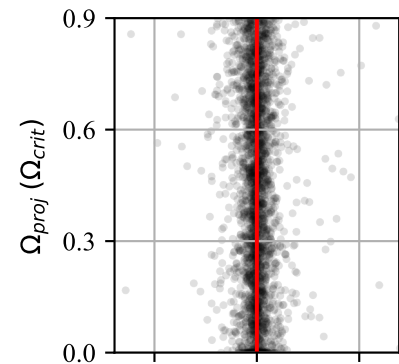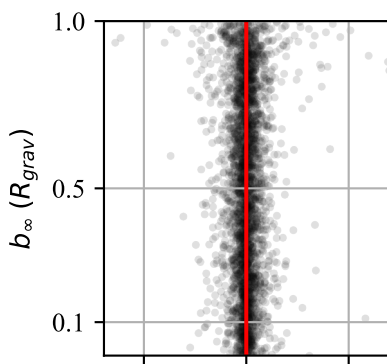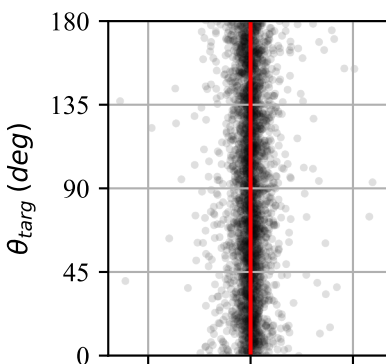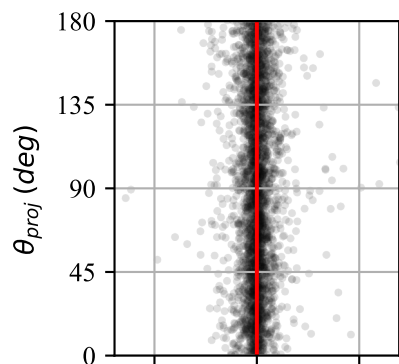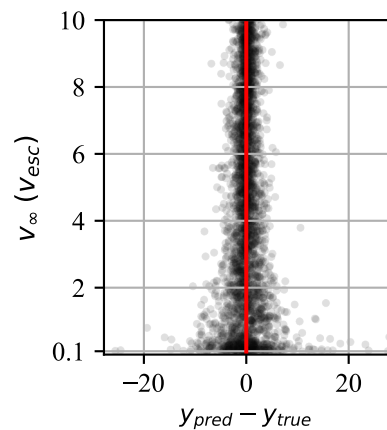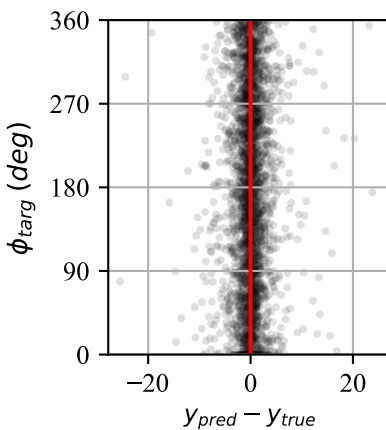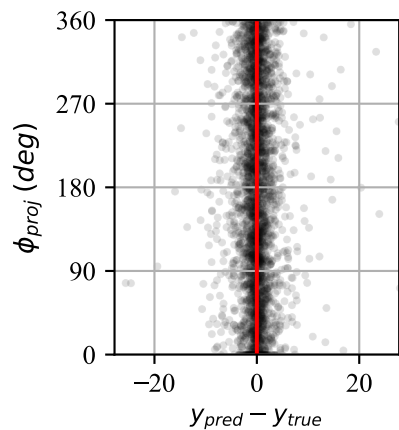

Supplement: Supplementary file 1 — Supplementary information (ZIP 48.3 MB) [file 40668_2020_34_MOESM1_ESM.zip › residuals_debris_stddev_altitude_xgb_11884.pdf]

Target:  $\phi_{\text{deb}}^{\text{stdev}}$ 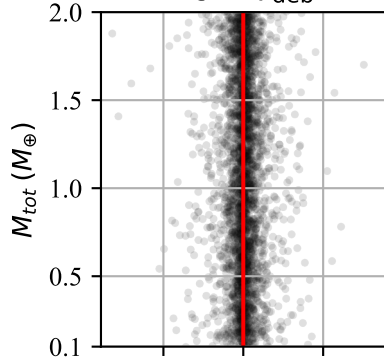

Method: GP

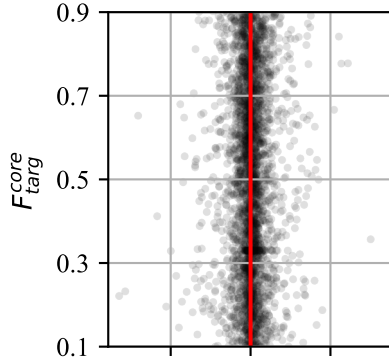

TSS = 11,884

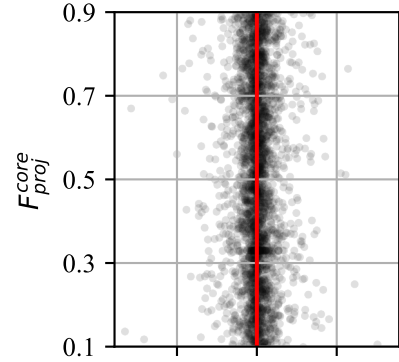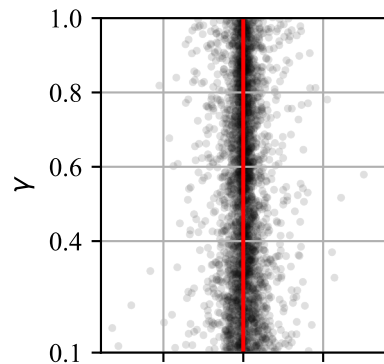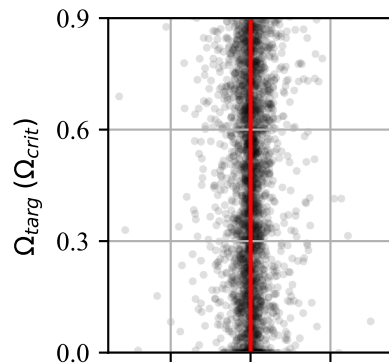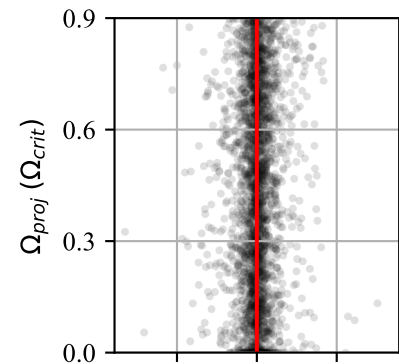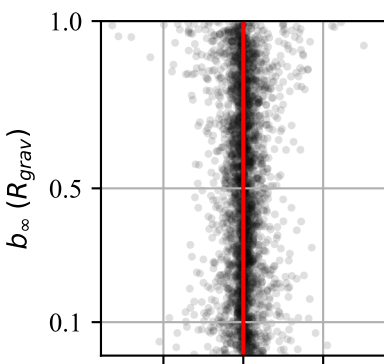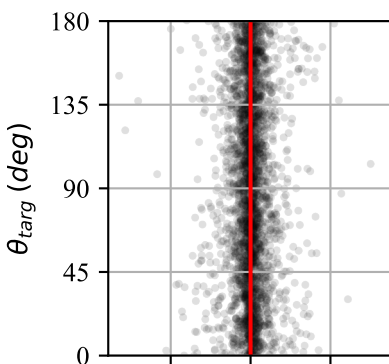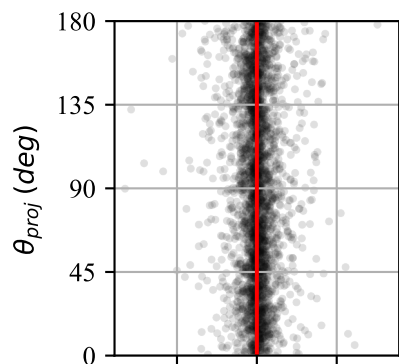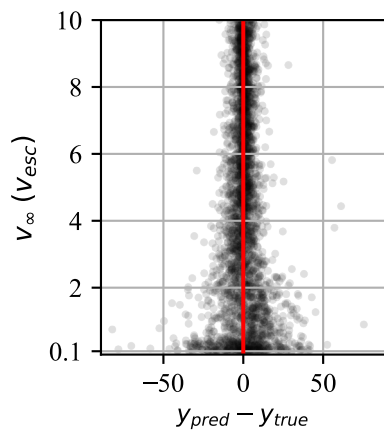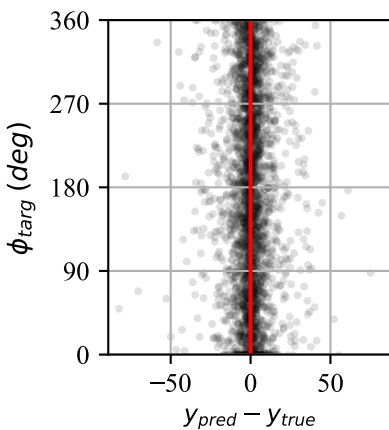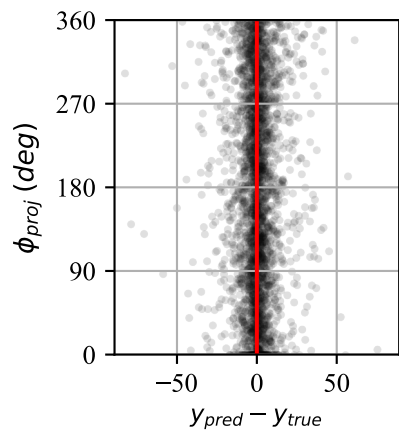

Supplement: Supplementary file 1 — Supplementary information (ZIP 48.3 MB) [file 40668_2020_34_MOESM1_ESM.zip › residuals_debris_stddev_azimuth_gp_11884.pdf]

Target:  $\phi_{\text{deb}}^{\text{stdev}}$ 

Method: MLP

TSS = 11,884

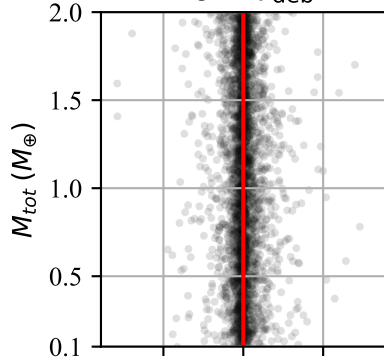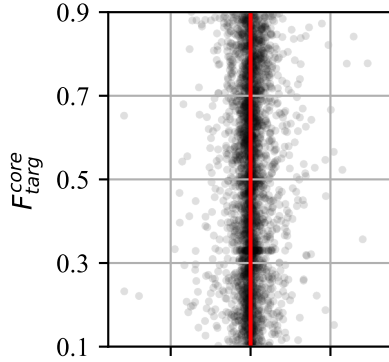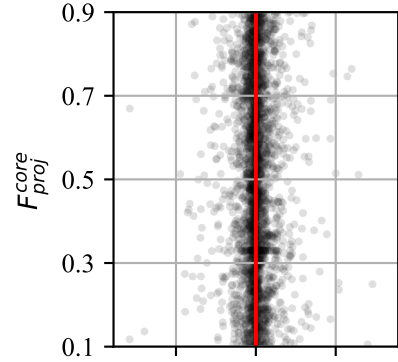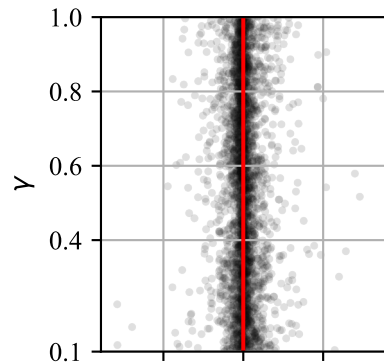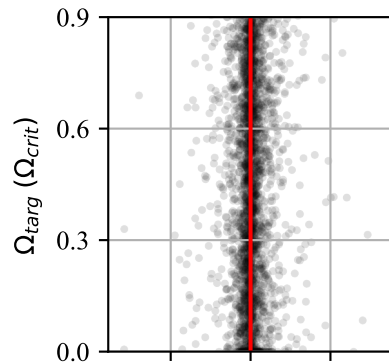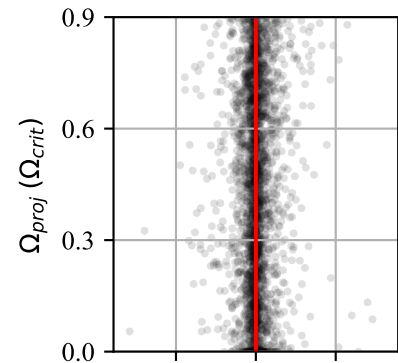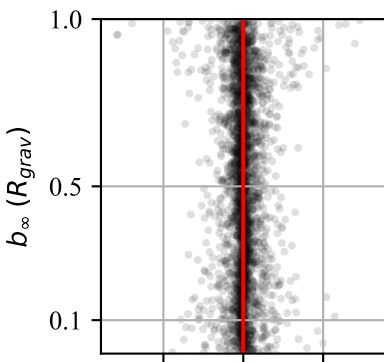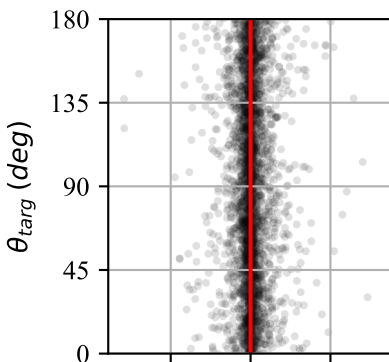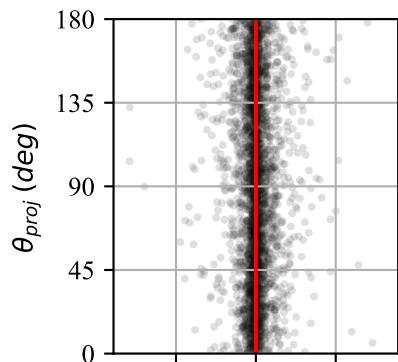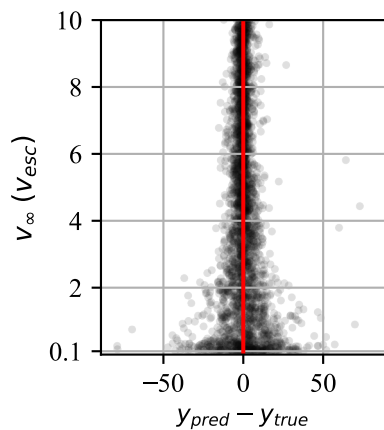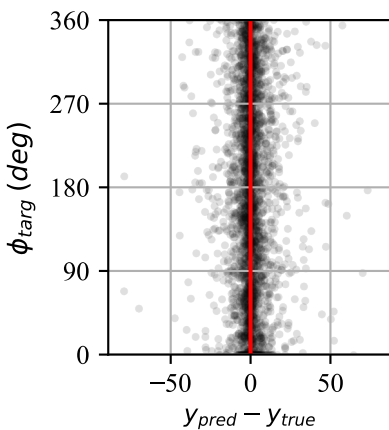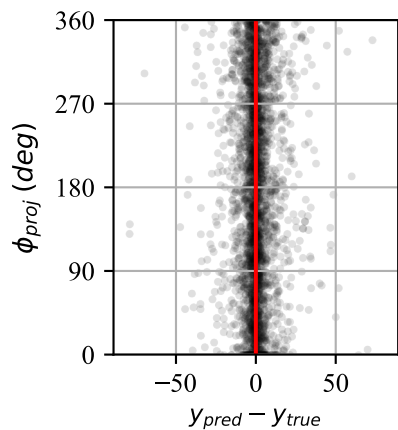

Supplement: Supplementary file 1 — Supplementary information (ZIP 48.3 MB) [file 40668_2020_34_MOESM1_ESM.zip › residuals_debris_stddev_azimuth_mlp_11884.pdf]

Target:  $\phi_{\text{deb}}^{\text{stdev}}$ 

Method: PCE

TSS = 11,884

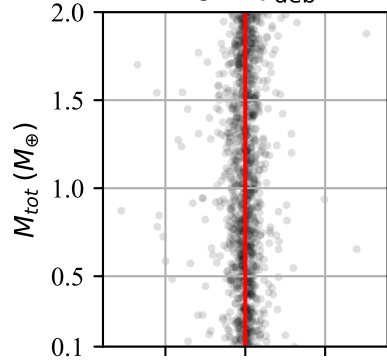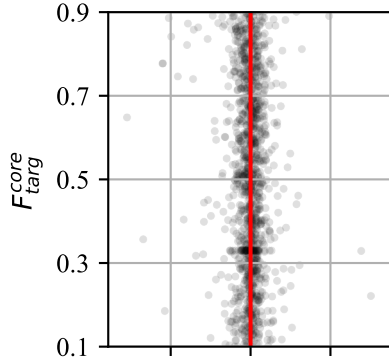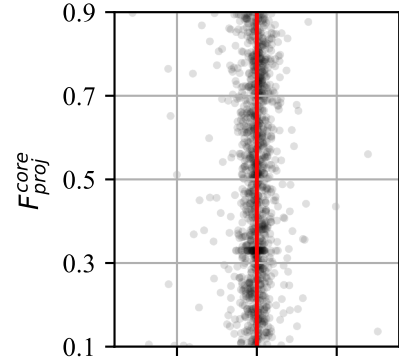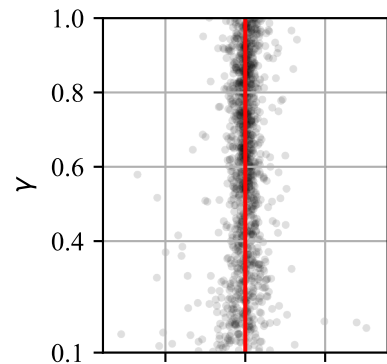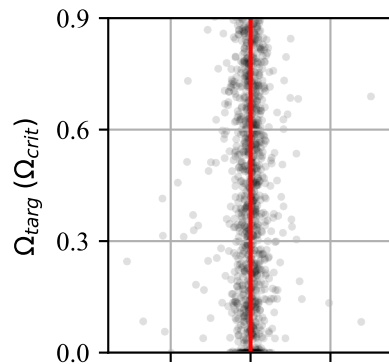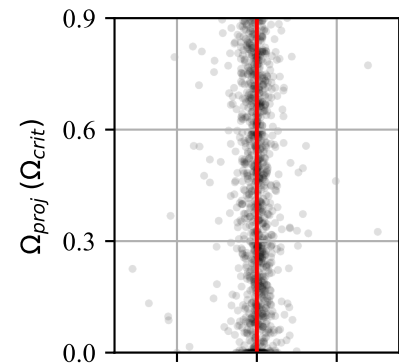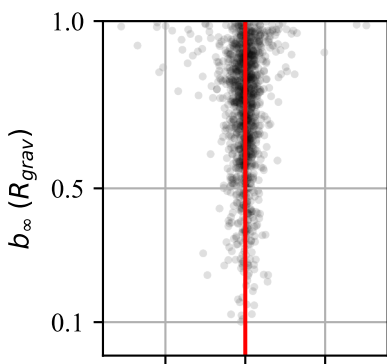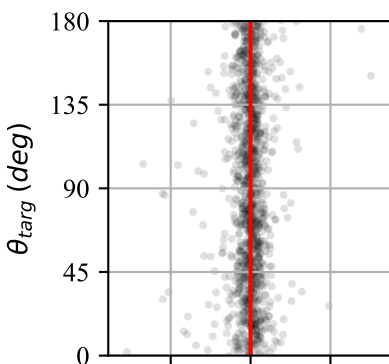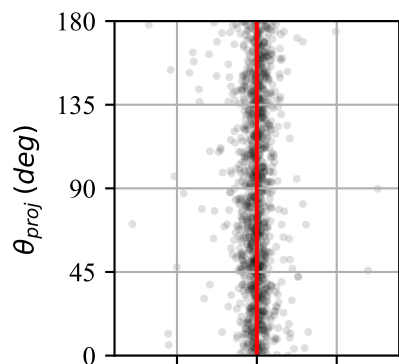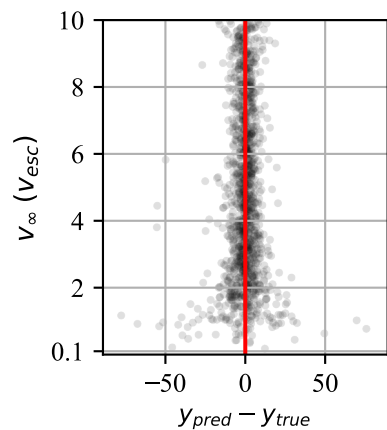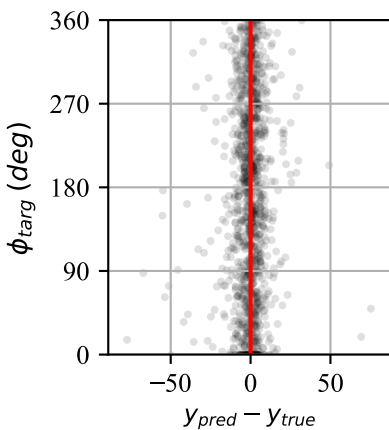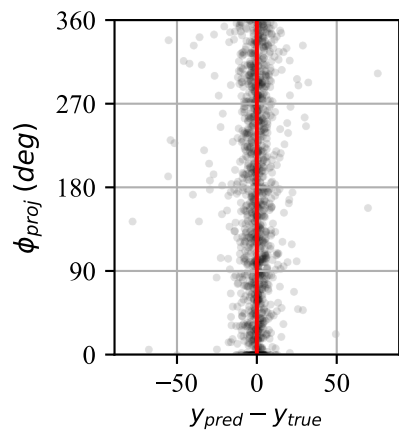

Supplement: Supplementary file 1 — Supplementary information (ZIP 48.3 MB) [file 40668_2020_34_MOESM1_ESM.zip › residuals_debris_stddev_azimuth_pce_11884.pdf]

Target:  $\phi_{\text{deb}}^{\text{stdev}}$ 

Method: XGB

TSS = 11,884

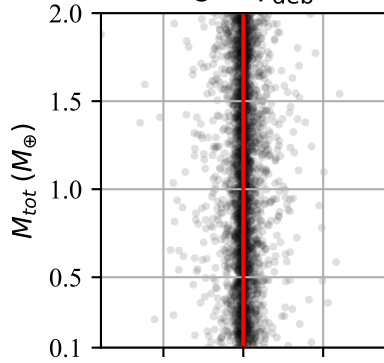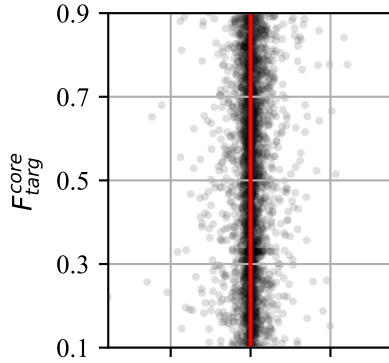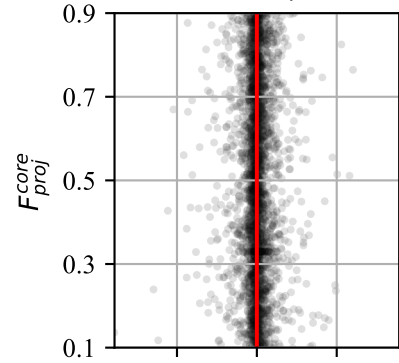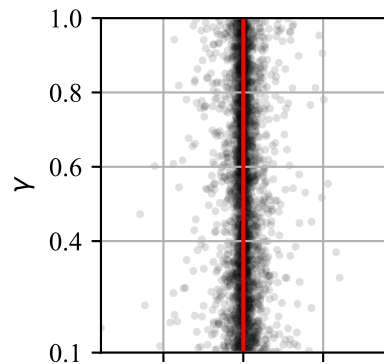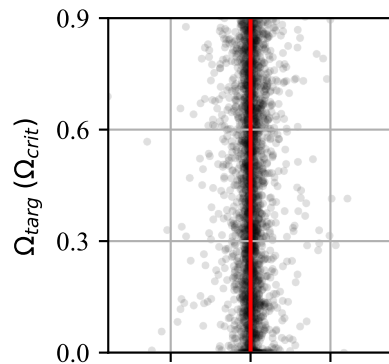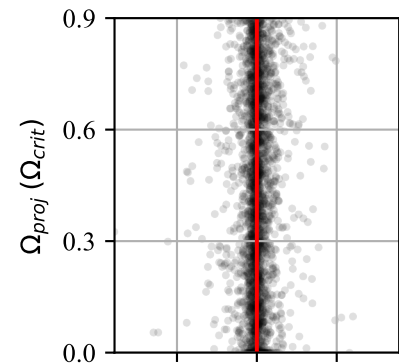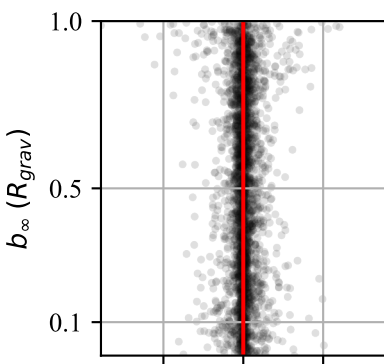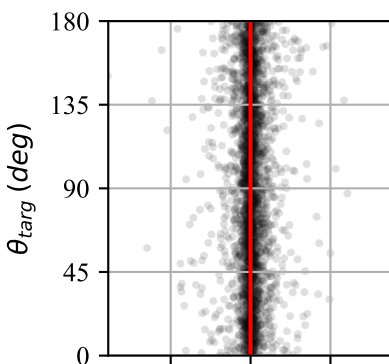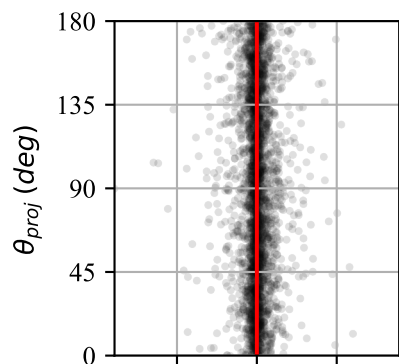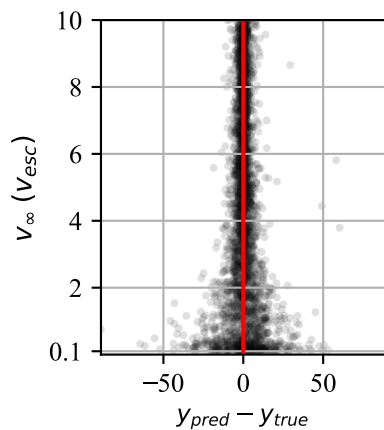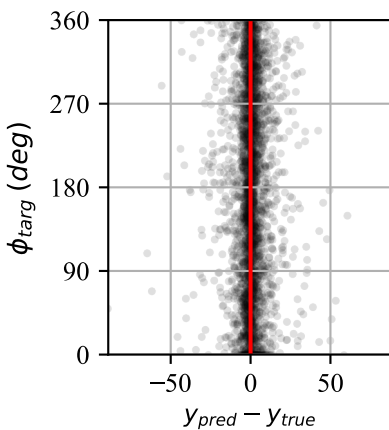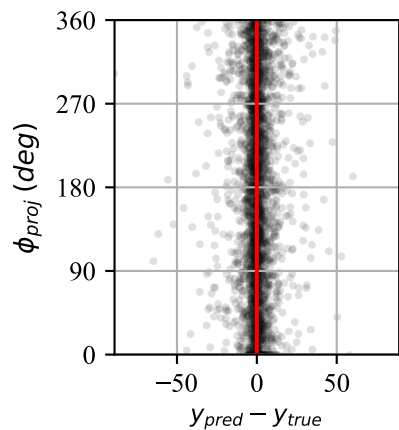

Supplement: Supplementary file 1 — Supplementary information (ZIP 48.3 MB) [file 40668_2020_34_MOESM1_ESM.zip › residuals_debris_stddev_azimuth_xgb_11884.pdf]

Target: J<sub>LR</sub>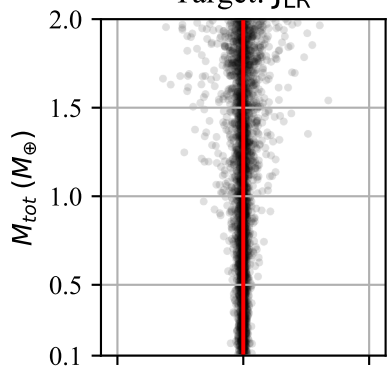

Method: GP

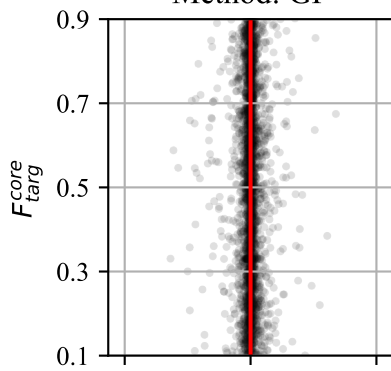

TSS = 11, 884

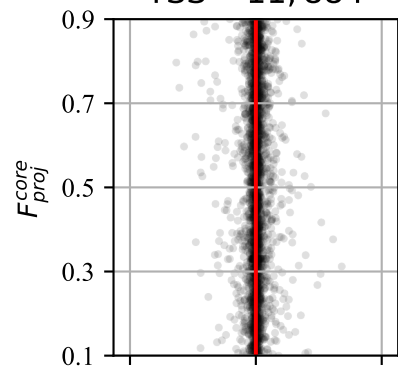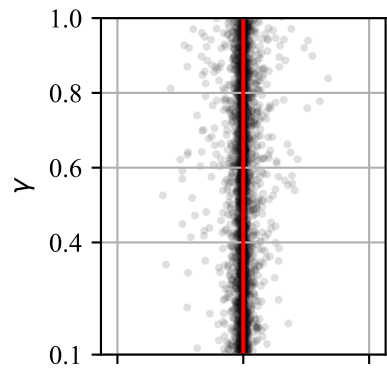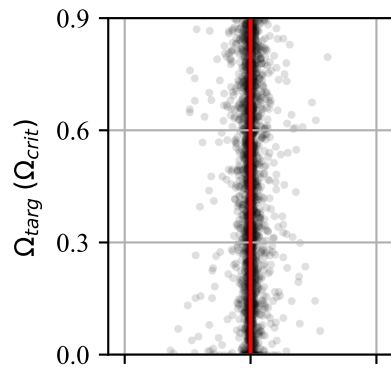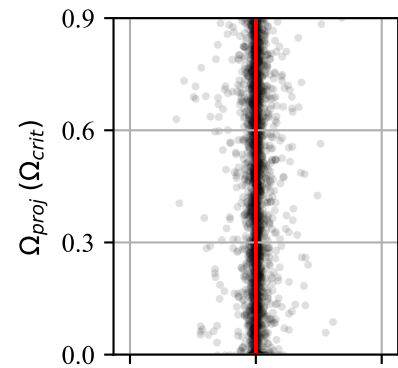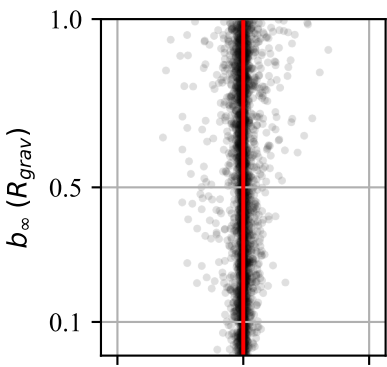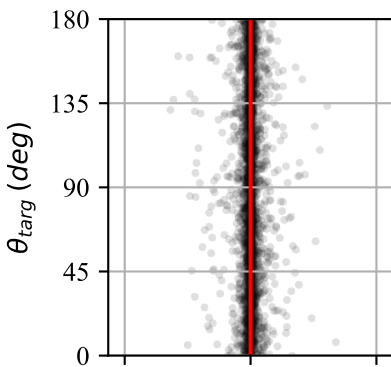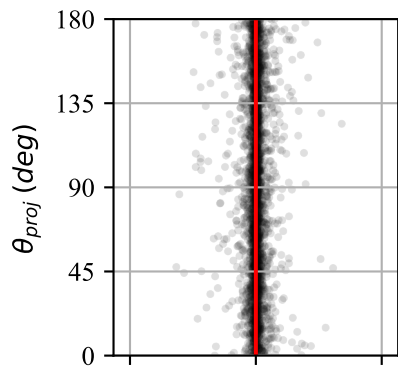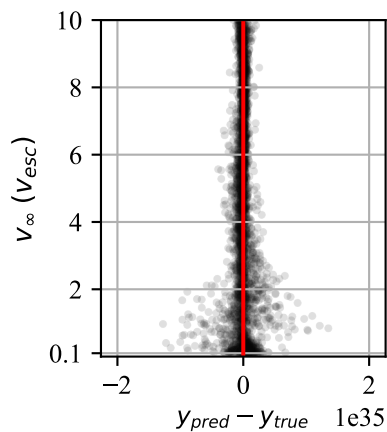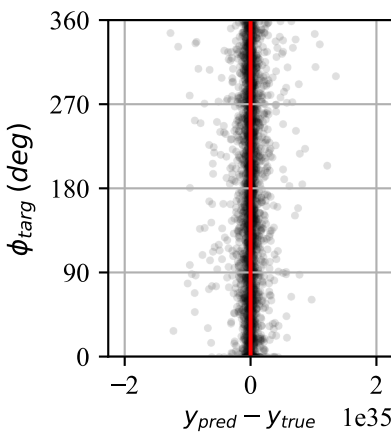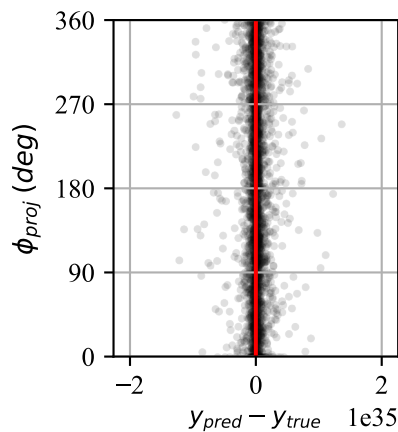

Supplement: Supplementary file 1 — Supplementary information (ZIP 48.3 MB) [file 40668_2020_34_MOESM1_ESM.zip › residuals_lr_angular_momentum_gp_11884.pdf]

Target: J<sub>LR</sub>

Method: MLP

TSS = 11,884

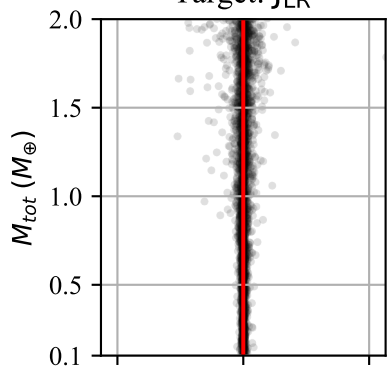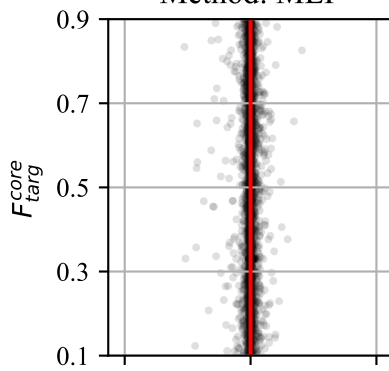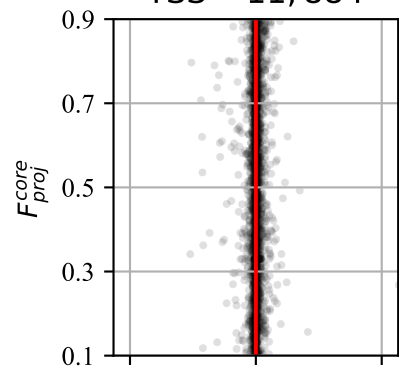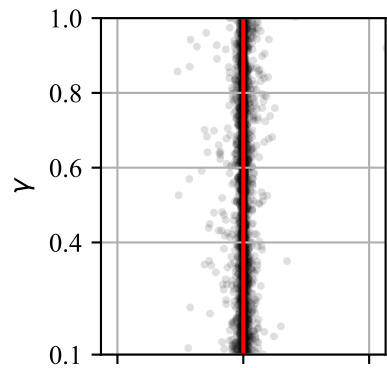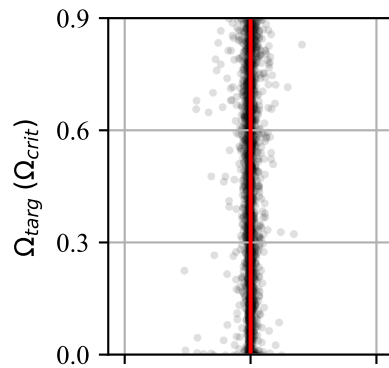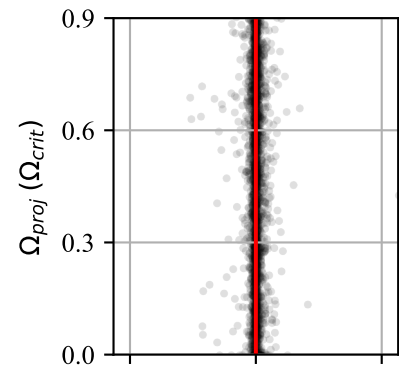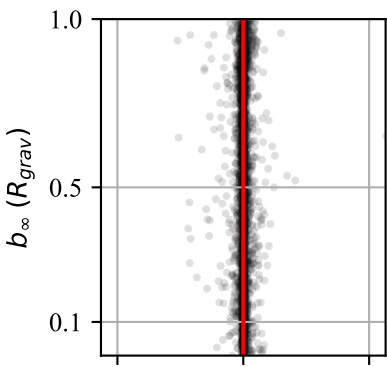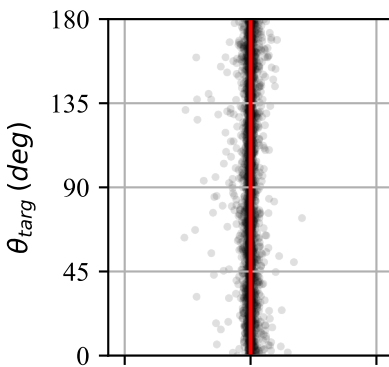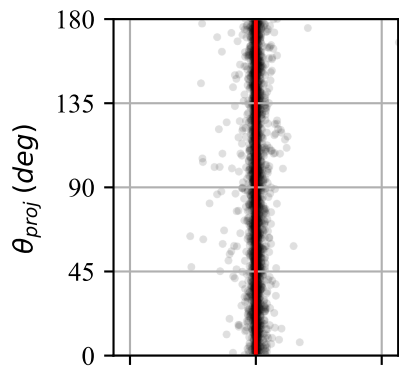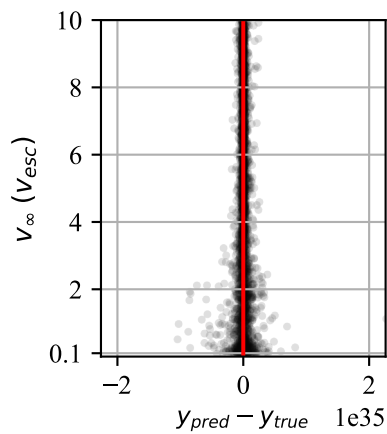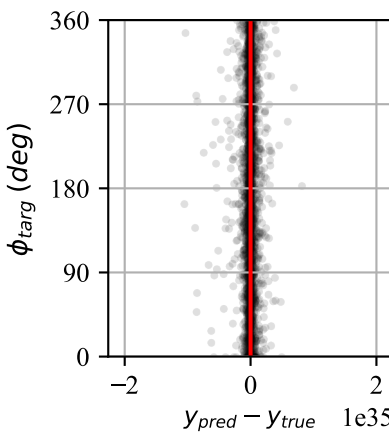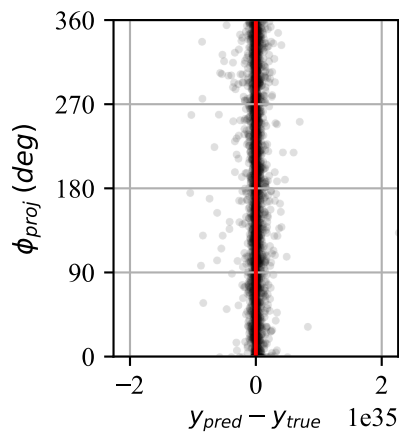

Supplement: Supplementary file 1 — Supplementary information (ZIP 48.3 MB) [file 40668_2020_34_MOESM1_ESM.zip › residuals_lr_angular_momentum_mlp_11884.pdf]

Target: J<sub>LR</sub>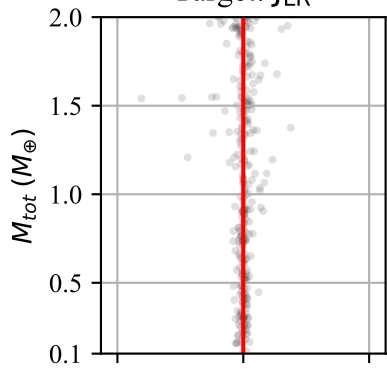

Method: PCE

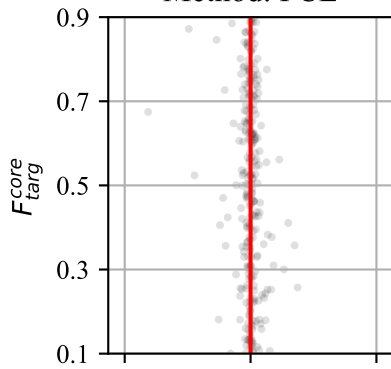

TSS = 11,884

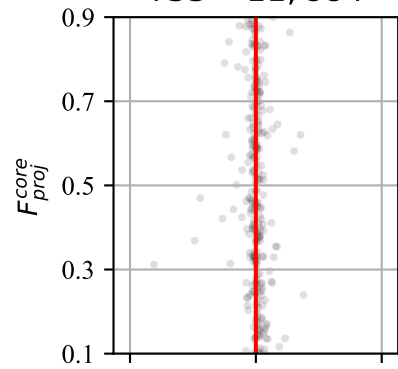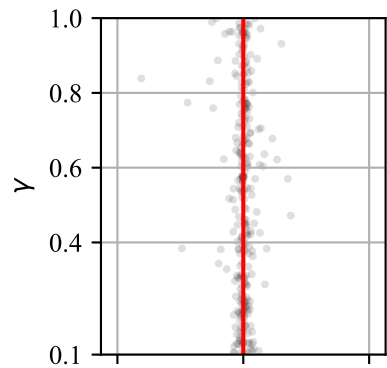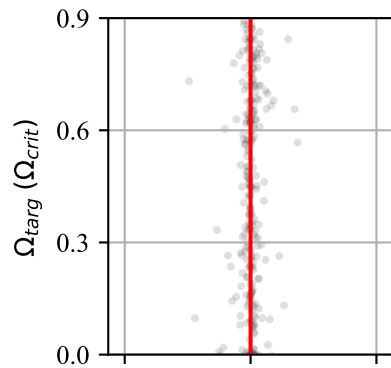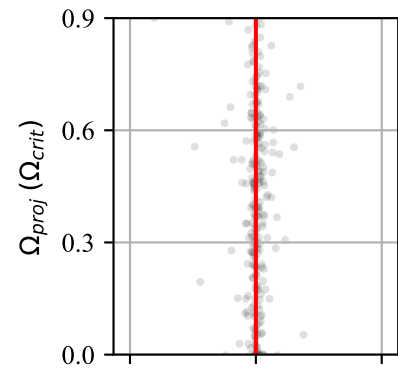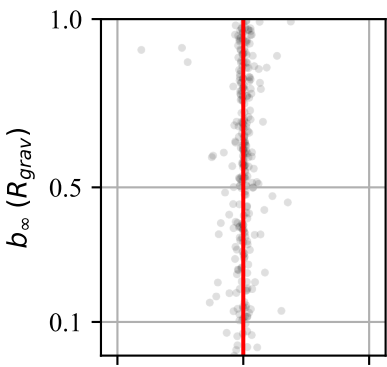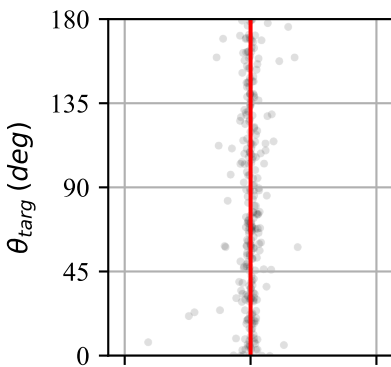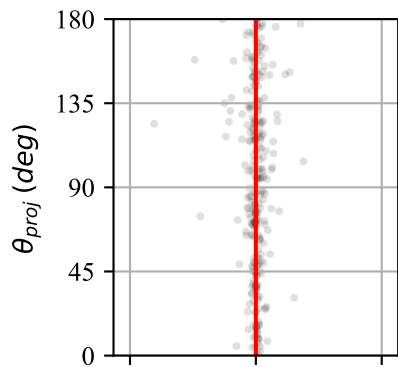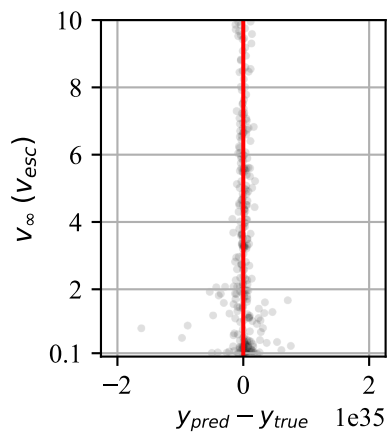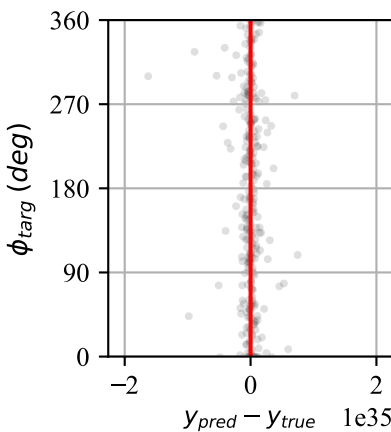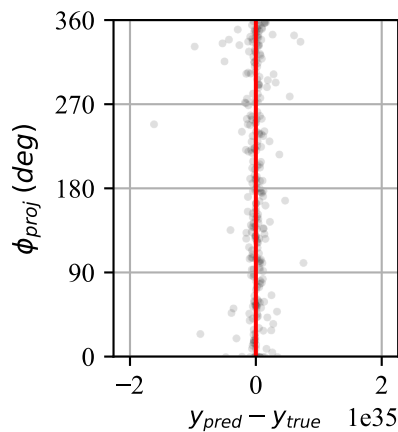

Supplement: Supplementary file 1 — Supplementary information (ZIP 48.3 MB) [file 40668_2020_34_MOESM1_ESM.zip › residuals_lr_angular_momentum_pce_11884.pdf]

Target: J<sub>LR</sub>

Method: XGB

TSS = 11, 884

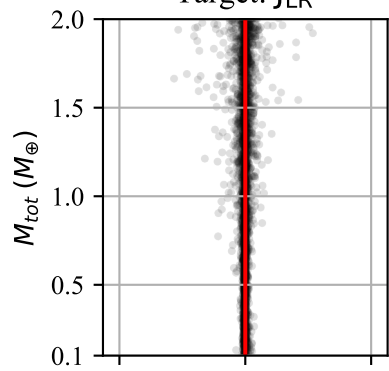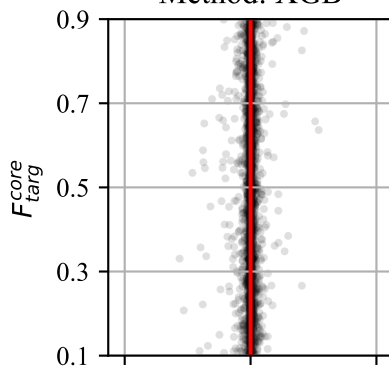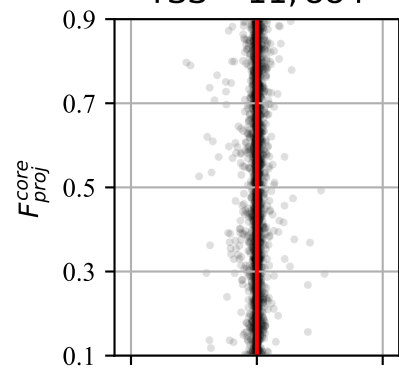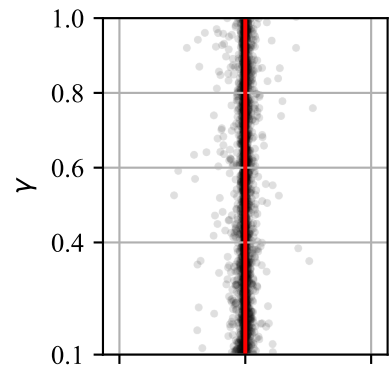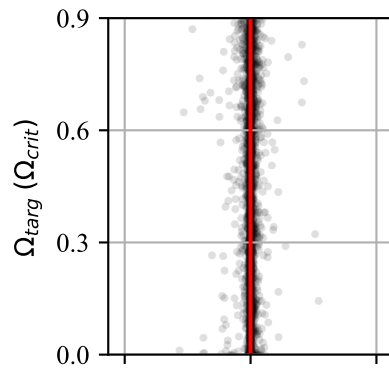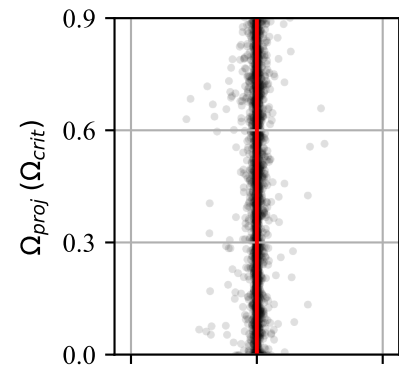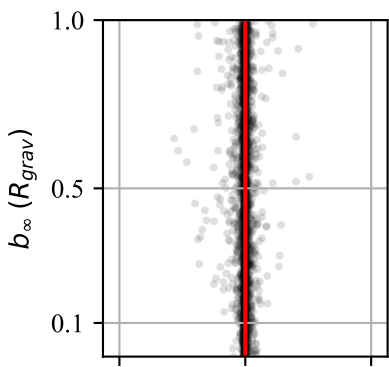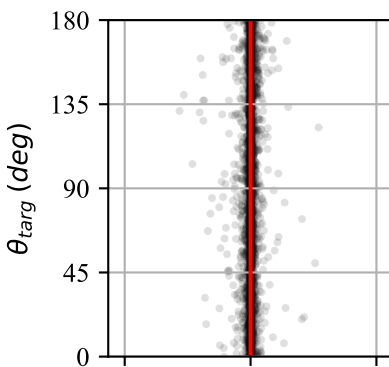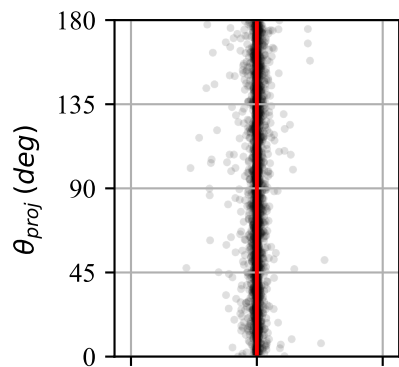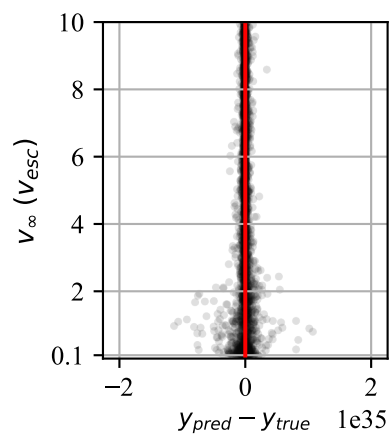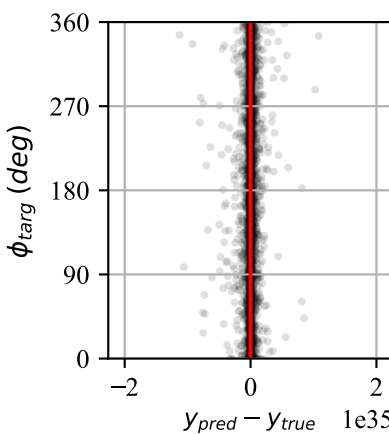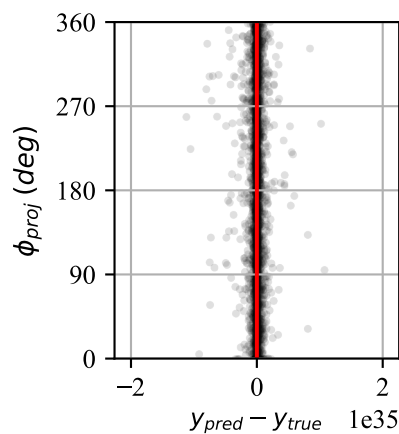

Supplement: Supplementary file 1 — Supplementary information (ZIP 48.3 MB) [file 40668_2020_34_MOESM1_ESM.zip › residuals_lr_angular_momentum_xgb_11884.pdf]

Target:  $F_{LR}^{core}$ 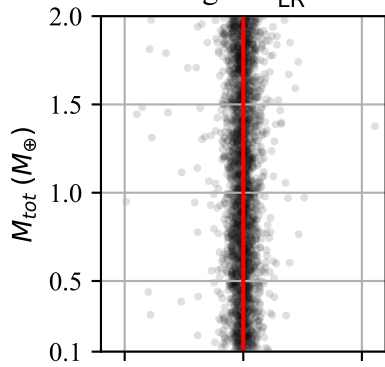

Method: GP

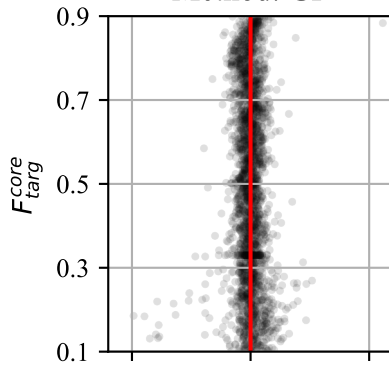

TSS = 11, 884

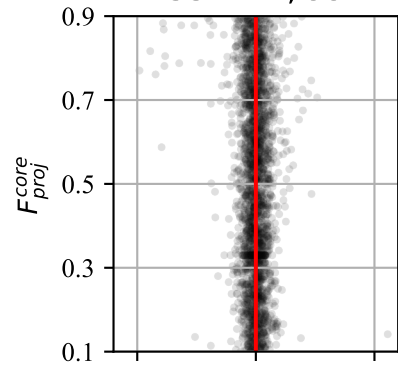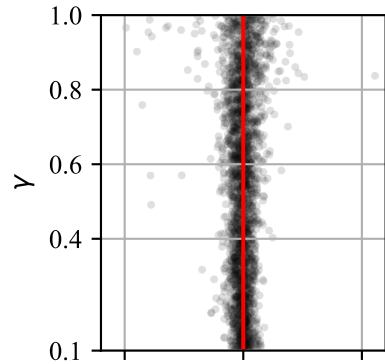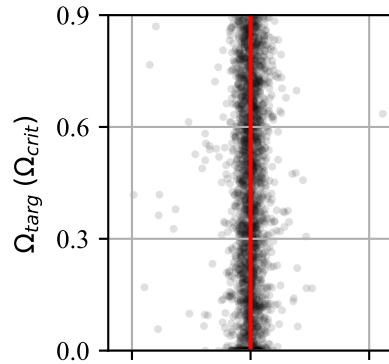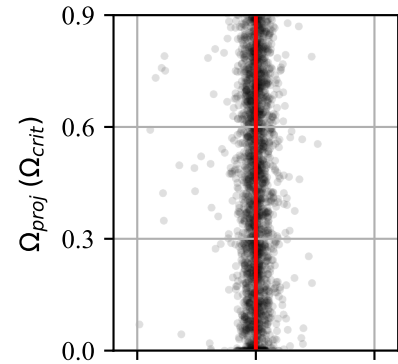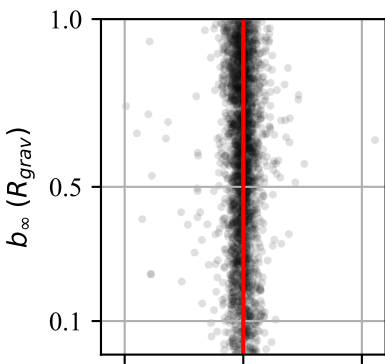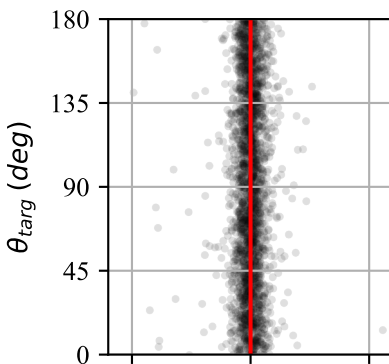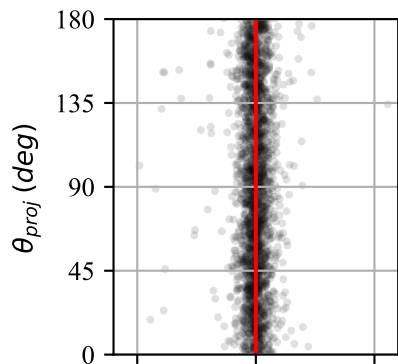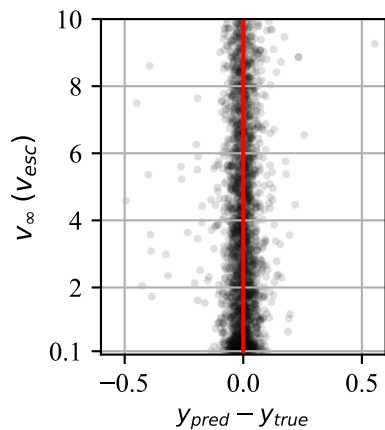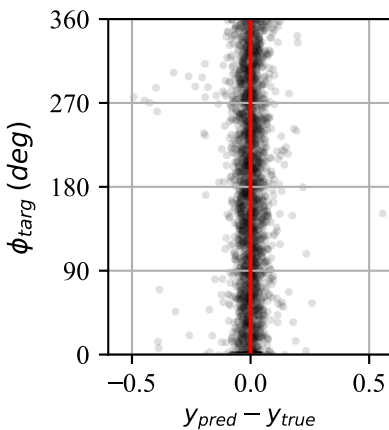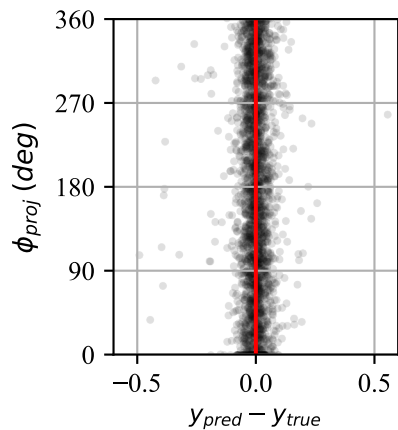

Supplement: Supplementary file 1 — Supplementary information (ZIP 48.3 MB) [file 40668_2020_34_MOESM1_ESM.zip › residuals_lr_core_fraction_gp_11884.pdf]

Target:  $F_{LR}^{core}$ 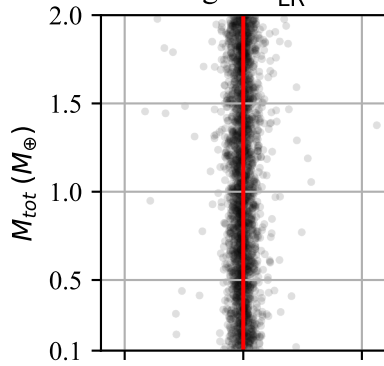

Method: MLP

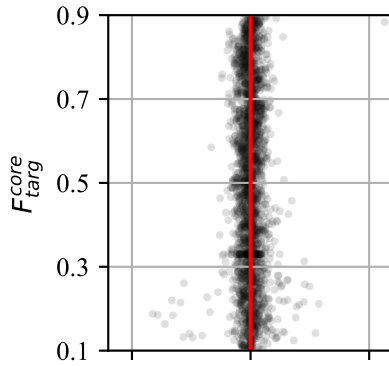

TSS = 11, 884

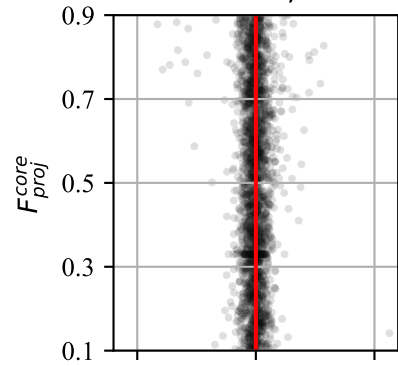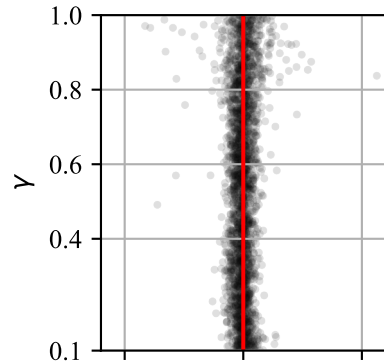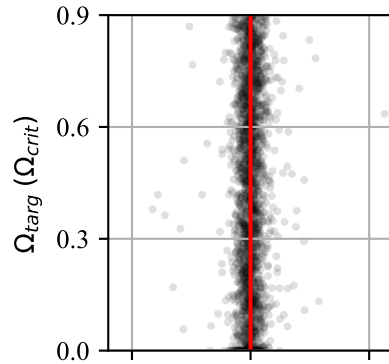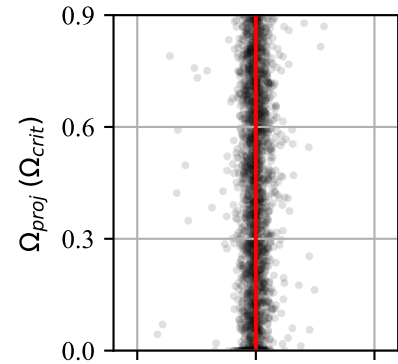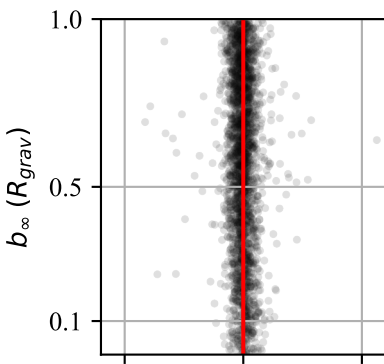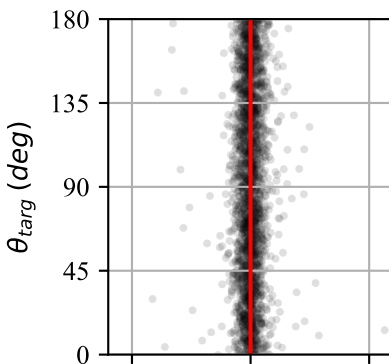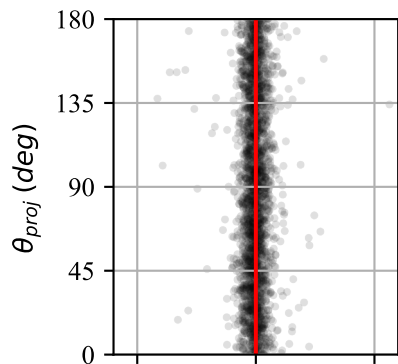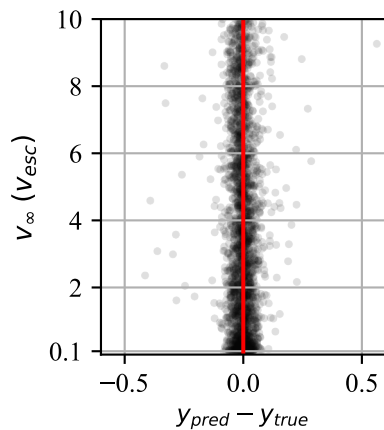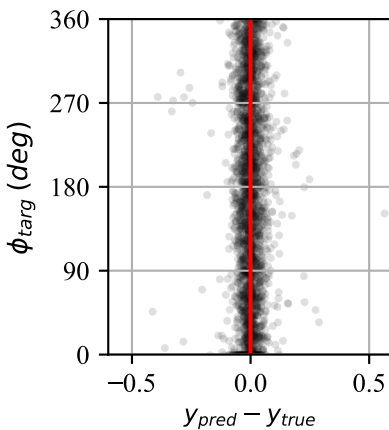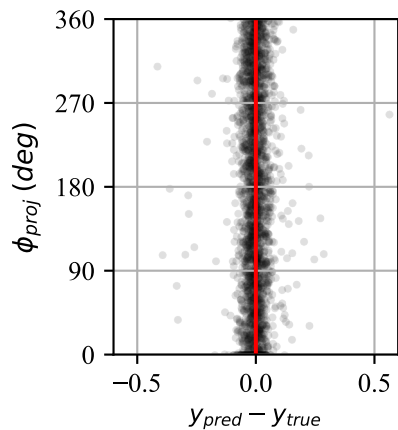

Supplement: Supplementary file 1 — Supplementary information (ZIP 48.3 MB) [file 40668_2020_34_MOESM1_ESM.zip › residuals_lr_core_fraction_mlp_11884.pdf]

Target:  $F_{LR}^{core}$ 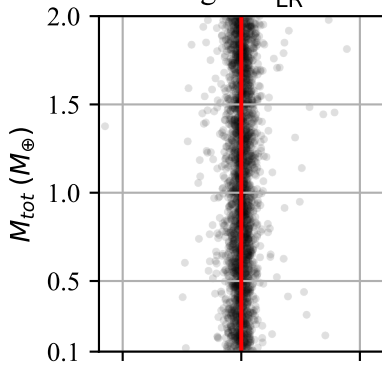

Method: PCE

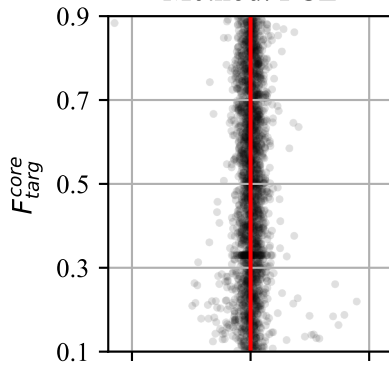

TSS = 11,884

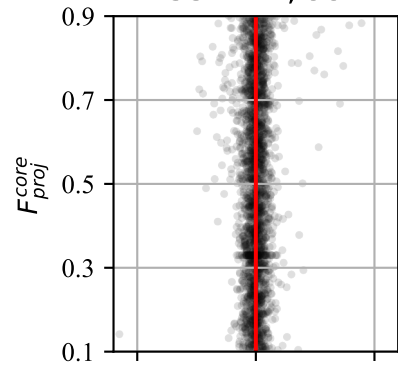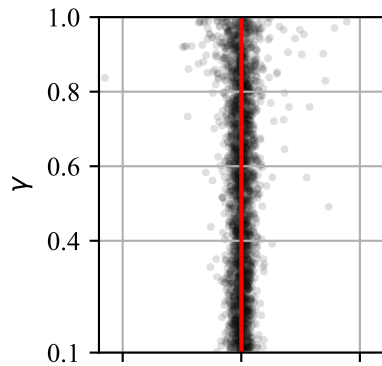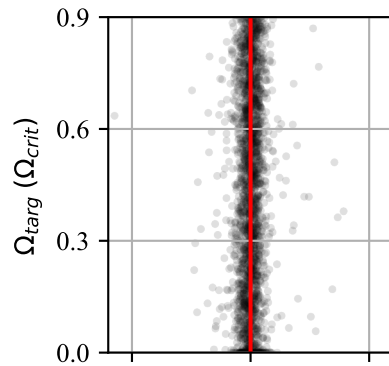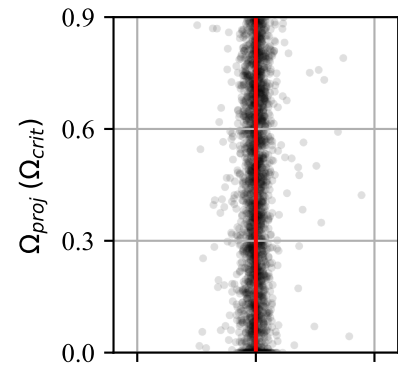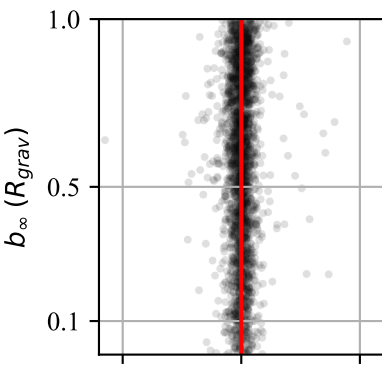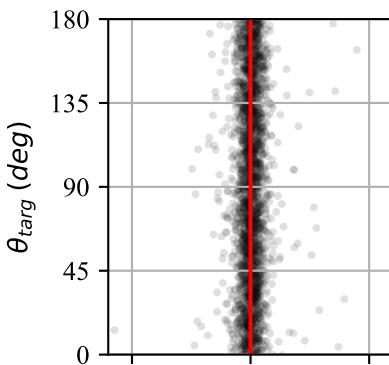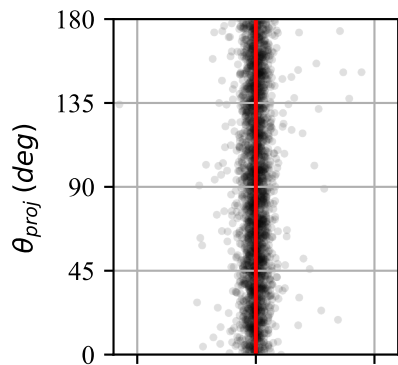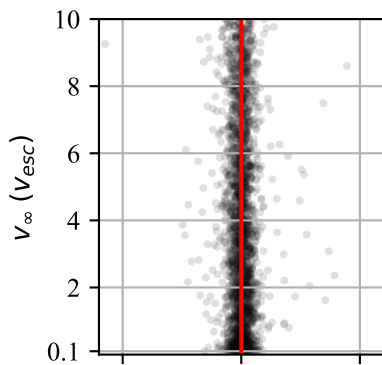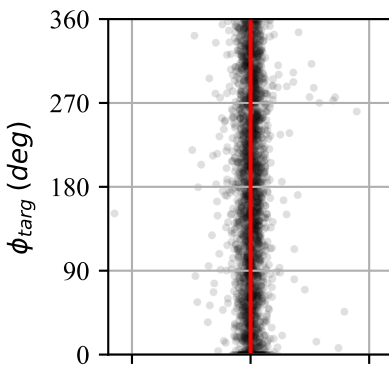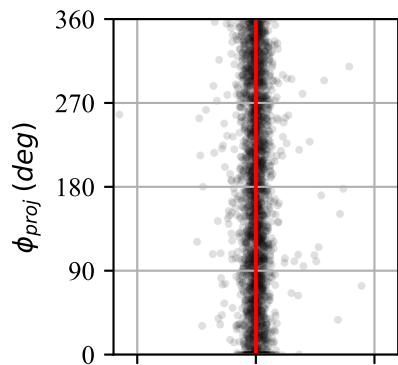 $y_{pred} - y_{true}$  $y_{pred} - y_{true}$  $y_{pred} - y_{true}$

Supplement: Supplementary file 1 — Supplementary information (ZIP 48.3 MB) [file 40668_2020_34_MOESM1_ESM.zip › residuals_lr_core_fraction_pce_11884.pdf]

Target:  $F_{LR}^{core}$ 

Method: XGB

TSS = 11, 884

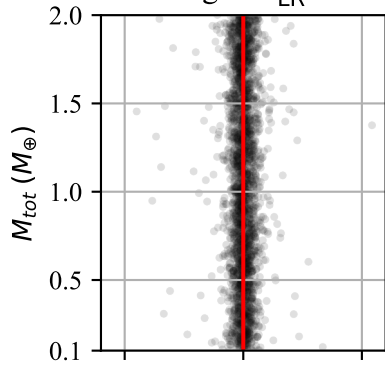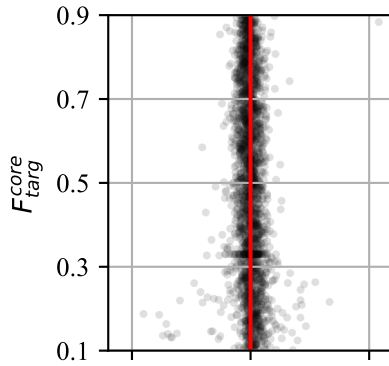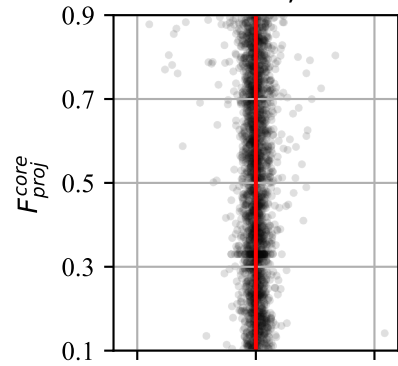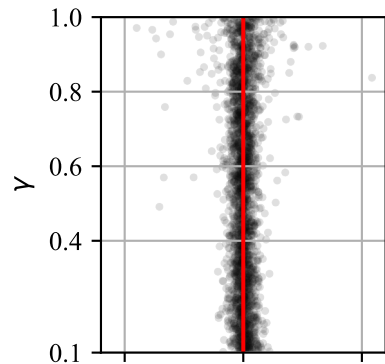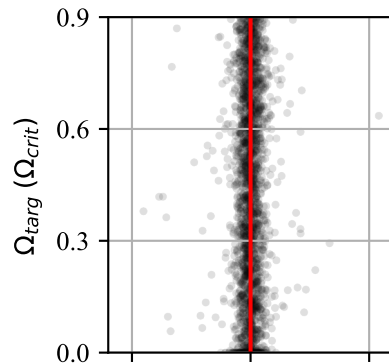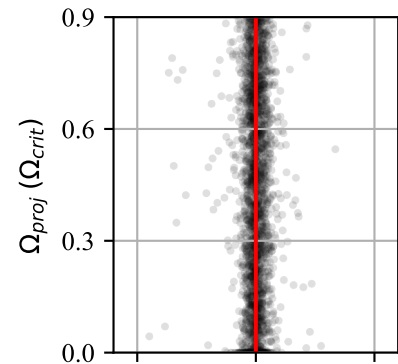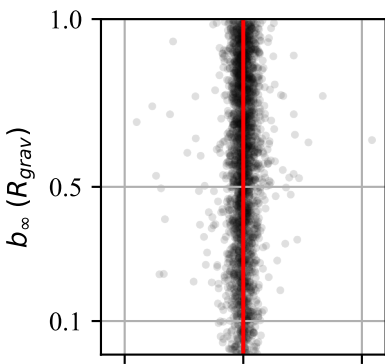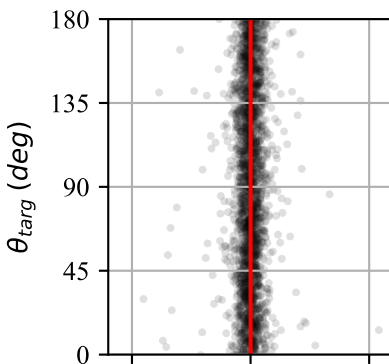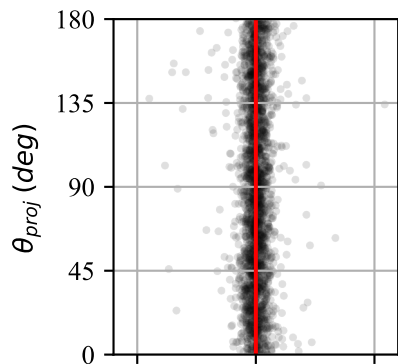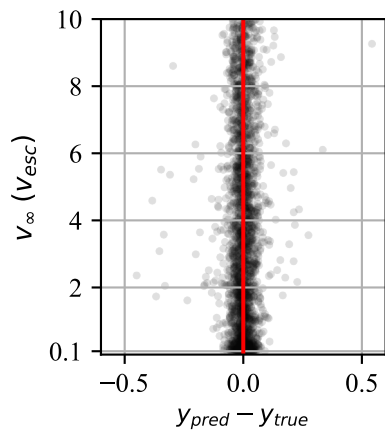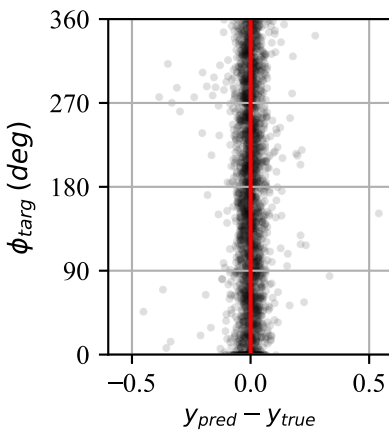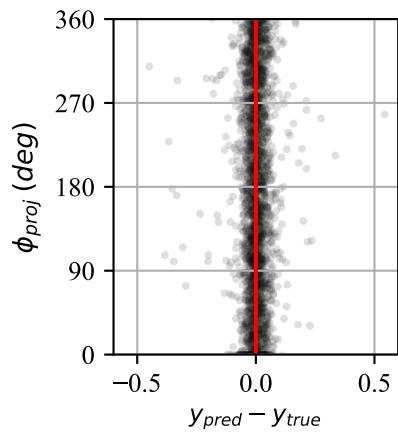

Supplement: Supplementary file 1 — Supplementary information (ZIP 48.3 MB) [file 40668_2020_34_MOESM1_ESM.zip › residuals_lr_core_fraction_xgb_11884.pdf]

Target:  $F_{LR}^{melt}$ 

Method: GP

TSS = 11,884

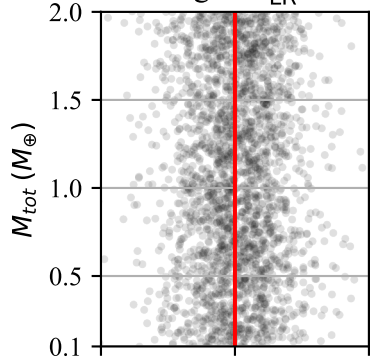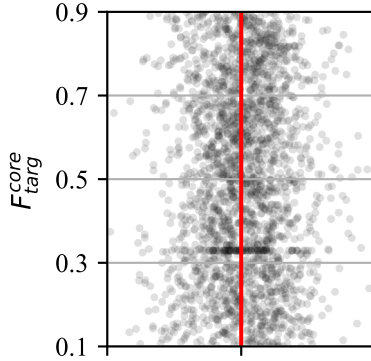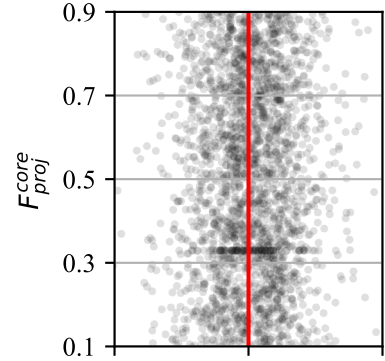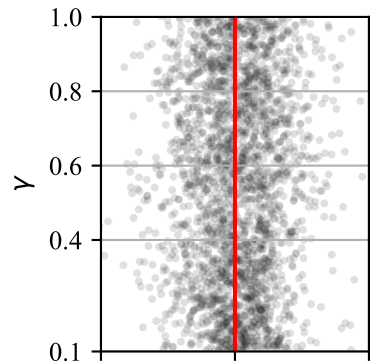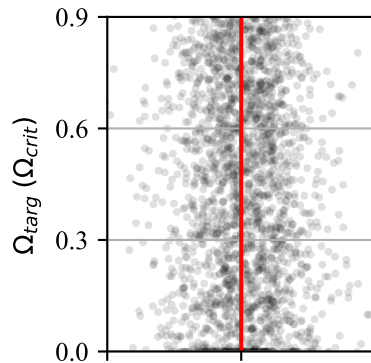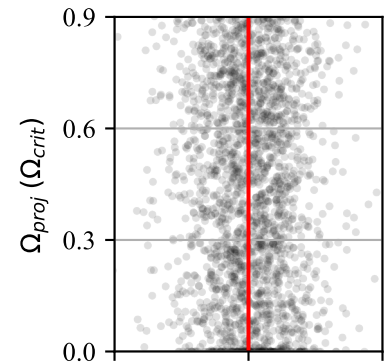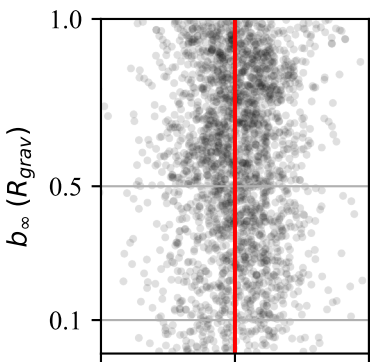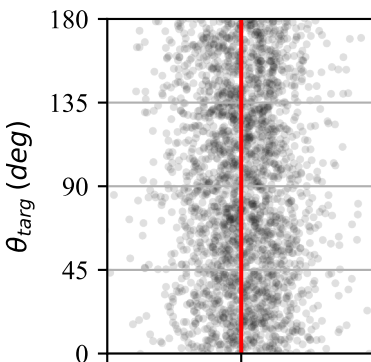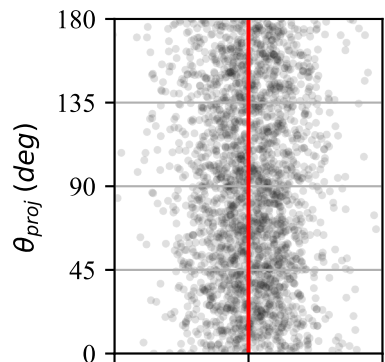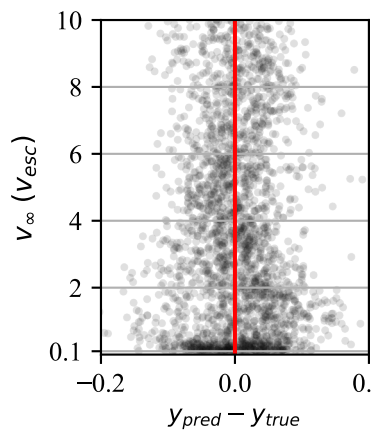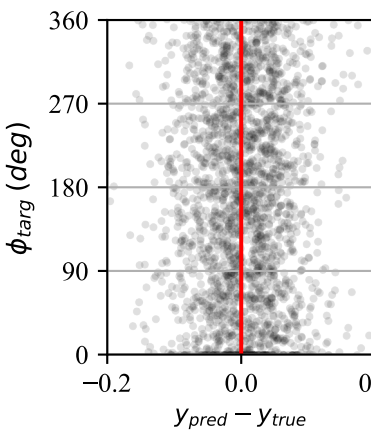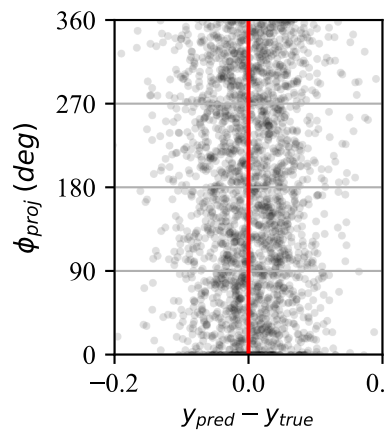

Supplement: Supplementary file 1 — Supplementary information (ZIP 48.3 MB) [file 40668_2020_34_MOESM1_ESM.zip › residuals_lr_expanded_gp_11884.pdf]

Target:  $F_{\text{LR}}^{\text{melt}}$ 

Method: XGB

TSS = 11,884

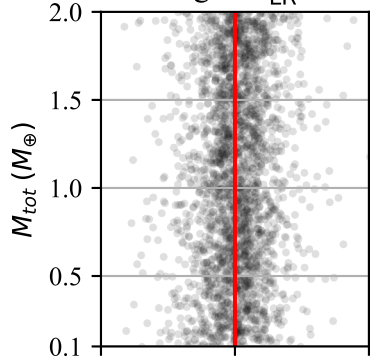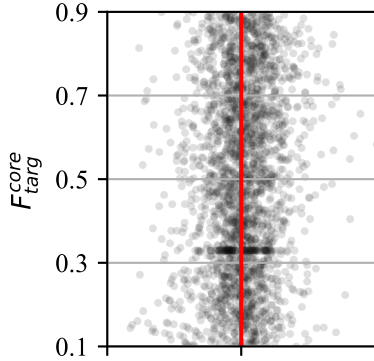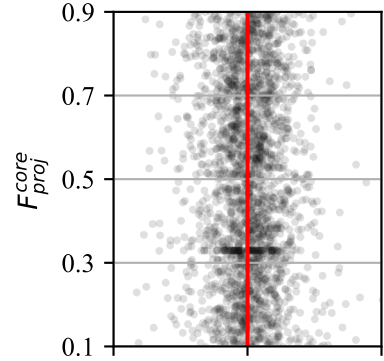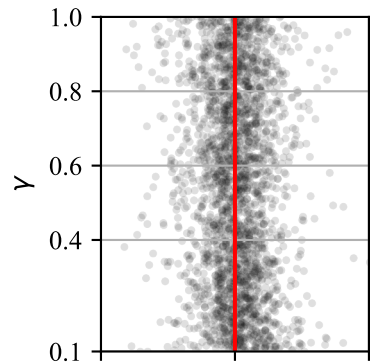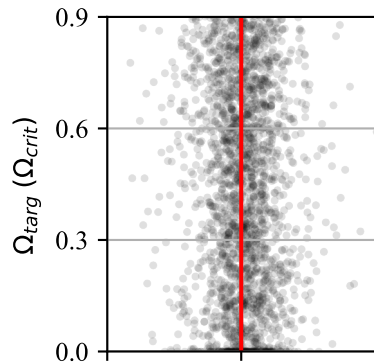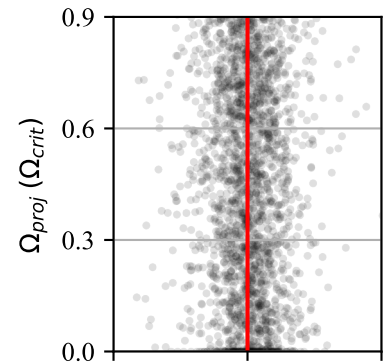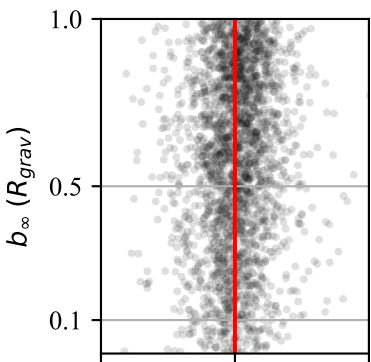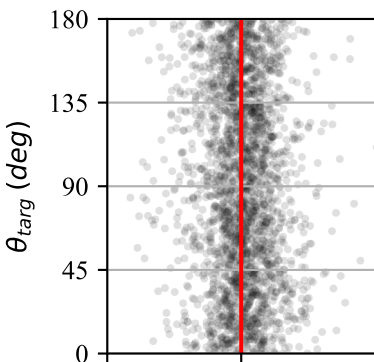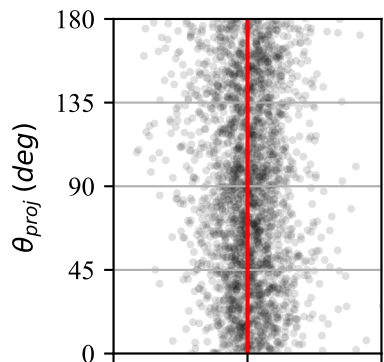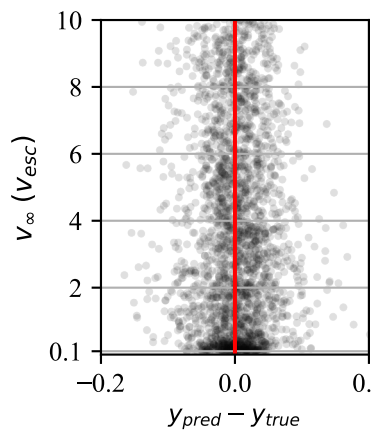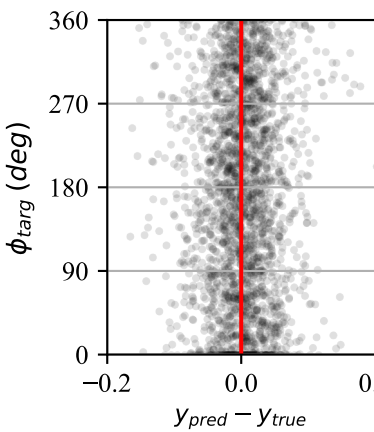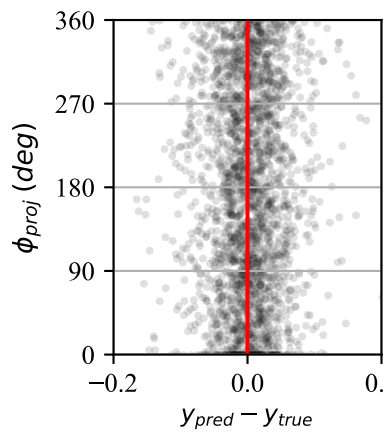

Supplement: Supplementary file 1 — Supplementary information (ZIP 48.3 MB) [file 40668_2020_34_MOESM1_ESM.zip › residuals_lr_expanded_xgb_11884.pdf]

Target:  $M_{LR}$ 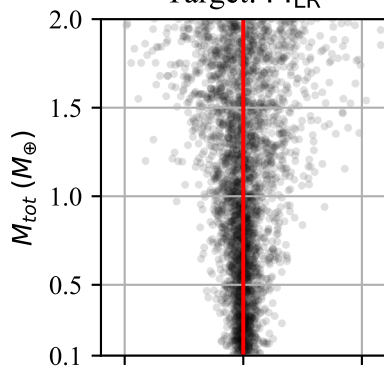

Method: GP

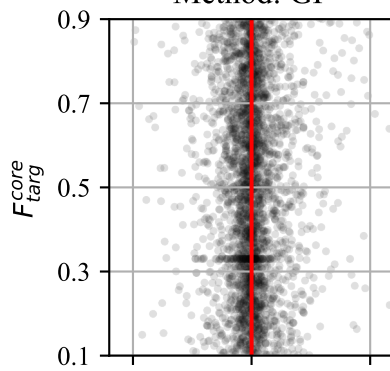

TSS = 11,884

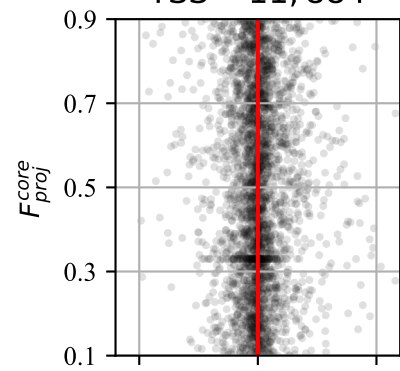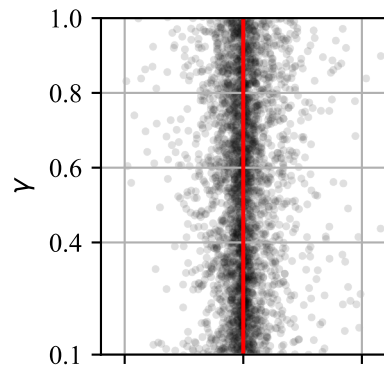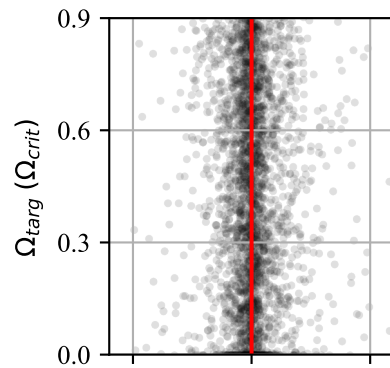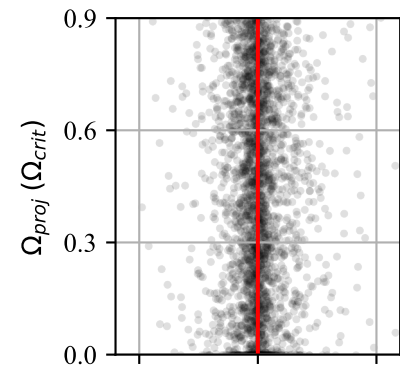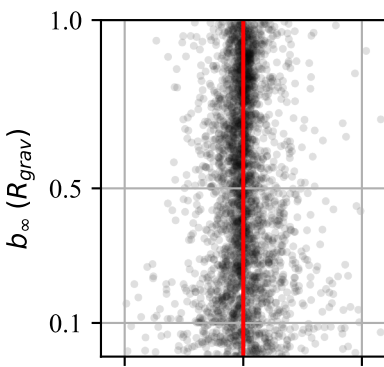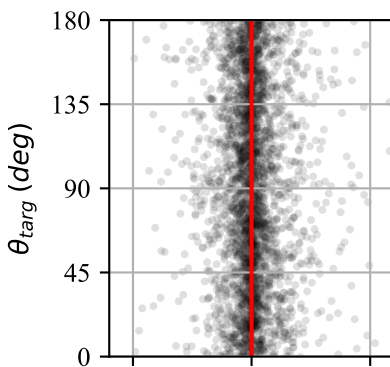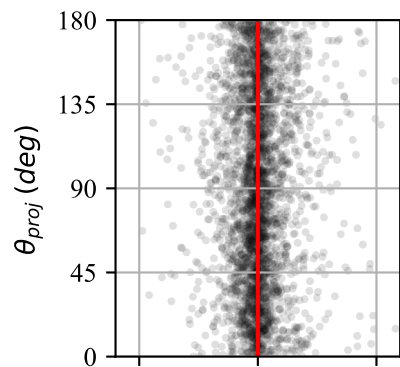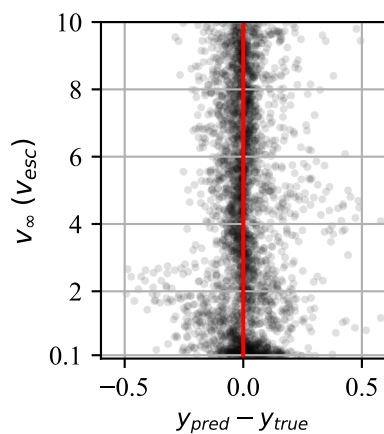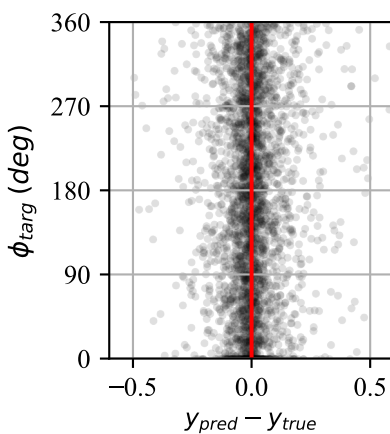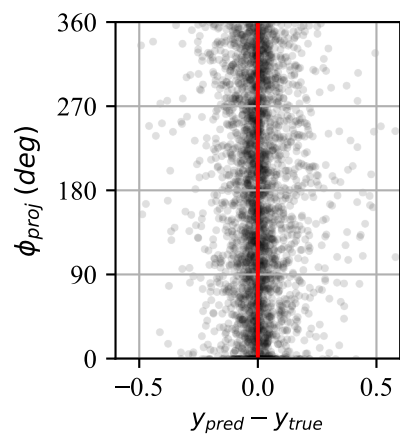

Supplement: Supplementary file 1 — Supplementary information (ZIP 48.3 MB) [file 40668_2020_34_MOESM1_ESM.zip › residuals_lr_mass_gp_11884.pdf]

Target:  $M_{\text{LR}}$ 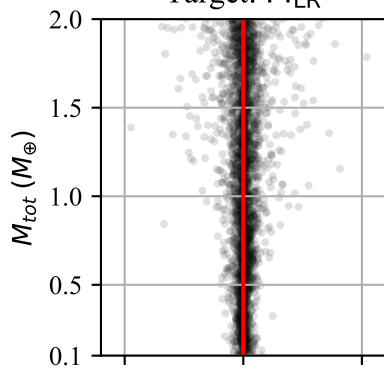

Method: MLP

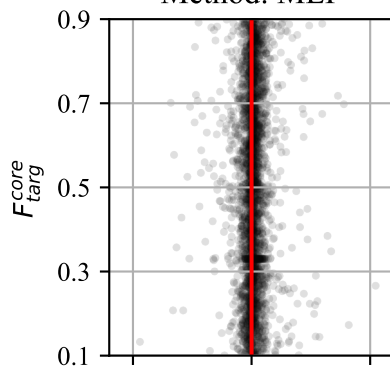

TSS = 11,884

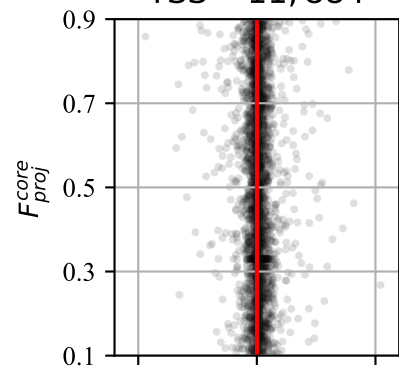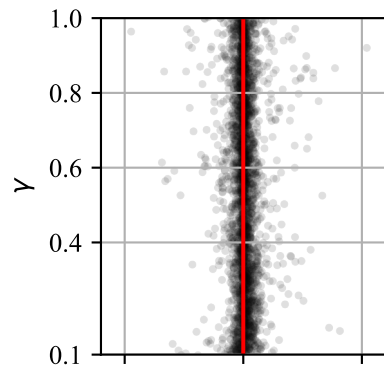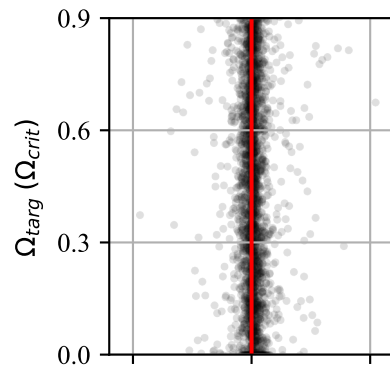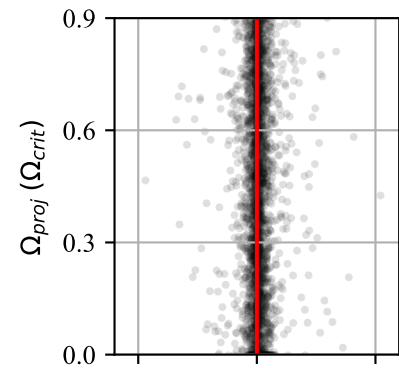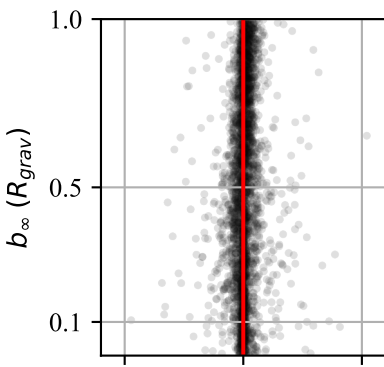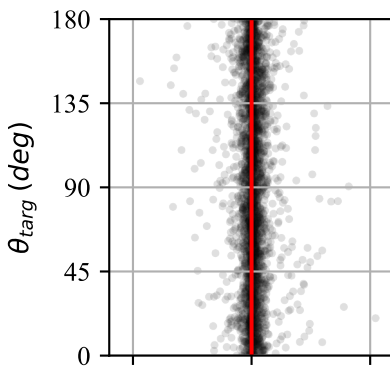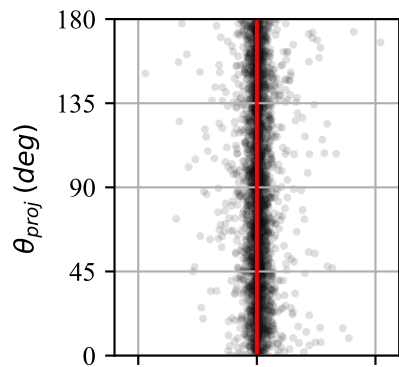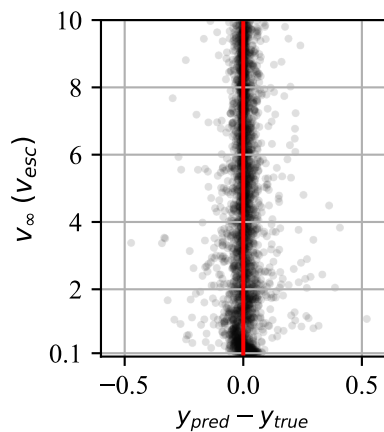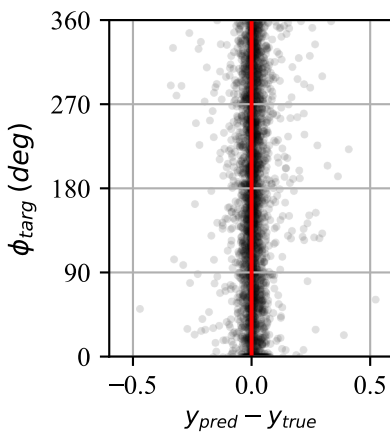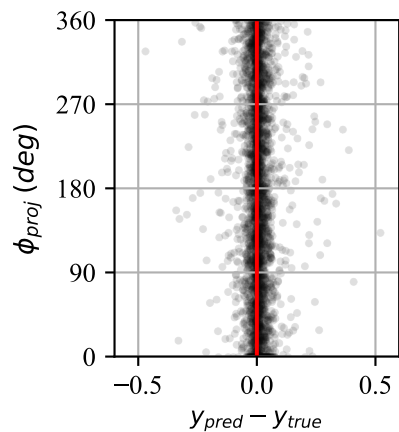

Supplement: Supplementary file 1 — Supplementary information (ZIP 48.3 MB) [file 40668_2020_34_MOESM1_ESM.zip › residuals_lr_mass_mlp_11884.pdf]

Target:  $M_{\text{LR}}^{\text{norm}}$ 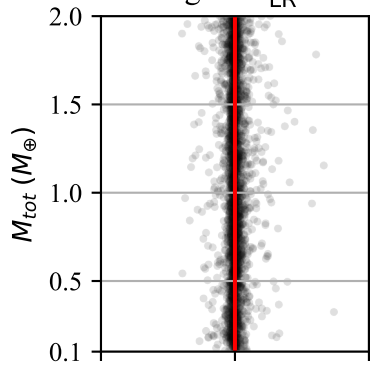

Method: MLP

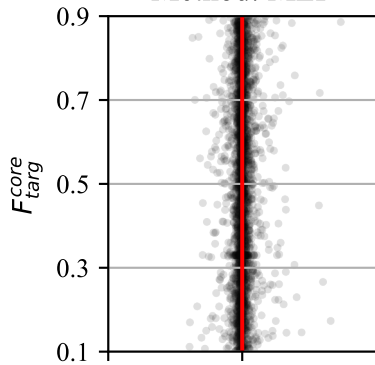

TSS = 11,884

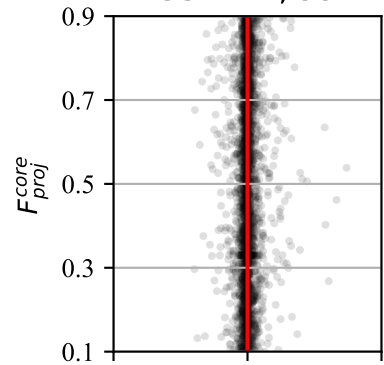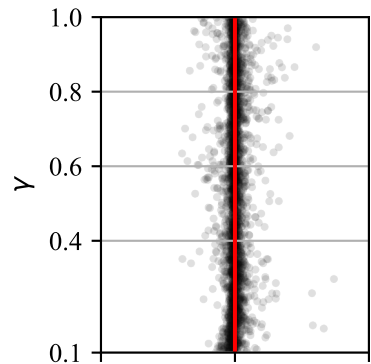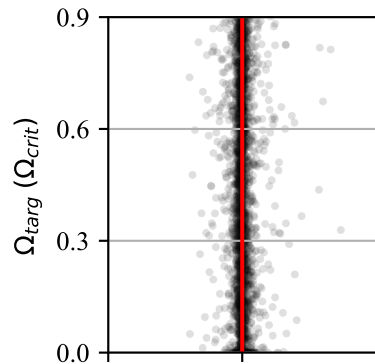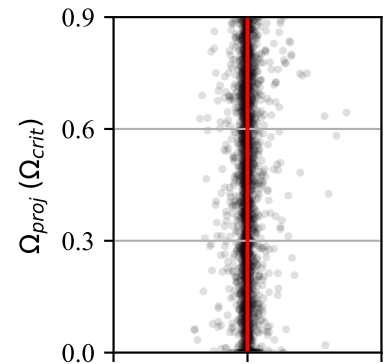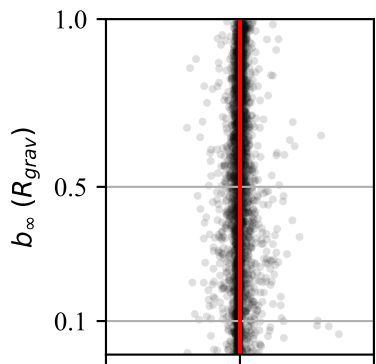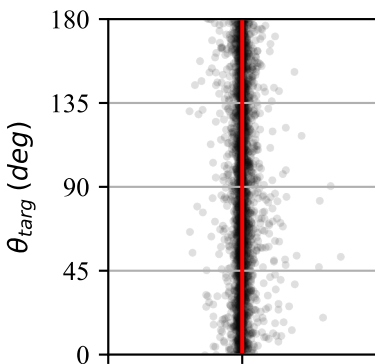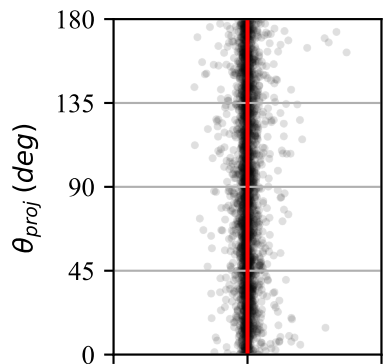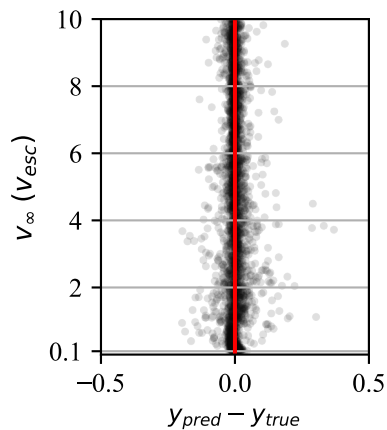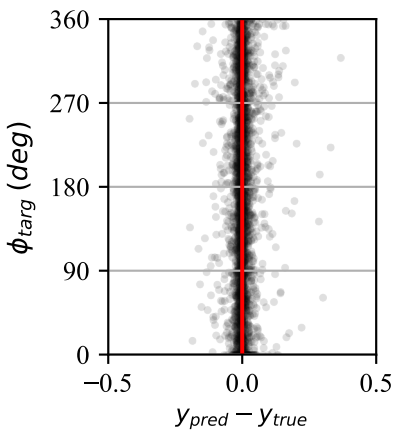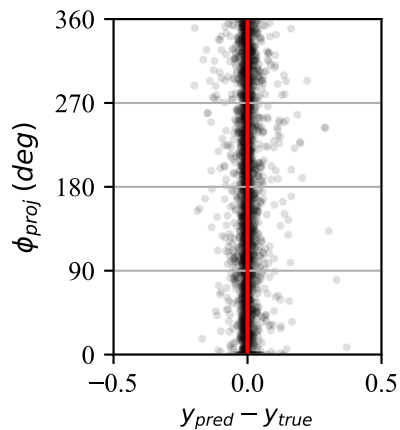

Supplement: Supplementary file 1 — Supplementary information (ZIP 48.3 MB) [file 40668_2020_34_MOESM1_ESM.zip › residuals_lr_mass_norm_mlp_11884.pdf]

Target:  $M_{\text{LR}}^{\text{norm}}$ 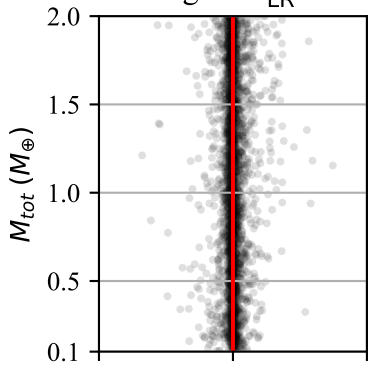

Method: XGB

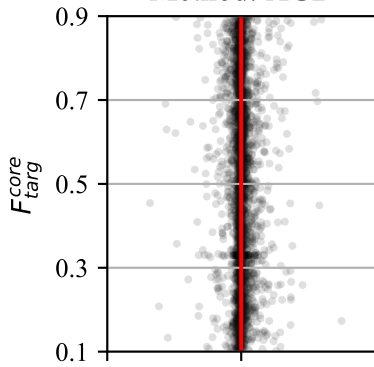

TSS = 11, 884

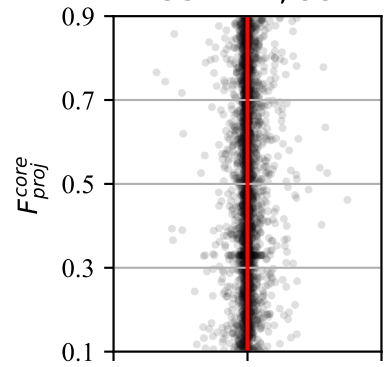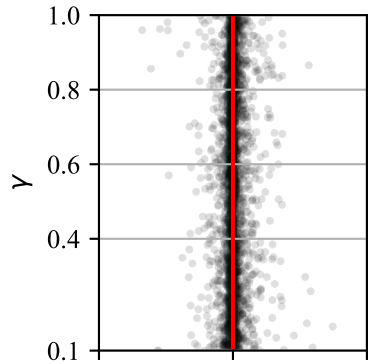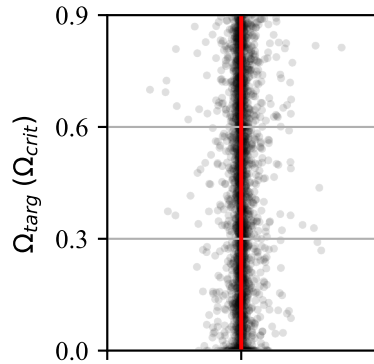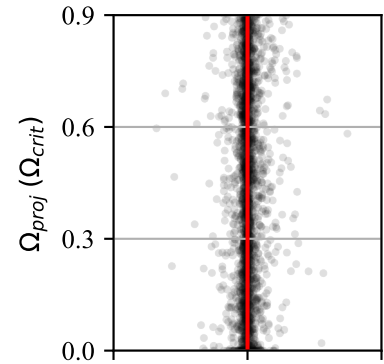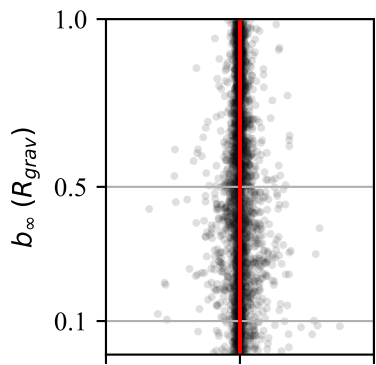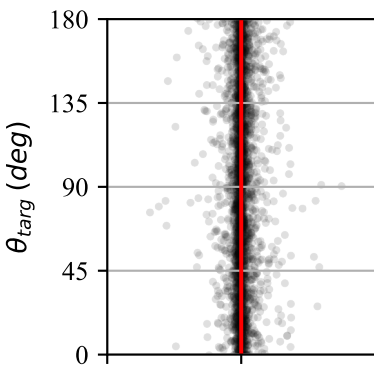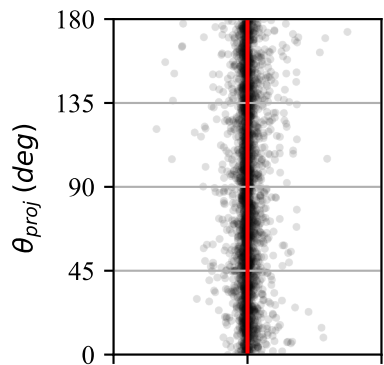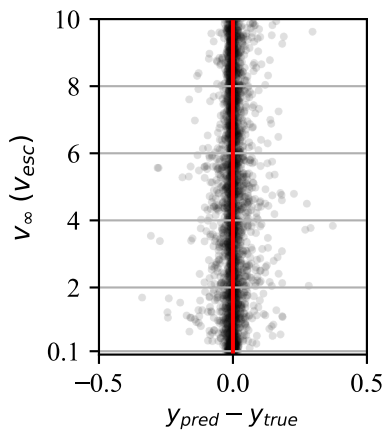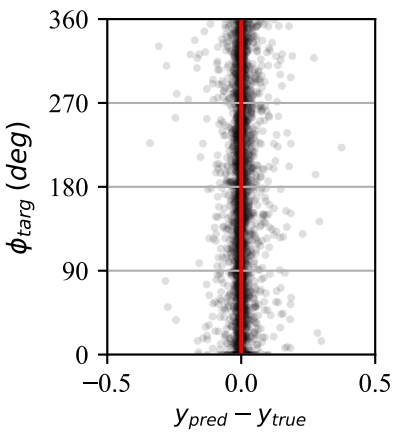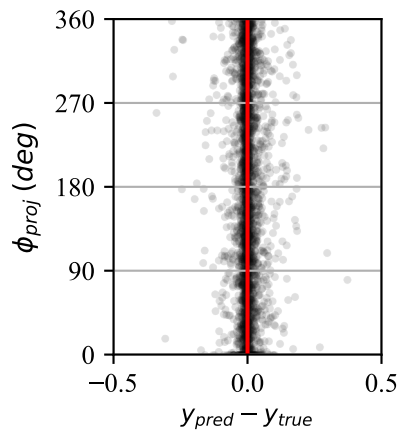

Supplement: Supplementary file 1 — Supplementary information (ZIP 48.3 MB) [file 40668_2020_34_MOESM1_ESM.zip › residuals_lr_mass_norm_xgb_11884.pdf]

Target:  $M_{\text{LR}}$ 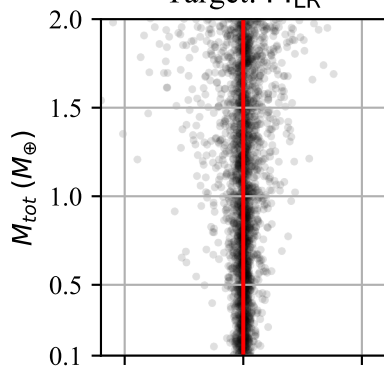

Method: PCE

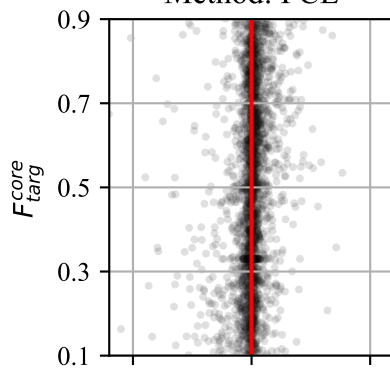

TSS = 11,884

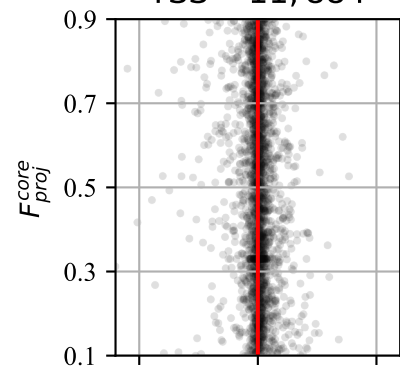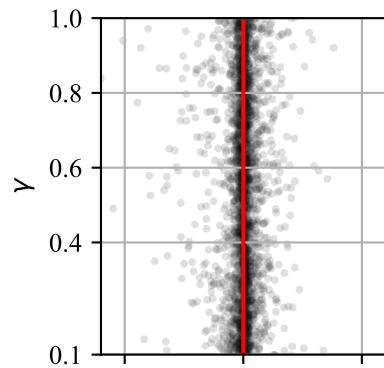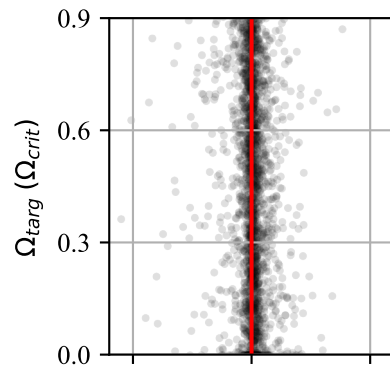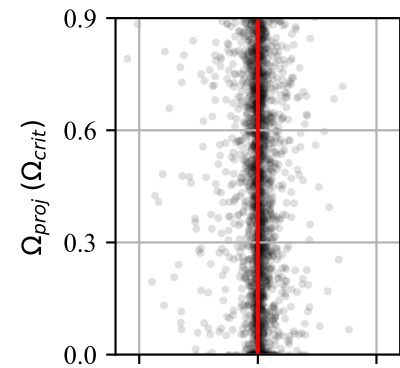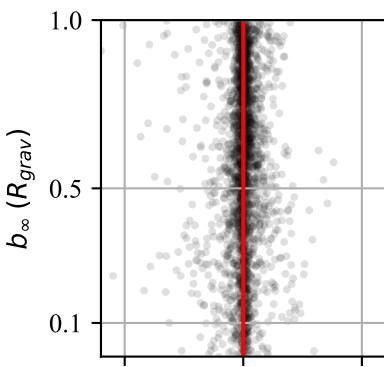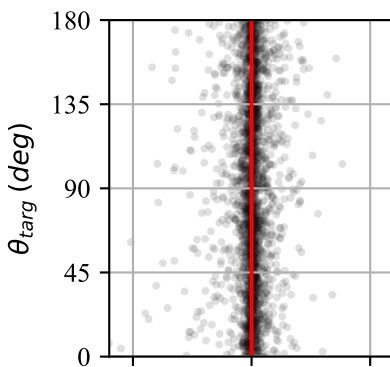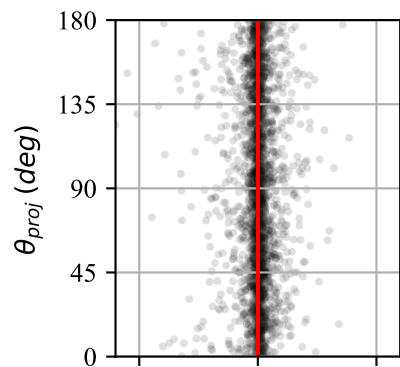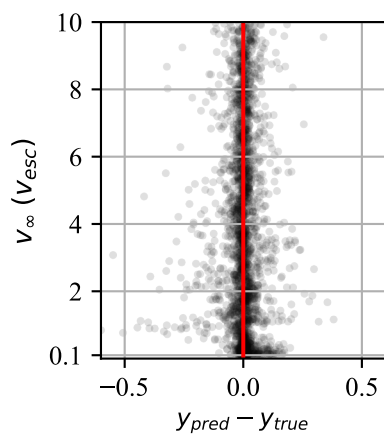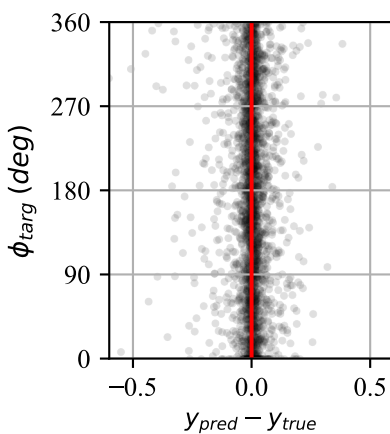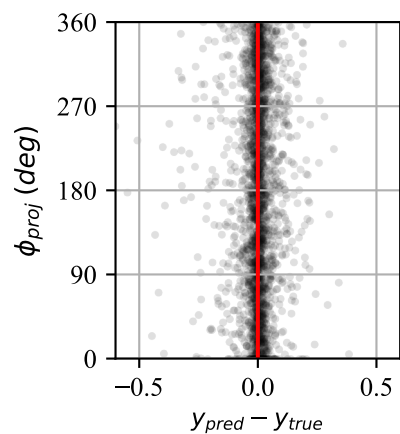

Supplement: Supplementary file 1 — Supplementary information (ZIP 48.3 MB) [file 40668_2020_34_MOESM1_ESM.zip › residuals_lr_mass_pce_11884.pdf]

Target:  $M_{\text{LR}}$ 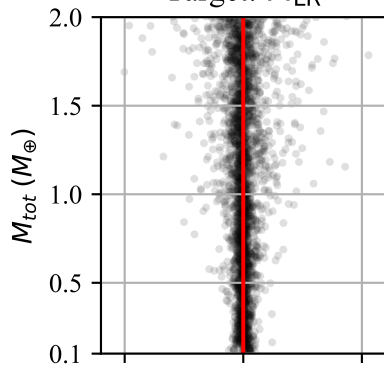

Method: XGB

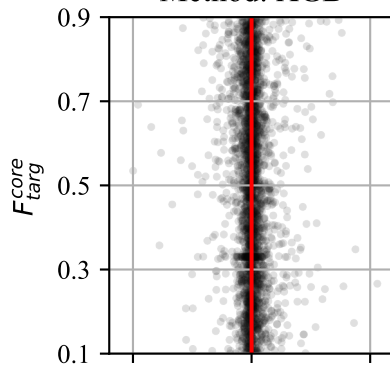

TSS = 11, 884

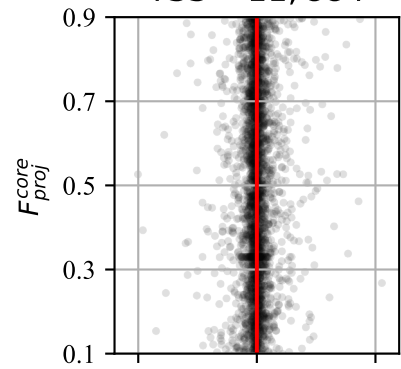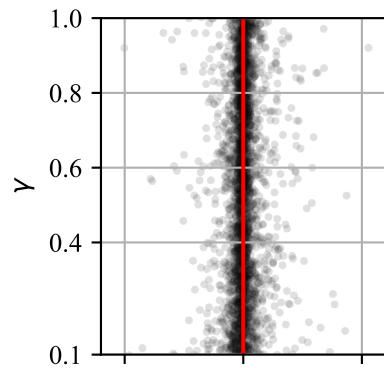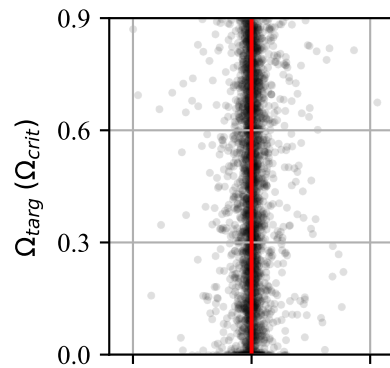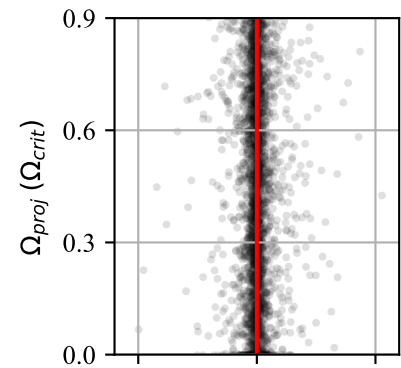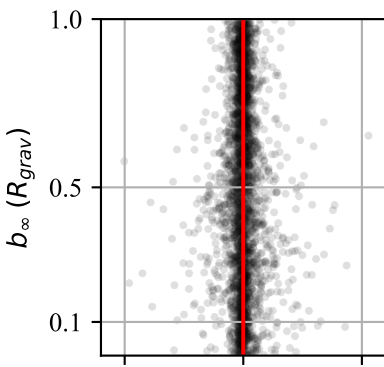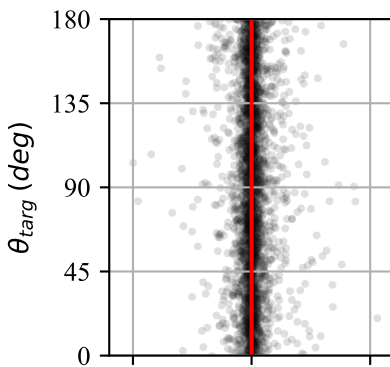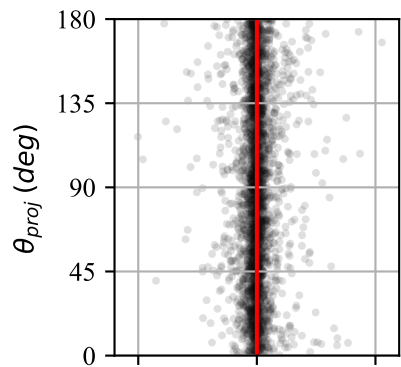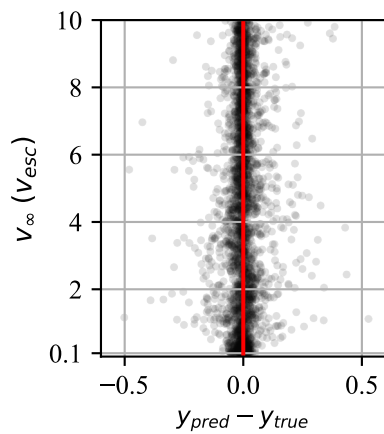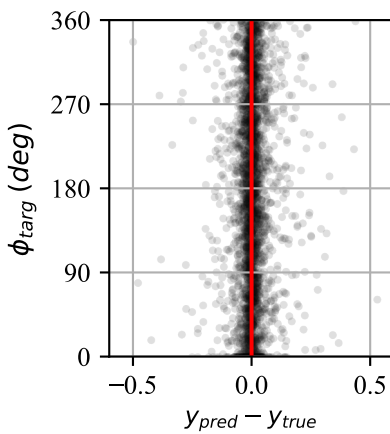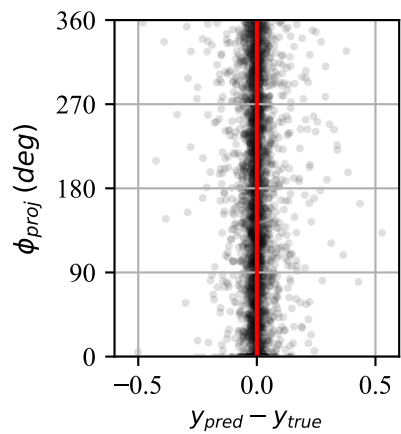

Supplement: Supplementary file 1 — Supplementary information (ZIP 48.3 MB) [file 40668_2020_34_MOESM1_ESM.zip › residuals_lr_mass_xgb_11884.pdf]

Target:  $\delta_{\text{LR}}^{\text{mix}}$ 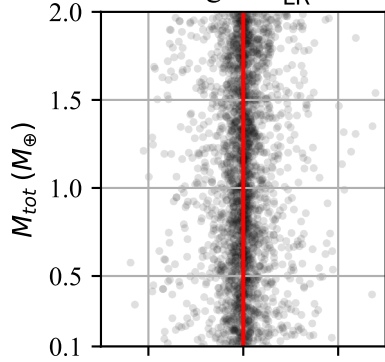

Method: GP

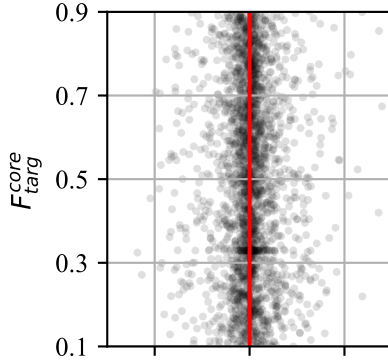

TSS = 11,884

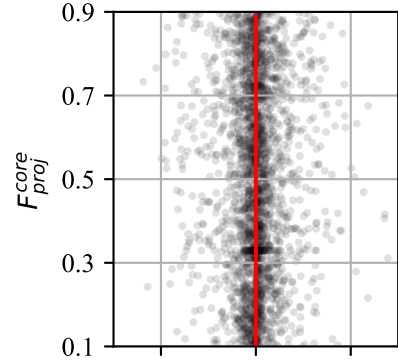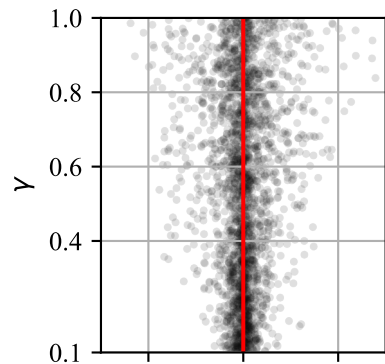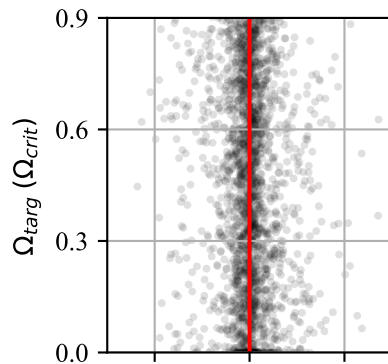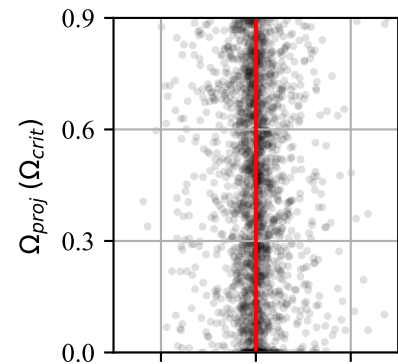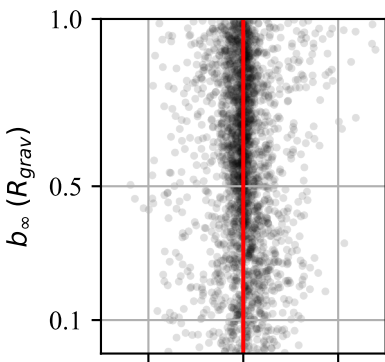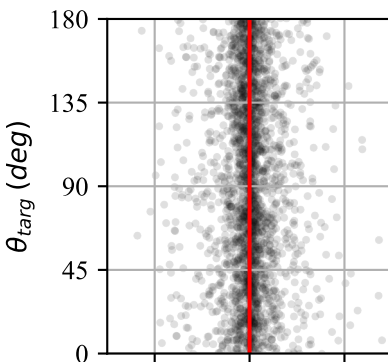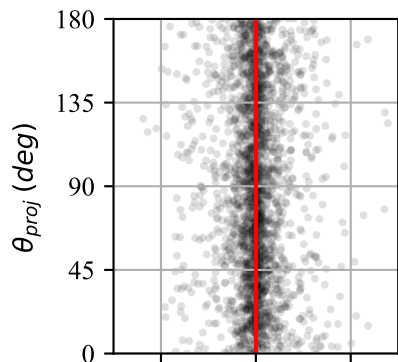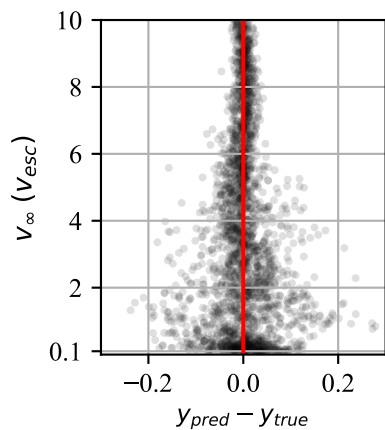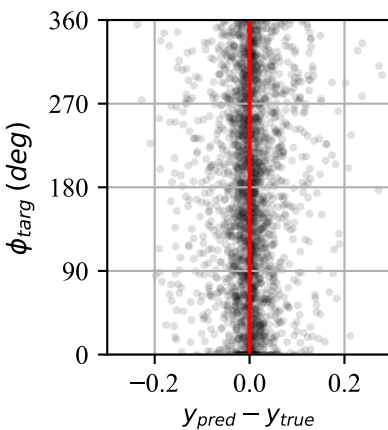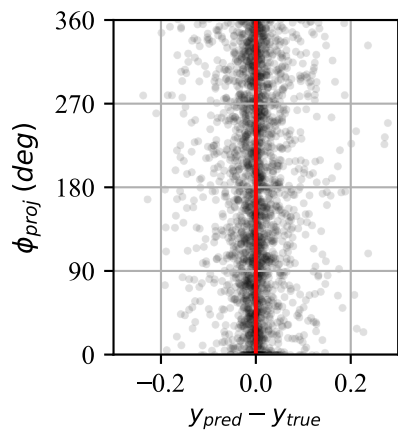

Supplement: Supplementary file 1 — Supplementary information (ZIP 48.3 MB) [file 40668_2020_34_MOESM1_ESM.zip › residuals_lr_mixing_impurity_gp_11884.pdf]

Target:  $\delta_{\text{LR}}^{\text{mix}}$ 

Method: MLP

TSS = 11,884

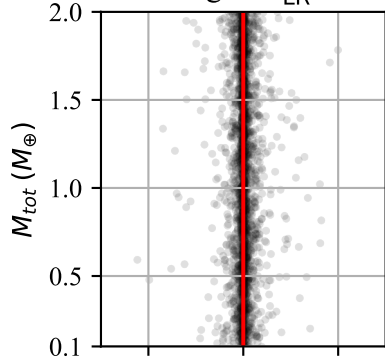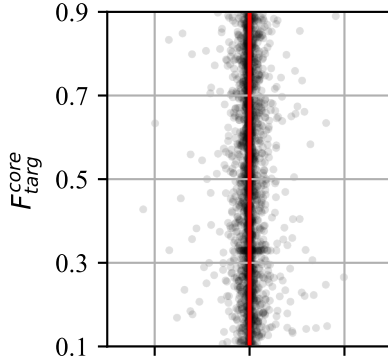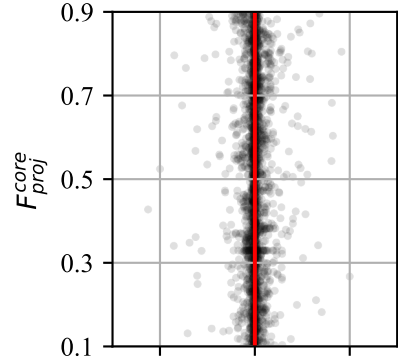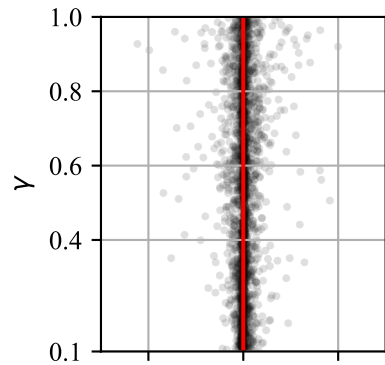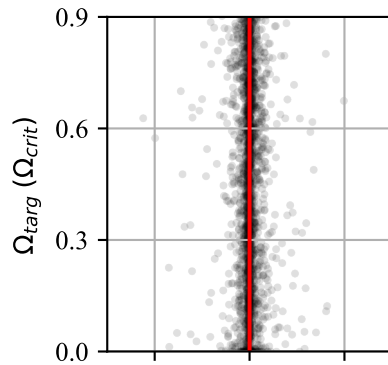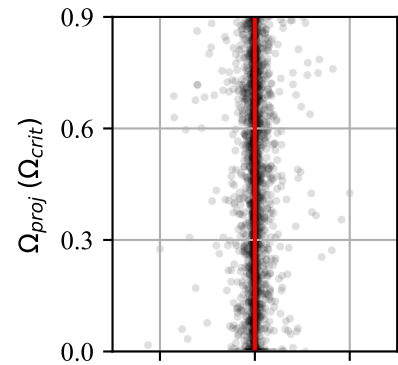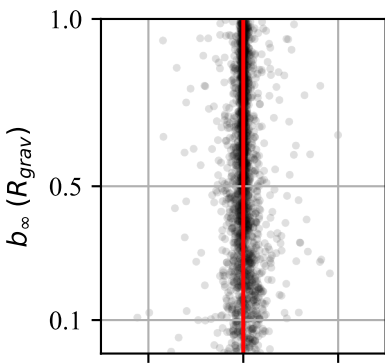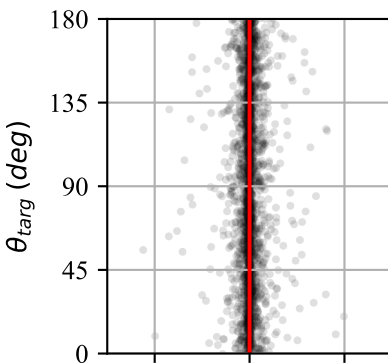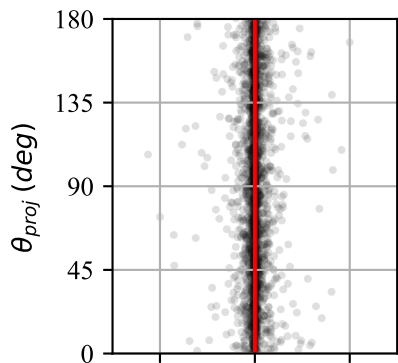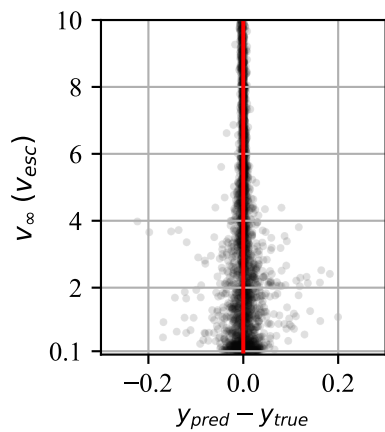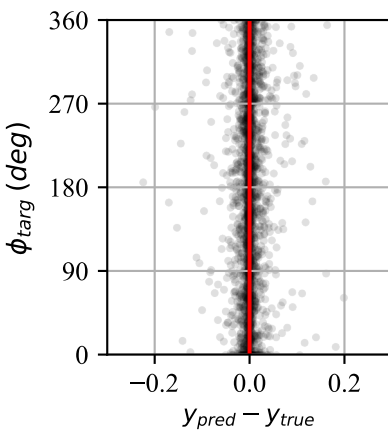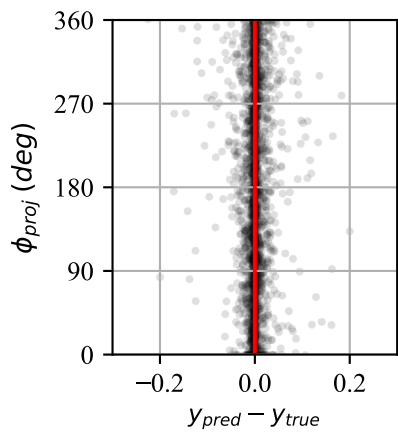

Supplement: Supplementary file 1 — Supplementary information (ZIP 48.3 MB) [file 40668_2020_34_MOESM1_ESM.zip › residuals_lr_mixing_impurity_mlp_11884.pdf]

Target:  $\delta_{\text{LR}}^{\text{mix}}$ 

Method: PCE

TSS = 11,884

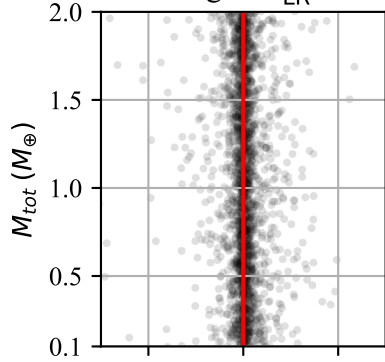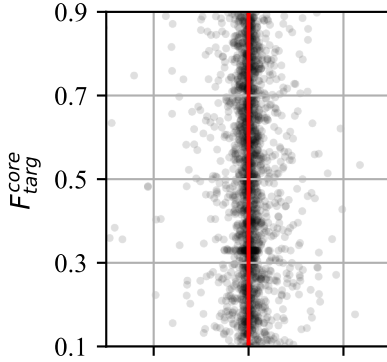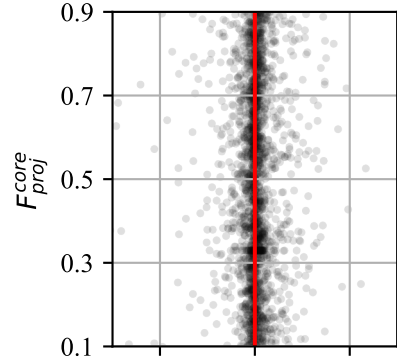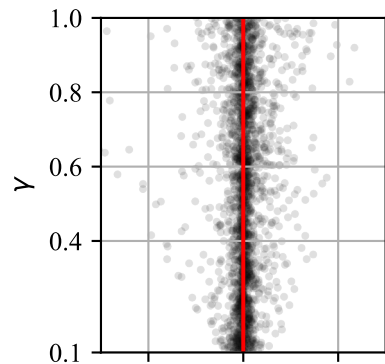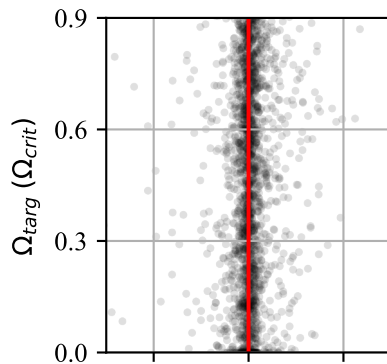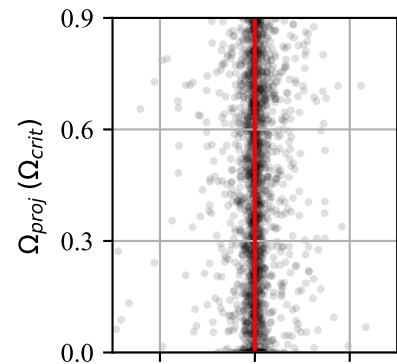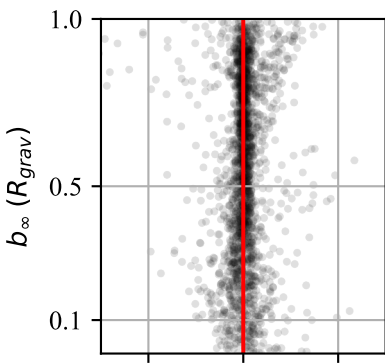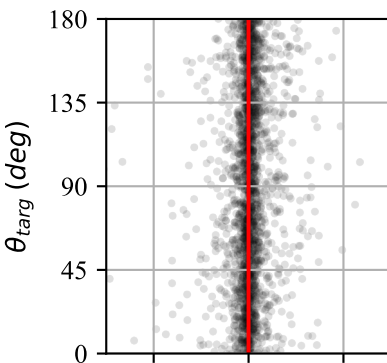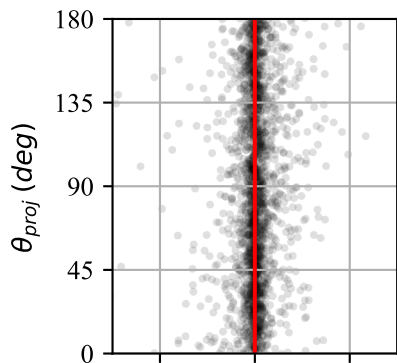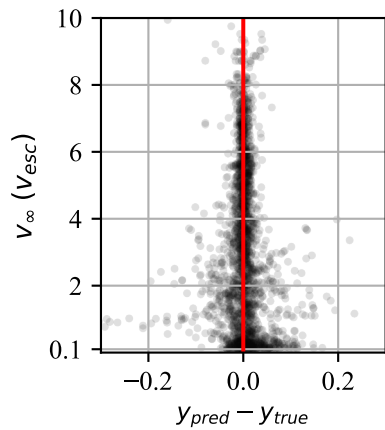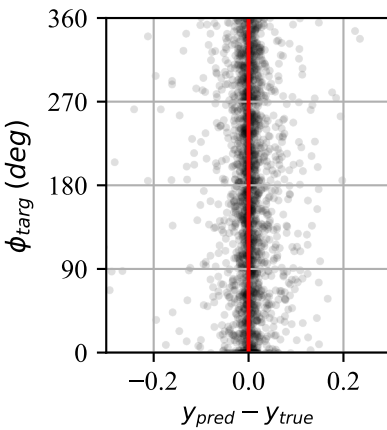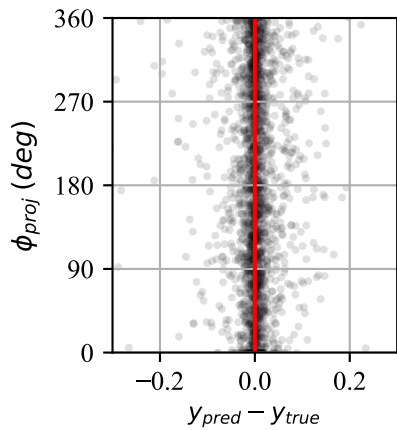

Supplement: Supplementary file 1 — Supplementary information (ZIP 48.3 MB) [file 40668_2020_34_MOESM1_ESM.zip › residuals_lr_mixing_impurity_pce_11884.pdf]

Target:  $\delta_{\text{LR}}^{\text{mix}}$ 

Method: XGB

TSS = 11,884

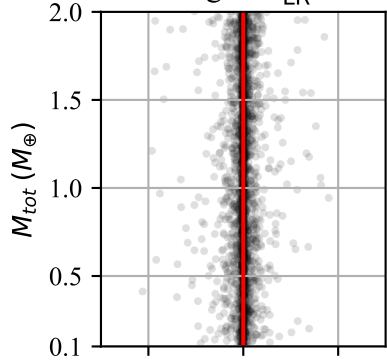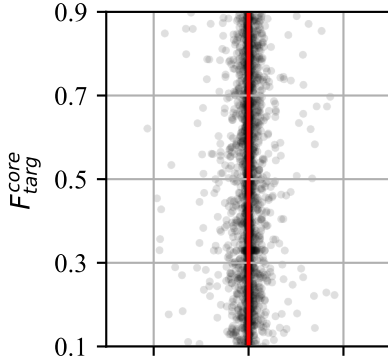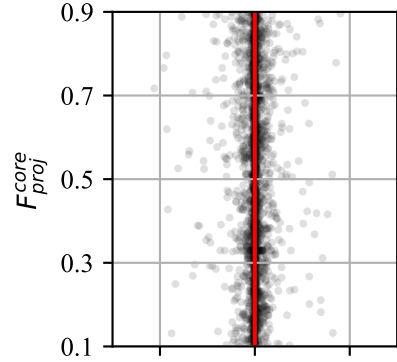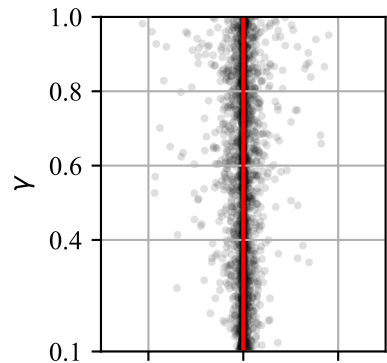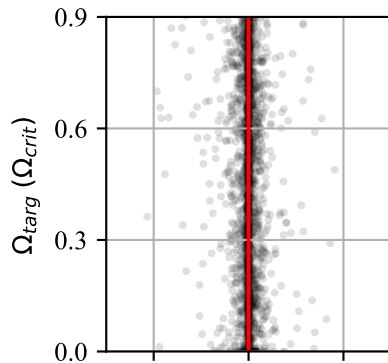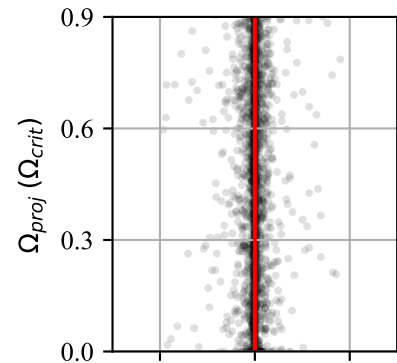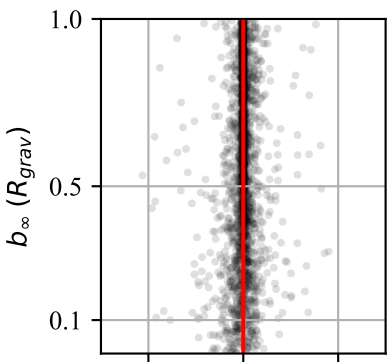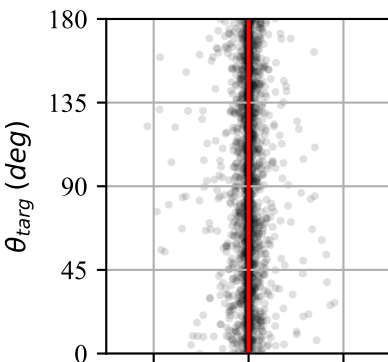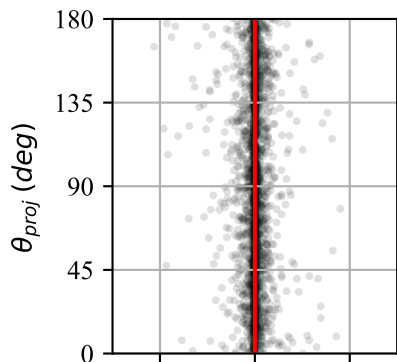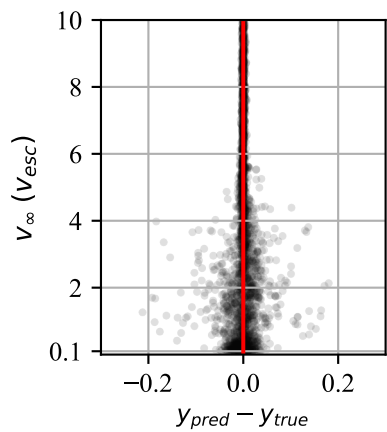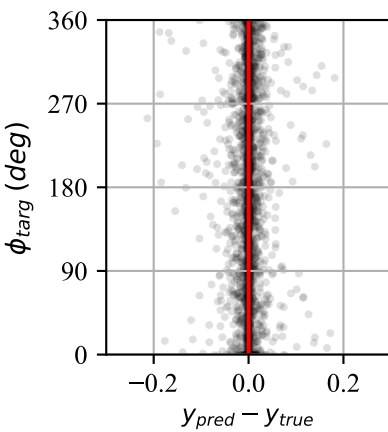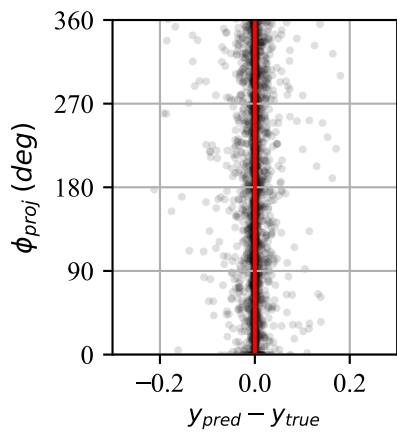

Supplement: Supplementary file 1 — Supplementary information (ZIP 48.3 MB) [file 40668_2020_34_MOESM1_ESM.zip › residuals_lr_mixing_impurity_xgb_11884.pdf]

Target:  $\Omega_{\text{LR}}$ 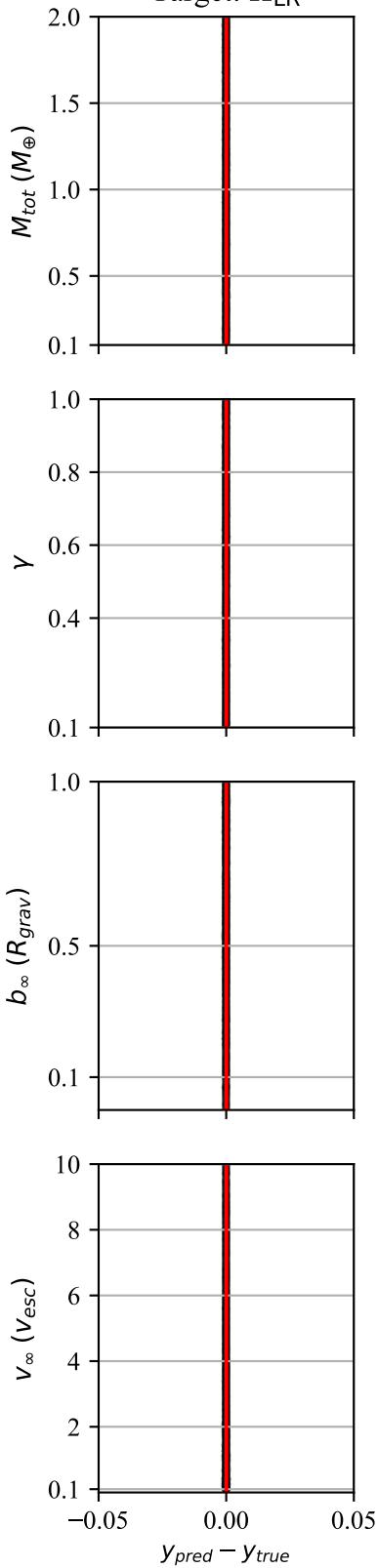

Method: GP

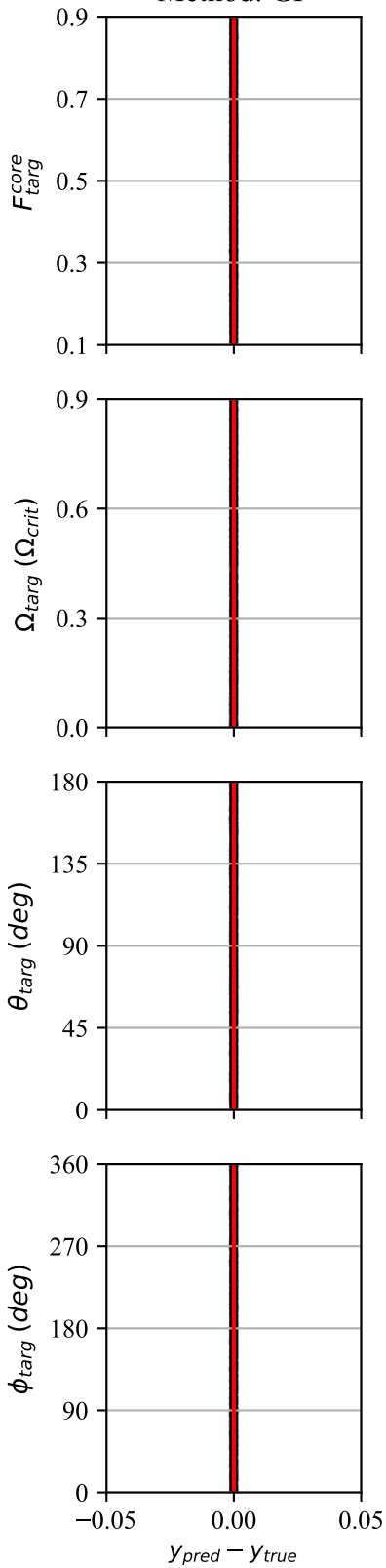

TSS = 11, 884

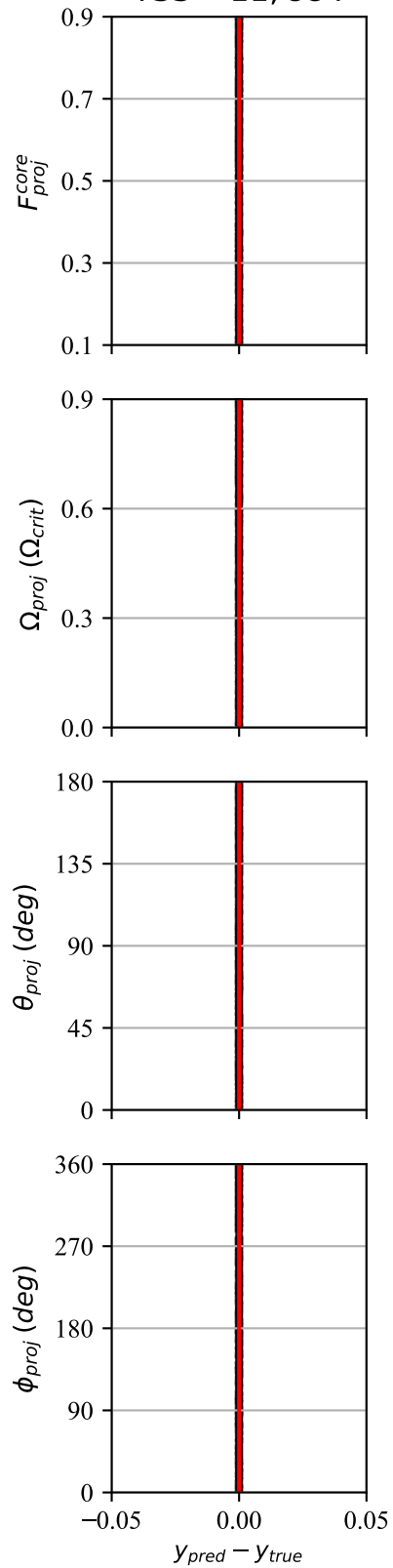

Supplement: Supplementary file 1 — Supplementary information (ZIP 48.3 MB) [file 40668_2020_34_MOESM1_ESM.zip › residuals_lr_omega_gp_11884.pdf]

Target:  $\Omega_{\text{LR}}$ 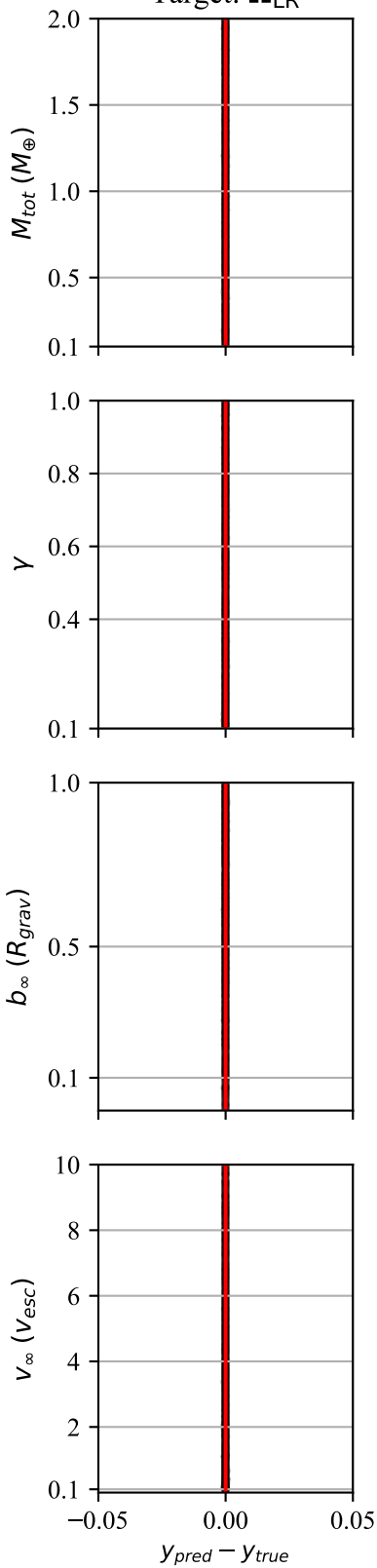

Method: MLP

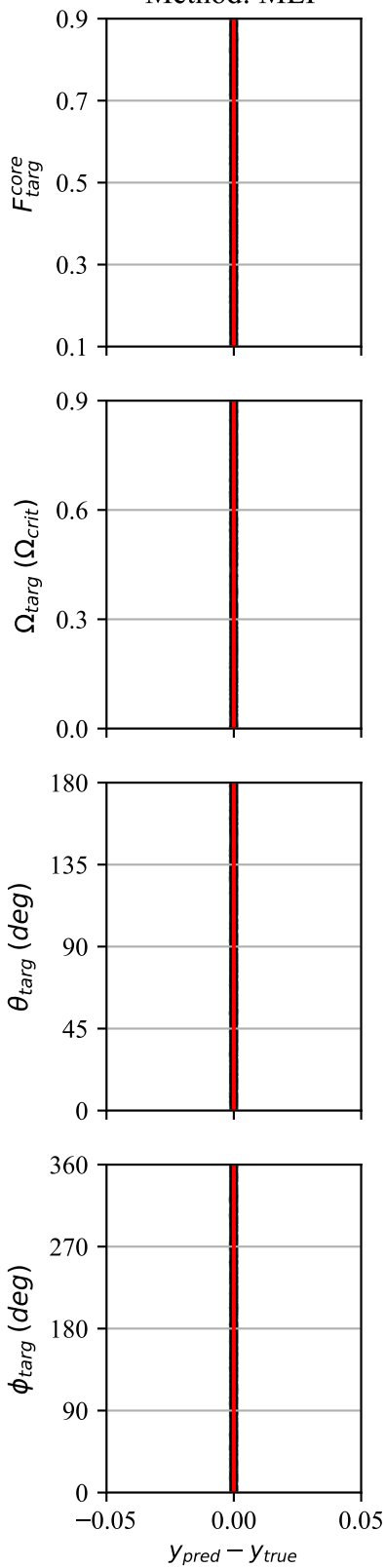

TSS = 11, 884

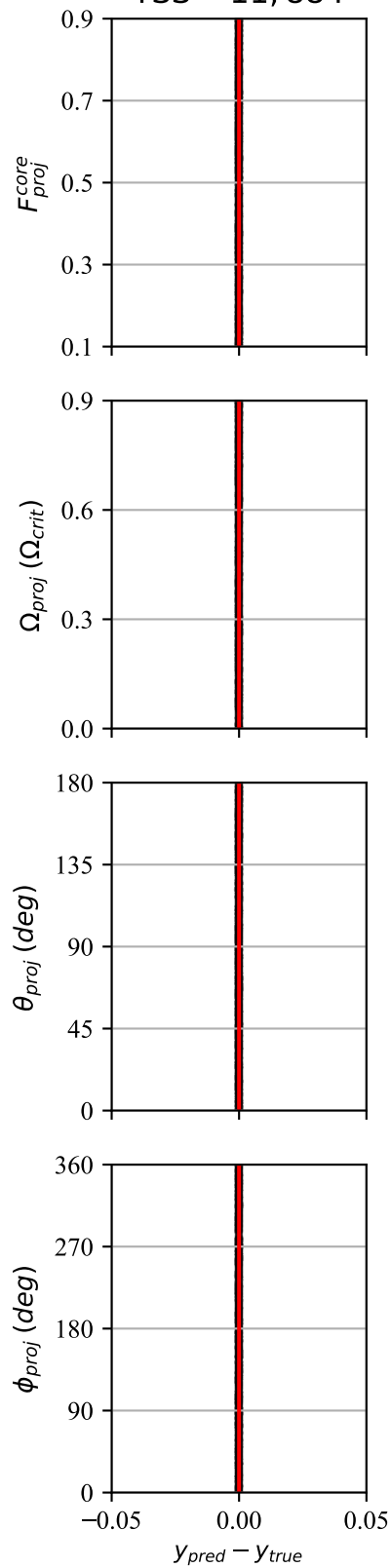

Supplement: Supplementary file 1 — Supplementary information (ZIP 48.3 MB) [file 40668_2020_34_MOESM1_ESM.zip › residuals_lr_omega_mlp_11884.pdf]

Target:  $\Omega_{\text{LR}}$ 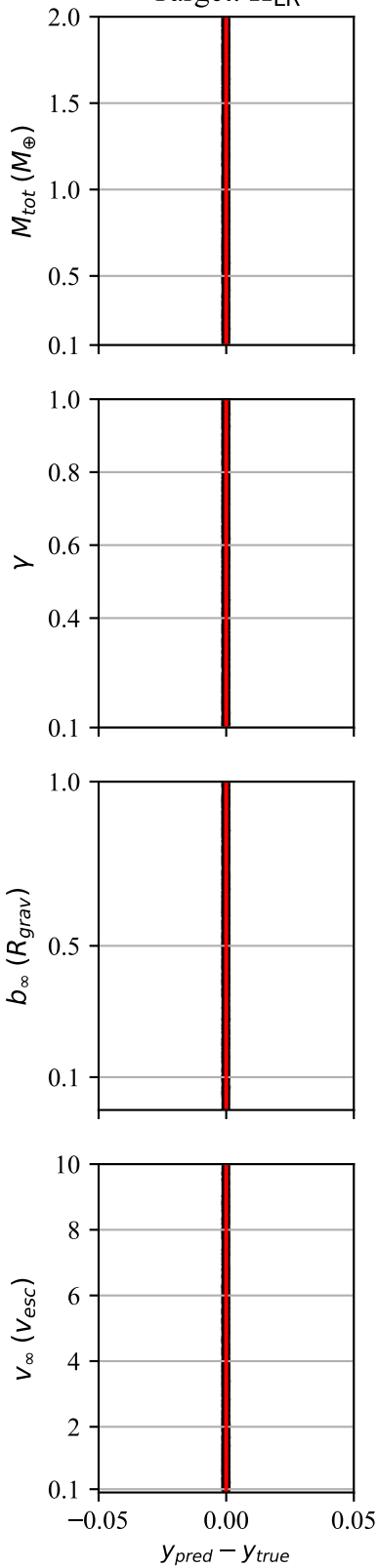

Method: PCE

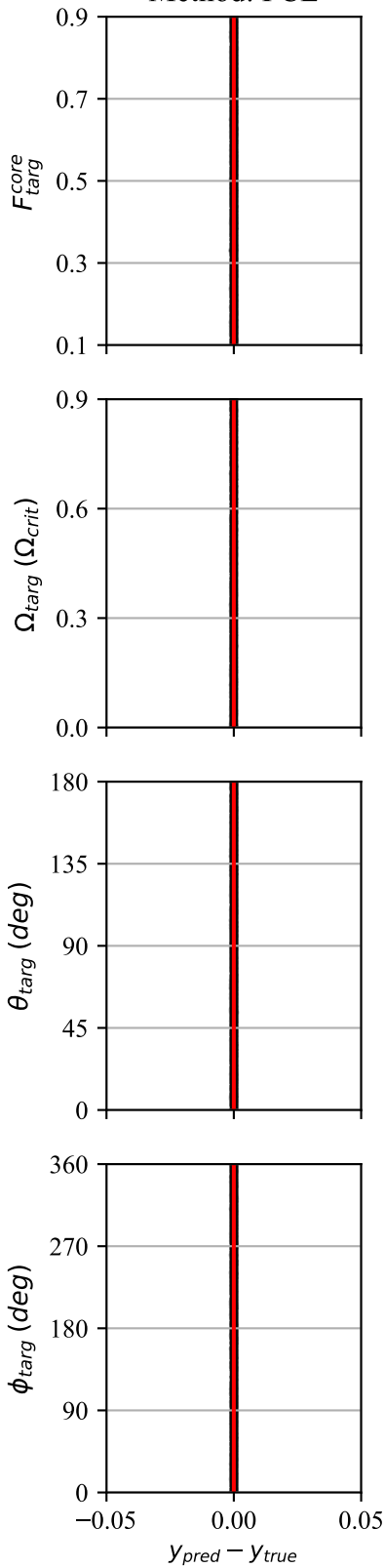

TSS = 11, 884

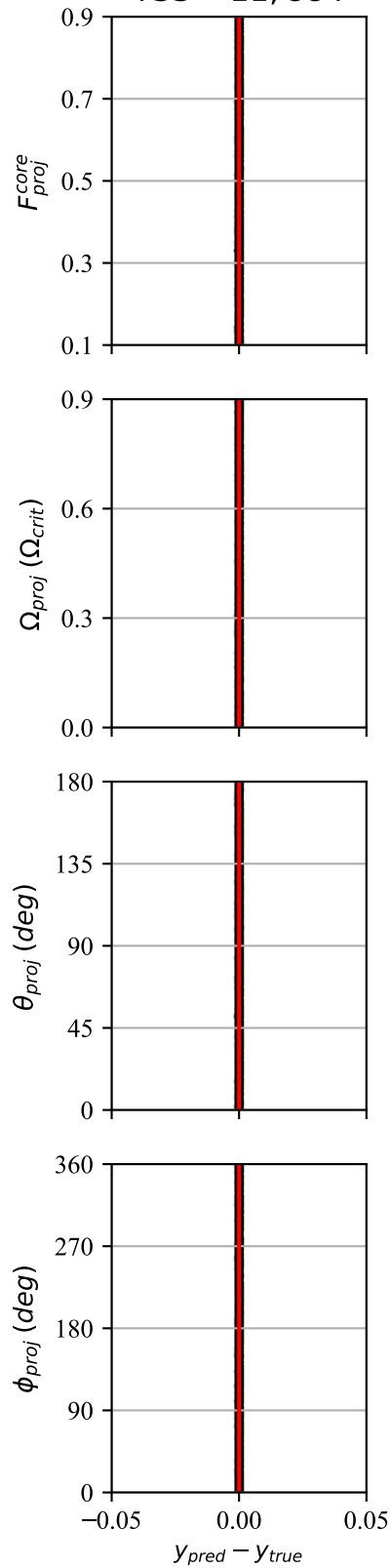

Supplement: Supplementary file 1 — Supplementary information (ZIP 48.3 MB) [file 40668_2020_34_MOESM1_ESM.zip › residuals_lr_omega_pce_11884.pdf]

Target:  $\Omega_{\text{LR}}$ 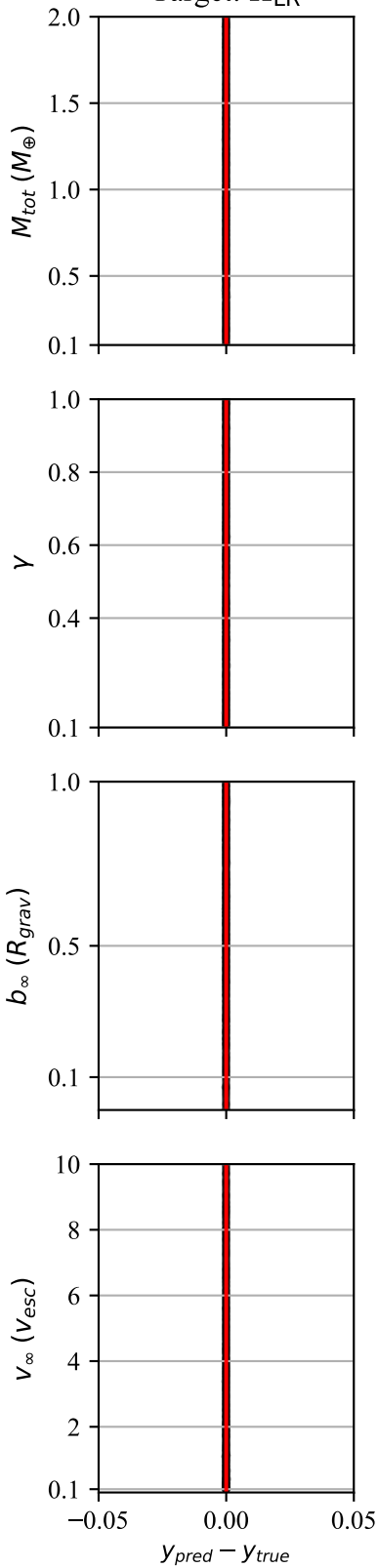

Method: XGB

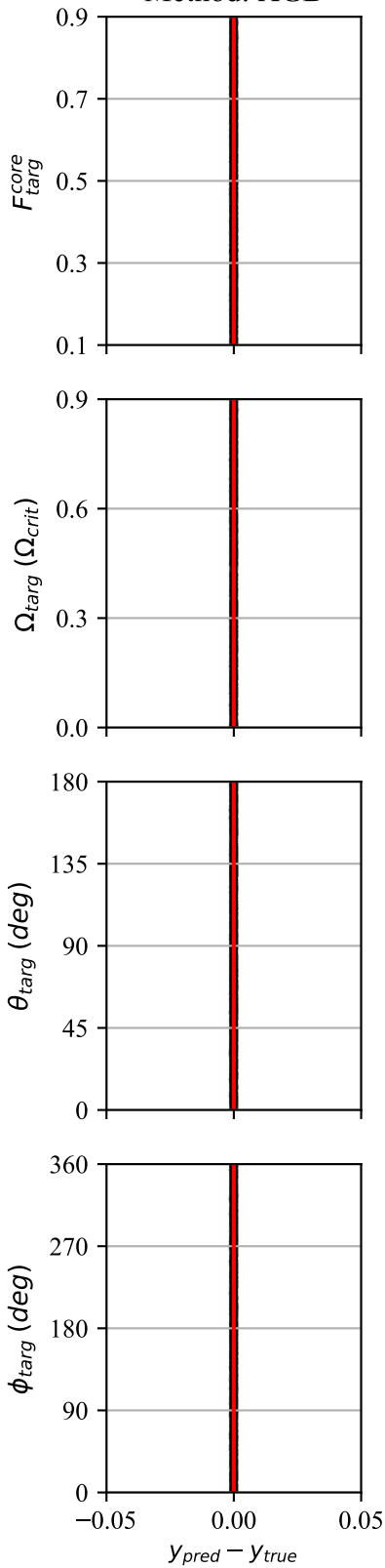

TSS = 11, 884

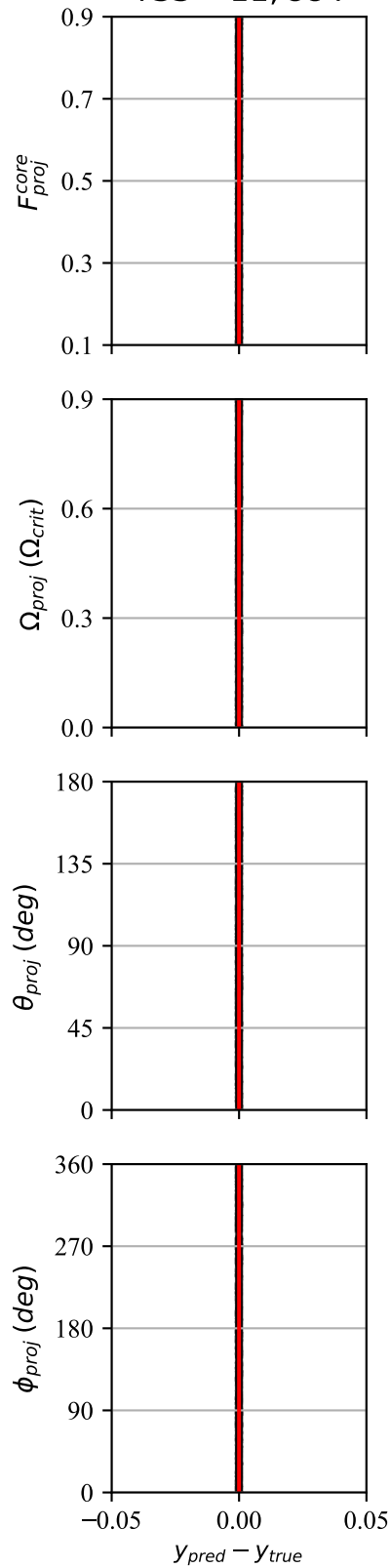

Supplement: Supplementary file 1 — Supplementary information (ZIP 48.3 MB) [file 40668_2020_34_MOESM1_ESM.zip › residuals_lr_omega_xgb_11884.pdf]

Target:  $\theta_{\text{LR}}$ 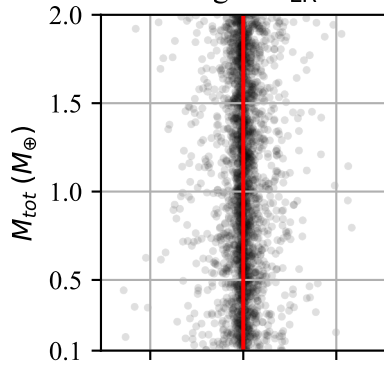

Method: GP

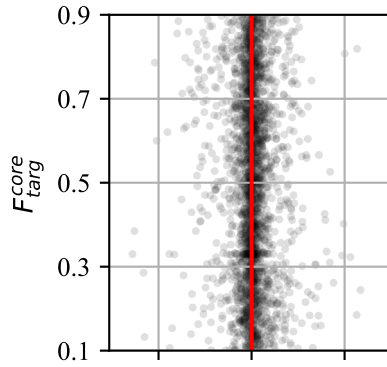

TSS = 11,884

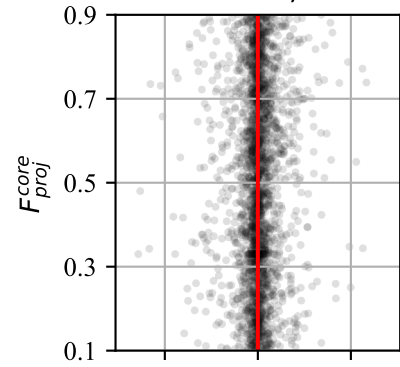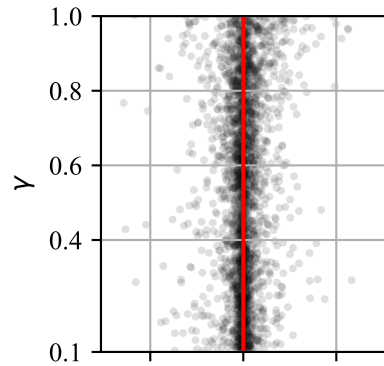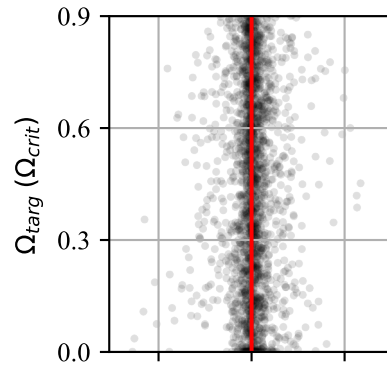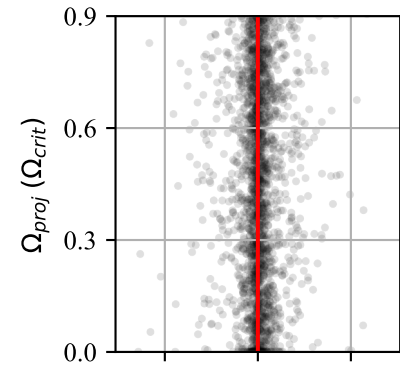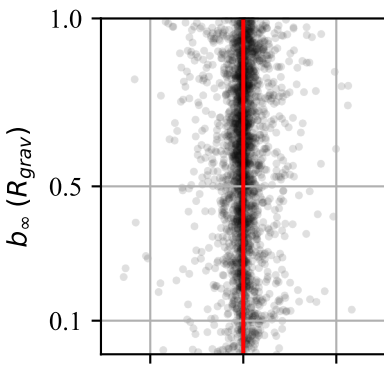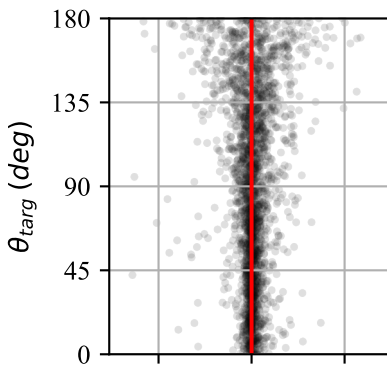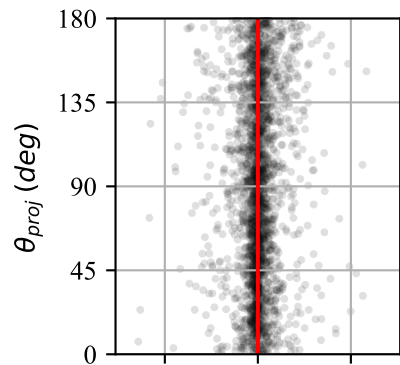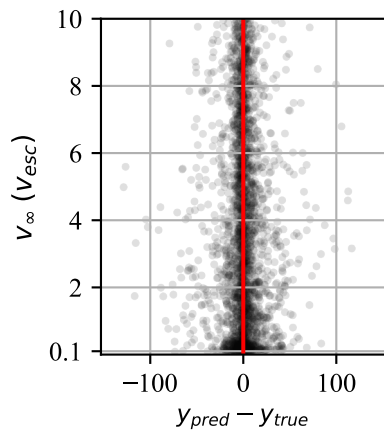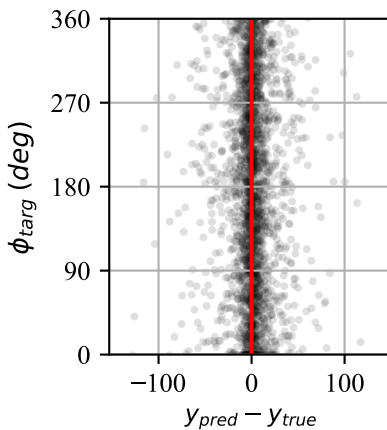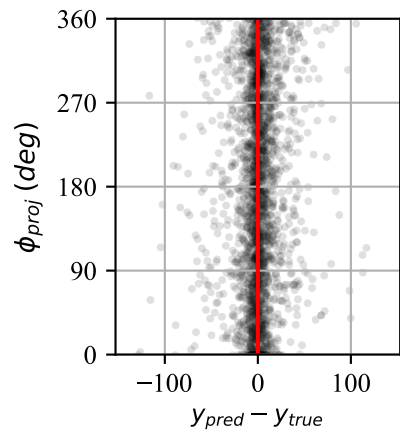

Supplement: Supplementary file 1 — Supplementary information (ZIP 48.3 MB) [file 40668_2020_34_MOESM1_ESM.zip › residuals_lr_theta_gp_11884.pdf]

Target:  $\theta_{\text{LR}}$ 

Method: MLP

TSS = 11,884

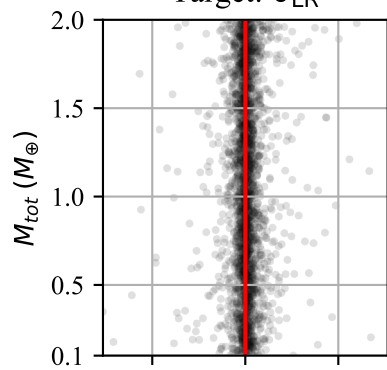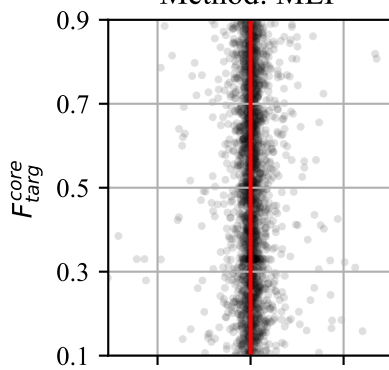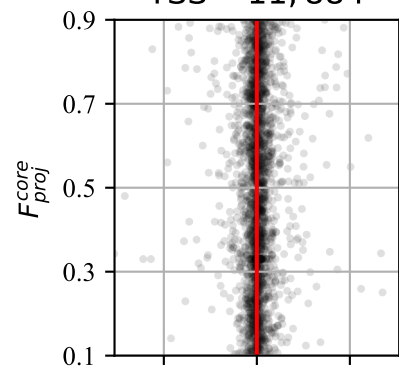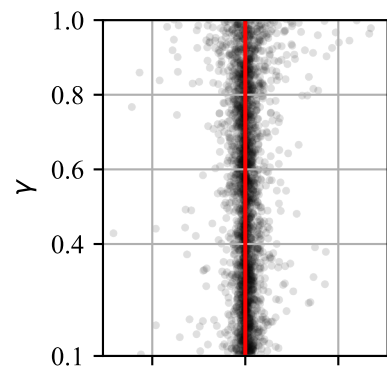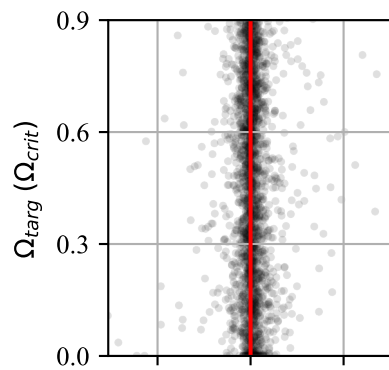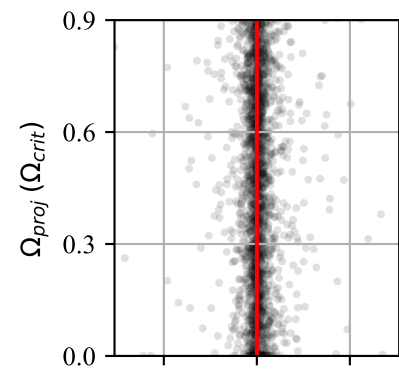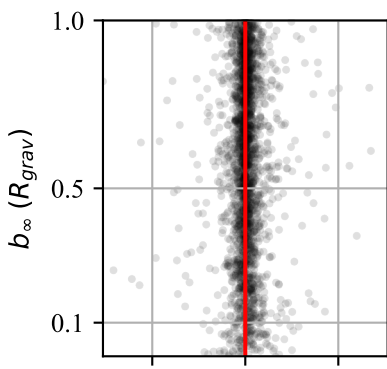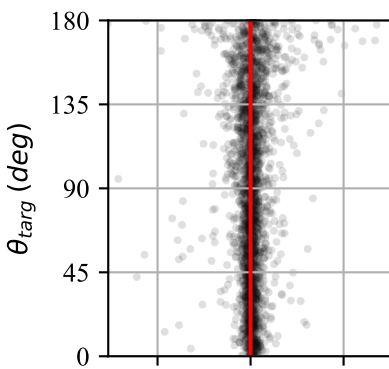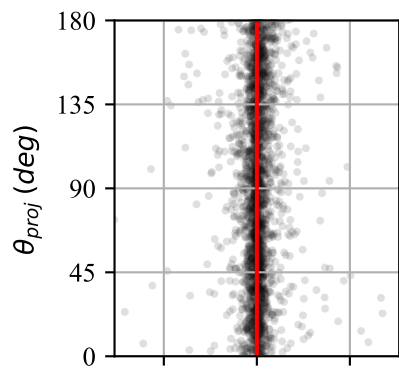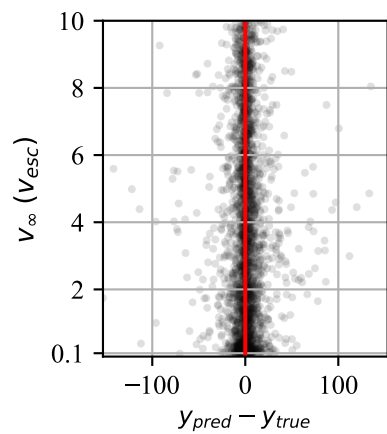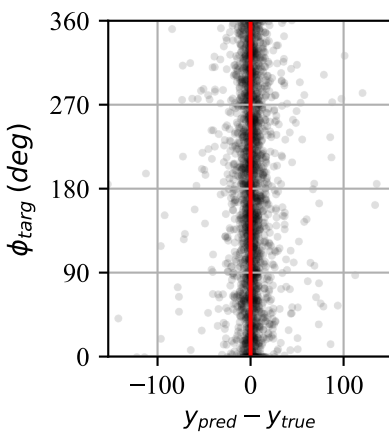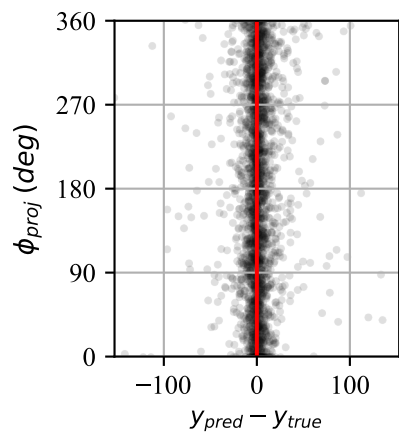

Supplement: Supplementary file 1 — Supplementary information (ZIP 48.3 MB) [file 40668_2020_34_MOESM1_ESM.zip › residuals_lr_theta_mlp_11884.pdf]

Target:  $\theta_{LR}$ 

Method: PCE

TSS = 11,884

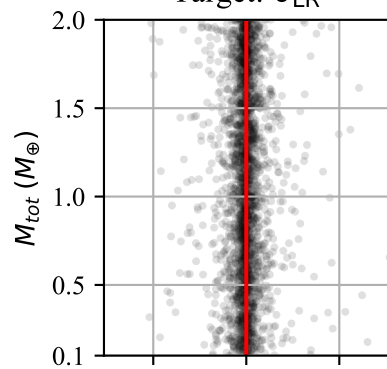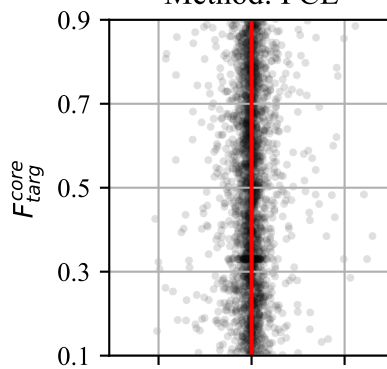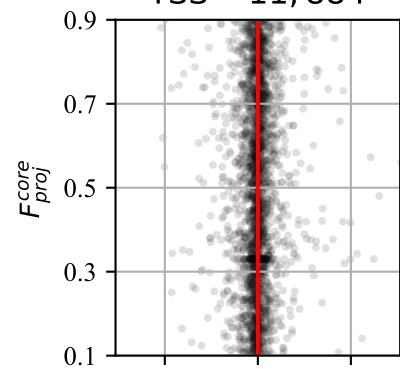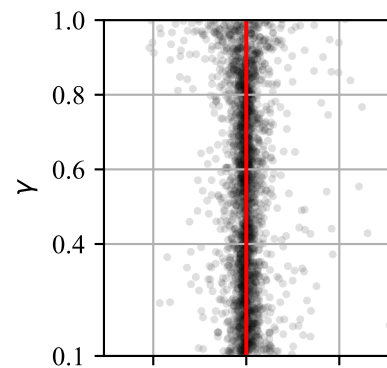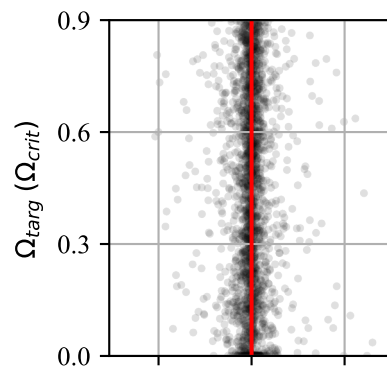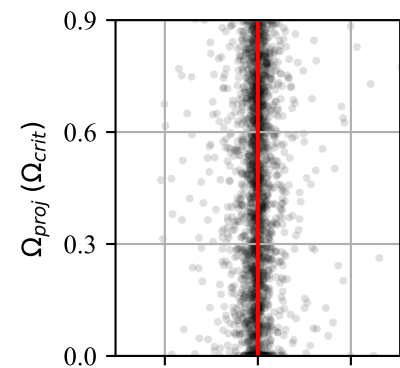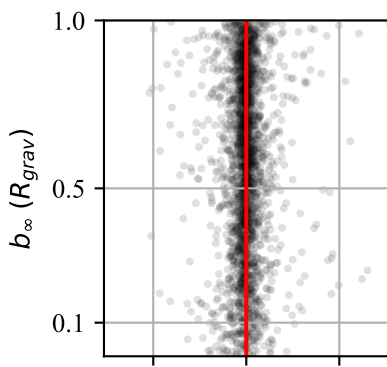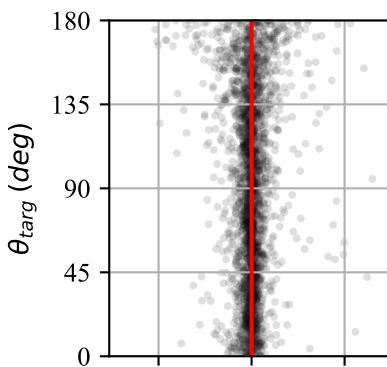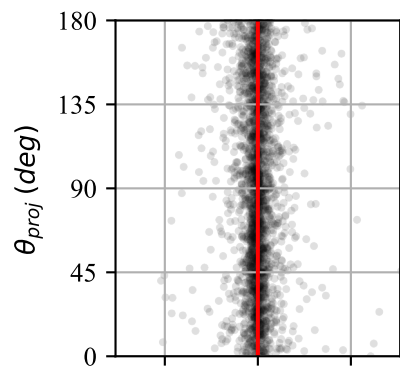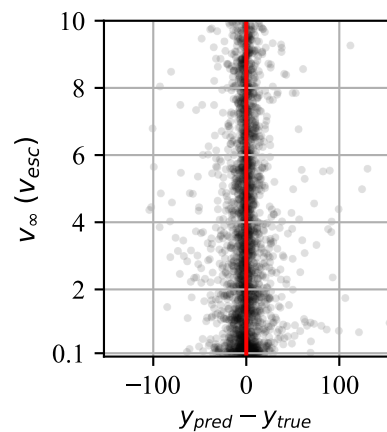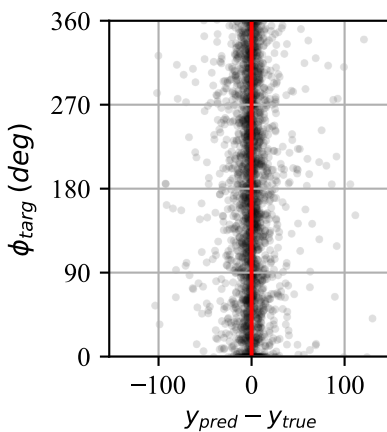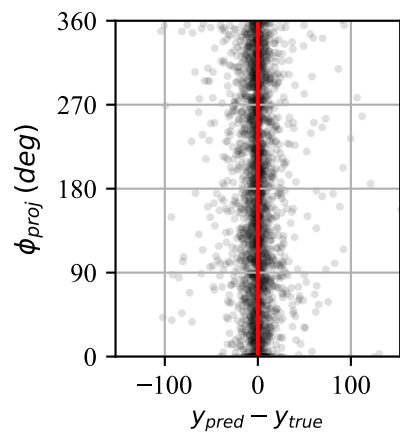

Supplement: Supplementary file 1 — Supplementary information (ZIP 48.3 MB) [file 40668_2020_34_MOESM1_ESM.zip › residuals_lr_theta_pce_11884.pdf]

Target:  $\theta_{\text{LR}}$ 

Method: XGB

TSS = 11,884

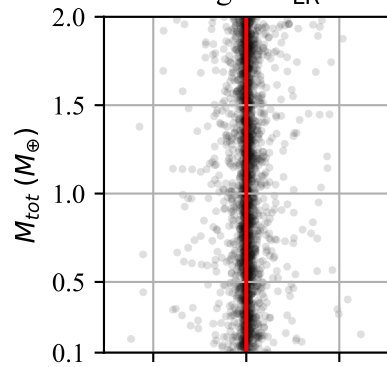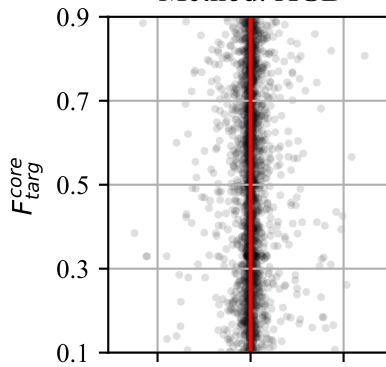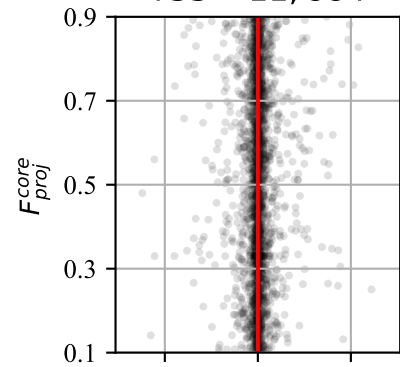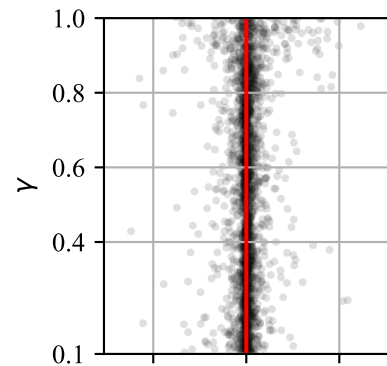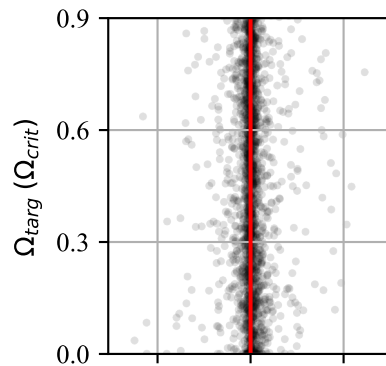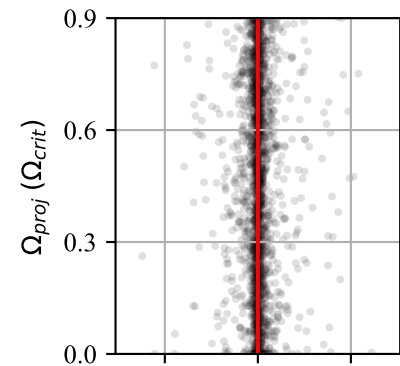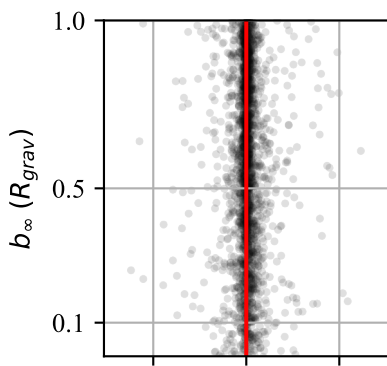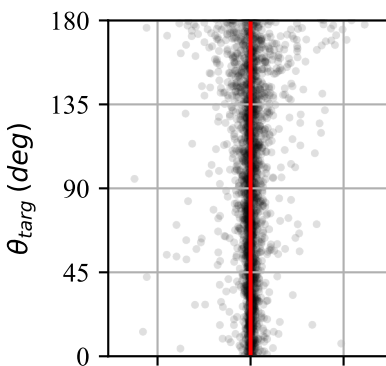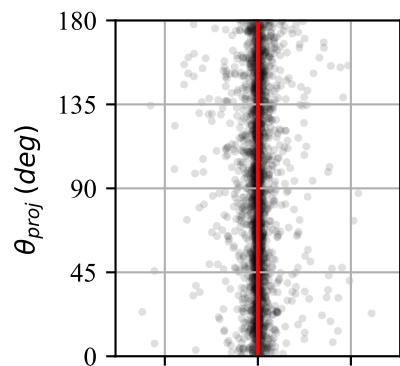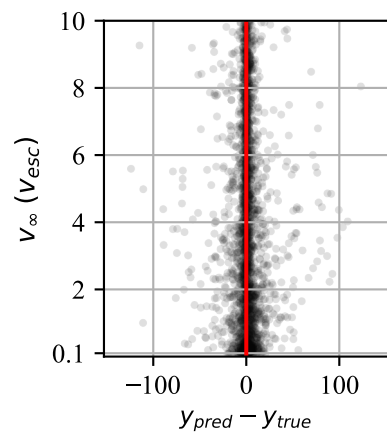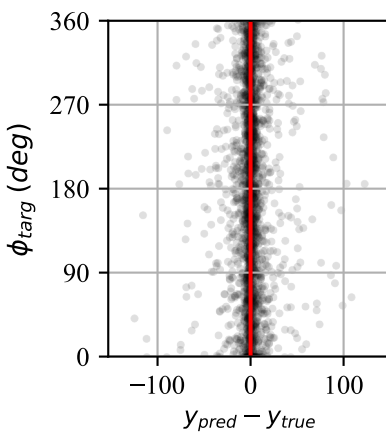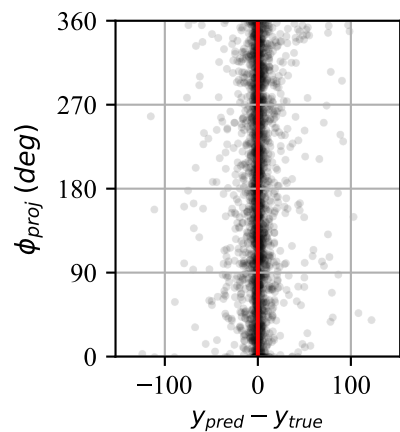

Supplement: Supplementary file 1 — Supplementary information (ZIP 48.3 MB) [file 40668_2020_34_MOESM1_ESM.zip › residuals_lr_theta_xgb_11884.pdf]

Target: J<sub>SLR</sub>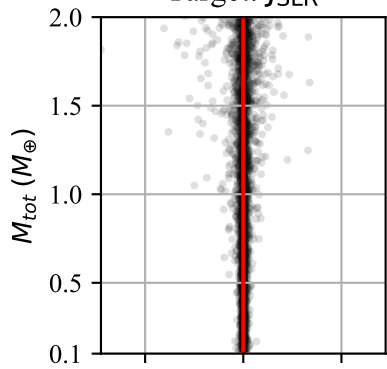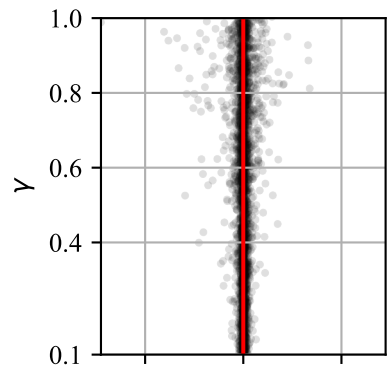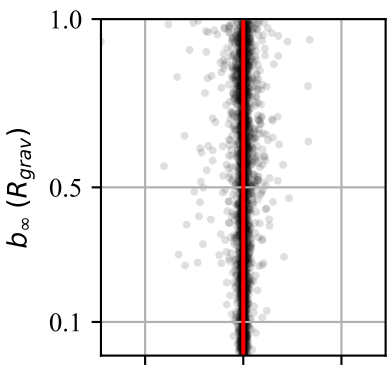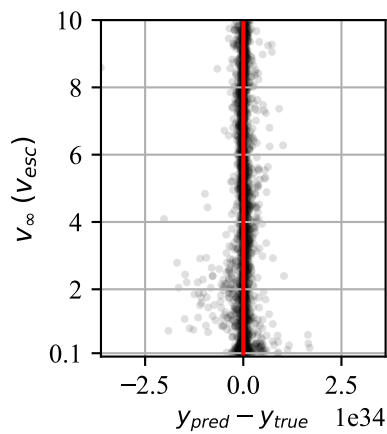

Method: GP

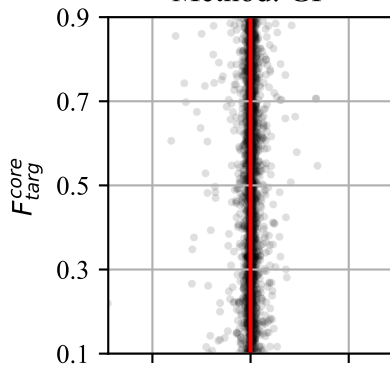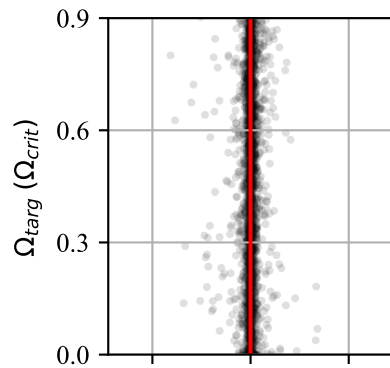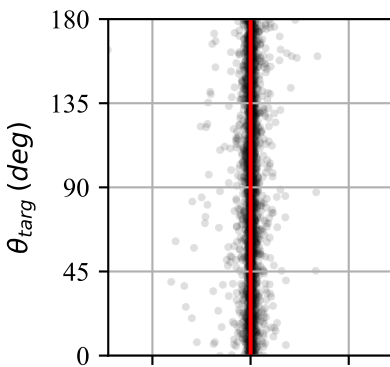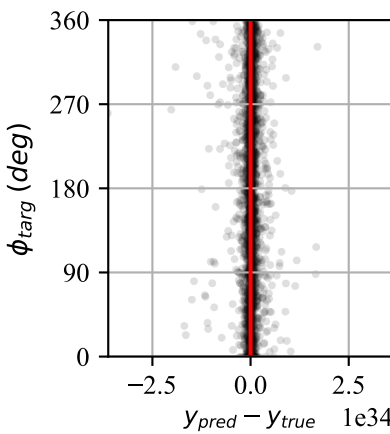

TSS = 11,884

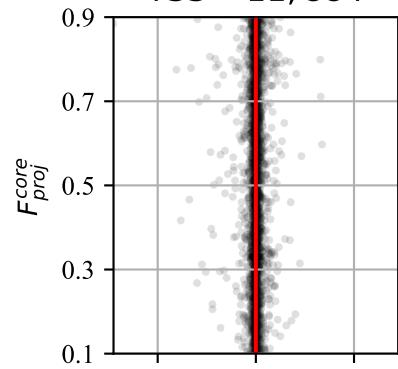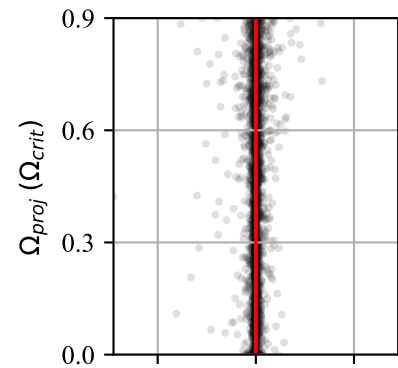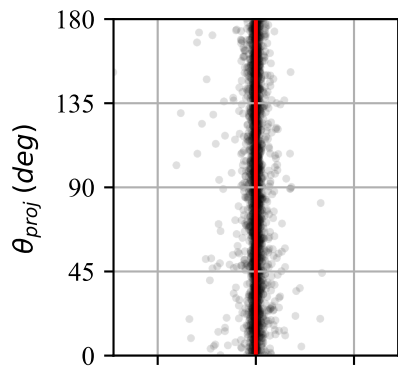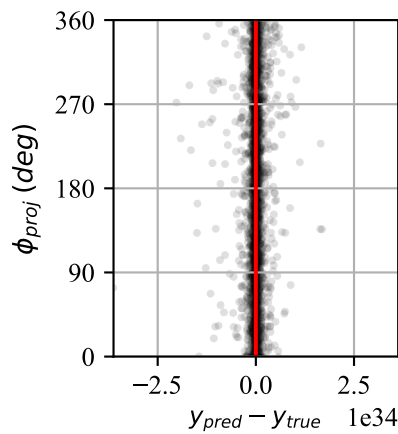

Supplement: Supplementary file 1 — Supplementary information (ZIP 48.3 MB) [file 40668_2020_34_MOESM1_ESM.zip › residuals_slr_angular_momentum_gp_11884.pdf]

Target: J<sub>SLR</sub>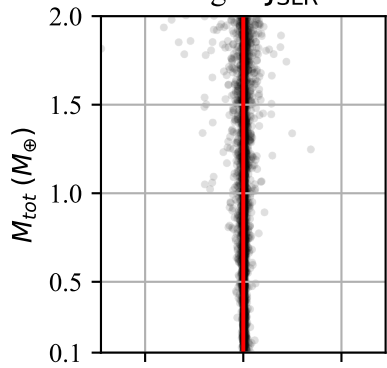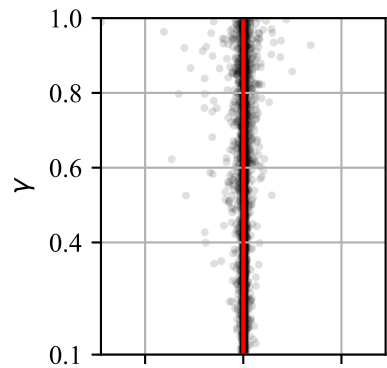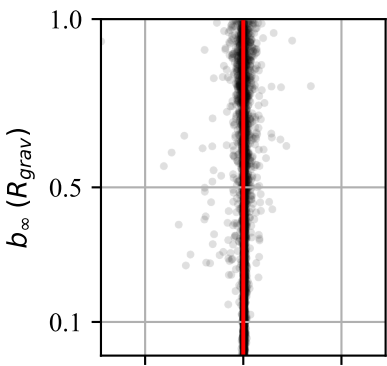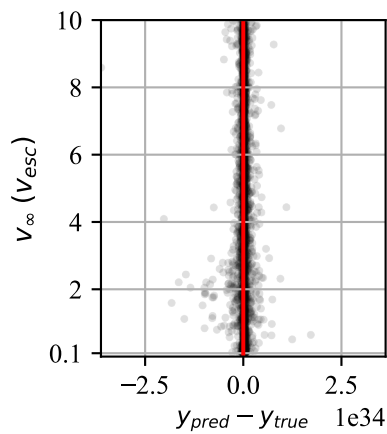

Method: MLP

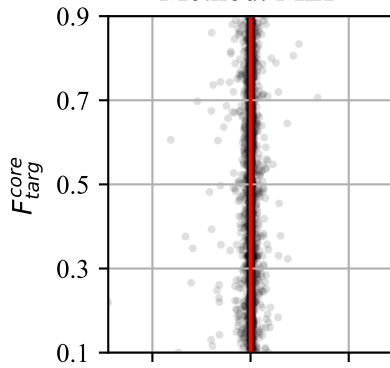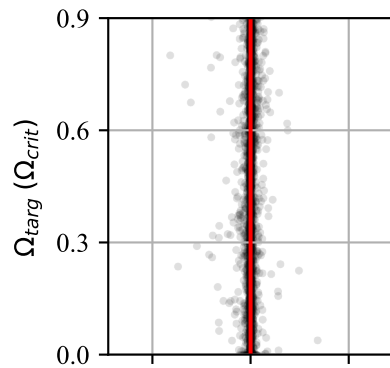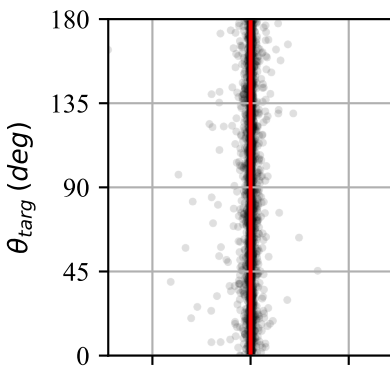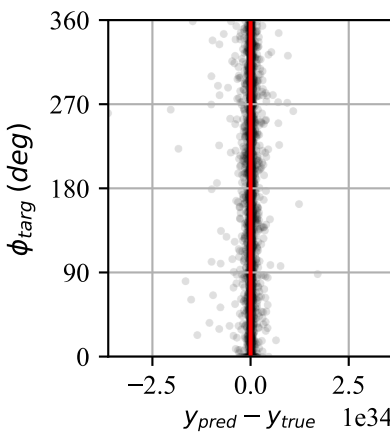

TSS = 11,884

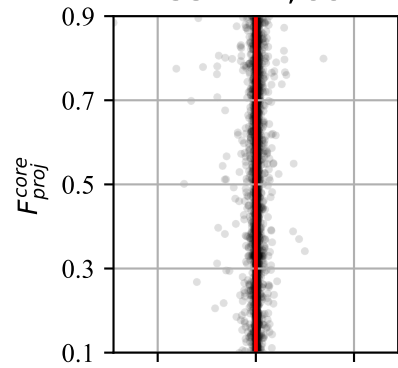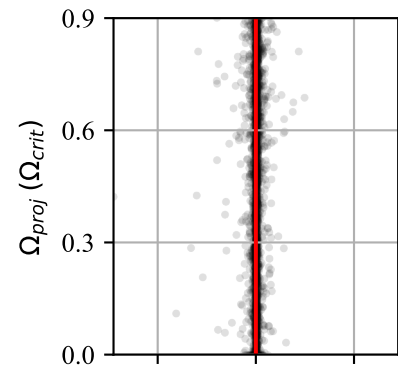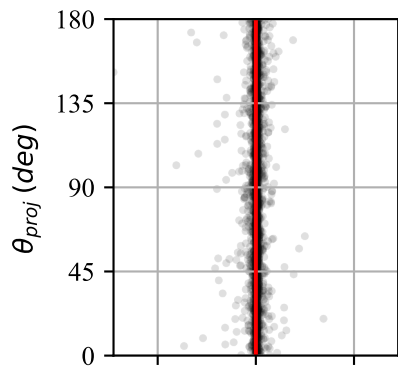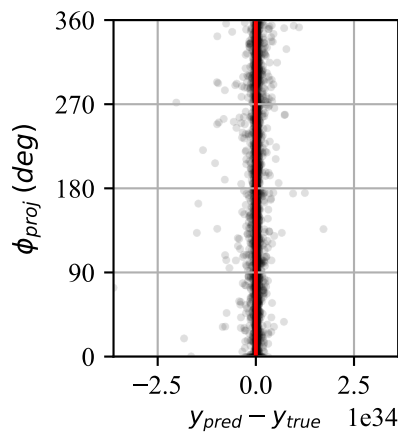

Supplement: Supplementary file 1 — Supplementary information (ZIP 48.3 MB) [file 40668_2020_34_MOESM1_ESM.zip › residuals_slr_angular_momentum_mlp_11884.pdf]

Target: J<sub>SLR</sub>

Method: PCE

TSS = 11,884

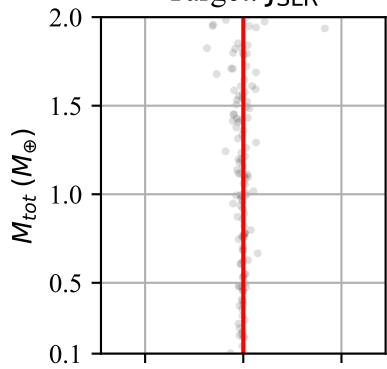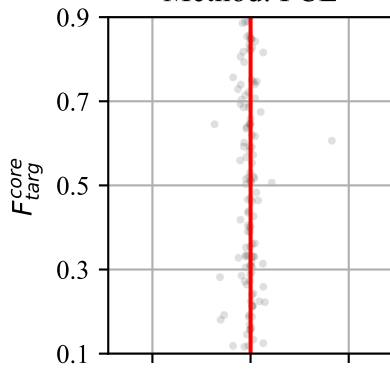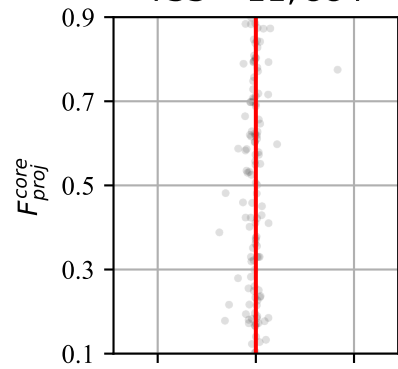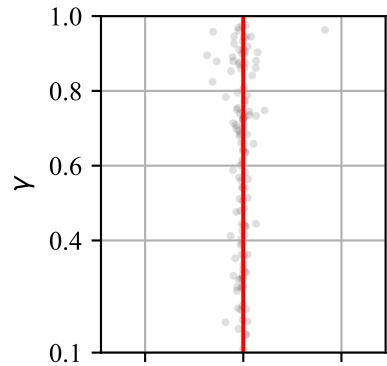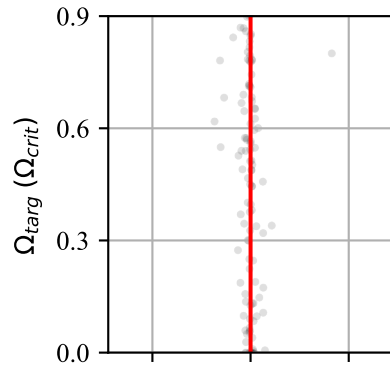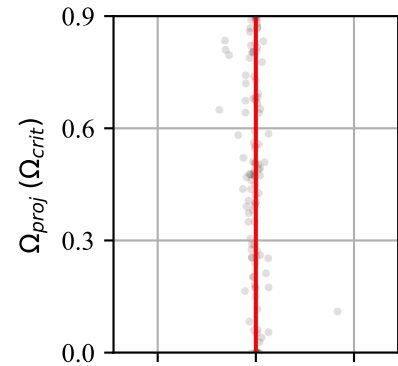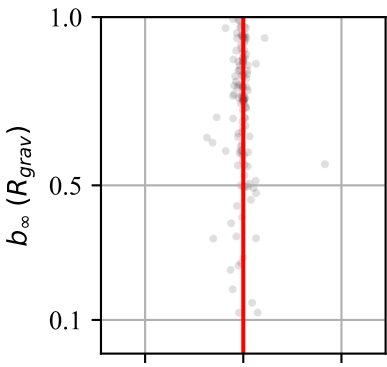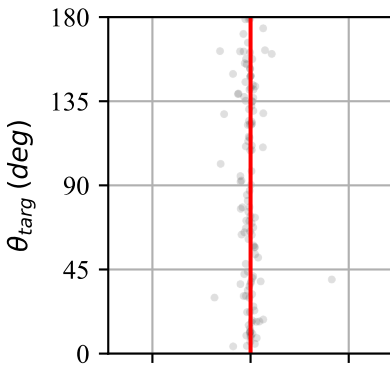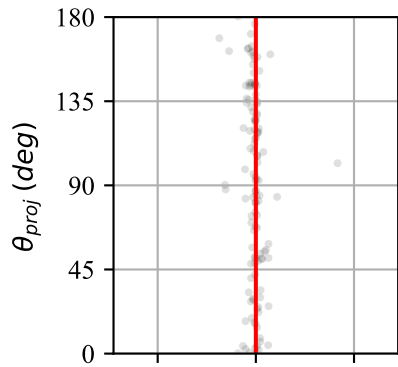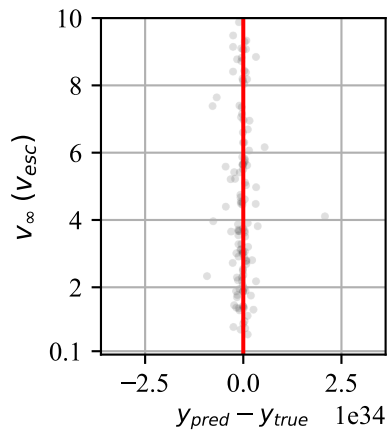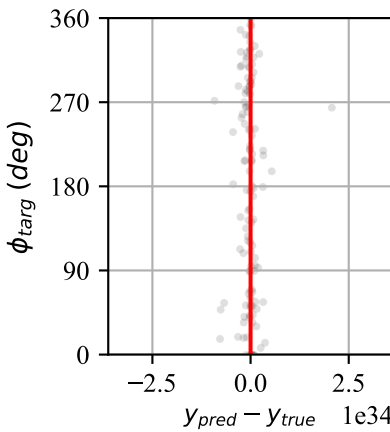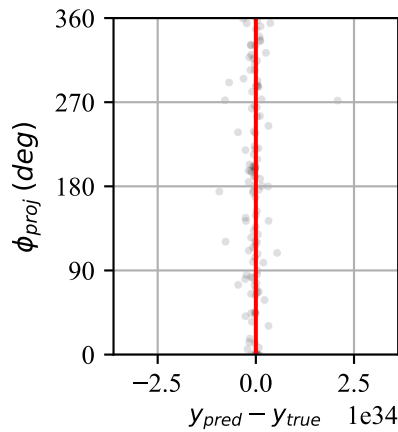

Supplement: Supplementary file 1 — Supplementary information (ZIP 48.3 MB) [file 40668_2020_34_MOESM1_ESM.zip › residuals_slr_angular_momentum_pce_11884.pdf]

Target: J<sub>SLR</sub>

Method: XGB

TSS = 11, 884

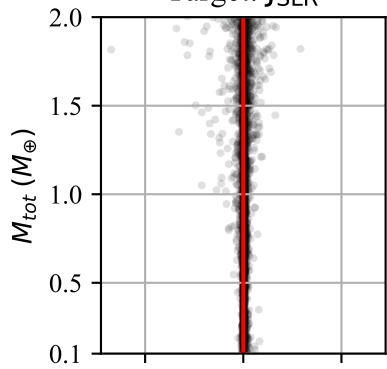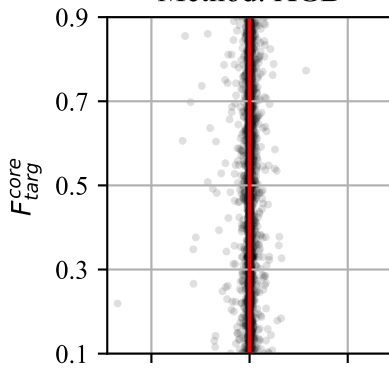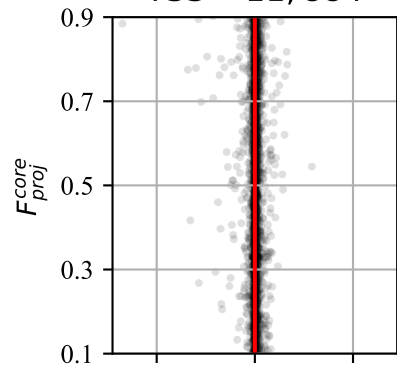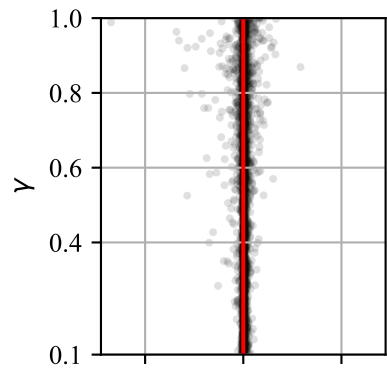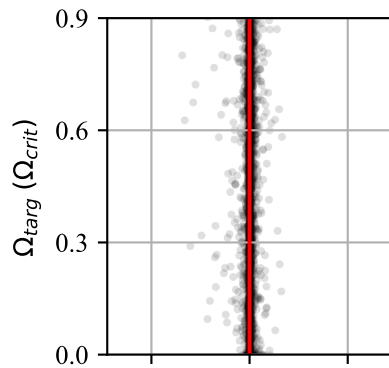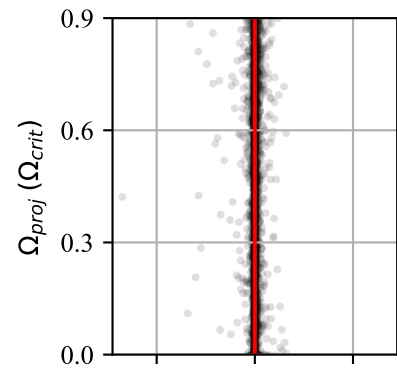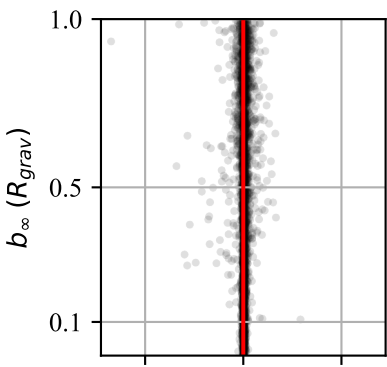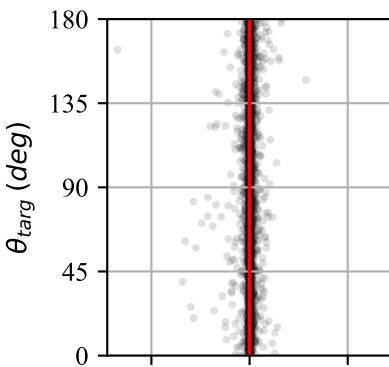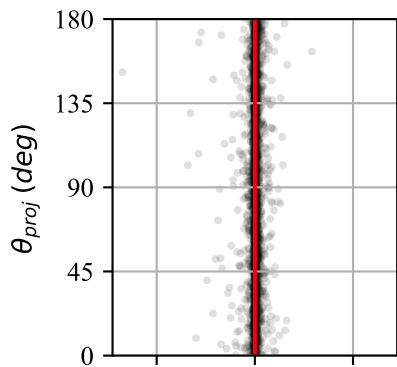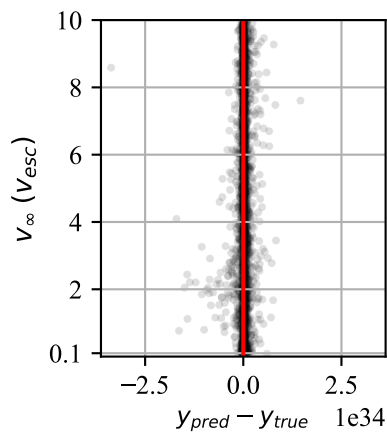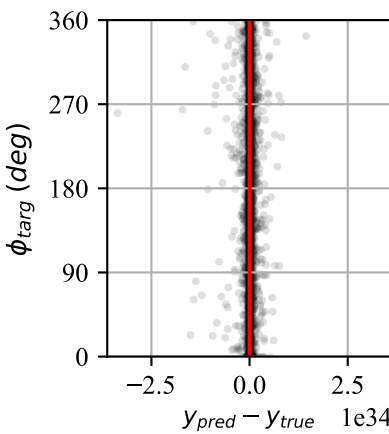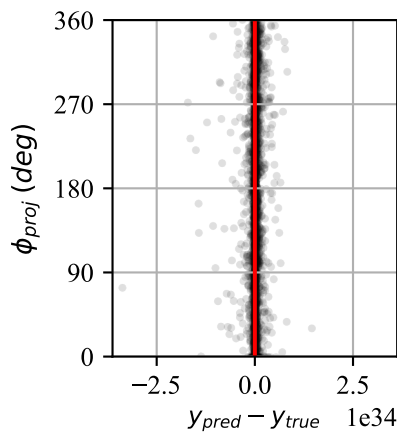

Supplement: Supplementary file 1 — Supplementary information (ZIP 48.3 MB) [file 40668_2020_34_MOESM1_ESM.zip › residuals_slr_angular_momentum_xgb_11884.pdf]

Target:  $F_{\text{SLR}}^{\text{core}}$ 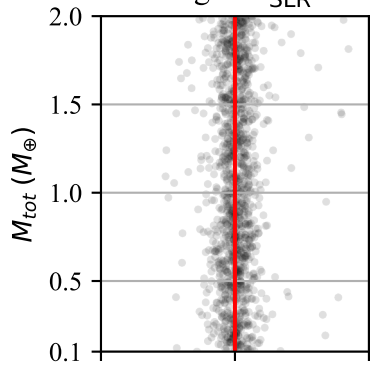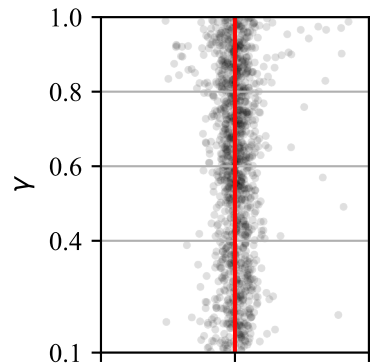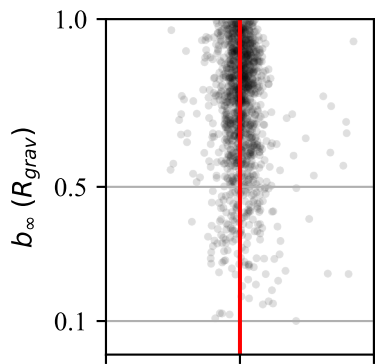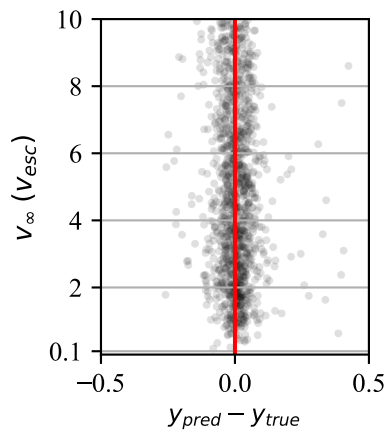

Method: MLP

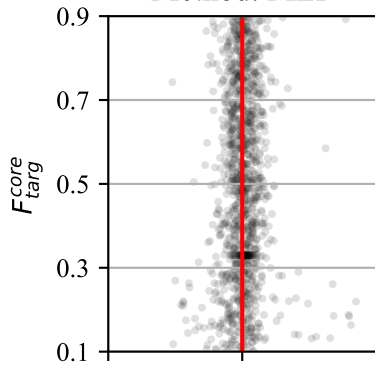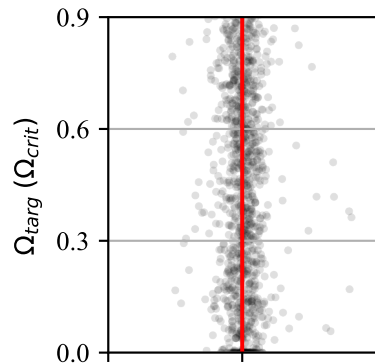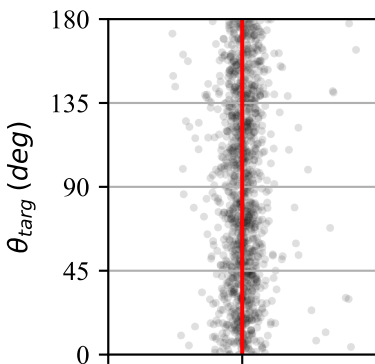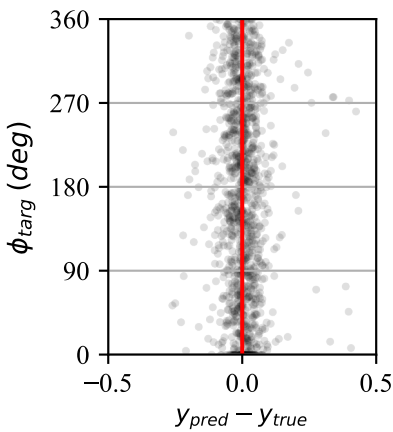

TSS = 11,884

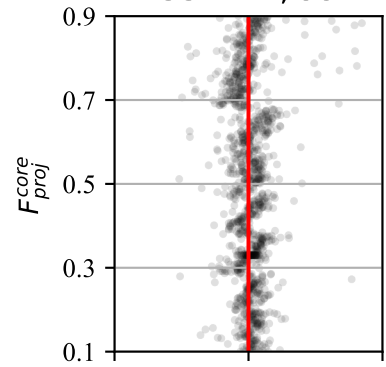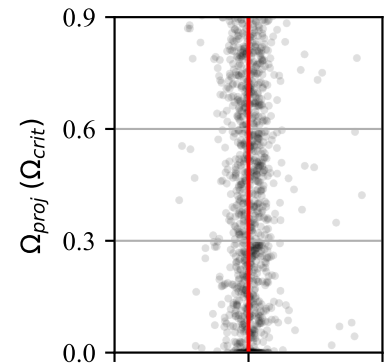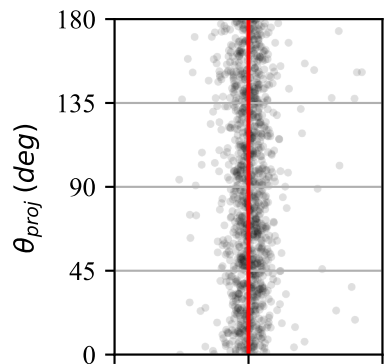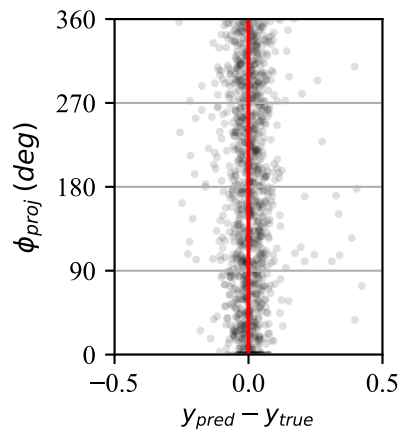

Supplement: Supplementary file 1 — Supplementary information (ZIP 48.3 MB) [file 40668_2020_34_MOESM1_ESM.zip › residuals_slr_core_fraction_mlp_11884.pdf]

Target:  $F_{\text{SLR}}^{\text{melt}}$ 

Method: XGB

TSS = 11,884

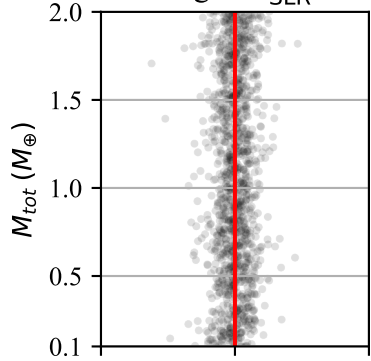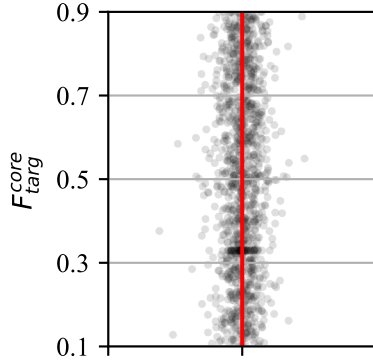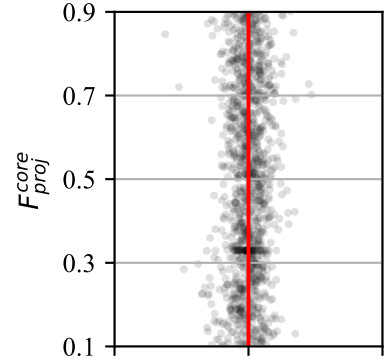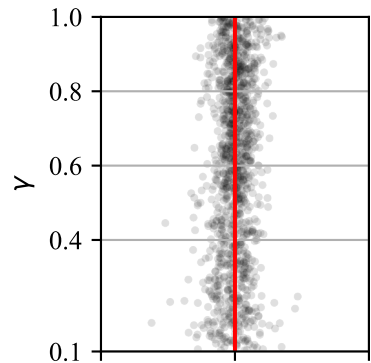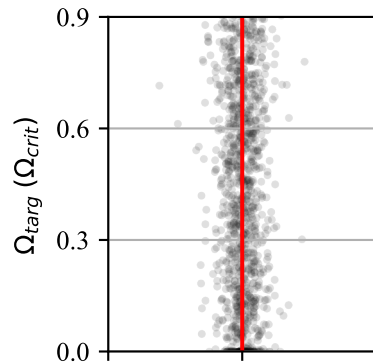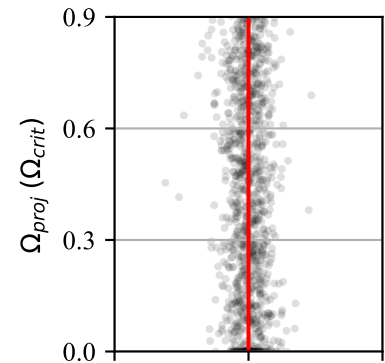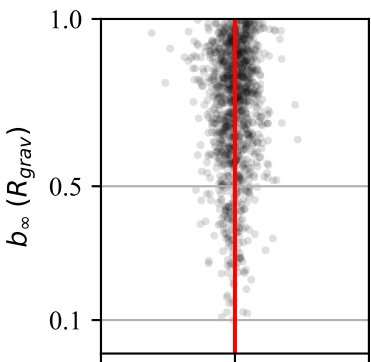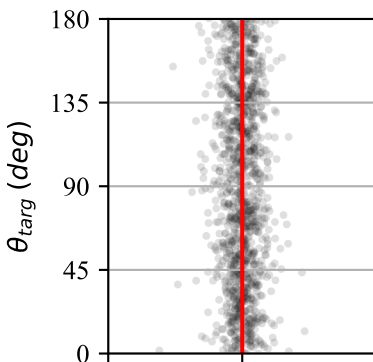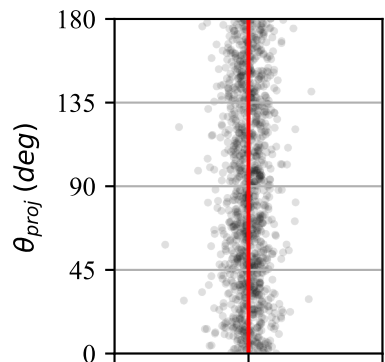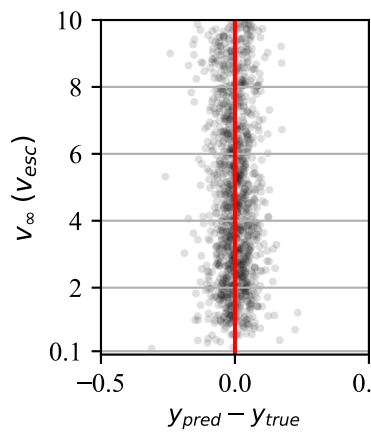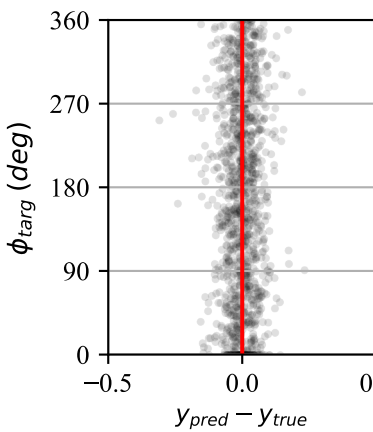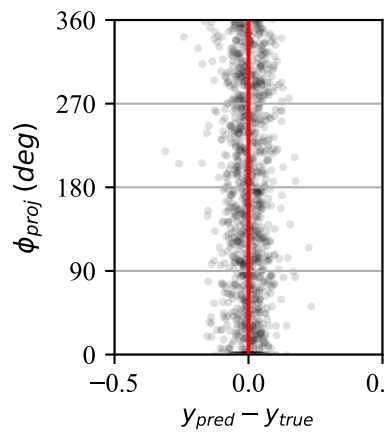

Supplement: Supplementary file 1 — Supplementary information (ZIP 48.3 MB) [file 40668_2020_34_MOESM1_ESM.zip › residuals_slr_expanded_xgb_11884.pdf]

Target:  $M_{\text{SLR}}$ 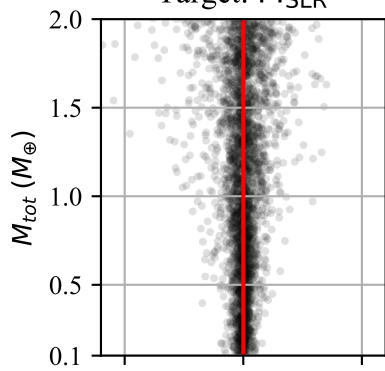

Method: GP

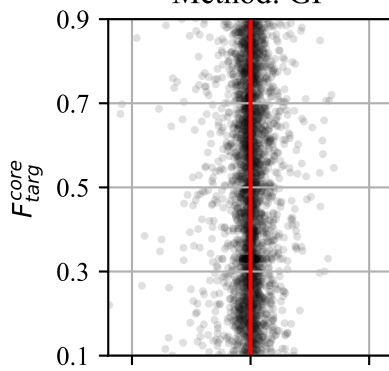

TSS = 11,884

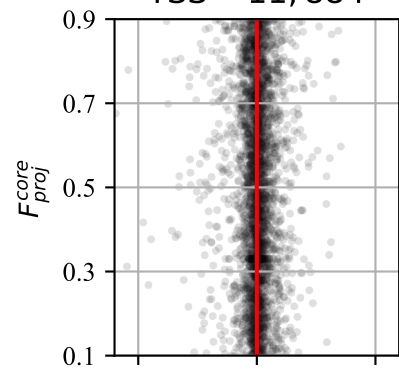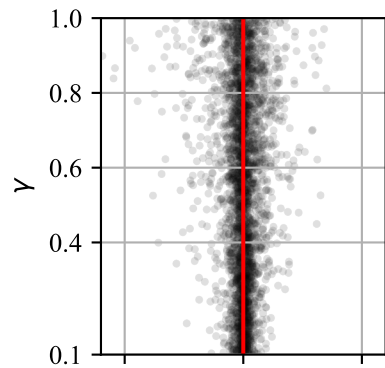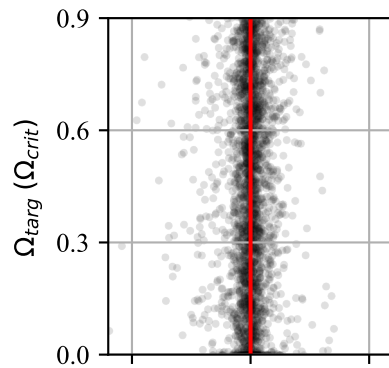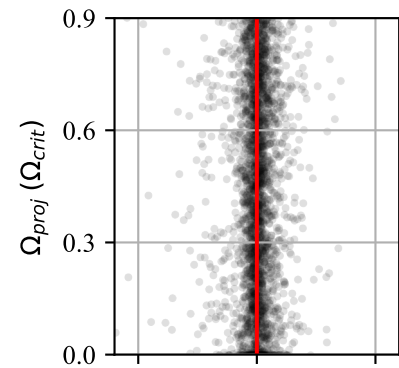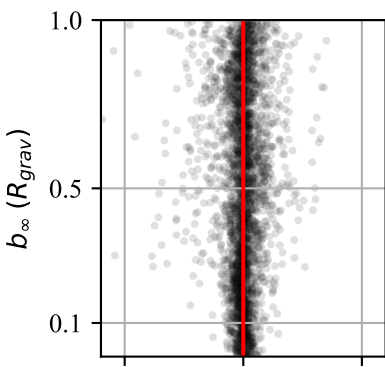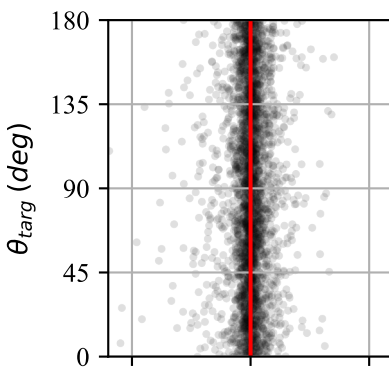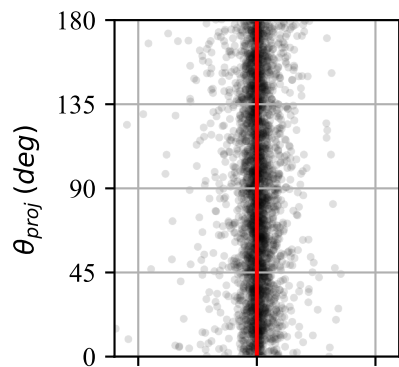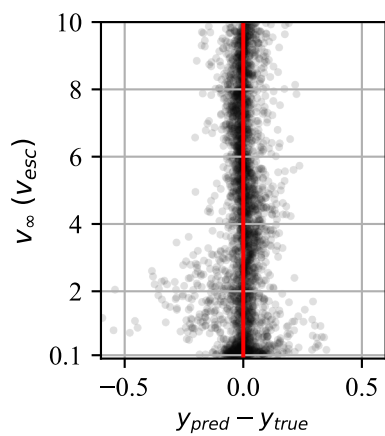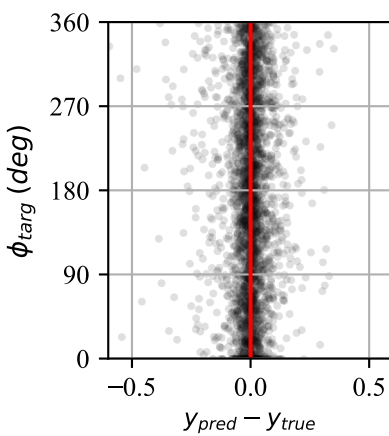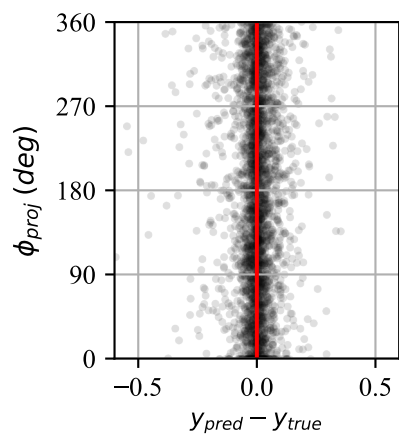

Supplement: Supplementary file 1 — Supplementary information (ZIP 48.3 MB) [file 40668_2020_34_MOESM1_ESM.zip › residuals_slr_mass_gp_11884.pdf]

Target:  $M_{\text{SLR}}$ 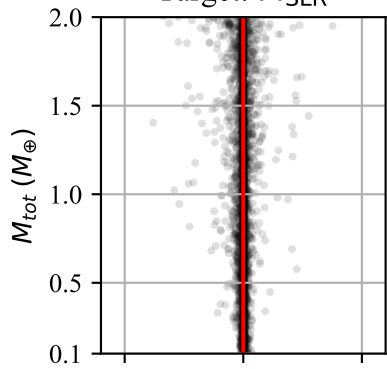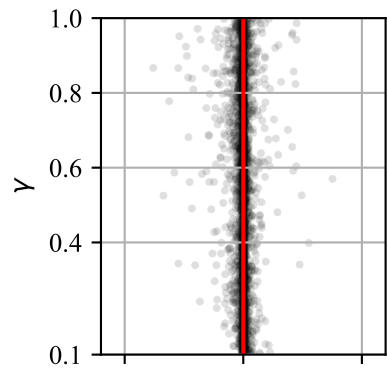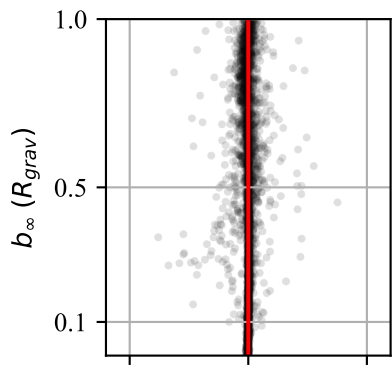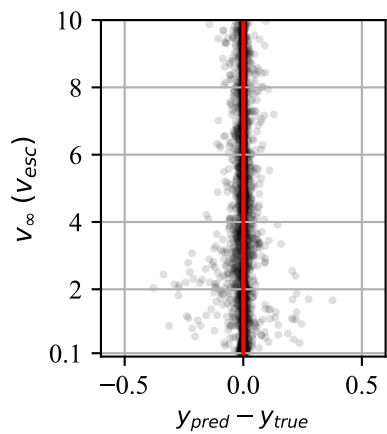

Method: MLP

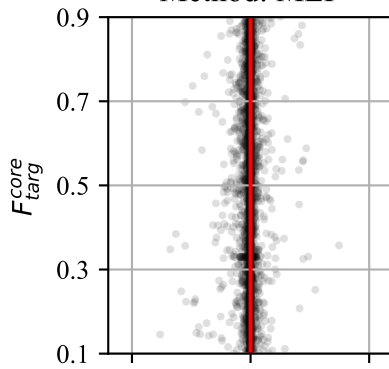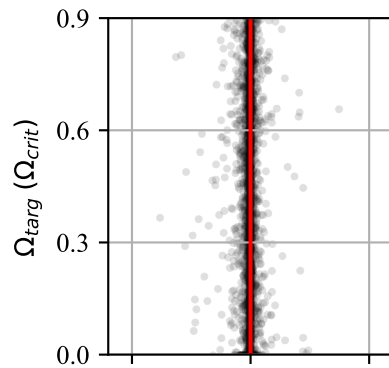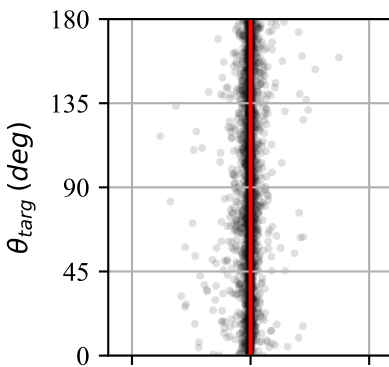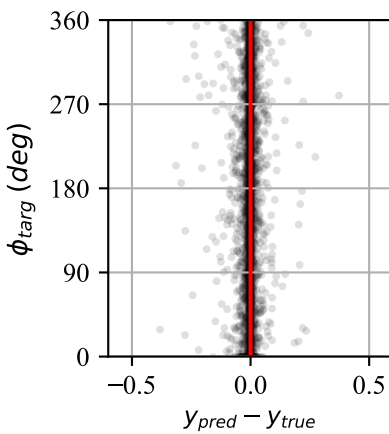

TSS = 11,884

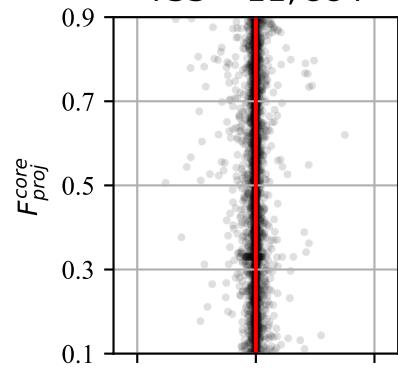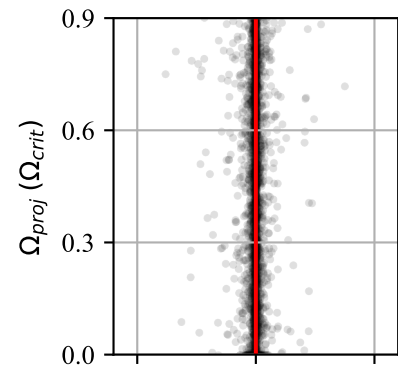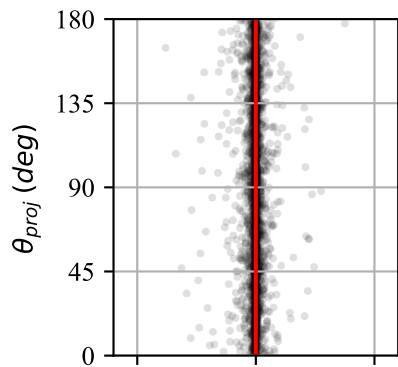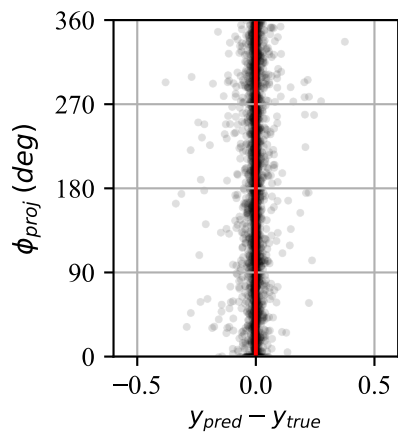

Supplement: Supplementary file 1 — Supplementary information (ZIP 48.3 MB) [file 40668_2020_34_MOESM1_ESM.zip › residuals_slr_mass_mlp_11884.pdf]

Target:  $M_{\text{SLR}}^{\text{norm}}$ 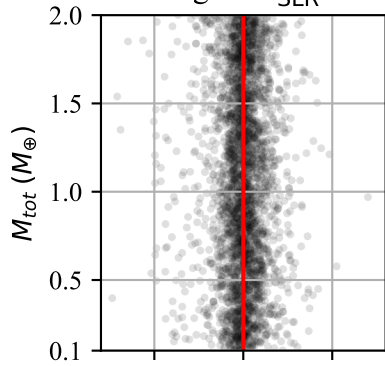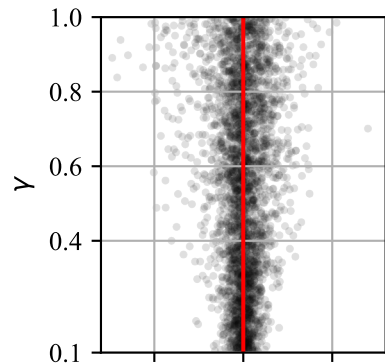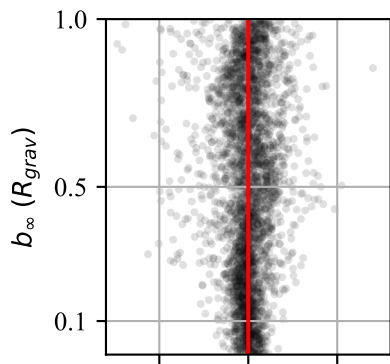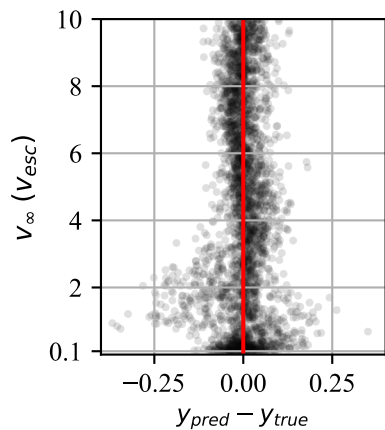

Method: GP

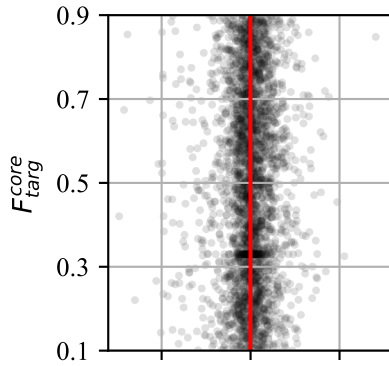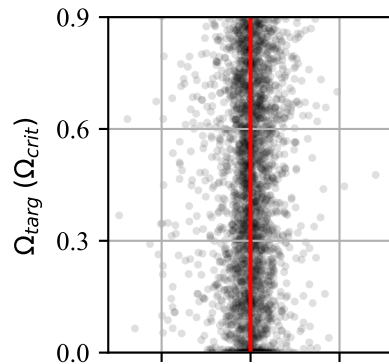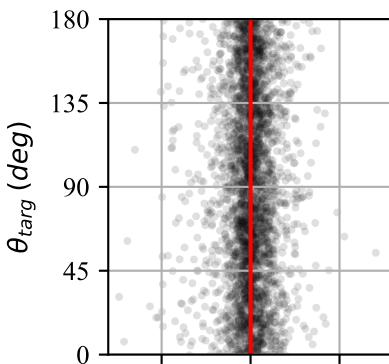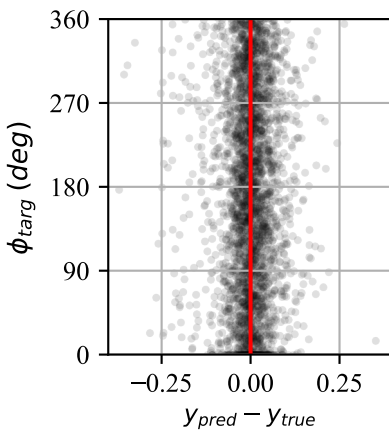

TSS = 11,884

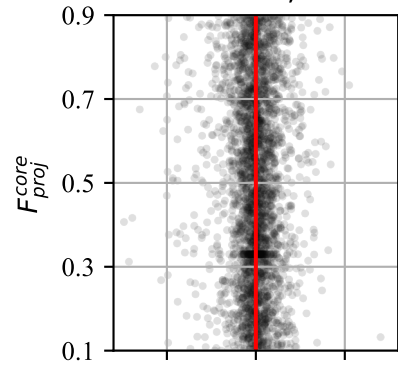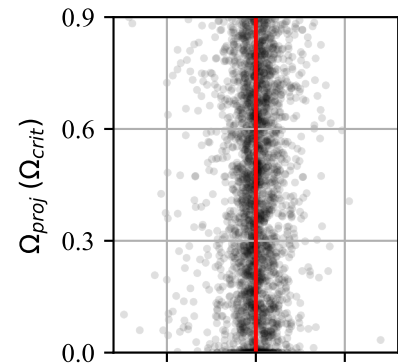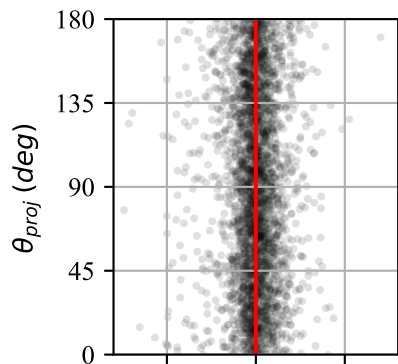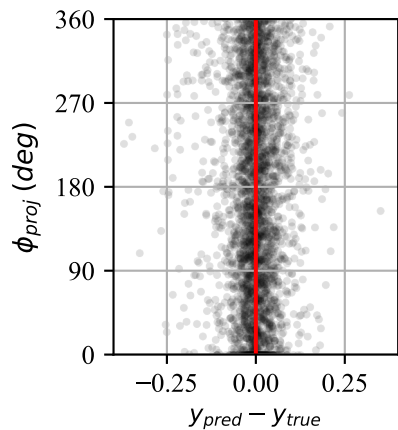

Supplement: Supplementary file 1 — Supplementary information (ZIP 48.3 MB) [file 40668_2020_34_MOESM1_ESM.zip › residuals_slr_mass_norm_gp_11884.pdf]

Target:  $M_{\text{SLR}}^{\text{norm}}$ 

Method: MLP

TSS = 11,884

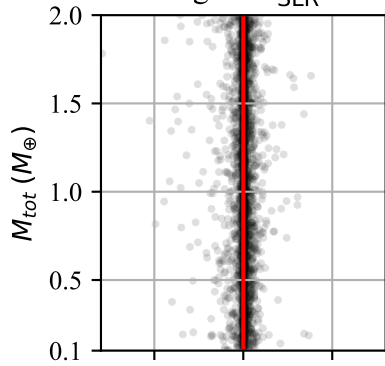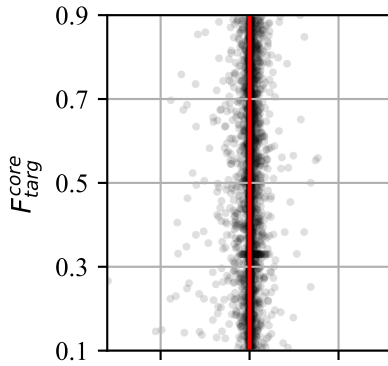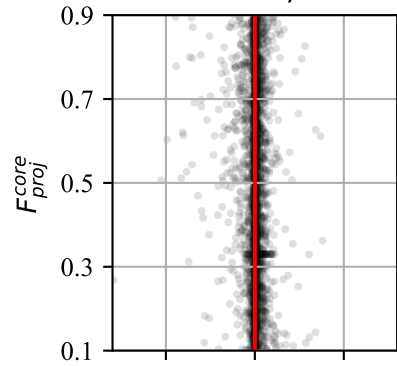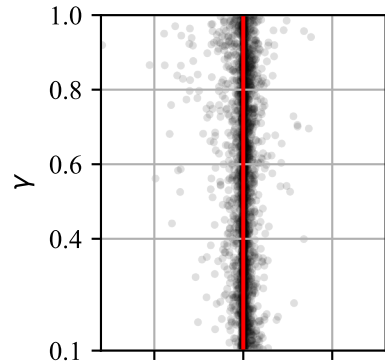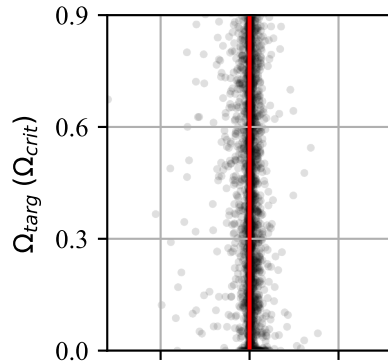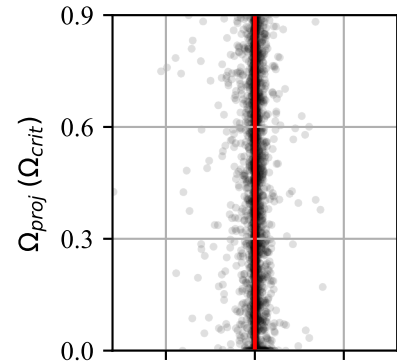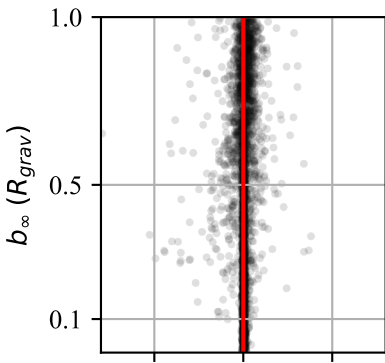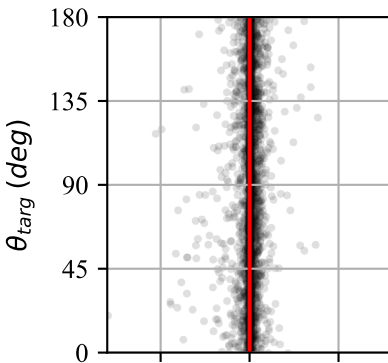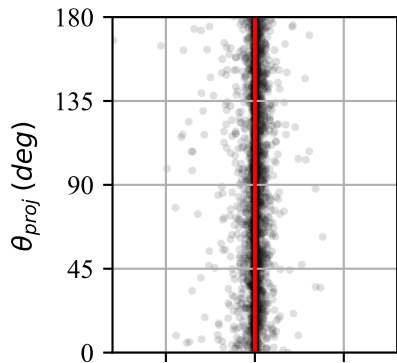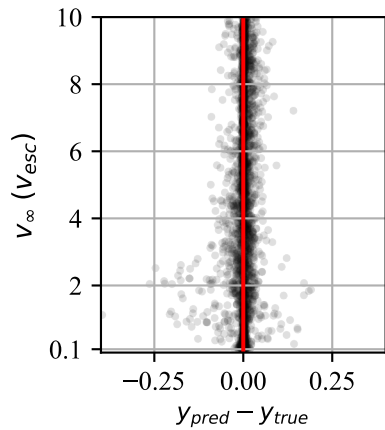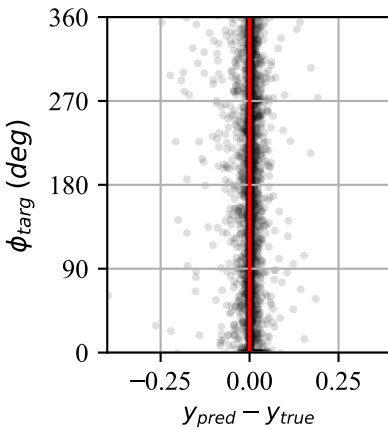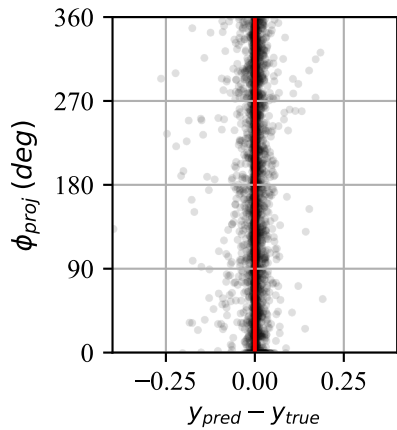

Supplement: Supplementary file 1 — Supplementary information (ZIP 48.3 MB) [file 40668_2020_34_MOESM1_ESM.zip › residuals_slr_mass_norm_mlp_11884.pdf]

Target:  $M_{\text{SLR}}^{\text{norm}}$ 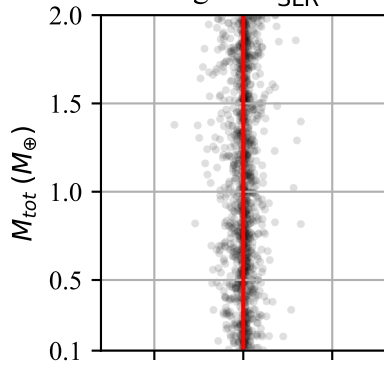

Method: PCE

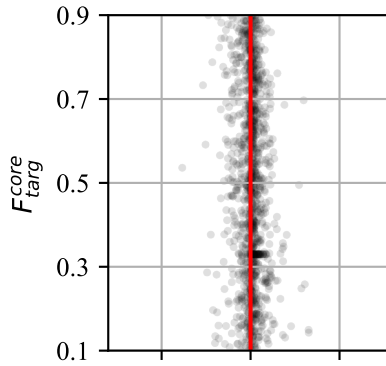

TSS = 11,884

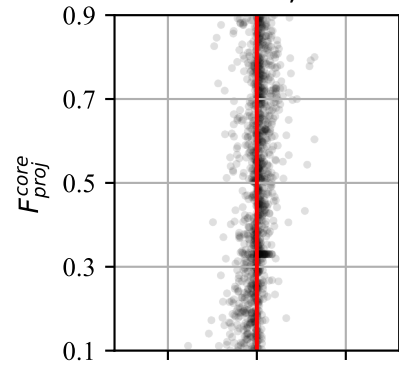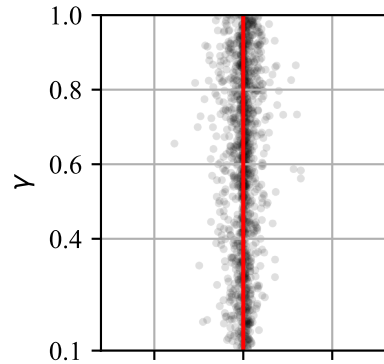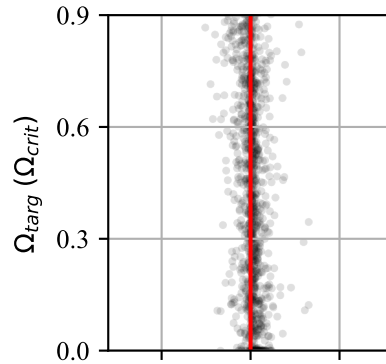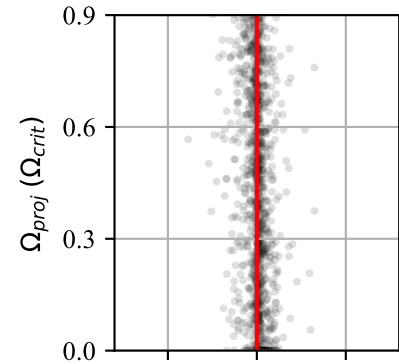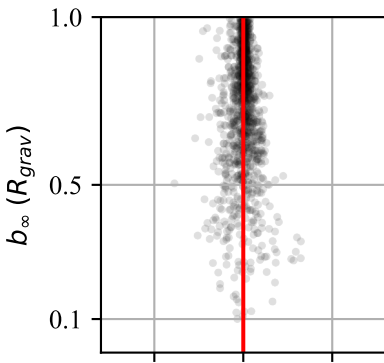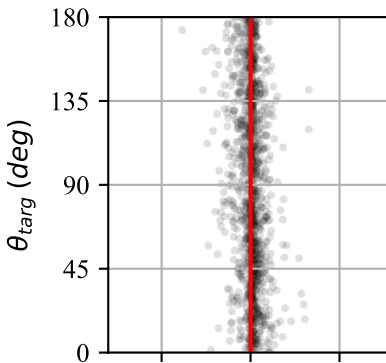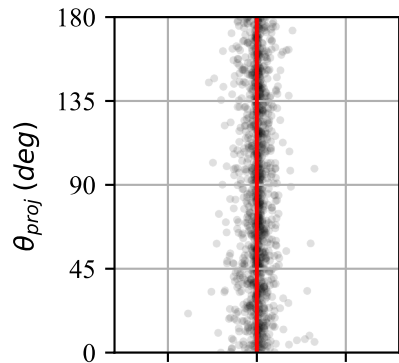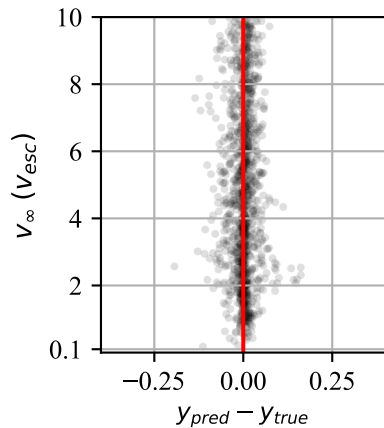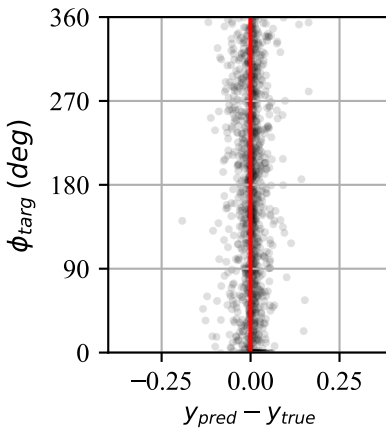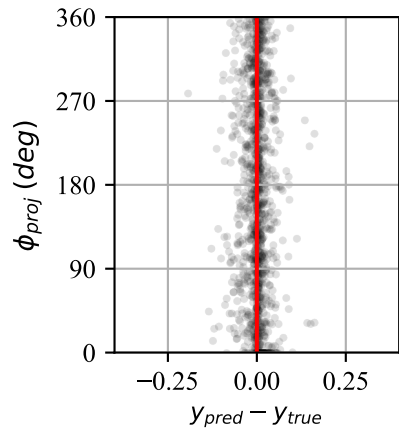

Supplement: Supplementary file 1 — Supplementary information (ZIP 48.3 MB) [file 40668_2020_34_MOESM1_ESM.zip › residuals_slr_mass_norm_pce_11884.pdf]

Target:  $M_{\text{SLR}}^{\text{norm}}$ 

Method: XGB

TSS = 11,884

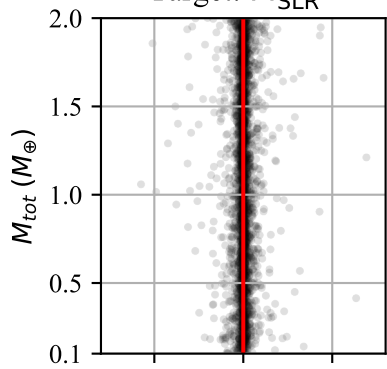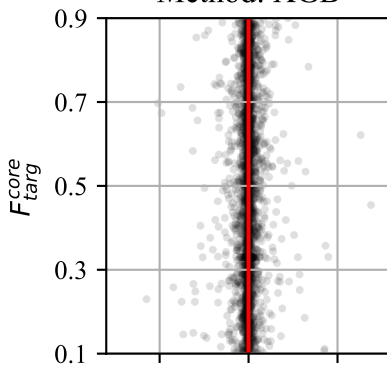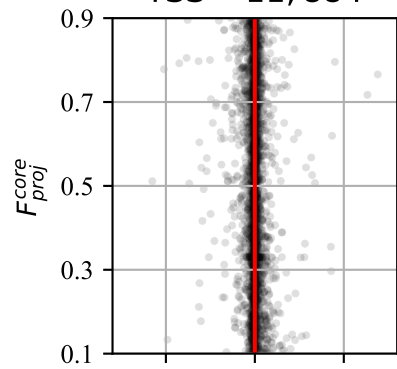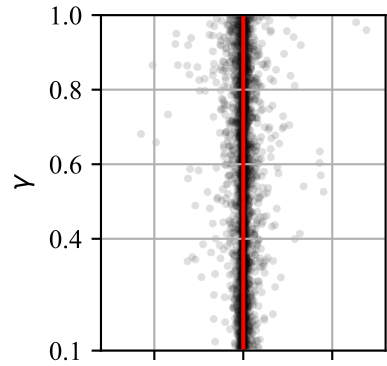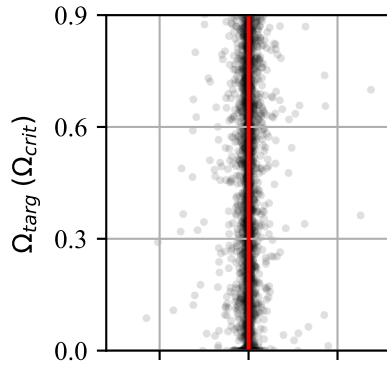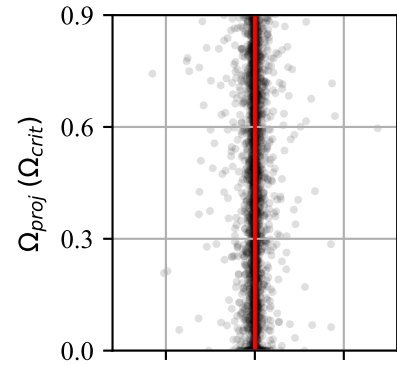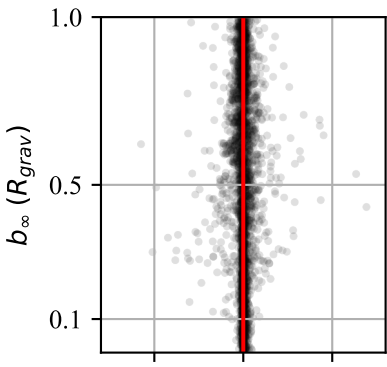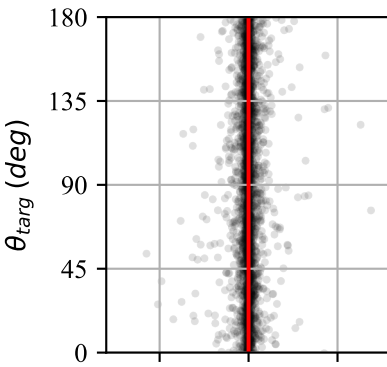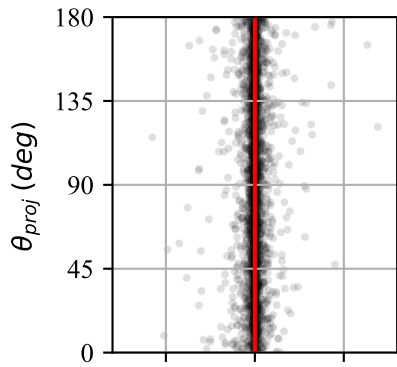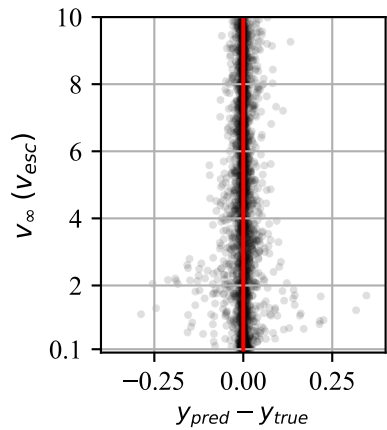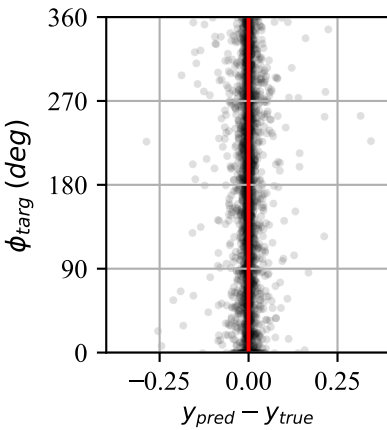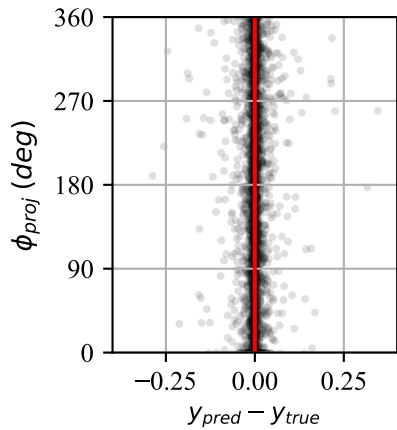

Supplement: Supplementary file 1 — Supplementary information (ZIP 48.3 MB) [file 40668_2020_34_MOESM1_ESM.zip › residuals_slr_mass_norm_xgb_11884.pdf]

Target:  $M_{\text{SLR}}$ 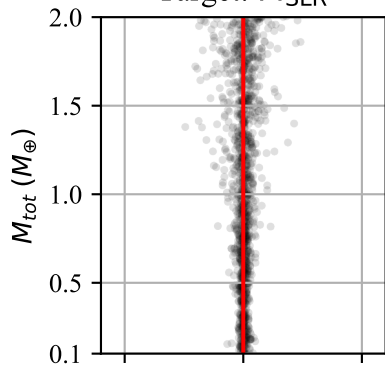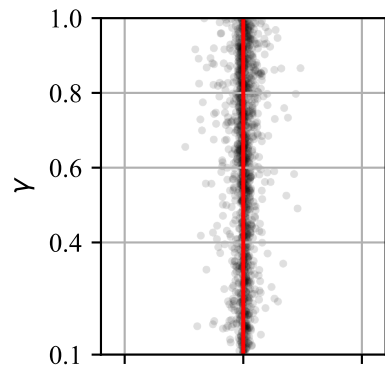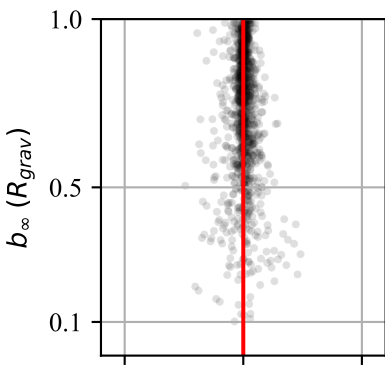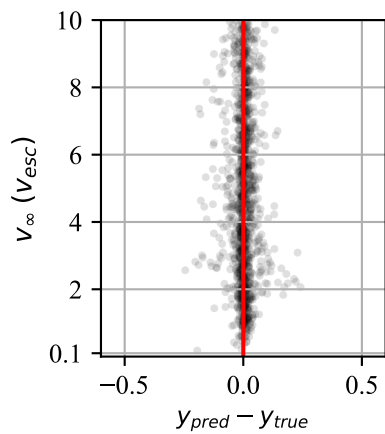

Method: PCE

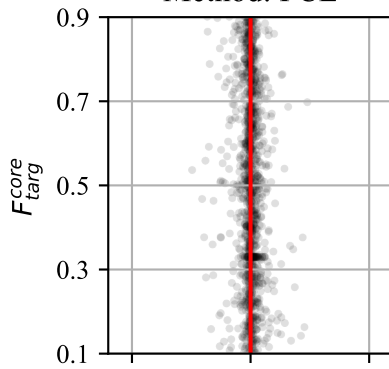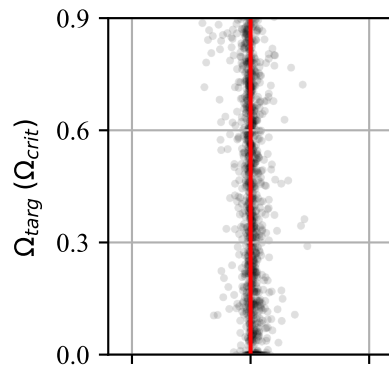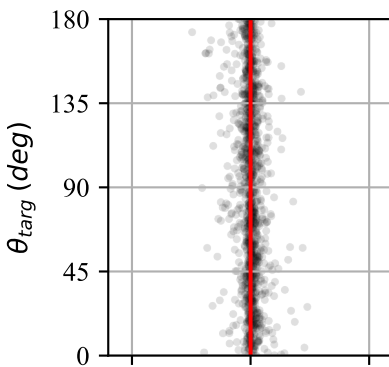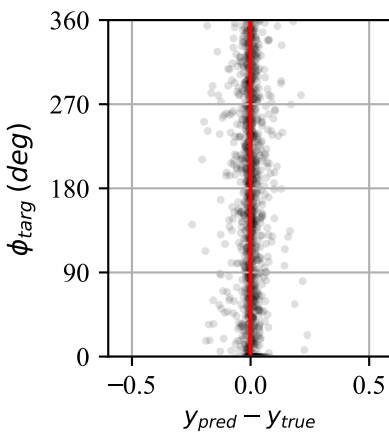

TSS = 11,884

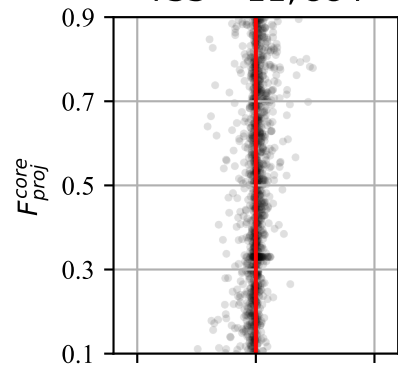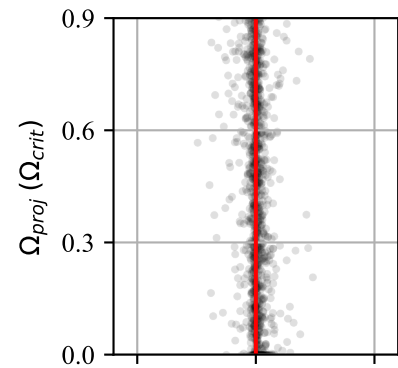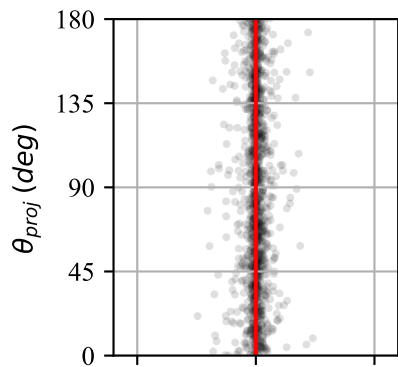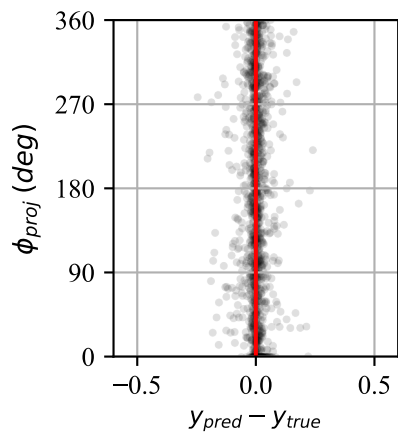

Supplement: Supplementary file 1 — Supplementary information (ZIP 48.3 MB) [file 40668_2020_34_MOESM1_ESM.zip › residuals_slr_mass_pce_11884.pdf]

Target:  $M_{\text{SLR}}$ 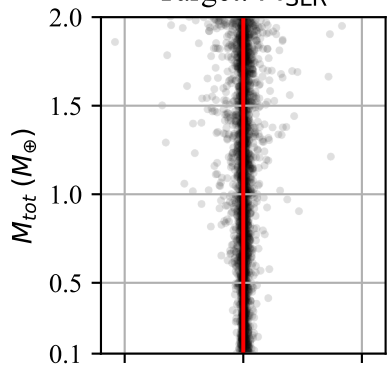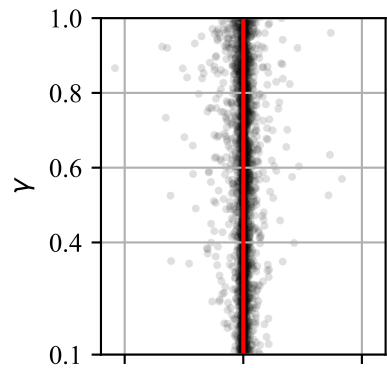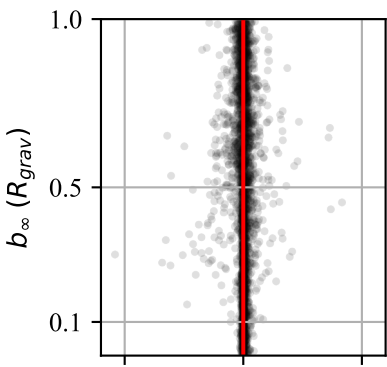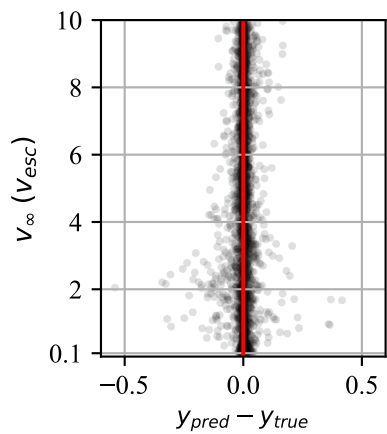

Method: XGB

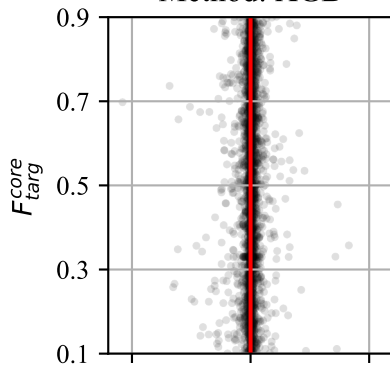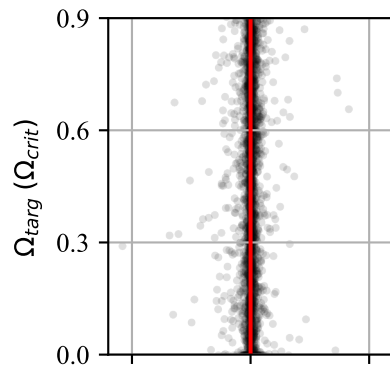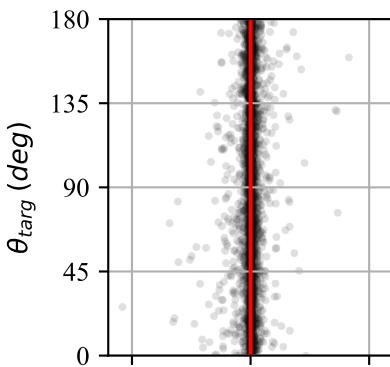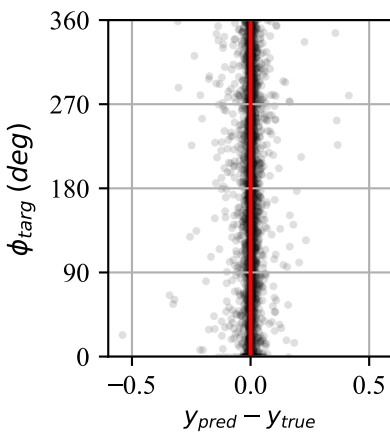

TSS = 11,884

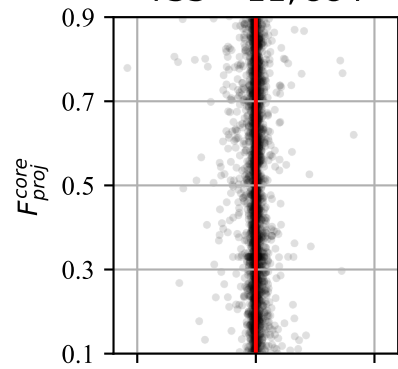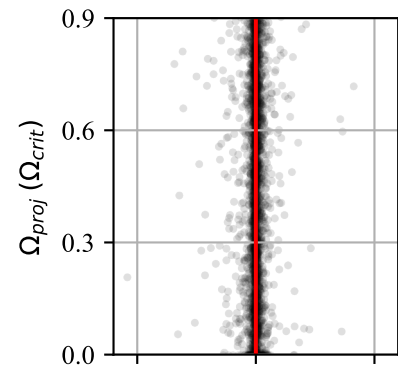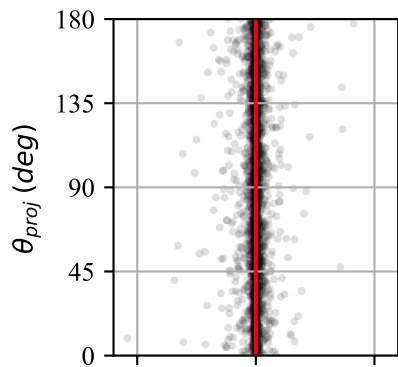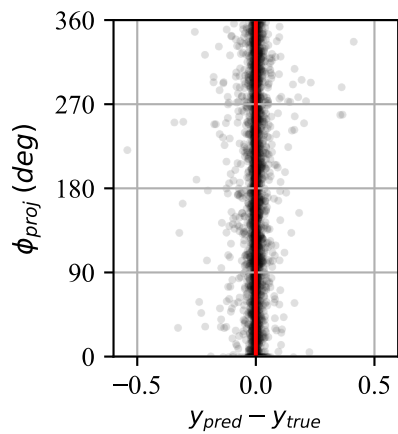

Supplement: Supplementary file 1 — Supplementary information (ZIP 48.3 MB) [file 40668_2020_34_MOESM1_ESM.zip › residuals_slr_mass_xgb_11884.pdf]

Target:  $\delta_{\text{SLR}}^{\text{mix}}$ 

Method: GP

TSS = 11,884

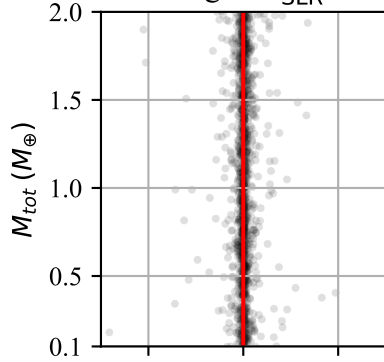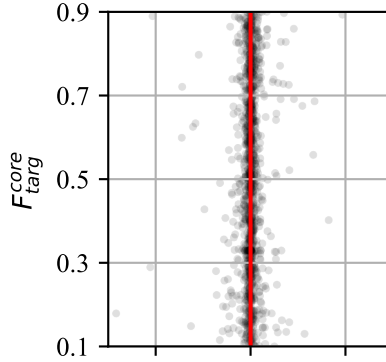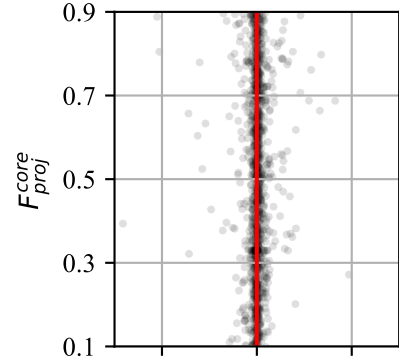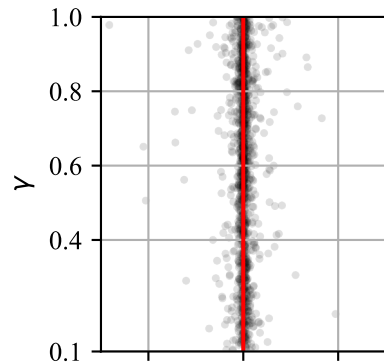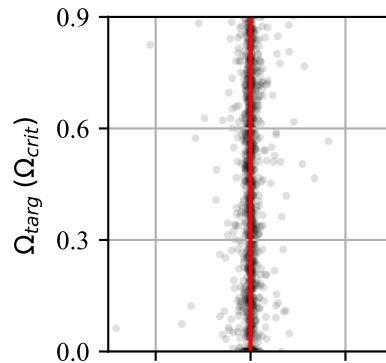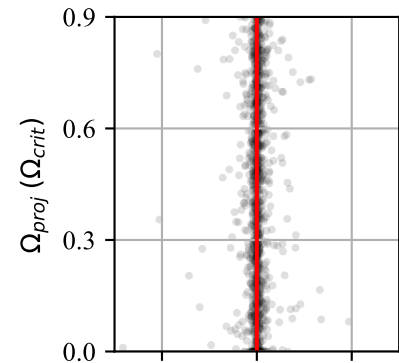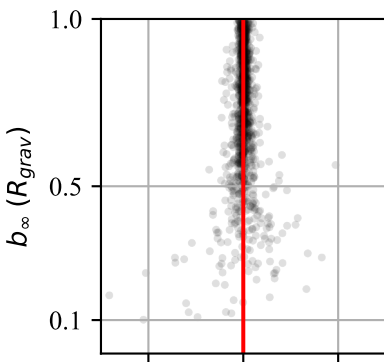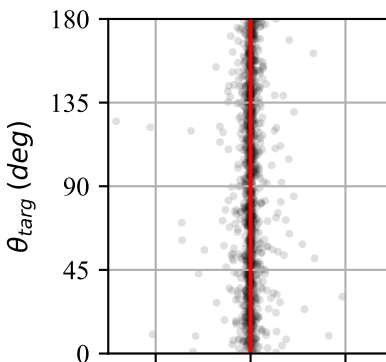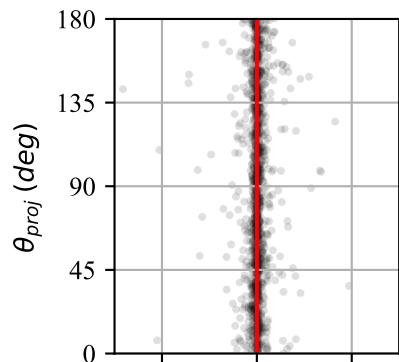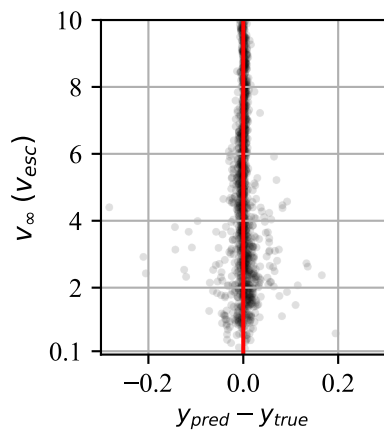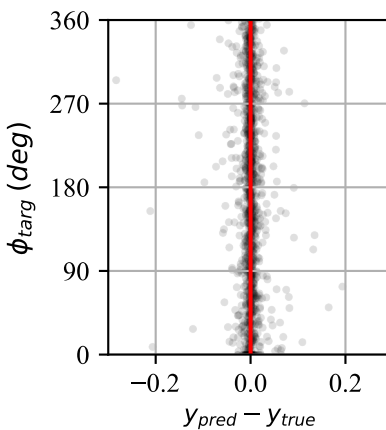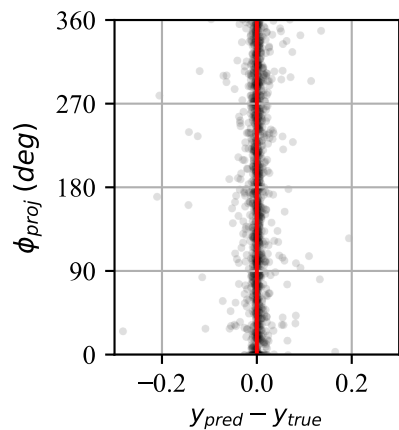

Supplement: Supplementary file 1 — Supplementary information (ZIP 48.3 MB) [file 40668_2020_34_MOESM1_ESM.zip › residuals_slr_mixing_impurity_gp_11884.pdf]

Target:  $\delta_{\text{SLR}}^{\text{mix}}$ 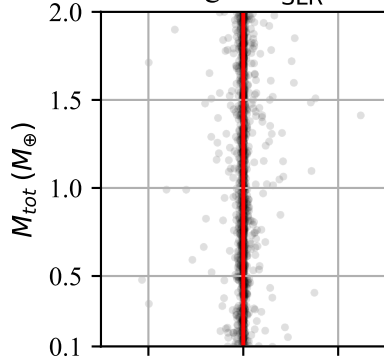

Method: MLP

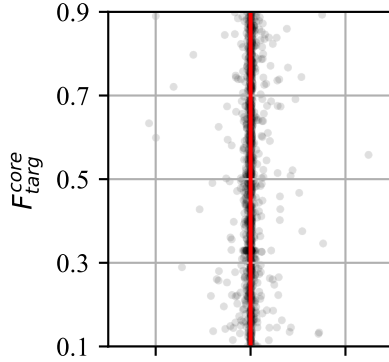

TSS = 11,884

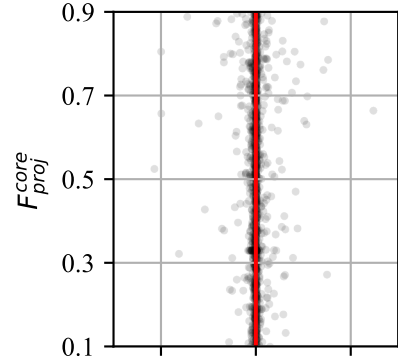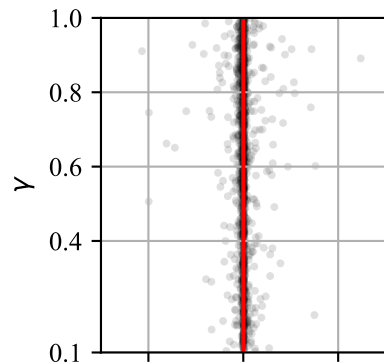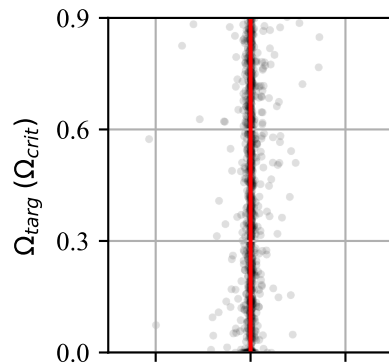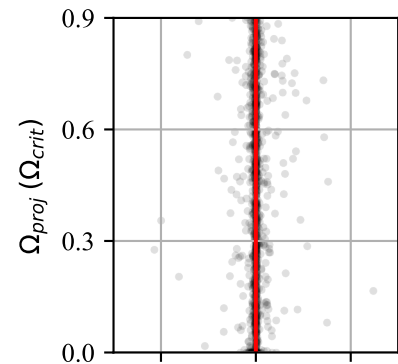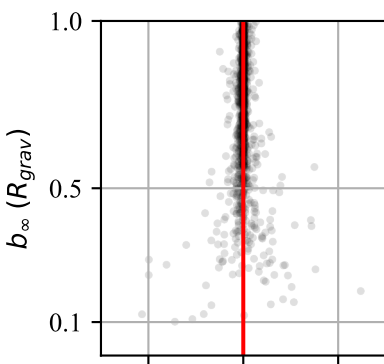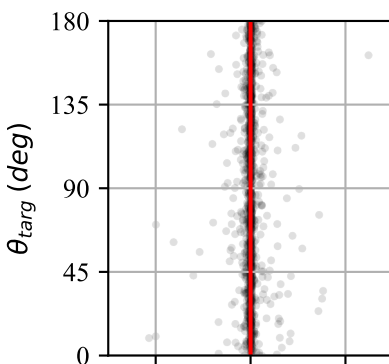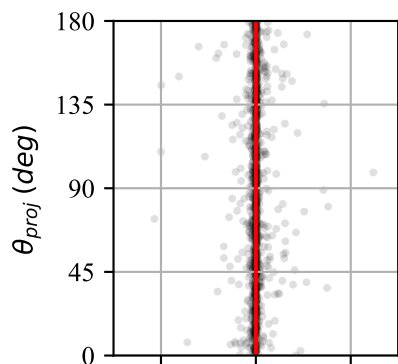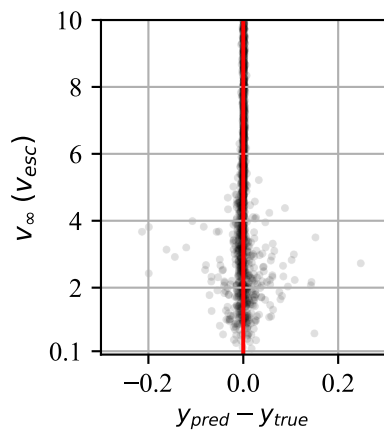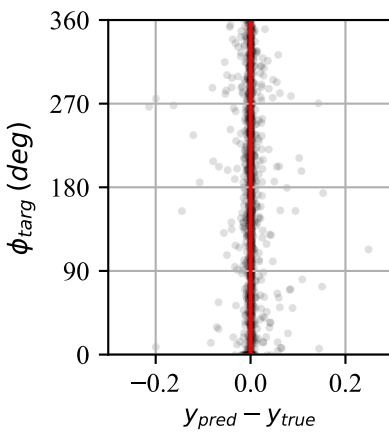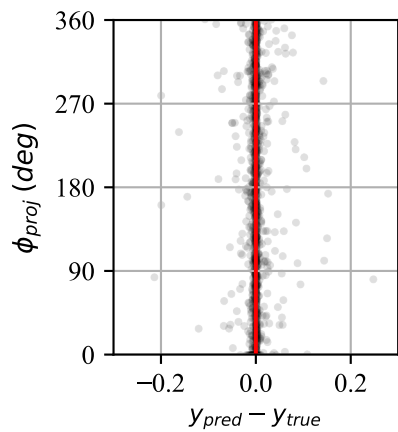

Supplement: Supplementary file 1 — Supplementary information (ZIP 48.3 MB) [file 40668_2020_34_MOESM1_ESM.zip › residuals_slr_mixing_impurity_mlp_11884.pdf]

Target:  $\delta_{\text{SLR}}^{\text{mix}}$ 

Method: PCE

TSS = 11,884

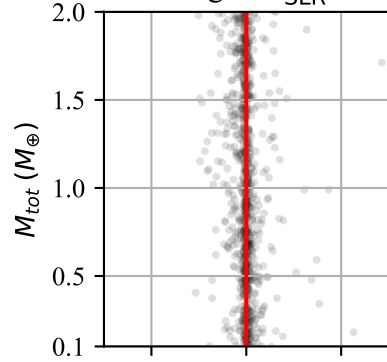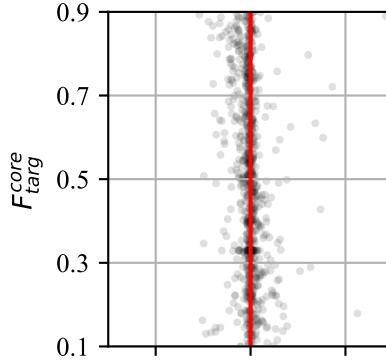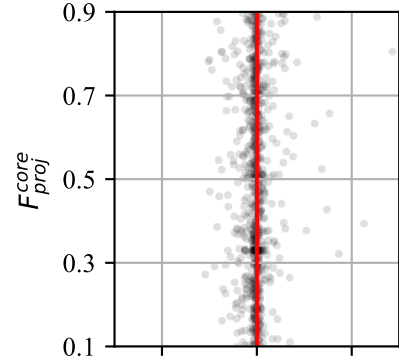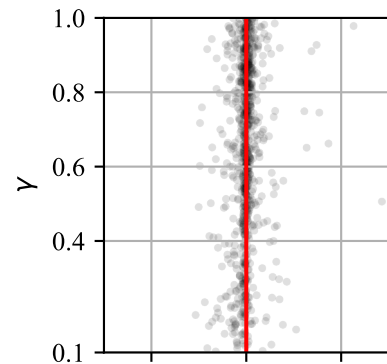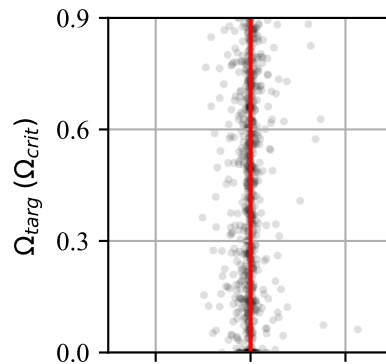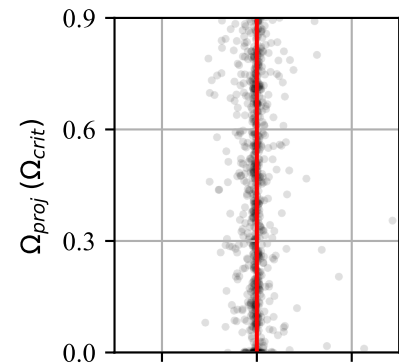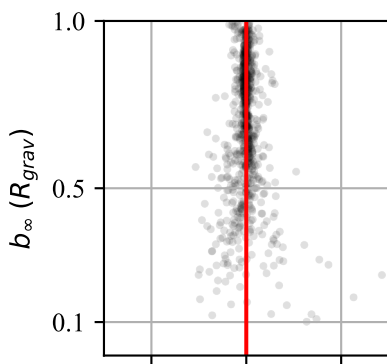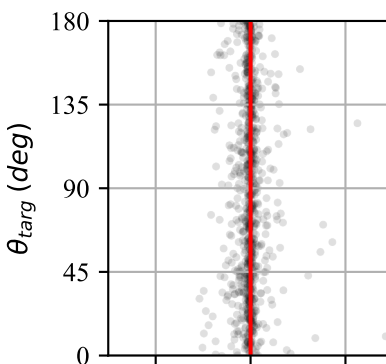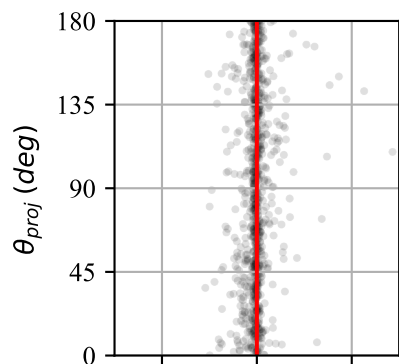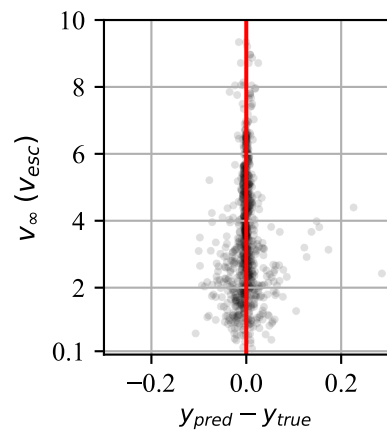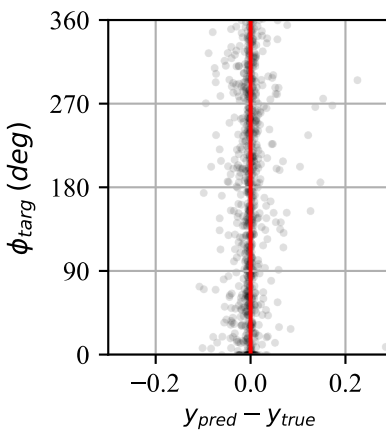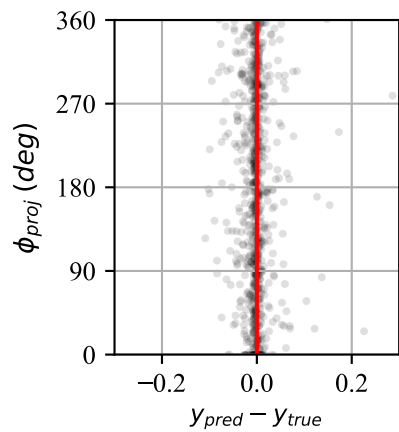

Supplement: Supplementary file 1 — Supplementary information (ZIP 48.3 MB) [file 40668_2020_34_MOESM1_ESM.zip › residuals_slr_mixing_impurity_pce_11884.pdf]

Target:  $\delta_{\text{SLR}}^{\text{mix}}$ 

Method: XGB

TSS = 11,884

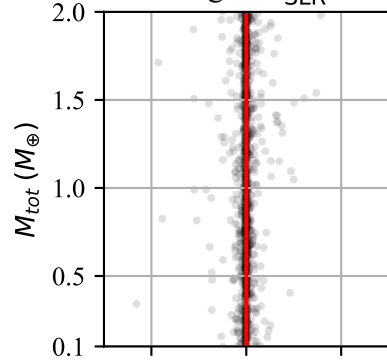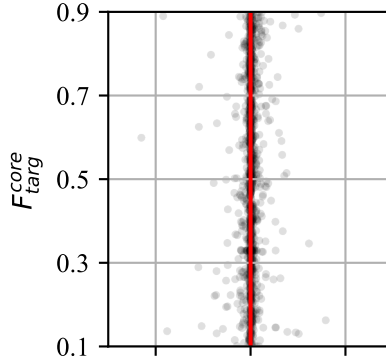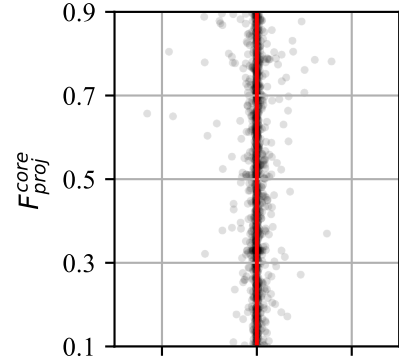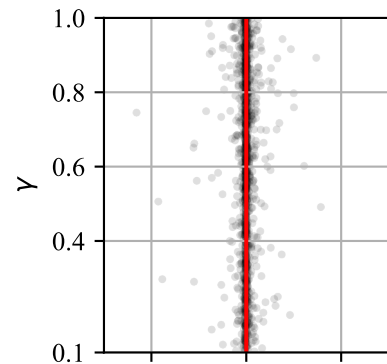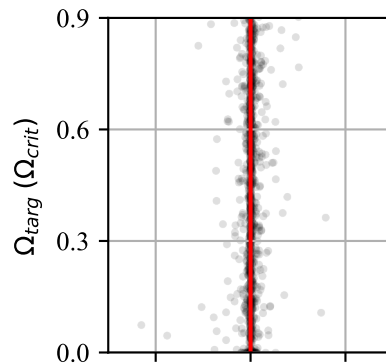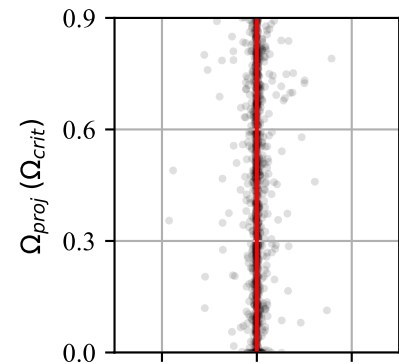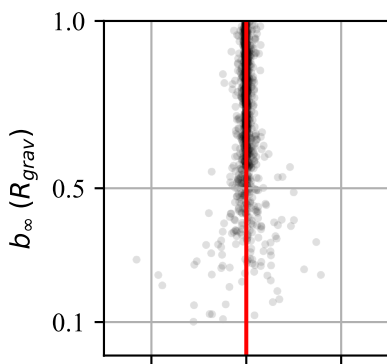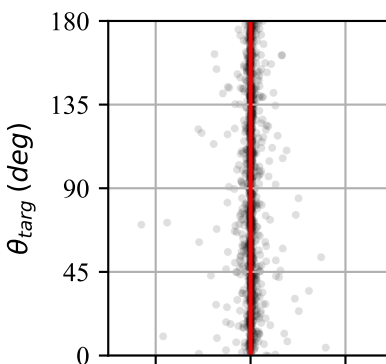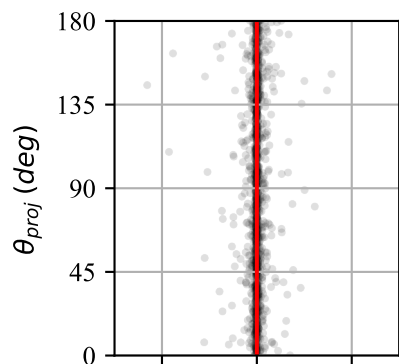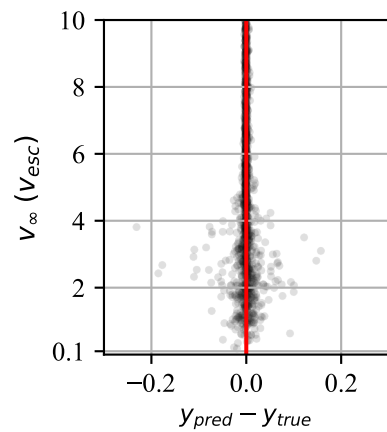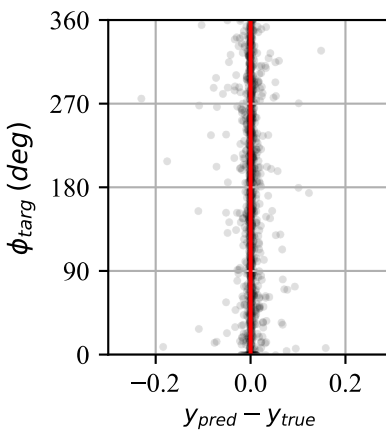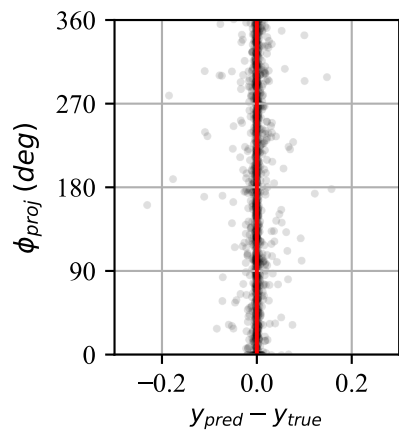

Supplement: Supplementary file 1 — Supplementary information (ZIP 48.3 MB) [file 40668_2020_34_MOESM1_ESM.zip › residuals_slr_mixing_impurity_xgb_11884.pdf]

Target:  $\Omega_{\text{SLR}}$ 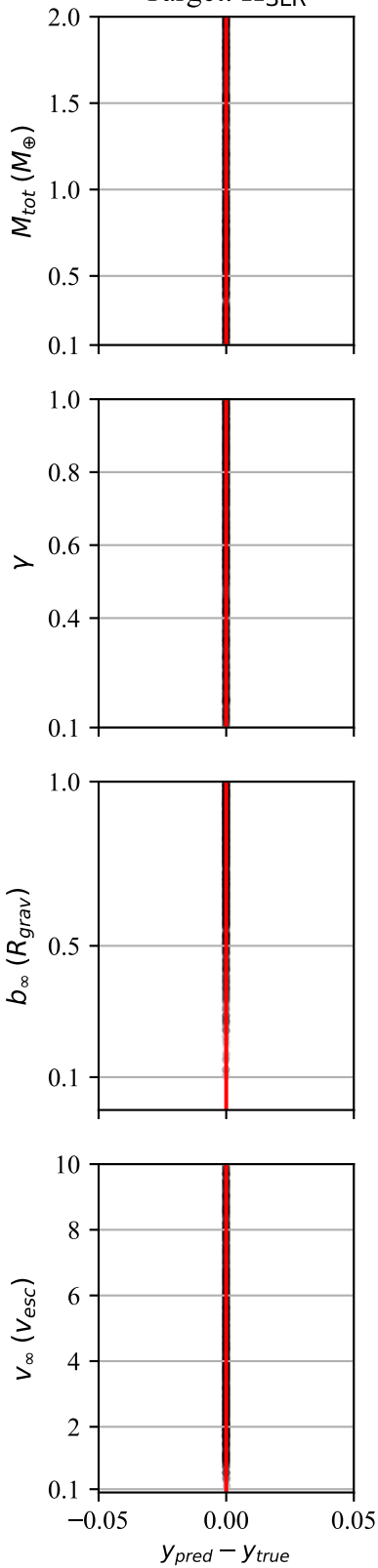

Method: GP

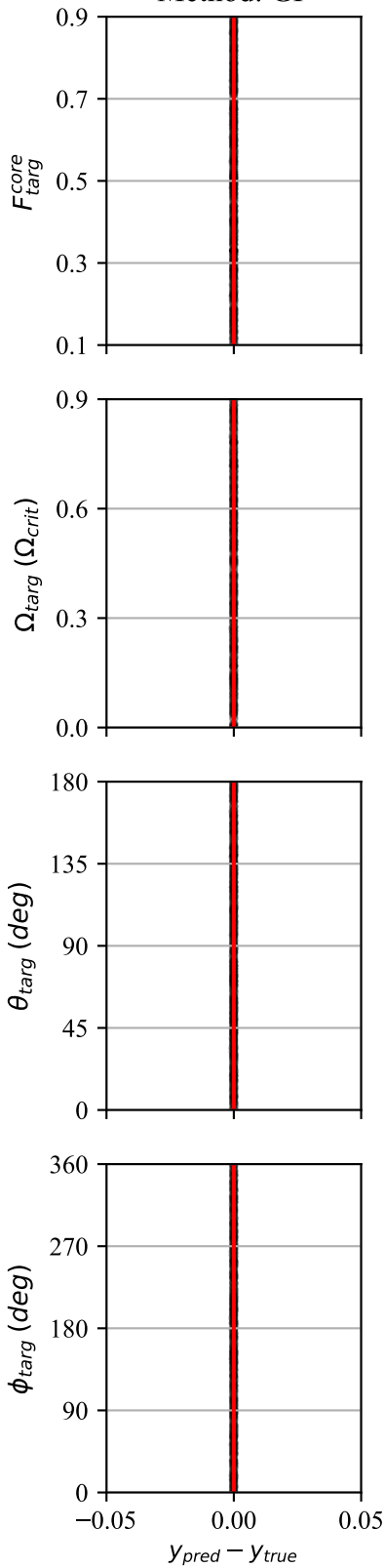

TSS = 11, 884

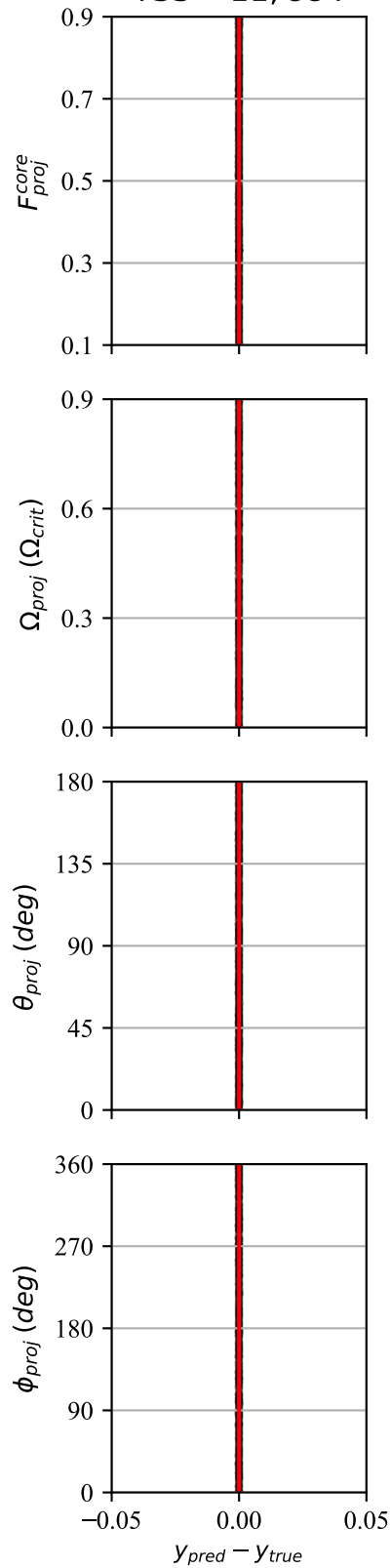

Supplement: Supplementary file 1 — Supplementary information (ZIP 48.3 MB) [file 40668_2020_34_MOESM1_ESM.zip › residuals_slr_omega_gp_11884.pdf]

Target:  $\Omega_{\text{SLR}}$ 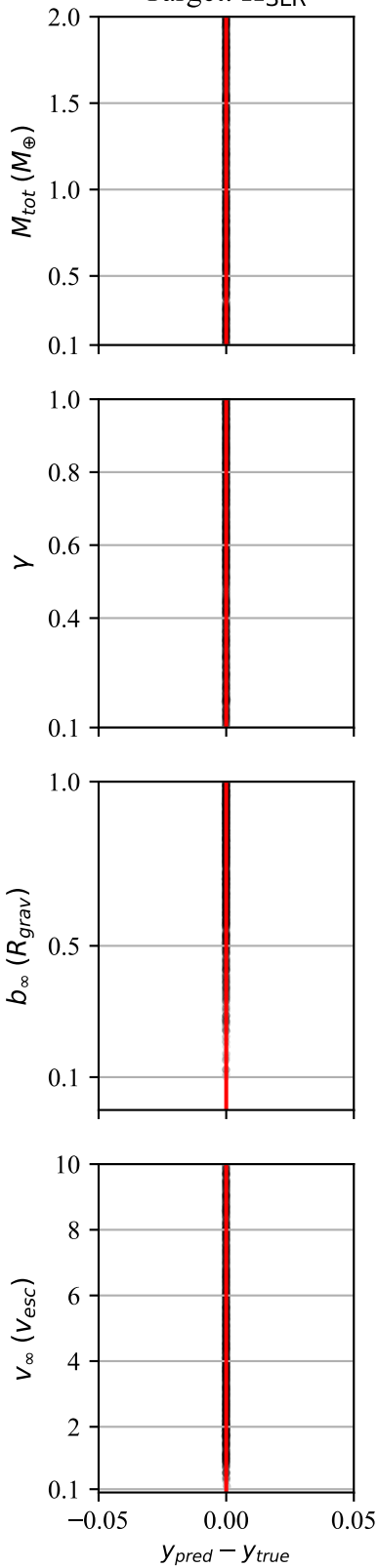

Method: MLP

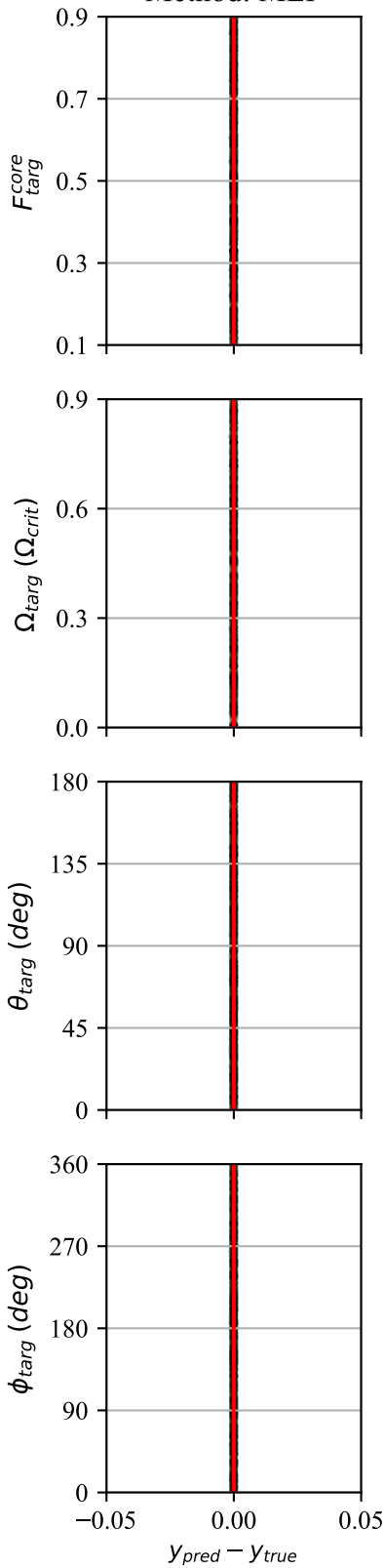

TSS = 11, 884

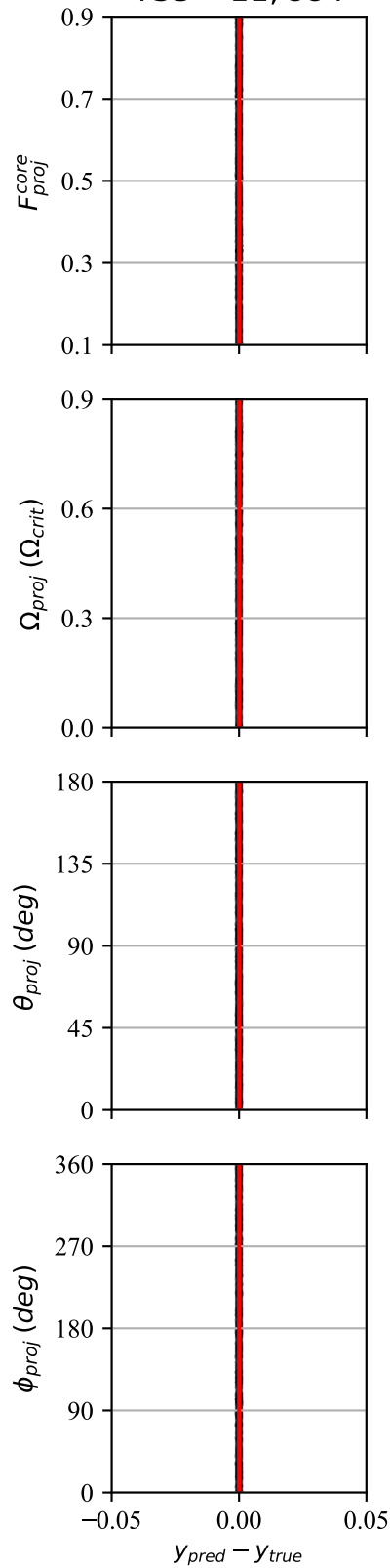

Supplement: Supplementary file 1 — Supplementary information (ZIP 48.3 MB) [file 40668_2020_34_MOESM1_ESM.zip › residuals_slr_omega_mlp_11884.pdf]

Target:  $\Omega_{\text{SLR}}$ 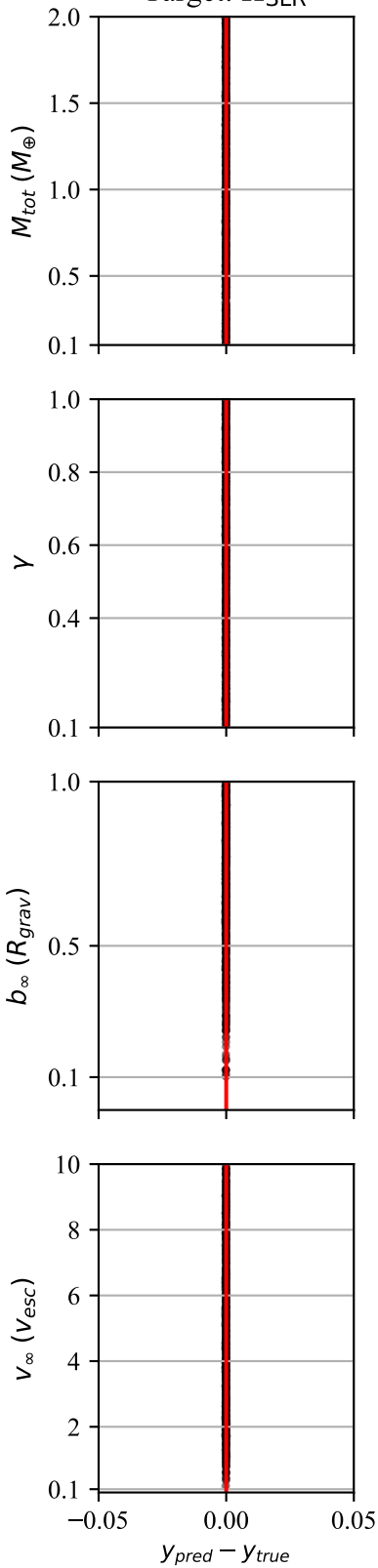

Method: PCE

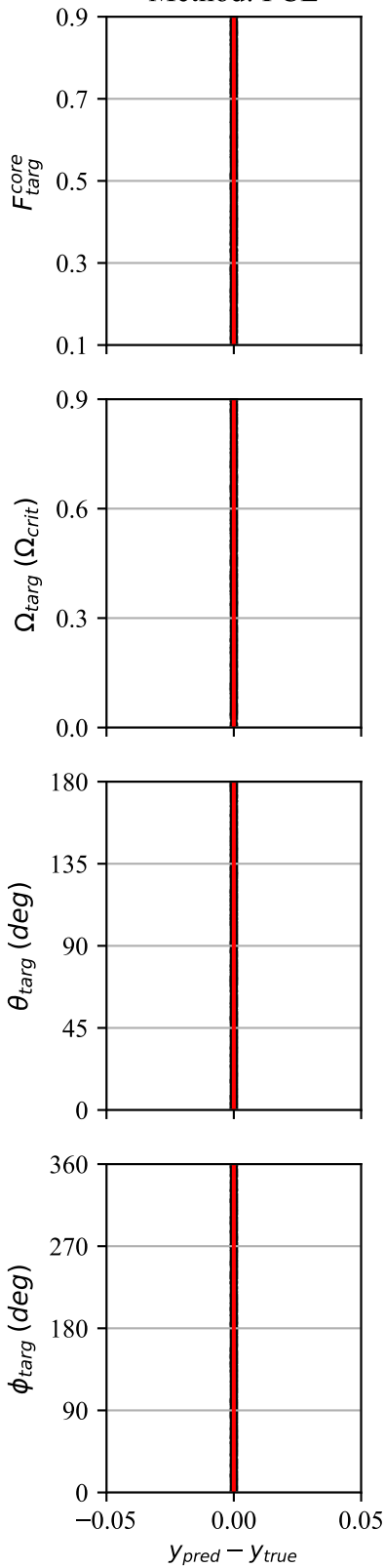

TSS = 11, 884

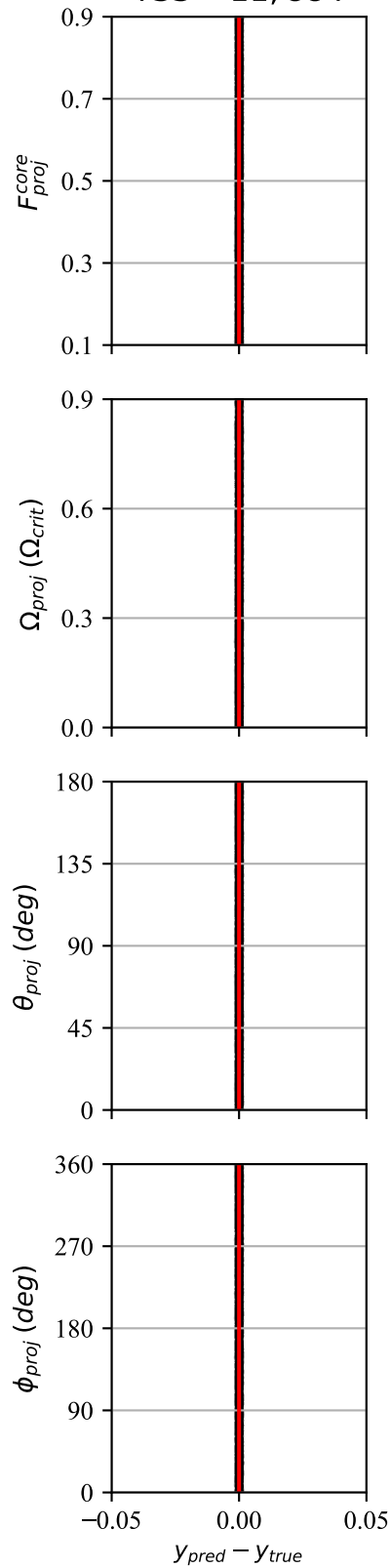

Supplement: Supplementary file 1 — Supplementary information (ZIP 48.3 MB) [file 40668_2020_34_MOESM1_ESM.zip › residuals_slr_omega_pce_11884.pdf]

Target:  $\Omega_{\text{SLR}}$ 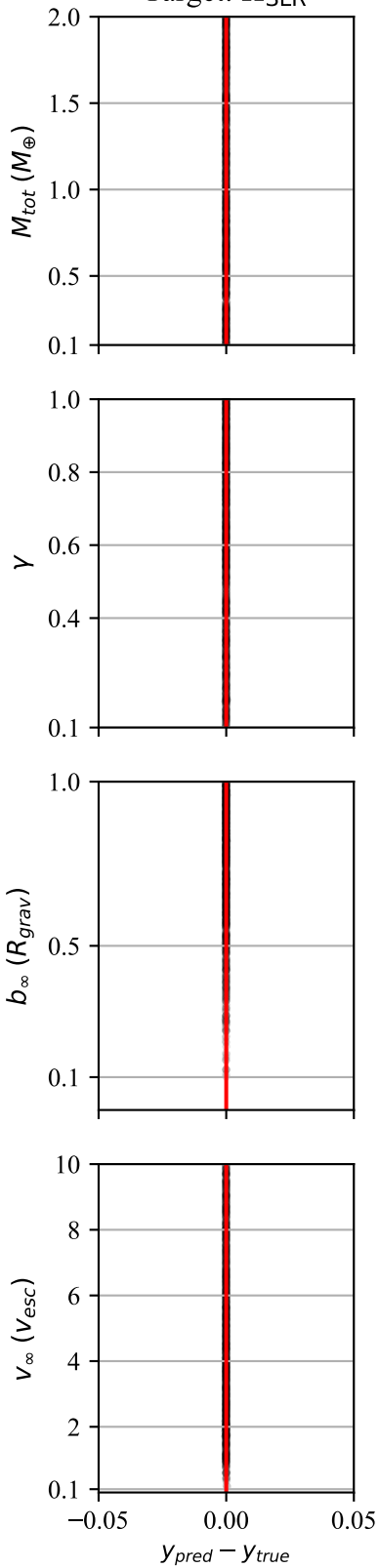

Method: XGB

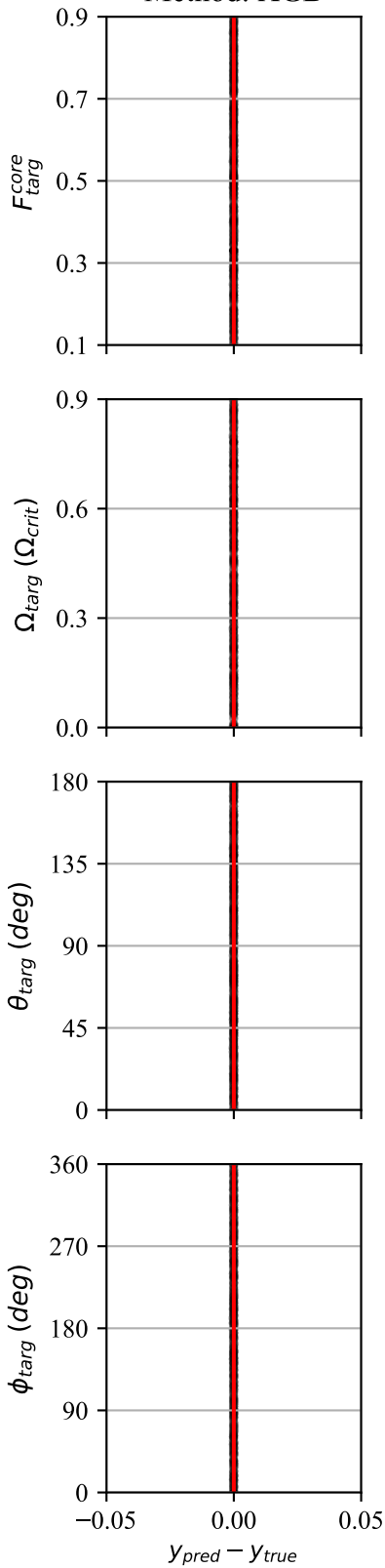

TSS = 11, 884

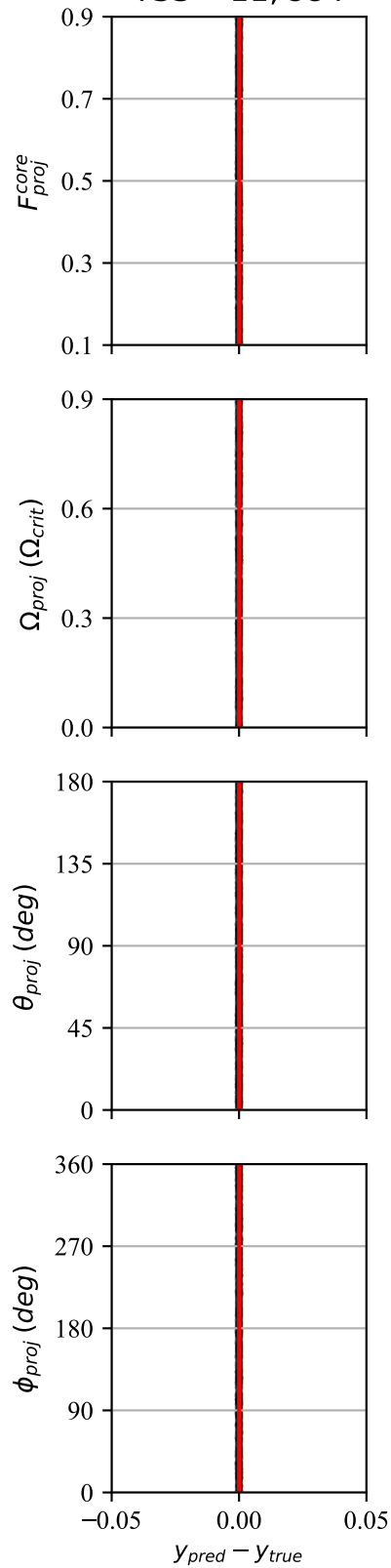

Supplement: Supplementary file 1 — Supplementary information (ZIP 48.3 MB) [file 40668_2020_34_MOESM1_ESM.zip › residuals_slr_omega_xgb_11884.pdf]

Target:  $\theta_{\text{SLR}}$ 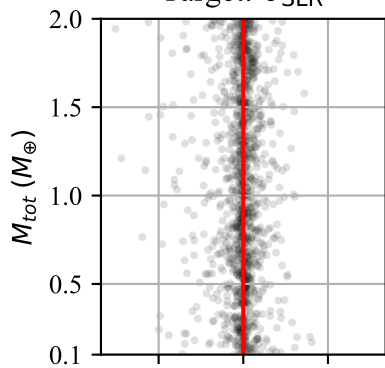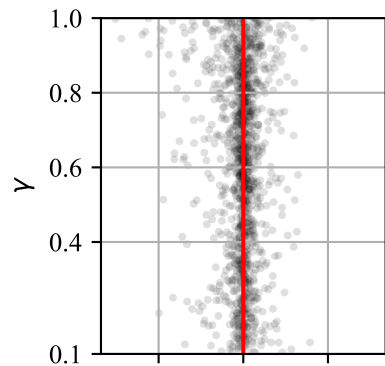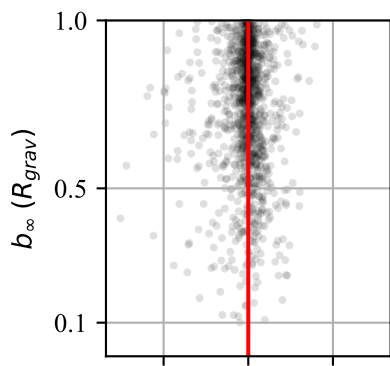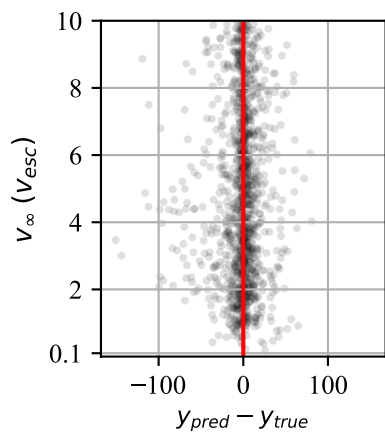

Method: GP

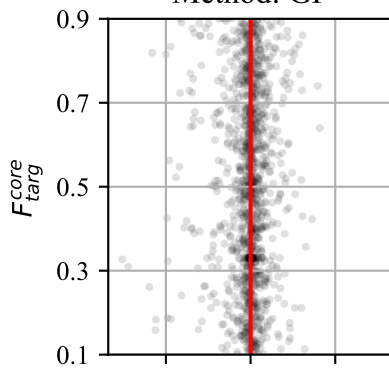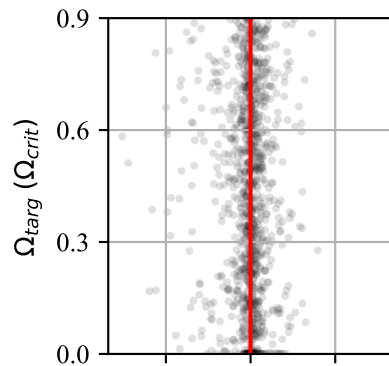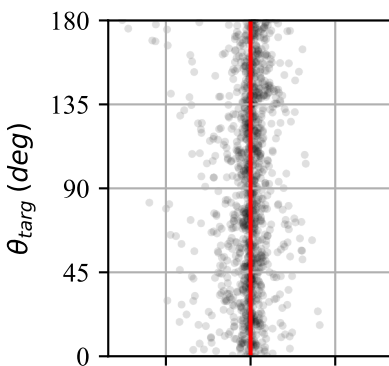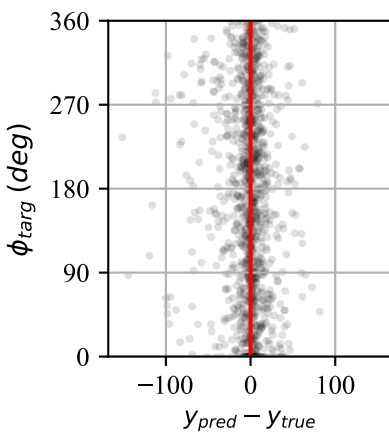

TSS = 11,884

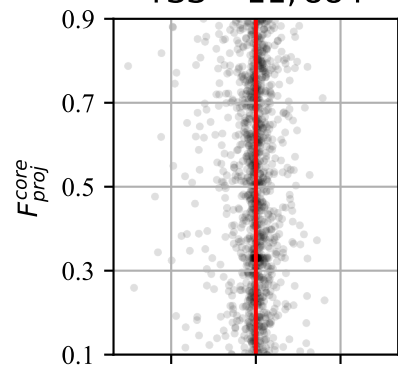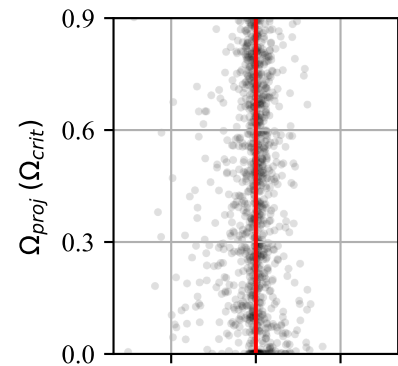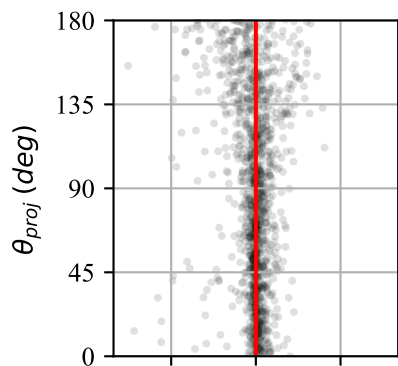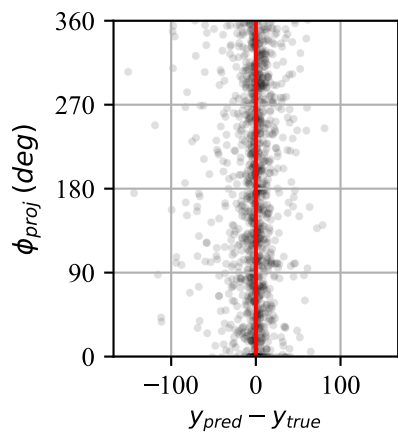

Supplement: Supplementary file 1 — Supplementary information (ZIP 48.3 MB) [file 40668_2020_34_MOESM1_ESM.zip › residuals_slr_theta_gp_11884.pdf]

Target:  $\theta_{\text{SLR}}$ 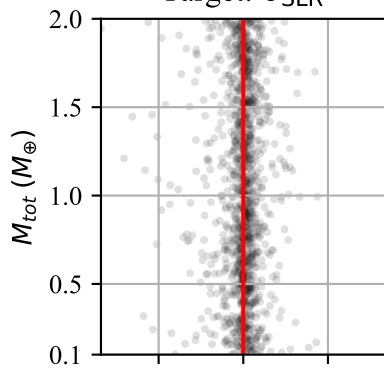

Method: MLP

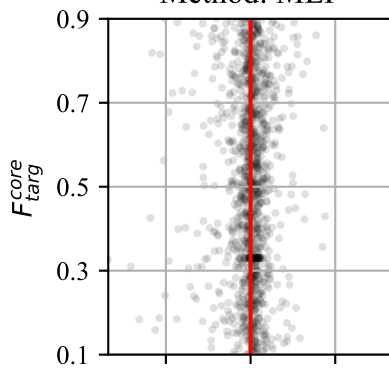

TSS = 11,884

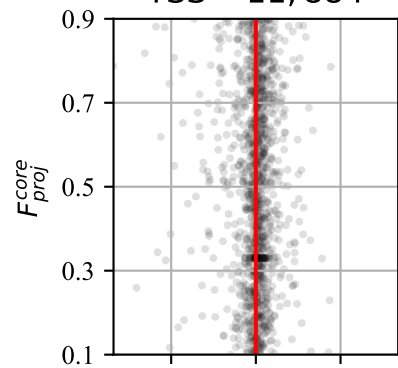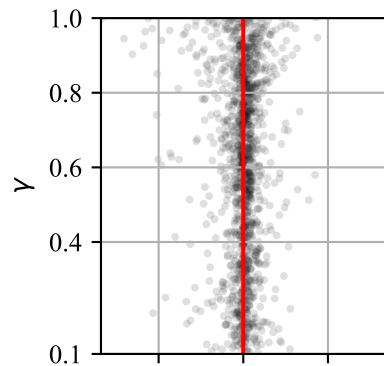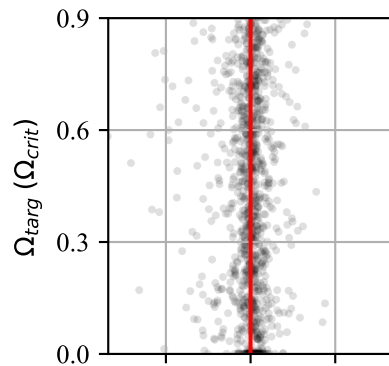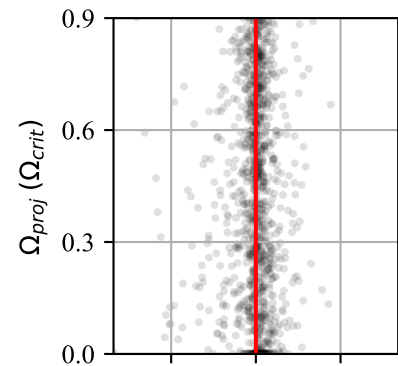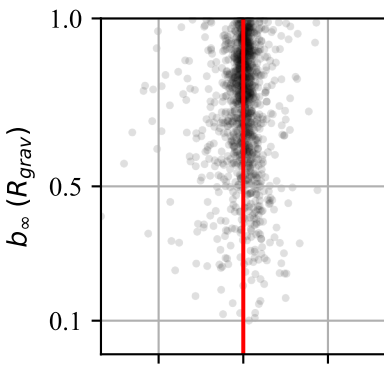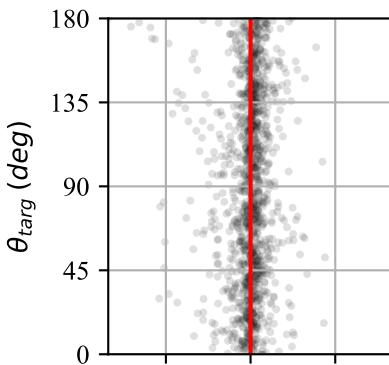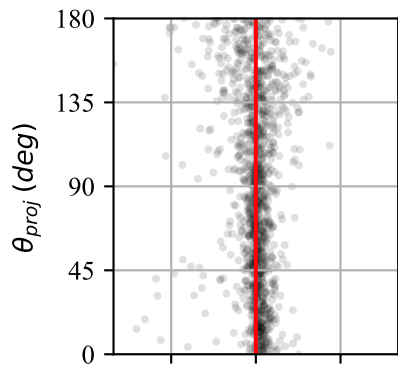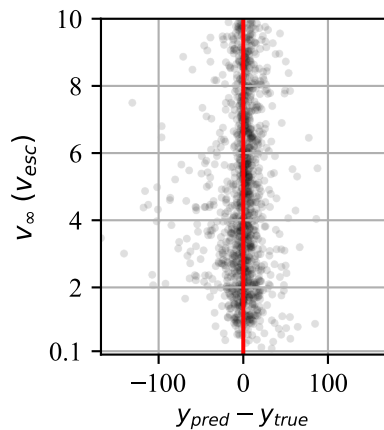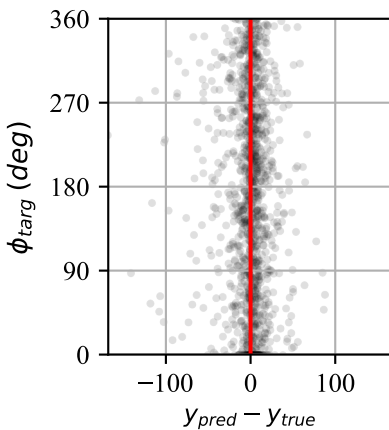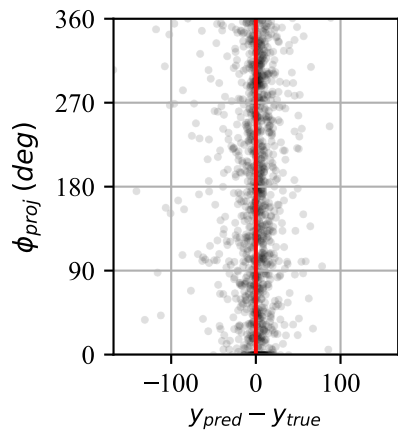

Supplement: Supplementary file 1 — Supplementary information (ZIP 48.3 MB) [file 40668_2020_34_MOESM1_ESM.zip › residuals_slr_theta_mlp_11884.pdf]

Target:  $\theta_{\text{SLR}}$ 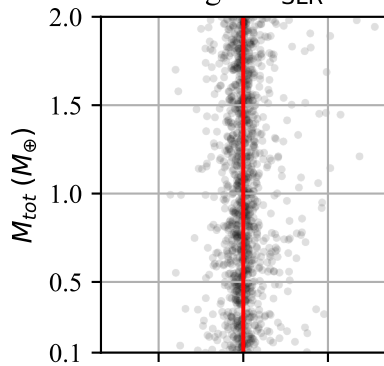

Method: PCE

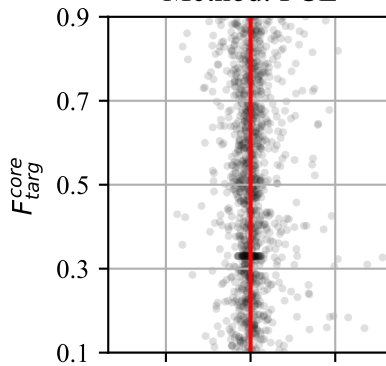

TSS = 11,884

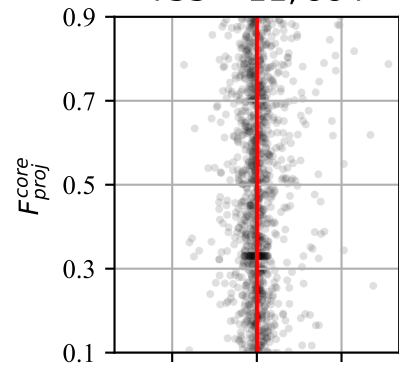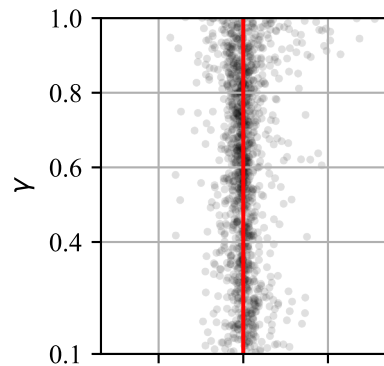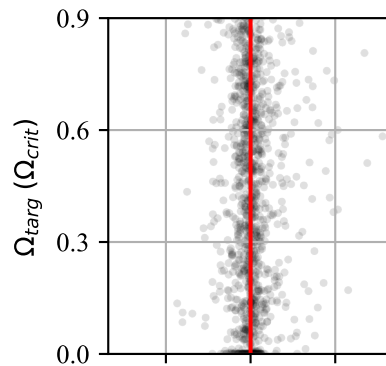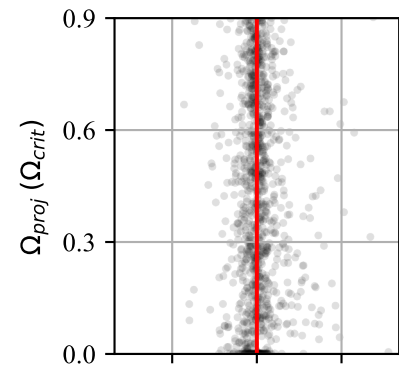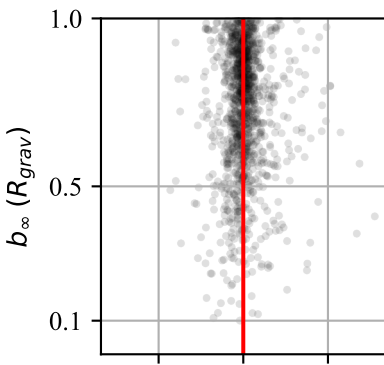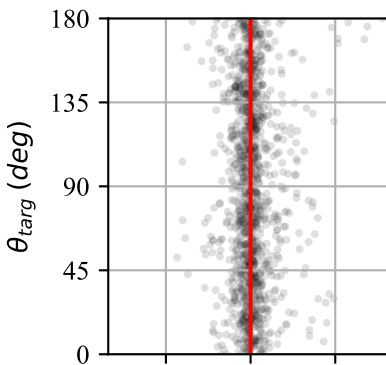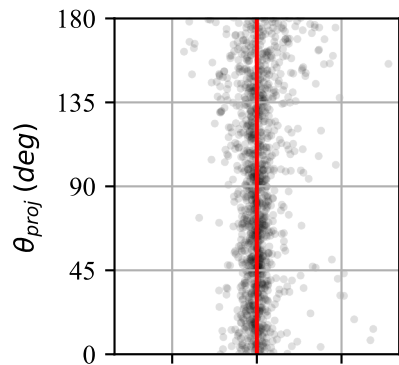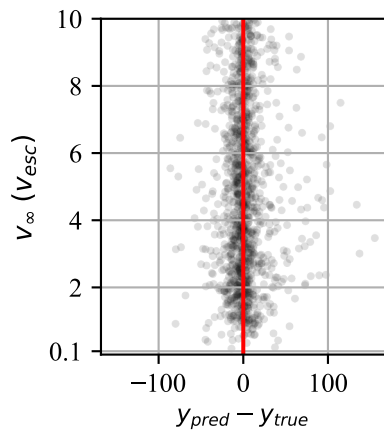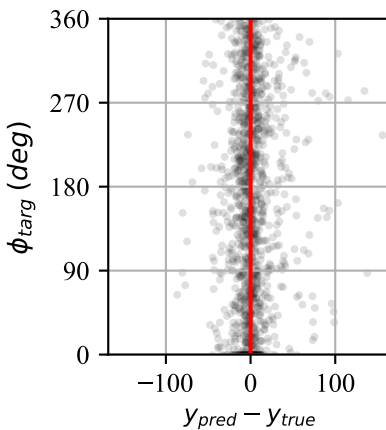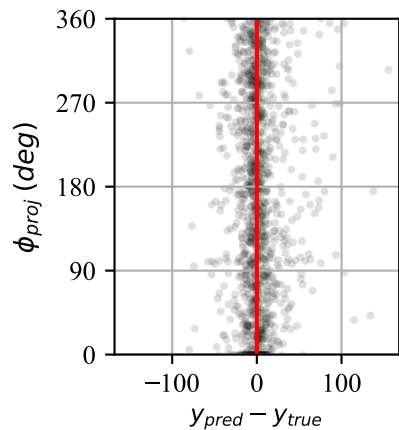

Supplement: Supplementary file 1 — Supplementary information (ZIP 48.3 MB) [file 40668_2020_34_MOESM1_ESM.zip › residuals_slr_theta_pce_11884.pdf]

Target:  $\theta_{\text{SLR}}$ 

Method: XGB

TSS = 11,884

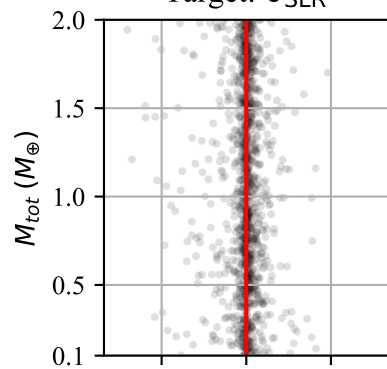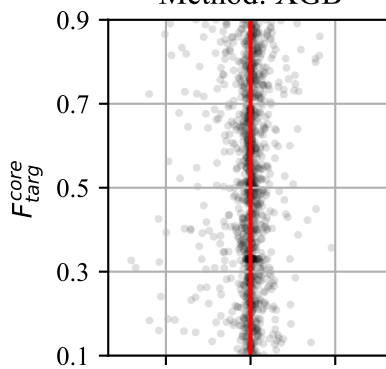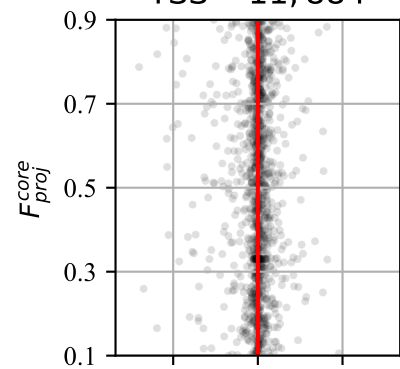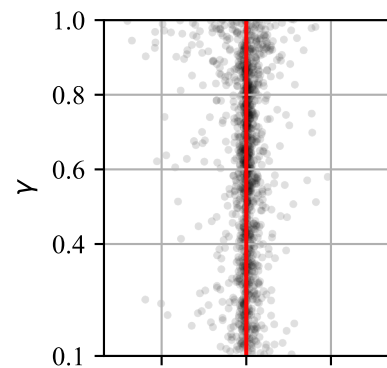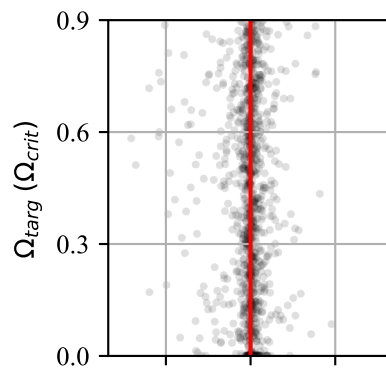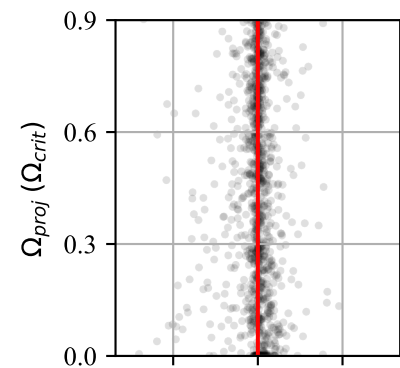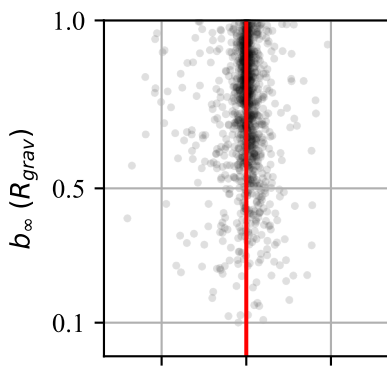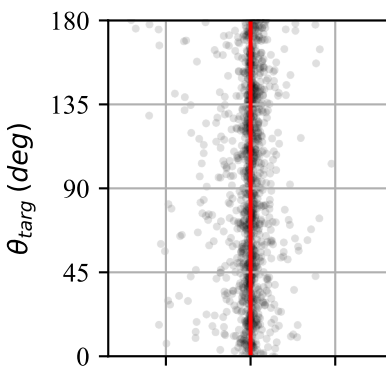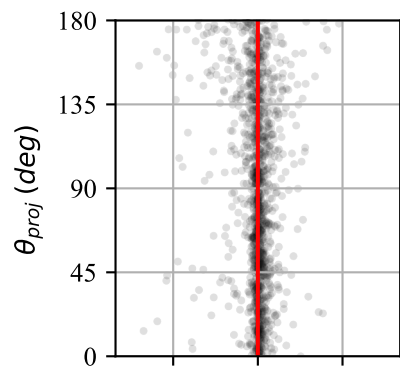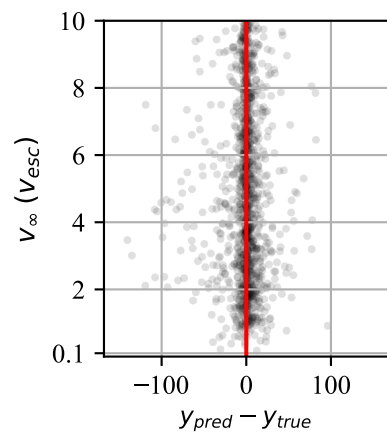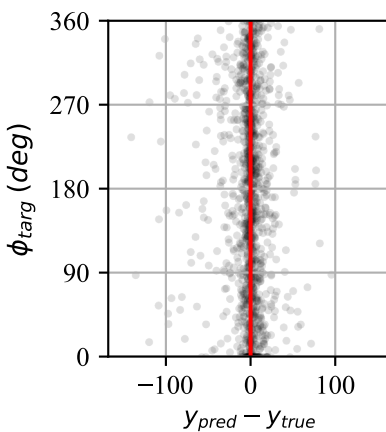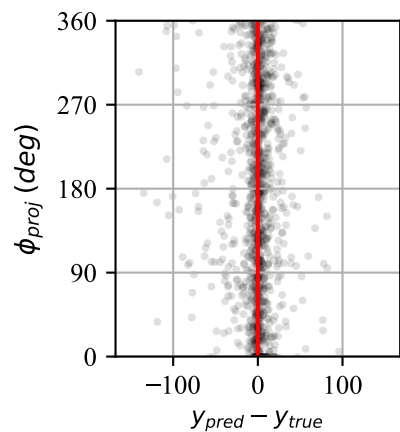

Supplement: Supplementary file 1 — Supplementary information (ZIP 48.3 MB) [file 40668_2020_34_MOESM1_ESM.zip › residuals_slr_theta_xgb_11884.pdf]
